# Decentralizing cancer care in sub-Saharan Africa through an integrated regional cancer centre model: The case of Kenya

**DOI:** 10.1371/journal.pgph.0002402

**Published:** 2023-09-22

**Authors:** Mary F. Nyangasi, Angela A. McLigeyo, David Kariuki, Siwillis Mithe, Albert Orwa, Valerian Mwenda

**Affiliations:** 1 National Cancer Control Program, Ministry of Health, Nairobi, Kenya; 2 Jaramogi Oginga Odinga Teaching and Referral Hospital, Kisumu, Kenya; 3 Nakuru Regional Cancer Centre, Nakuru, Kenya; 4 Department of Clinical Medicine and Therapeutics, University of Nairobi, Nairobi, Kenya; Makerere University, UGANDA

## Abstract

For 50 years, comprehensive cancer treatment services were provided at one public hospital and a few private facilities in the capital city. In 2019, the services were decentralized to new national and regional centers to increase service accessibility using an integration model. This study aimed to analyze the status of the utilization of services at regional cancer centers. We analyzed data from the district health information system, focusing on patient demographics, visit type, cancer stage, and the type of treatment provided. For comparison, a trend analysis of new cancer cases recorded at the main national referral hospital between 2011–2021 was also conducted. We conducted a descriptive analysis of the variables of interest; the median was used to summarize continuous variables and percentages were used for categorical variables. A total of 29,321 patients visited the regional centers in 2021; the median age was 57 years (IQR 44–68) and 57.3% (16,815) were female. Visits to regional centres represented 38.8% (29,321/75,501) of all visits to public cancer centers; new visits accounted for 16.4% (4814/29321), and the rest were follow-up visits. Most patients (71%) had an advanced disease. The proportion of male patients with advanced-stage cancer was significantly higher than that of female patients (74% vs. 69%, P<0.001). Of the 15,275 patients who received treatment at regional centers, 69.1% (10,550) received chemotherapy.The increased patient visits show good service uptake at the regional centers, implying improved access. These findings can inform policies that will guide future expansion and service improvement. We recommend optimizing cancer service delivery at regional centers across the care continuum to improve patient outcomes.

## Introduction

Cancer is a major public health concern worldwide, with an estimated 19.3 million new cancer cases and almost 10.0 million cancer deaths in 2020 [1]. In sub-Saharan Africa (SSA), there were an estimated 1.1 million new cancer cases recorded in that year as compared to 626,400 new cases in 2012 [2] indicating a rising burden of the disease. In many low- and middle-income countries (LMIC), substantial access barriers exist along the entire continuum of care [3]. While the number of absolute cases is lower than that for other continents, SSA has a mortality-incidence ratio that exceeds higher-income continents [4,5]. More than 70% of all cancer deaths globally occur in LMIC, an estimate that is projected to increase to 75% by 2030 [5]. In higher-income countries, there has been an improvement in life expectancy with increased screening and surveillance programs, and improved cancer therapies [6,7]. However, the cancer landscape in Africa is often characterized by late detection when standard treatment is ineffective, resulting in an increased risk of adverse events and high mortality [8–10].

Kenya, like many SSA countries, is experiencing an epidemiological transition, with a declining burden of communicable diseases and rising burden of cancer. In 2020, the country recorded more than 42,000 new cancer cases [1]. Cancer is also an increasing contributor to overall mortality, from 3% in 2000 to 8% in 2019, with an estimated 27,000 deaths by 2020 [1,11]. It has also been identified as a major cause of catastrophic health expenditures, impacting financial security and sustainable development [12].

Comprehensive cancer centers can be standalone facilities or units integrated within existing health facilities [13,14] that provide other related services. Although national cancer control programs address the functions and delivery of many components of cancer control, such as prevention, early detection, diagnosis, treatment, and palliation, the delivery of most of these services is anchored in comprehensive cancer centers [13]. Investing in these facilities is pertinent in ensuring service delivery, economic growth, and achieving sustainable development goals (SDGs). For 50 years, comprehensive cancer management services in Kenya had only been offered at one public national referral hospital and later on in a limited number of private health facilities concentrated within the capital city of Nairobi, with the country generally having limited capacity to deliver cancer services. The decentralization of cancer services not only increases access to care but also results in a reduction in morbidity and mortality [15–17]. Therefore, in line with the Universal Health Coverage agenda, in 2019, the Ministry of Health, through the National Cancer Control Program in collaboration with county governments, decentralized cancer treatment services to twelve regional cancer centers and an additional two national referral hospitals. These were established through integration of cancer treatment services in facilities that already had existing capacity for pathology, radiology, palliative care, gynecology, pediatrics and surgery among other multidisciplinary services relevant for cancer management. We described the sociodemographic patterns, patient visits, availability of diagnostic results and staging, treatment modalities received, and reasons for referral from the cancer centers following the decentralization of treatment services.

## Methods

### Study design, setting, and population

This was a descriptive study based on secondary aggregated data from cancer centers between January and December 2021. Data from the hospital-based cancer registry (HBCR) at the main national referral hospital was also reviewed for comparison. The study population comprised all patients who visited the 12 regional cancer centers, as well as the referral hospital.

### Data sources

Data were extracted from the district health information system (DHIS2). The cancer centers summarize information for all patients seen monthly and uploaded this data to the DHIS2. The second data source was the hospital-based cancer registry at the main national referral hospital, which collected key variables for all patients managed at the facility.

### Study variables

DHIS2 aggregates information on patient visit type, histological diagnosis and staging, HIV results, treatment modalities provided, palliative care provision, and referral. The HBCR collected sociodemographic details, tumor details, treatment, follow-up, and outcome information; only the cancer incidence date and type were utilized in this analysis. Patient visits were categorized as new or follow-up. Stage at cancer presentation was classified as I, II, III, or IV based on the globally recognized TNM classification [18]. The treatment modalities received at cancer centers were categorized as chemotherapy, radiotherapy, surgery, palliative care, and nuclear medicine. Reasons for referral from the regional cancer centers included chemotherapy, radiotherapy, nuclear medicine, or other specified purposes.

### Data management

Data was exported into Microsoft Excel sheets, reviewed for consistency and completeness, and analyzed. Frequencies are presented as absolute values and percentages. Categorical data are summarized as graphs, frequency charts, proportions, and tables, followed by associations using the chi-square test. Associations between categorical variables and patient visits were assessed using contingency tables and chi-squared tests at a 5% level of significance. Data analysis was performed using Stata 17 software (StataCorp. College Station, TX, USA).

### Ethical considerations

The data analyzed from DHIS2 are publicly available service statistics data used for programmatic purposes and for planning, both nationally and sub-nationally; this did not include any personal/case-level information. Data from the HBCR at the national referral hospital were de-identified before analysis. Given the nature of this data, a waiver of consent was obtained through the Health Management and Information Services Unit at the Ministry of Health.

## Results

### Sociodemographic characteristics

A total of 29,321 patients visited regional cancer centers in 2021, with the majority being female 16,815 (57.3%). The median patient age was 57 years (IQR 44–68). The age distribution among female patients peaked at 40–59 years while that for males peaked at 60 years and above. Approximately 3% of all the patients were pediatric cancer patients (0–19 years). There were significant differences between the numbers of male and female patients aged ≥ 30 years as shown in Table 1.

**Table 1:** Sociodemographic characteristics of patients visiting the Regional Cancer Centres in 2021.

	Female (n = 16,815)	Male (n = 12,506)	Total (n = 29,321)	P-value
**Median Age (Years [IQR])**	53 [41–63]	62 [51–72]	57 [44–68]	<0.001*
**Age Group (Years)**	**n (%)**	**n (%)**	**n (%)**	
<10	120 (0.7)	111 (0.9)	231 (0.8)	0.864
10–19	350 (2.1)	297 (2.4)	647 (2.2)	0.797
20–29	730 (4.3)	492 (3.9)	1,222 (4.2)	0.731
30–39	2,413 (14.4)	739 (5.9)	3,152 (10.7)	<0.001*
40–49	3,606 (21.4)	1,238 (9.9)	4,844 (16.5)	<0.001*
50–59	4,154 (24.7)	2,305 (18.4)	6,459 (22.0)	<0.001*
60–69	3,195 (19.0)	3,319 (26.5)	6,514 (22.2)	<0.001*
70+	2,247 (13.4)	4,005 (32.0)	6,252 (21.3)	<0.001*

* Significant at the 0.05 level; Mann-Whitney test and Chi-Square tests of association were used to compute the p-value.

### Number of patient visits

**Fig 1:**
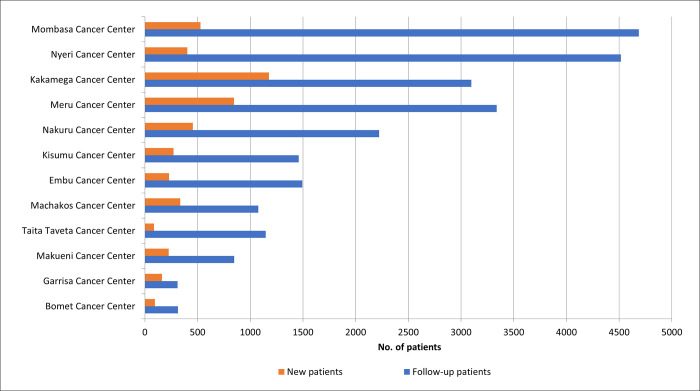
Distribution of Patient Visits by Regional Cancer Centers in 2021 (n = 29,321).

Patient visits to regional cancer centers accounted for 38.8% (29,321/75,501) of the total number of cancer visits. Of the 29,321 patient visits recorded at the regional centers, 16.4% (4814/29321) were new (Fig 1). The Mombasa Cancer Centre had the highest number of visits, at 17.8% (5214/29321) as shown in Fig 1.

**Fig 2:**
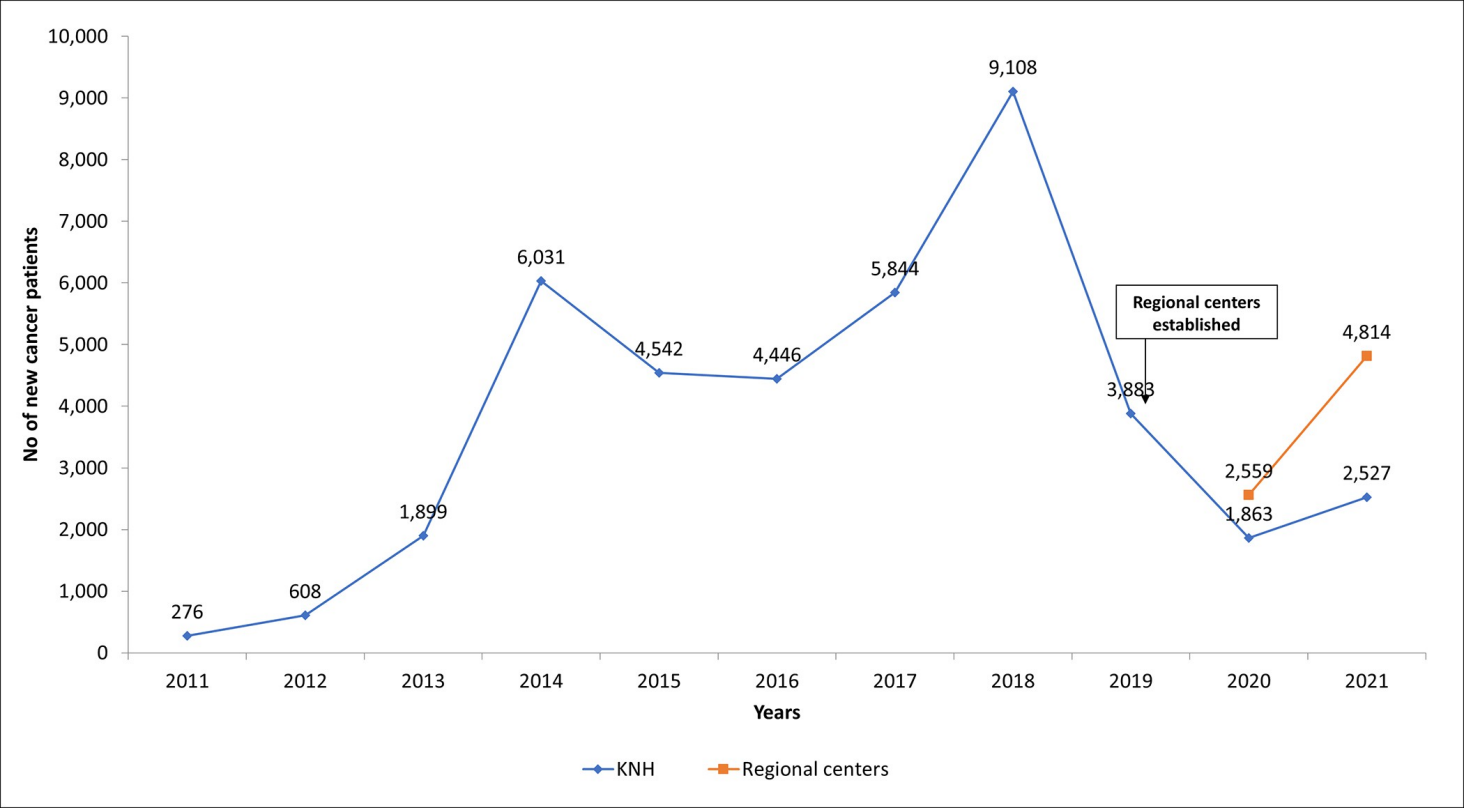
Trends of new cancer patients at the main referral hospital (2011 to 2021) and the regional cancer centers (2020–2021).

Between 2018 and 2020, a drop in the number of patients registered in the main national referral hospital cancer registry was observed, with a corresponding increase in new patients seen at regional centers from 2020 as shown in Fig 2.

### Cancer staging information

Only approximately 36% (10,533/29,321) of the patients had stage information recorded with 38.4% in stage 4, 32.8% in stage 3, 20.0% in stage 2, and 9.1% in stage 1. In this study, we explored the relationship between gender and cancer stage. Table 2 shows the significant association between sex and cancer stage. The proportion of male patients with advanced disease was significantly higher than that of female patients (74% vs. 69%, P<0.001).

**Table 2:** Cancer staging by gender.

Stage	Female (N = 6,255) n (%)	Male (N = 4,278) n (%)	Total (N = 10,533) n (%)	P-value
Stage 1 & 2	1928 (31%)	1106 (26%)	3034 (29%)	<0.001
Stage 3 & 4	4327 (69%)	3172 (74%)	7499 (71%)	<0.001

**Fig 3:**
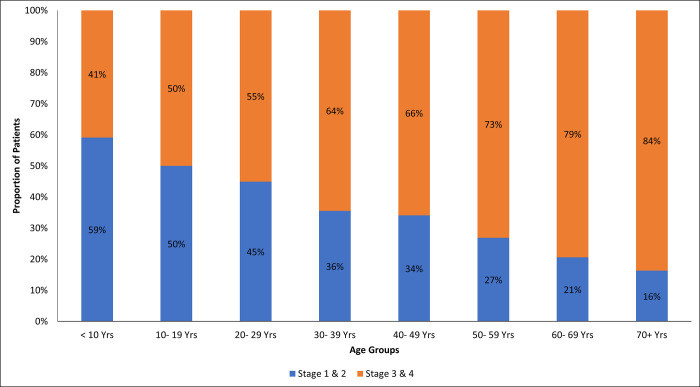
Cancer stage by age group at the regional cancer centers, 2021.

The proportion of patients with advanced-stage disease increased steadily with advancing age, being the lowest in patients aged less than ten years and the highest in those aged ≥ 70 years as shown in Fig 3.

We found significant differences in the cancer stage distribution across the various centers (P < 0.001). Kisumu and Machakos had more than half of the cancer patients presenting in the early stages, while the rest of the centers had most patients presenting in the advanced stages as in Table 3.

**Table 3:** Cancer staging distribution by cancer center.

Cancer Centre	Cancer stage		P-value
	Stage 1 & 2 n (%)	Stage 3 & 44 n (%)	
Mombasa (N = 5004)	2163 (43.2)	2841 (56.8)	< 0.001*
Kakamega (N = 1349)	141 (10.5)	1208 (89.5)	< 0.001*
Nakuru (N = 1055)	180 (17.1)	875 (82.9)	< 0.001*
Makueni (N = 933)	37 (4.0)	896 (96.0)	< 0.001*
Meru (N = 434)	88 (20.3)	346 (79.7)	< 0.001*
Bomet (N = 384)	24 (6.3)	360 (93.7)	< 0.001*
Kisumu (N = 351)	218 (62.1)	133 (37.9)	< 0.001*
Garissa (N = 328)	25 (7.6)	303 (92.4)	< 0.001*
Taita Taveta (N = 325)	64 (19.7)	261 (80.3)	< 0.001*
Nyeri (N = 321)	67 (20.9)	254 (79.1)	< 0.001*
Machakos (N = 49)	27 (55.1)	22 (44.9)	< 0.001*

* Significant at 0.05.

### Treatment modalities received

Of the 15,275 patients who received treatment at regional centers, 10,550 (69.1%) received chemotherapy, and 16,964 (57.9%) received palliative care. Radiotherapy was the most common reason for referral from the regional cancer centers to other facilities (50.3%).

### Availability of histology and HIV test results

**Fig 4:**
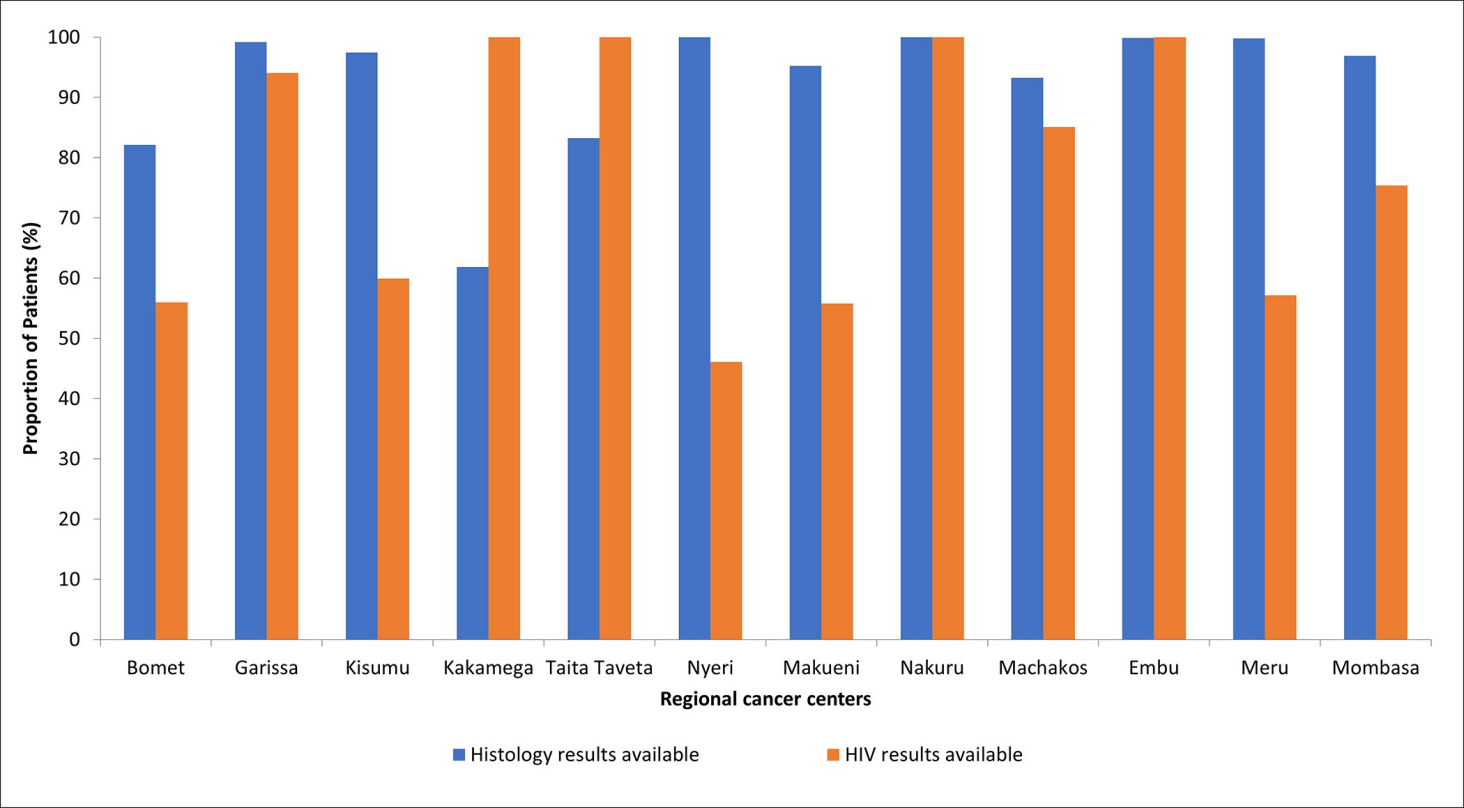
Histology and HIV results availability at the regional cancer centres.

Overall, 92.4% of patients seen at regional cancer centers had histological results, with the lowest rate at Kakamega (62%) and the highest rate (100%) at Embu and Meru. The availability of HIV results was lower than that for histology in most centers, except for Kakamega and Taita Taveta centers; the average availability was 77.5% as shown below in Fig 4.

## Discussion

This study aimed to assess sociodemographic characteristics, staging characteristics, and utilization of cancer services following the establishment of regional cancer centers. More than half of the patients seen at the centers were female, with male patients being significantly older. A decrease in the number of new patients was noted at the main national hospital, coinciding with the establishment of regional centers. Chemotherapy was the most common treatment modality used in regional centers and half of all the patients were referred to other facilities for radiotherapy.

Female patients were almost a decade younger than male patients. This could be due to the higher incidence of breast and cervical cancer in Kenya as well as better health-seeking behaviour among women. This is similar to a study by Rajesh et al., in which 58% of all cases were female, and the incidence peaked in the 60–64 years age group [9]. The GLOBOCAN 2020 findings for Kenya indicate that females had a higher incidence and were disproportionately affected with cancer occurring at a younger age [1]. Another study conducted in Kenya in 2019 found that the majority of patients were female (57%), with the most frequent age being 52 years compared to 62 years in males [19]. A study conducted in Tanzania also reported that a majority of females (62.1%) had a mean age of 48 years [20].

Only 3% of the patients visiting the centers were children aged 0–19 years. Pediatric cancers are rarer than adult cancers, with approximately 3,272 cases reported in 2018 [2] although this could be an underrepresentation as we expect pediatric cases to account for at least 10% of the cancer patient population in this setting. As these centers do not have pediatric cancer specialists, most children with cancer are seen at various service delivery points without aggregation of this data and then referred to the national referral hospitals. An accurate appraisal of childhood cancer incidence and outcomes is non-existent in many LMICs, partly due to weak cancer registration systems necessary to record and report these data [21].

We found that over 38% of all patient visits to public cancer centers occurred at regional cancer centers. This implies an improvement in service access in terms of geographical access, acceptability, and affordability, to some extent. A study in Mozambique found that living in the proximity of a health facility and having greater availability of staff and equipment increases the probability of seeking care [22]. Participants in a study conducted in Kenya prior to decentralization reported that centralization of cancer diagnostic and treatment services in the capital city was a key challenge since long distances affected patient compliance with treatment and follow-up [23]. Another Kenyan study reported limited access to cancer diagnosis and treatment since most cancer care services were concentrated within a five-kilometer radius of each other in the capital city [19]. Therefore, limited capacity for cancer diagnosis and treatment has implications for access, availability, and outcomes.

We noted that only approximately 36% of the patients had stage information, and most presented with advanced disease. This implies poor patient knowledge and awareness about their disease status. Age was a significant predictor of a late-stage presentation. Although aging is well recognized as a risk factor for cancer, targeted cancer control interventions may be required for older persons to enable early diagnosis. There was a significant association between sex and cancer stage, with the majority of those presenting in advanced stages being older male patients. A study in Botswana suggested that frequent interactions with the health system by women could explain this finding [24]. A systematic review conducted in SSA showed that the percentage of late-stage breast cancer at diagnosis in black populations from sub-Saharan Africa around 2010 was higher than that in black and white populations in the USA 40 years ago [25]. Another study conducted in Tanzania reported late diagnosis with a median delay between first symptoms and cancer diagnosis of almost one year [26].

The Kisumu and Machakos cancer centers recorded a relatively higher proportion of patients presenting at earlier stages (62% and 55%, respectively) than the national status, where about 30% of patients present in the early stages of cancer. This can be explained by the existence of robust cancer screening and early diagnostic programs that have been in place in these two counties. Therefore, cancer control strategies in regional centers should target establishing screening and early diagnosis programs to improve the chances of patients presenting in earlier stages of the disease to increase chances of cure and boost survival rates.

Although some regional centers do not have functional pathology laboratory services in place due to a myriad of factors, most patients had histological and HIV results before treatment initiation. Deficiency in pathology and other cancer diagnostic services, both in volume and quality, is one of the main cancer control challenges in SSA [27–29]. Persons living with HIV have a higher risk of developing malignant neoplasms. A study by Calkins et al. found that CD4 count decline associated with cancer treatment was concerning for persons with HIV, and immunosuppression in persons with HIV driven by cancer treatment, rather than the HIV disease process, could result in an increased risk of mortality [30]. Therefore, knowledge of the HIV status of all cancer patients prior to treatment, especially in regions with higher HIV prevalence, is important and may be a good quality of care indicator for enhancing survival rates. Clinicians should ensure that all patients with cancer have both a histological and HIV diagnosis and clinical staging is conducted prior to treatment initiation.

Most of the regional cancer centers provided chemotherapy, surgery, hormonal therapy, and palliative care. Chemotherapy is one of the cancer treatment modalities that has been shown to be feasible even in a resource-limited clinical setting [31]. However, such cancer centers must establish systems to monitor quality of care. Palliative care has also been widely embraced at regional sites in line with the Kenya Palliative Care Policy 2021–2030 [32]. As of 2021, radiotherapy services had not yet been established at any regional center, but at the time of writing, radiotherapy services have since been established at three regional centers in Nakuru, Garissa, and Mombasa.

As expected, most referrals from regional centers were for radiotherapy to the national referral hospitals. Approximately 50% of all patients with cancer require radiation therapy during the course of their illness, accounting for 40% of the curative treatment for cancer [33]. Although three regional cancer centers have established radiotherapy services since then, efforts to expand to more regional centers have been greatly hindered by the limited availability of specialized radiotherapy personnel. Strengthening the existing local training programs while addressing career progression and retention may enhance the availability of these human resources.

### Strengths and limitations

Our study has two strengths. First, it was conducted when Kenya aggressively pursued decentralization of cancer services. Second, we analyzed information from all regional cancer centers; therefore, our findings are representative of the actual situation of the cancer service decentralization program. A major limitation of this study was that the surveillance platforms used as data sources did not comprehensively capture all the important cancer diagnosis and treatment variables, especially for different cancer types. This limited exploration of patterns across different cancer types would have been useful in determining the specific services that need to be reinforced for each cancer type. Another major limitation was the missing staging information for majority of patients visiting the cancer centers.

## Conclusion

Decentralization presents an opportunity for patients to be managed in less-specialized local hospitals. Service uptake was good in the regional cancer centers, with most patients receiving chemotherapy, although the majority were in advanced stages of the disease. Effective cancer control requires a comprehensive strategy encompassing screening, early diagnosis programs so that patients are diagnosed in early stages to improve clinical outcomes. There is a need to strengthen programs for patient registration and surveillance in cancer centers to improve data use for planning and research for policy. Further studies are required to determine which tailored interventions may be useful to improve staging and earlier diagnosis of cancer as persons age, particularly among males in our setting. Nevertheless, improving access to comprehensive cancer treatment remains essential as it produces large health benefits and may have significant returns on investment.

## Supporting information

S1 Data Dataset. (PDF)
