# Supplementary material for: Decentralizing cancer care in sub-Saharan Africa through an integrated regional cancer centre model: The case of Kenya
Source: PLOS Glob Public Health. 2023 Sep 22;3(9):e0002402. doi: 10.1371/journal.pgph.0002402 (PMC10516416; doi:10.1371/journal.pgph.0002402)
Supplement: S1 Data — (PDF) [file pgph.0002402.s001.pdf]

Facility
Nyeri Cancer Centre
Meru Cancer Centre
Taita Taveta Cancer Centre
Kakamega Cancer Centre
Garissa Cancer Centre
Embu Cancer Centre
Makueni Cancer Centre
Kisumu Cancer Centre
Mombasa Cancer Centre
Mombasa Cancer Centre
Garissa Cancer Centre
Nakuru Cancer Centre
Taita Taveta Cancer Centre
Embu Cancer Centre
Embu Cancer Centre
Bomet Cancer Centre
Machakos Cancer Centre
Nyeri Cancer Centre
Kenyatta National Hospital
Kenyatta National Hospital
Nyeri Cancer Centre
Machakos Cancer Centre
Kisumu Cancer Centre
Meru Cancer Centre
Meru Cancer Centre
Kisumu Cancer Centre
Machakos Cancer Centre
Taita Taveta Cancer Centre
Nakuru Cancer Centre
Embu Cancer Centre
Garissa Cancer Centre
Meru Cancer Centre
Bomet Cancer Centre
Kenyatta University Teaching Refferal and Research Hospital
Kenyatta University Teaching Refferal and Research Hospital
Mombasa Cancer Centre
Mombasa Cancer Centre
Makueni Cancer Centre
Embu Cancer Centre
Garissa Cancer Centre
Taita Taveta Cancer Centre
Taita Taveta Cancer Centre
Nyeri Cancer Centre
Kenyatta University Teaching Refferal and Research Hospital
Nyeri Cancer Centre
Kenyatta University Teaching Refferal and Research Hospital
Taita Taveta Cancer Centre
Taita Taveta Cancer Centre
Meru Cancer Centre

Meru Cancer Centre
Makueni Cancer Centre
Bomet Cancer Centre
Mombasa Cancer Centre
Garissa Cancer Centre
Mombasa Cancer Centre
Embu Cancer Centre
Kakamega Cancer Centre
Kakamega Cancer Centre
Kisumu Cancer Centre
Kisumu Cancer Centre
Kenyatta National Hospital
Kenyatta National Hospital
Taita Taveta Cancer Centre
Taita Taveta Cancer Centre
Garissa Cancer Centre
Garissa Cancer Centre
Nyeri Cancer Centre
Nyeri Cancer Centre
Makueni Cancer Centre
Nyeri Cancer Centre
Nyeri Cancer Centre
Garissa Cancer Centre
Taita Taveta Cancer Centre
Nakuru Cancer Centre
Nakuru Cancer Centre
Embu Cancer Centre
Bomet Cancer Centre
Meru Cancer Centre
Meru Cancer Centre
Kisumu Cancer Centre
Kisumu Cancer Centre
Taita Taveta Cancer Centre
Embu Cancer Centre
Kenyatta University Teaching Refferal and Research Hospital
Kenyatta University Teaching Refferal and Research Hospital
Makueni Cancer Centre
Garissa Cancer Centre
Bomet Cancer Centre
Kakamega Cancer Centre
Taita Taveta Cancer Centre
Nyeri Cancer Centre
Kisumu Cancer Centre
Nakuru Cancer Centre
Nakuru Cancer Centre
Makueni Cancer Centre
Bomet Cancer Centre
Kisumu Cancer Centre
Machakos Cancer Centre
Kisumu Cancer Centre

Machakos Cancer Centre
Garissa Cancer Centre
Taita Taveta Cancer Centre
Embu Cancer Centre
Taita Taveta Cancer Centre
Garissa Cancer Centre
Makueni Cancer Centre
Kisumu Cancer Centre
Machakos Cancer Centre
Machakos Cancer Centre
Nakuru Cancer Centre
Nyeri Cancer Centre
Embu Cancer Centre
Nyeri Cancer Centre
Nyeri Cancer Centre
Garissa Cancer Centre
Nakuru Cancer Centre
Bomet Cancer Centre
Kisumu Cancer Centre
Nakuru Cancer Centre
Bomet Cancer Centre
Mombasa Cancer Centre
Mombasa Cancer Centre
Embu Cancer Centre
Nakuru Cancer Centre
Taita Taveta Cancer Centre
Meru Cancer Centre
Kenyatta University Teaching Refferal and Research Hospital
Meru Cancer Centre
Kenyatta University Teaching Refferal and Research Hospital
Kenyatta University Teaching Refferal and Research Hospital
Meru Cancer Centre
Embu Cancer Centre
Taita Taveta Cancer Centre
Meru Cancer Centre
Kenyatta National Hospital
Kenyatta National Hospital
Nakuru Cancer Centre
Garissa Cancer Centre
Bomet Cancer Centre
Meru Cancer Centre
Meru Cancer Centre
Kenyatta University Teaching Refferal and Research Hospital
Kenyatta University Teaching Refferal and Research Hospital
Bomet Cancer Centre
Nakuru Cancer Centre
Embu Cancer Centre
Taita Taveta Cancer Centre
Garissa Cancer Centre
Mombasa Cancer Centre

Mombasa Cancer Centre
Garissa Cancer Centre
Bomet Cancer Centre
Garissa Cancer Centre
Kenyatta University Teaching Refferal and Research Hospital
Kenyatta University Teaching Refferal and Research Hospital
Meru Cancer Centre
Meru Cancer Centre
Taita Taveta Cancer Centre
Nakuru Cancer Centre
Kenyatta University Teaching Refferal and Research Hospital
Kenyatta University Teaching Refferal and Research Hospital
Taita Taveta Cancer Centre
Meru Cancer Centre
Bomet Cancer Centre
Meru Cancer Centre
Nakuru Cancer Centre
Mombasa Cancer Centre
Mombasa Cancer Centre
Garissa Cancer Centre
Taita Taveta Cancer Centre
Meru Cancer Centre
Kenyatta National Hospital
Embu Cancer Centre
Kenyatta National Hospital
Kenyatta University Teaching Refferal and Research Hospital
Kenyatta University Teaching Refferal and Research Hospital
Mombasa Cancer Centre
Mombasa Cancer Centre
Embu Cancer Centre
Garissa Cancer Centre
Garissa Cancer Centre
Nakuru Cancer Centre
Kenyatta University Teaching Refferal and Research Hospital
Kenyatta University Teaching Refferal and Research Hospital
Bomet Cancer Centre
Kenyatta University Teaching Refferal and Research Hospital
Meru Cancer Centre
Meru Cancer Centre
Bomet Cancer Centre
Kenyatta University Teaching Refferal and Research Hospital
Nakuru Cancer Centre
Embu Cancer Centre
Taita Taveta Cancer Centre
Nakuru Cancer Centre
Kenyatta University Teaching Refferal and Research Hospital
Taita Taveta Cancer Centre
Embu Cancer Centre
Bomet Cancer Centre
Nakuru Cancer Centre

Kenyatta University Teaching Refferal and Research Hospital
Meru Cancer Centre
Kenyatta University Teaching Refferal and Research Hospital
Bomet Cancer Centre
Garissa Cancer Centre
Taita Taveta Cancer Centre
Embu Cancer Centre
Kenyatta University Teaching Refferal and Research Hospital
Kenyatta University Teaching Refferal and Research Hospital
Garissa Cancer Centre
Garissa Cancer Centre
Bomet Cancer Centre
Meru Cancer Centre
Embu Cancer Centre
Meru Cancer Centre
Nakuru Cancer Centre
Taita Taveta Cancer Centre
Kenyatta University Teaching Refferal and Research Hospital
Taita Taveta Cancer Centre
Embu Cancer Centre
Bomet Cancer Centre
Nakuru Cancer Centre
Garissa Cancer Centre
Garissa Cancer Centre
Kenyatta University Teaching Refferal and Research Hospital
Meru Cancer Centre
Nyeri Cancer Centre
Kakamega Cancer Centre
Kakamega Cancer Centre
Garissa Cancer Centre
Meru Cancer Centre
Embu Cancer Centre
Makueni Cancer Centre
Kisumu Cancer Centre
Kisumu Cancer Centre
Mombasa Cancer Centre
Mombasa Cancer Centre
Kenyatta University Teaching Refferal and Research Hospital
Embu Cancer Centre
Kakamega Cancer Centre
Garissa Cancer Centre
Kakamega Cancer Centre
Kisumu Cancer Centre
Nyeri Cancer Centre
Kenyatta National Hospital
Kenyatta National Hospital
Meru Cancer Centre
Meru Cancer Centre
Kenyatta University Teaching Refferal and Research Hospital
Kenyatta University Teaching Refferal and Research Hospital

Kakamega Cancer Centre
Kenyatta National Hospital
Nyeri Cancer Centre
Embu Cancer Centre
Kisumu Cancer Centre
Kisumu Cancer Centre
Meru Cancer Centre
Meru Cancer Centre
Kisumu Cancer Centre
Kenyatta University Teaching Refferal and Research Hospital
Kenyatta University Teaching Refferal and Research Hospital
Mombasa Cancer Centre
Mombasa Cancer Centre
Makueni Cancer Centre
Garissa Cancer Centre
Nyeri Cancer Centre
Nyeri Cancer Centre
Meru Cancer Centre
Kenyatta University Teaching Refferal and Research Hospital
Kenyatta University Teaching Refferal and Research Hospital
Meru Cancer Centre
Makueni Cancer Centre
Mombasa Cancer Centre
Mombasa Cancer Centre
Garissa Cancer Centre
Kakamega Cancer Centre
Kisumu Cancer Centre
Kisumu Cancer Centre
Kenyatta National Hospital
Kisumu Cancer Centre
Kenyatta National Hospital
Garissa Cancer Centre
Makueni Cancer Centre
Nyeri Cancer Centre
Kakamega Cancer Centre
Kakamega Cancer Centre
Meru Cancer Centre
Kakamega Cancer Centre
Makueni Cancer Centre
Kakamega Cancer Centre
Meru Cancer Centre
Garissa Cancer Centre
Kisumu Cancer Centre
Nyeri Cancer Centre
Nakuru Cancer Centre
Nakuru Cancer Centre
Kisumu Cancer Centre
Kisumu Cancer Centre
Nyeri Cancer Centre
Garissa Cancer Centre

Kakamega Cancer Centre
Kenyatta University Teaching Refferal and Research Hospital
Kakamega Cancer Centre
Kenyatta University Teaching Refferal and Research Hospital
Makueni Cancer Centre
Meru Cancer Centre
Meru Cancer Centre
Embu Cancer Centre
Garissa Cancer Centre
Kakamega Cancer Centre
Kisumu Cancer Centre
Makueni Cancer Centre
Nyeri Cancer Centre
Nakuru Cancer Centre
Nakuru Cancer Centre
Kisumu Cancer Centre
Kisumu Cancer Centre
Kakamega Cancer Centre
Meru Cancer Centre
Garissa Cancer Centre
Kenyatta University Teaching Refferal and Research Hospital
Kisumu Cancer Centre
Kisumu Cancer Centre
Garissa Cancer Centre
Makueni Cancer Centre
Meru Cancer Centre
Meru Cancer Centre
Nakuru Cancer Centre
Embu Cancer Centre
Nyeri Cancer Centre
Nyeri Cancer Centre
Makueni Cancer Centre
Kenyatta University Teaching Refferal and Research Hospital
Kenyatta University Teaching Refferal and Research Hospital
Kakamega Cancer Centre
Nakuru Cancer Centre
Nakuru Cancer Centre
Garissa Cancer Centre
Kisumu Cancer Centre
Meru Cancer Centre
Nyeri Cancer Centre
Kakamega Cancer Centre
Garissa Cancer Centre
Garissa Cancer Centre
Makueni Cancer Centre
Mombasa Cancer Centre
Mombasa Cancer Centre
Kisumu Cancer Centre
Embu Cancer Centre
Embu Cancer Centre

Kakamega Cancer Centre
Makueni Cancer Centre
Kenyatta National Hospital
Kenyatta National Hospital
Machakos Cancer Centre
Nyeri Cancer Centre
Machakos Cancer Centre
Meru Cancer Centre
Meru Cancer Centre
Kisumu Cancer Centre
Kakamega Cancer Centre
Machakos Cancer Centre
Machakos Cancer Centre
Kisumu Cancer Centre
Garissa Cancer Centre
Embu Cancer Centre
Embu Cancer Centre
Garissa Cancer Centre
Meru Cancer Centre
Kenyatta University Teaching Refferal and Research Hospital
Kenyatta University Teaching Refferal and Research Hospital
Mombasa Cancer Centre
Mombasa Cancer Centre
Embu Cancer Centre
Garissa Cancer Centre
Nyeri Cancer Centre
Machakos Cancer Centre
Kenyatta University Teaching Refferal and Research Hospital
Kenyatta University Teaching Refferal and Research Hospital
Nyeri Cancer Centre
Machakos Cancer Centre
Meru Cancer Centre
Meru Cancer Centre
Garissa Cancer Centre
Mombasa Cancer Centre
Mombasa Cancer Centre
Garissa Cancer Centre
Kakamega Cancer Centre
Kakamega Cancer Centre
Embu Cancer Centre
Kisumu Cancer Centre
Kisumu Cancer Centre
Kenyatta National Hospital
Kisumu Cancer Centre
Machakos Cancer Centre
Machakos Cancer Centre
Kenyatta National Hospital
Garissa Cancer Centre
Makueni Cancer Centre
Nyeri Cancer Centre

Meru Cancer Centre
Kisumu Cancer Centre
Makueni Cancer Centre
Nyeri Cancer Centre
Garissa Cancer Centre
Garissa Cancer Centre
Nakuru Cancer Centre
Nakuru Cancer Centre
Machakos Cancer Centre
Kisumu Cancer Centre
Kisumu Cancer Centre
Nyeri Cancer Centre
Meru Cancer Centre
Nakuru Cancer Centre
Embu Cancer Centre
Kenyatta University Teaching Refferal and Research Hospital
Makueni Cancer Centre
Kenyatta University Teaching Refferal and Research Hospital
Makueni Cancer Centre
Garissa Cancer Centre
Garissa Cancer Centre
Nyeri Cancer Centre
Meru Cancer Centre
Makueni Cancer Centre
Embu Cancer Centre
Embu Cancer Centre
Nakuru Cancer Centre
Nakuru Cancer Centre
Kisumu Cancer Centre
Kisumu Cancer Centre
Machakos Cancer Centre
Machakos Cancer Centre
Kakamega Cancer Centre
Garissa Cancer Centre
Garissa Cancer Centre
Kisumu Cancer Centre
Garissa Cancer Centre
Garissa Cancer Centre
Kakamega Cancer Centre
Makueni Cancer Centre
Machakos Cancer Centre
Machakos Cancer Centre
Embu Cancer Centre
Nyeri Cancer Centre
Embu Cancer Centre
Machakos Cancer Centre
Machakos Cancer Centre
Makueni Cancer Centre
Nyeri Cancer Centre
Kakamega Cancer Centre

Garissa Cancer Centre
Garissa Cancer Centre
Nakuru Cancer Centre
Nakuru Cancer Centre
Embu Cancer Centre
Embu Cancer Centre
Mombasa Cancer Centre
Taita Taveta Cancer Centre
Taita Taveta Cancer Centre
Garissa Cancer Centre
Meru Cancer Centre
Mombasa Cancer Centre
Garissa Cancer Centre
Garissa Cancer Centre
Embu Cancer Centre
Embu Cancer Centre
Taita Taveta Cancer Centre
Taita Taveta Cancer Centre
Kenyatta National Hospital
Kenyatta National Hospital
Nyeri Cancer Centre
Nyeri Cancer Centre
Meru Cancer Centre
Kisumu Cancer Centre
Meru Cancer Centre
Kisumu Cancer Centre
Bomet Cancer Centre
Taita Taveta Cancer Centre
Garissa Cancer Centre
Garissa Cancer Centre
Mombasa Cancer Centre
Mombasa Cancer Centre
Bomet Cancer Centre
Garissa Cancer Centre
Kenyatta University Teaching Refferal and Research Hospital
Kenyatta University Teaching Refferal and Research Hospital
Meru Cancer Centre
Taita Taveta Cancer Centre
Taita Taveta Cancer Centre
Machakos Cancer Centre
Garissa Cancer Centre
Machakos Cancer Centre
Meru Cancer Centre
Taita Taveta Cancer Centre
Bomet Cancer Centre
Kenyatta University Teaching Refferal and Research Hospital
Kenyatta University Teaching Refferal and Research Hospital
Bomet Cancer Centre
Meru Cancer Centre
Mombasa Cancer Centre

Mombasa Cancer Centre
Kisumu Cancer Centre
Garissa Cancer Centre
Garissa Cancer Centre
Taita Taveta Cancer Centre
Bomet Cancer Centre
Kenyatta National Hospital
Kenyatta National Hospital
Nyeri Cancer Centre
Nyeri Cancer Centre
Makueni Cancer Centre
Makueni Cancer Centre
Nyeri Cancer Centre
Nyeri Cancer Centre
Garissa Cancer Centre
Garissa Cancer Centre
Nakuru Cancer Centre
Nakuru Cancer Centre
Taita Taveta Cancer Centre
Taita Taveta Cancer Centre
Bomet Cancer Centre
Bomet Cancer Centre
Garissa Cancer Centre
Bomet Cancer Centre
Kenyatta University Teaching Refferal and Research Hospital
Bomet Cancer Centre
Meru Cancer Centre
Kenyatta University Teaching Refferal and Research Hospital
Taita Taveta Cancer Centre
Garissa Cancer Centre
Kisumu Cancer Centre
Kisumu Cancer Centre
Garissa Cancer Centre
Taita Taveta Cancer Centre
Taita Taveta Cancer Centre
Bomet Cancer Centre
Kisumu Cancer Centre
Garissa Cancer Centre
Nakuru Cancer Centre
Nakuru Cancer Centre
Garissa Cancer Centre
Garissa Cancer Centre
Kisumu Cancer Centre
Machakos Cancer Centre
Machakos Cancer Centre
Kisumu Cancer Centre
Garissa Cancer Centre
Taita Taveta Cancer Centre
Machakos Cancer Centre
Makueni Cancer Centre

Makueni Cancer Centre
Machakos Cancer Centre
Taita Taveta Cancer Centre
Nakuru Cancer Centre
Nakuru Cancer Centre
Garissa Cancer Centre
Bomet Cancer Centre
Bomet Cancer Centre
Makueni Cancer Centre
Mombasa Cancer Centre
Mombasa Cancer Centre
Embu Cancer Centre
Makueni Cancer Centre
Embu Cancer Centre
Bomet Cancer Centre
Kenyatta National Hospital
Kenyatta National Hospital
Nyeri Cancer Centre
Nyeri Cancer Centre
Meru Cancer Centre
Meru Cancer Centre
Kisumu Cancer Centre
Kisumu Cancer Centre
Makueni Cancer Centre
Taita Taveta Cancer Centre
Garissa Cancer Centre
Garissa Cancer Centre
Bomet Cancer Centre
Kenyatta University Teaching Refferal and Research Hospital
Kenyatta University Teaching Refferal and Research Hospital
Mombasa Cancer Centre
Mombasa Cancer Centre
Garissa Cancer Centre
Taita Taveta Cancer Centre
Bomet Cancer Centre
Bomet Cancer Centre
Kenyatta University Teaching Refferal and Research Hospital
Kenyatta University Teaching Refferal and Research Hospital
Machakos Cancer Centre
Meru Cancer Centre
Meru Cancer Centre
Mombasa Cancer Centre
Mombasa Cancer Centre
Kenyatta National Hospital
Bomet Cancer Centre
Kenyatta National Hospital
Taita Taveta Cancer Centre
Taita Taveta Cancer Centre
Nyeri Cancer Centre
Nyeri Cancer Centre

Nyeri Cancer Centre
Nyeri Cancer Centre
Garissa Cancer Centre
Nakuru Cancer Centre
Nakuru Cancer Centre
Taita Taveta Cancer Centre
Bomet Cancer Centre
Kisumu Cancer Centre
Kenyatta University Teaching Refferal and Research Hospital
Kisumu Cancer Centre
Garissa Cancer Centre
Meru Cancer Centre
Bomet Cancer Centre
Taita Taveta Cancer Centre
Makueni Cancer Centre
Kenyatta University Teaching Refferal and Research Hospital
Machakos Cancer Centre
Makueni Cancer Centre
Nakuru Cancer Centre
Nakuru Cancer Centre
Machakos Cancer Centre
Machakos Cancer Centre
Garissa Cancer Centre
Taita Taveta Cancer Centre
Kisumu Cancer Centre
Kisumu Cancer Centre
Taita Taveta Cancer Centre
Makueni Cancer Centre
Makueni Cancer Centre
Machakos Cancer Centre
Machakos Cancer Centre
Kisumu Cancer Centre
Makueni Cancer Centre
Makueni Cancer Centre
Garissa Cancer Centre
Taita Taveta Cancer Centre
Nakuru Cancer Centre
Nakuru Cancer Centre
Kisumu Cancer Centre
Taita Taveta Cancer Centre
Meru Cancer Centre
Mombasa Cancer Centre
Mombasa Cancer Centre
Embu Cancer Centre
Embu Cancer Centre
Garissa Cancer Centre
Kenyatta National Hospital
Kenyatta National Hospital
Bomet Cancer Centre
Nyeri Cancer Centre

Nyeri Cancer Centre
Meru Cancer Centre
Kisumu Cancer Centre
Kisumu Cancer Centre
Meru Cancer Centre
Garissa Cancer Centre
Mombasa Cancer Centre
Bomet Cancer Centre
Kenyatta University Teaching Refferal and Research Hospital
Kenyatta University Teaching Refferal and Research Hospital
Mombasa Cancer Centre
Meru Cancer Centre
Machakos Cancer Centre
Machakos Cancer Centre
Bomet Cancer Centre
Meru Cancer Centre
Bomet Cancer Centre
Taita Taveta Cancer Centre
Kenyatta University Teaching Refferal and Research Hospital
Kenyatta University Teaching Refferal and Research Hospital
Meru Cancer Centre
Mombasa Cancer Centre
Mombasa Cancer Centre
Garissa Cancer Centre
Kenyatta National Hospital
Bomet Cancer Centre
Kenyatta National Hospital
Taita Taveta Cancer Centre
Nyeri Cancer Centre
Nyeri Cancer Centre
Nyeri Cancer Centre
Nyeri Cancer Centre
Garissa Cancer Centre
Nakuru Cancer Centre
Nakuru Cancer Centre
Taita Taveta Cancer Centre
Bomet Cancer Centre
Kisumu Cancer Centre
Kisumu Cancer Centre
Kenyatta University Teaching Refferal and Research Hospital
Kenyatta University Teaching Refferal and Research Hospital
Taita Taveta Cancer Centre
Garissa Cancer Centre
Taita Taveta Cancer Centre
Nakuru Cancer Centre
Nakuru Cancer Centre
Kisumu Cancer Centre
Kisumu Cancer Centre
Machakos Cancer Centre
Machakos Cancer Centre

Garissa Cancer Centre
Bomet Cancer Centre
Taita Taveta Cancer Centre
Embu Cancer Centre
Machakos Cancer Centre
Machakos Cancer Centre
Nakuru Cancer Centre
Garissa Cancer Centre
Nakuru Cancer Centre
Bomet Cancer Centre
Makueni Cancer Centre
Mombasa Cancer Centre
Mombasa Cancer Centre
Kisumu Cancer Centre
Embu Cancer Centre
Makueni Cancer Centre
Embu Cancer Centre
Kenyatta National Hospital
Kenyatta National Hospital
Nyeri Cancer Centre
Nyeri Cancer Centre
Kisumu Cancer Centre
Meru Cancer Centre
Kisumu Cancer Centre
Meru Cancer Centre
Bomet Cancer Centre
Bomet Cancer Centre
Kenyatta University Teaching Refferal and Research Hospital
Kenyatta University Teaching Refferal and Research Hospital
Mombasa Cancer Centre
Mombasa Cancer Centre
Kakamega Cancer Centre
Makueni Cancer Centre
Kenyatta University Teaching Refferal and Research Hospital
Kenyatta University Teaching Refferal and Research Hospital
Bomet Cancer Centre
Meru Cancer Centre
Bomet Cancer Centre
Nyeri Cancer Centre
Meru Cancer Centre
Mombasa Cancer Centre
Mombasa Cancer Centre
Bomet Cancer Centre
Kenyatta National Hospital
Kenyatta National Hospital
Garissa Cancer Centre
Nyeri Cancer Centre
Nyeri Cancer Centre
Kakamega Cancer Centre
Nyeri Cancer Centre

Nyeri Cancer Centre
Garissa Cancer Centre
Nakuru Cancer Centre
Nakuru Cancer Centre
Taita Taveta Cancer Centre
Bomet Cancer Centre
Bomet Cancer Centre
Kisumu Cancer Centre
Kisumu Cancer Centre
Nyeri Cancer Centre
Kenyatta University Teaching Refferal and Research Hospital
Kenyatta University Teaching Refferal and Research Hospital
Garissa Cancer Centre
Machakos Cancer Centre
Bomet Cancer Centre
Nakuru Cancer Centre
Nakuru Cancer Centre
Bomet Cancer Centre
Kisumu Cancer Centre
Machakos Cancer Centre
Machakos Cancer Centre
Bomet Cancer Centre
Kisumu Cancer Centre
Embu Cancer Centre
Makueni Cancer Centre
Machakos Cancer Centre
Machakos Cancer Centre
Bomet Cancer Centre
Nakuru Cancer Centre
Nakuru Cancer Centre
Meru Cancer Centre
Meru Cancer Centre
Meru Cancer Centre
Meru Cancer Centre
Meru Cancer Centre
Meru Cancer Centre
Meru Cancer Centre
Meru Cancer Centre
Meru Cancer Centre
Meru Cancer Centre
Meru Cancer Centre
Bomet Cancer Centre
Mombasa Cancer Centre
Bomet Cancer Centre
Mombasa Cancer Centre
Nakuru Cancer Centre
Meru Cancer Centre
Meru Cancer Centre
Makueni Cancer Centre
Machakos Cancer Centre

Makueni Cancer Centre
Machakos Cancer Centre
Bomet Cancer Centre
Makueni Cancer Centre
Machakos Cancer Centre
Machakos Cancer Centre
Bomet Cancer Centre
Nyeri Cancer Centre
Meru Cancer Centre
Meru Cancer Centre
Nyeri Cancer Centre
Bomet Cancer Centre
Mombasa Cancer Centre
Taita Taveta Cancer Centre
Machakos Cancer Centre
Machakos Cancer Centre
Meru Cancer Centre
Meru Cancer Centre
Mombasa Cancer Centre
Bomet Cancer Centre
Nakuru Cancer Centre
Bomet Cancer Centre
Bomet Cancer Centre
Meru Cancer Centre
Meru Cancer Centre
Machakos Cancer Centre
Taita Taveta Cancer Centre
Mombasa Cancer Centre
Mombasa Cancer Centre
Makueni Cancer Centre
Bomet Cancer Centre
Machakos Cancer Centre
Machakos Cancer Centre
Machakos Cancer Centre
Machakos Cancer Centre
Meru Cancer Centre
Nakuru Cancer Centre
Nakuru Cancer Centre
Bomet Cancer Centre
Meru Cancer Centre
Bomet Cancer Centre
Meru Cancer Centre
Nyeri Cancer Centre
Nakuru Cancer Centre
Makueni Cancer Centre
Machakos Cancer Centre
Meru Cancer Centre
Taita Taveta Cancer Centre
Machakos Cancer Centre
Bomet Cancer Centre

Nyeri Cancer Centre
Nyeri Cancer Centre
Nakuru Cancer Centre
Nakuru Cancer Centre
Machakos Cancer Centre
Nyeri Cancer Centre
Machakos Cancer Centre
Bomet Cancer Centre
Bomet Cancer Centre
Makueni Cancer Centre
Nyeri Cancer Centre
Bomet Cancer Centre
Bomet Cancer Centre
Makueni Cancer Centre
Taita Taveta Cancer Centre
Nakuru Cancer Centre
Makueni Cancer Centre
Nakuru Cancer Centre
Bomet Cancer Centre
Meru Cancer Centre
Garissa Cancer Centre
Mombasa Cancer Centre
Mombasa Cancer Centre
Nakuru Cancer Centre
Meru Cancer Centre
Meru Cancer Centre
Makueni Cancer Centre
Machakos Cancer Centre
Makueni Cancer Centre
Meru Cancer Centre
Mombasa Cancer Centre
Mombasa Cancer Centre
Garissa Cancer Centre
Machakos Cancer Centre
Nakuru Cancer Centre
Garissa Cancer Centre
Makueni Cancer Centre
Machakos Cancer Centre
Nakuru Cancer Centre
Mombasa Cancer Centre
Mombasa Cancer Centre
Garissa Cancer Centre
Meru Cancer Centre
Meru Cancer Centre
Garissa Cancer Centre
Garissa Cancer Centre
Mombasa Cancer Centre
Mombasa Cancer Centre
Makueni Cancer Centre
Machakos Cancer Centre

Meru Cancer Centre
Meru Cancer Centre
Meru Cancer Centre
Nakuru Cancer Centre
Machakos Cancer Centre
Makueni Cancer Centre
Meru Cancer Centre
Mombasa Cancer Centre
Mombasa Cancer Centre
Garissa Cancer Centre
Nakuru Cancer Centre
Makueni Cancer Centre
Garissa Cancer Centre
Meru Cancer Centre
Meru Cancer Centre
Garissa Cancer Centre
Machakos Cancer Centre
Mombasa Cancer Centre
Mombasa Cancer Centre
Makueni Cancer Centre
Meru Cancer Centre
Meru Cancer Centre
Garissa Cancer Centre
Garissa Cancer Centre
Nakuru Cancer Centre
Machakos Cancer Centre
Meru Cancer Centre
Meru Cancer Centre
Garissa Cancer Centre
Garissa Cancer Centre
Nakuru Cancer Centre
Mombasa Cancer Centre
Mombasa Cancer Centre
Makueni Cancer Centre
Machakos Cancer Centre
Nakuru Cancer Centre
Mombasa Cancer Centre
Garissa Cancer Centre
Garissa Cancer Centre
Makueni Cancer Centre
Nakuru Cancer Centre
Mombasa Cancer Centre
Machakos Cancer Centre
Mombasa Cancer Centre
Meru Cancer Centre
Garissa Cancer Centre
Garissa Cancer Centre
Mombasa Cancer Centre
Mombasa Cancer Centre
Garissa Cancer Centre

Garissa Cancer Centre
Meru Cancer Centre
Machakos Cancer Centre
Makueni Cancer Centre
Nakuru Cancer Centre
Makueni Cancer Centre
Machakos Cancer Centre
Meru Cancer Centre
Meru Cancer Centre
Nakuru Cancer Centre
Garissa Cancer Centre
Garissa Cancer Centre
Mombasa Cancer Centre
Mombasa Cancer Centre
Bomet Cancer Centre
Garissa Cancer Centre
Makueni Cancer Centre
Meru Cancer Centre
Meru Cancer Centre
Kisumu Cancer Centre
Machakos Cancer Centre
Makueni Cancer Centre
Taita Taveta Cancer Centre
Taita Taveta Cancer Centre
Meru Cancer Centre
Taita Taveta Cancer Centre
Taita Taveta Cancer Centre
Kisumu Cancer Centre
Garissa Cancer Centre
Bomet Cancer Centre
Kisumu Cancer Centre
Bomet Cancer Centre
Machakos Cancer Centre
Machakos Cancer Centre
Kakamega Cancer Centre
Taita Taveta Cancer Centre
Taita Taveta Cancer Centre
Nyeri Cancer Centre
Garissa Cancer Centre
Meru Cancer Centre
Meru Cancer Centre
Bomet Cancer Centre
Mombasa Cancer Centre
Mombasa Cancer Centre
Garissa Cancer Centre
Kisumu Cancer Centre
Taita Taveta Cancer Centre
Taita Taveta Cancer Centre
Machakos Cancer Centre
Machakos Cancer Centre

Meru Cancer Centre
Meru Cancer Centre
Makueni Cancer Centre
Makueni Cancer Centre
Meru Cancer Centre
Bomet Cancer Centre
Meru Cancer Centre
Machakos Cancer Centre
Machakos Cancer Centre
Taita Taveta Cancer Centre
Taita Taveta Cancer Centre
Mombasa Cancer Centre
Garissa Cancer Centre
Garissa Cancer Centre
Kisumu Cancer Centre
Mombasa Cancer Centre
Kisumu Cancer Centre
Taita Taveta Cancer Centre
Taita Taveta Cancer Centre
Bomet Cancer Centre
Garissa Cancer Centre
Machakos Cancer Centre
Kisumu Cancer Centre
Meru Cancer Centre
Garissa Cancer Centre
Nakuru Cancer Centre
Nakuru Cancer Centre
Taita Taveta Cancer Centre
Taita Taveta Cancer Centre
Meru Cancer Centre
Meru Cancer Centre
Garissa Cancer Centre
Nyeri Cancer Centre
Makueni Cancer Centre
Machakos Cancer Centre
Meru Cancer Centre
Taita Taveta Cancer Centre
Taita Taveta Cancer Centre
Nyeri Cancer Centre
Machakos Cancer Centre
Bomet Cancer Centre
Kisumu Cancer Centre
Garissa Cancer Centre
Garissa Cancer Centre
Nyeri Cancer Centre
Taita Taveta Cancer Centre
Taita Taveta Cancer Centre
Machakos Cancer Centre
Nakuru Cancer Centre
Nyeri Cancer Centre

Machakos Cancer Centre
Nakuru Cancer Centre
Bomet Cancer Centre
Garissa Cancer Centre
Kisumu Cancer Centre
Garissa Cancer Centre
Bomet Cancer Centre
Kisumu Cancer Centre
Makueni Cancer Centre
Taita Taveta Cancer Centre
Taita Taveta Cancer Centre
Kisumu Cancer Centre
Nakuru Cancer Centre
Nakuru Cancer Centre
Machakos Cancer Centre
Machakos Cancer Centre
Kakamega Cancer Centre
Meru Cancer Centre
Taita Taveta Cancer Centre
Taita Taveta Cancer Centre
Bomet Cancer Centre
Garissa Cancer Centre
Bomet Cancer Centre
Mombasa Cancer Centre
Mombasa Cancer Centre
Kakamega Cancer Centre
Makueni Cancer Centre
Kisumu Cancer Centre
Meru Cancer Centre
Meru Cancer Centre
Makueni Cancer Centre
Machakos Cancer Centre
Machakos Cancer Centre
Kisumu Cancer Centre
Makueni Cancer Centre
Makueni Cancer Centre
Bomet Cancer Centre
Makueni Cancer Centre
Kisumu Cancer Centre
Bomet Cancer Centre
Machakos Cancer Centre
Machakos Cancer Centre
Nyeri Cancer Centre
Taita Taveta Cancer Centre
Meru Cancer Centre
Meru Cancer Centre
Mombasa Cancer Centre
Bomet Cancer Centre
Mombasa Cancer Centre
Nyeri Cancer Centre

Meru Cancer Centre
Taita Taveta Cancer Centre
Kakamega Cancer Centre
Machakos Cancer Centre
Kisumu Cancer Centre
Machakos Cancer Centre
Meru Cancer Centre
Makueni Cancer Centre
Bomet Cancer Centre
Machakos Cancer Centre
Machakos Cancer Centre
Makueni Cancer Centre
Meru Cancer Centre
Taita Taveta Cancer Centre
Meru Cancer Centre
Kisumu Cancer Centre
Mombasa Cancer Centre
Mombasa Cancer Centre
Makueni Cancer Centre
Makueni Cancer Centre
Kisumu Cancer Centre
Bomet Cancer Centre
Machakos Cancer Centre
Machakos Cancer Centre
Nyeri Cancer Centre
Makueni Cancer Centre
Kisumu Cancer Centre
Meru Cancer Centre
Nakuru Cancer Centre
Nakuru Cancer Centre
Taita Taveta Cancer Centre
Bomet Cancer Centre
Meru Cancer Centre
Meru Cancer Centre
Bomet Cancer Centre
Nyeri Cancer Centre
Taita Taveta Cancer Centre
Makueni Cancer Centre
Machakos Cancer Centre
Machakos Cancer Centre
Meru Cancer Centre
Kakamega Cancer Centre
Taita Taveta Cancer Centre
Nyeri Cancer Centre
Kisumu Cancer Centre
Makueni Cancer Centre
Machakos Cancer Centre
Nakuru Cancer Centre
Taita Taveta Cancer Centre
Machakos Cancer Centre

Nyeri Cancer Centre
Nyeri Cancer Centre
Nakuru Cancer Centre
Bomet Cancer Centre
Kisumu Cancer Centre
Makueni Cancer Centre
Makueni Cancer Centre
Machakos Cancer Centre
Nyeri Cancer Centre
Machakos Cancer Centre
Bomet Cancer Centre
Kakamega Cancer Centre
Kisumu Cancer Centre
Makueni Cancer Centre
Makueni Cancer Centre
Taita Taveta Cancer Centre
Kisumu Cancer Centre
Nakuru Cancer Centre
Nakuru Cancer Centre
Makueni Cancer Centre
Makueni Cancer Centre
Machakos Cancer Centre
Machakos Cancer Centre
Bomet Cancer Centre
Taita Taveta Cancer Centre
Nyeri Cancer Centre
Mombasa Cancer Centre
Mombasa Cancer Centre
Meru Cancer Centre
Meru Cancer Centre
Machakos Cancer Centre
Kisumu Cancer Centre
Makueni Cancer Centre
Kisumu Cancer Centre
Kakamega Cancer Centre
Meru Cancer Centre
Meru Cancer Centre
Nyeri Cancer Centre
Mombasa Cancer Centre
Mombasa Cancer Centre
Machakos Cancer Centre
Nakuru Cancer Centre
Kisumu Cancer Centre
Kakamega Cancer Centre
Kakamega Cancer Centre
Machakos Cancer Centre
Nyeri Cancer Centre
Nyeri Cancer Centre
Meru Cancer Centre
Meru Cancer Centre

Mombasa Cancer Centre
Mombasa Cancer Centre
Makueni Cancer Centre
Meru Cancer Centre
Machakos Cancer Centre
Kakamega Cancer Centre
Kakamega Cancer Centre
Meru Cancer Centre
Kisumu Cancer Centre
Nyeri Cancer Centre
Meru Cancer Centre
Machakos Cancer Centre
Meru Cancer Centre
Makueni Cancer Centre
Mombasa Cancer Centre
Kisumu Cancer Centre
Mombasa Cancer Centre
Kakamega Cancer Centre
Kakamega Cancer Centre
Makueni Cancer Centre
Kisumu Cancer Centre
Meru Cancer Centre
Machakos Cancer Centre
Kakamega Cancer Centre
Nyeri Cancer Centre
Mombasa Cancer Centre
Makueni Cancer Centre
Kisumu Cancer Centre
Nyeri Cancer Centre
Nakuru Cancer Centre
Kakamega Cancer Centre
Machakos Cancer Centre
Kakamega Cancer Centre
Meru Cancer Centre
Meru Cancer Centre
Nakuru Cancer Centre
Mombasa Cancer Centre
Nyeri Cancer Centre
Kakamega Cancer Centre
Machakos Cancer Centre
Makueni Cancer Centre
Meru Cancer Centre
Meru Cancer Centre
Nakuru Cancer Centre
Nyeri Cancer Centre
Kakamega Cancer Centre
Nyeri Cancer Centre
Kisumu Cancer Centre
Mombasa Cancer Centre
Makueni Cancer Centre

Nyeri Cancer Centre
Nyeri Cancer Centre
Nakuru Cancer Centre
Kakamega Cancer Centre
Machakos Cancer Centre
Kisumu Cancer Centre
Meru Cancer Centre
Makueni Cancer Centre
Nyeri Cancer Centre
Nyeri Cancer Centre
Kakamega Cancer Centre
Kakamega Cancer Centre
Machakos Cancer Centre
Kisumu Cancer Centre
Makueni Cancer Centre
Makueni Cancer Centre
Kisumu Cancer Centre
Machakos Cancer Centre
Kakamega Cancer Centre
Nyeri Cancer Centre
Kakamega Cancer Centre
Meru Cancer Centre
Nakuru Cancer Centre
Mombasa Cancer Centre
Mombasa Cancer Centre
Nyeri Cancer Centre
Garissa Cancer Centre
Kisumu Cancer Centre
Makueni Cancer Centre
Meru Cancer Centre
Meru Cancer Centre
Machakos Cancer Centre
Kakamega Cancer Centre
Machakos Cancer Centre
Taita Taveta Cancer Centre
Taita Taveta Cancer Centre
Kisumu Cancer Centre
Meru Cancer Centre
Meru Cancer Centre
Kisumu Cancer Centre
Makueni Cancer Centre
Taita Taveta Cancer Centre
Taita Taveta Cancer Centre
Kisumu Cancer Centre
Nyeri Cancer Centre
Garissa Cancer Centre
Nakuru Cancer Centre
Kisumu Cancer Centre
Kisumu Cancer Centre
Machakos Cancer Centre

Machakos Cancer Centre
Kakamega Cancer Centre
Kakamega Cancer Centre
Garissa Cancer Centre
Nyeri Cancer Centre
Taita Taveta Cancer Centre
Meru Cancer Centre
Meru Cancer Centre
Garissa Cancer Centre
Mombasa Cancer Centre
Mombasa Cancer Centre
Nyeri Cancer Centre
Meru Cancer Centre
Meru Cancer Centre
Taita Taveta Cancer Centre
Taita Taveta Cancer Centre
Machakos Cancer Centre
Machakos Cancer Centre
Kakamega Cancer Centre
Makueni Cancer Centre
Nakuru Cancer Centre
Kisumu Cancer Centre
Kisumu Cancer Centre
Nyeri Cancer Centre
Nakuru Cancer Centre
Makueni Cancer Centre
Meru Cancer Centre
Machakos Cancer Centre
Machakos Cancer Centre
Taita Taveta Cancer Centre
Taita Taveta Cancer Centre
Meru Cancer Centre
Mombasa Cancer Centre
Kisumu Cancer Centre
Kakamega Cancer Centre
Mombasa Cancer Centre
Kisumu Cancer Centre
Garissa Cancer Centre
Taita Taveta Cancer Centre
Taita Taveta Cancer Centre
Nakuru Cancer Centre
Makueni Cancer Centre
Kisumu Cancer Centre
Kisumu Cancer Centre
Garissa Cancer Centre
Meru Cancer Centre
Kakamega Cancer Centre
Machakos Cancer Centre
Machakos Cancer Centre
Nyeri Cancer Centre

Kisumu Cancer Centre
Kisumu Cancer Centre
Nyeri Cancer Centre
Meru Cancer Centre
Garissa Cancer Centre
Nakuru Cancer Centre
Nakuru Cancer Centre
Taita Taveta Cancer Centre
Taita Taveta Cancer Centre
Kakamega Cancer Centre
Machakos Cancer Centre
Machakos Cancer Centre
Meru Cancer Centre
Meru Cancer Centre
Nakuru Cancer Centre
Nakuru Cancer Centre
Nyeri Cancer Centre
Taita Taveta Cancer Centre
Taita Taveta Cancer Centre
Machakos Cancer Centre
Kakamega Cancer Centre
Makueni Cancer Centre
Machakos Cancer Centre
Meru Cancer Centre
Taita Taveta Cancer Centre
Kakamega Cancer Centre
Kisumu Cancer Centre
Makueni Cancer Centre
Kisumu Cancer Centre
Mombasa Cancer Centre
Nyeri Cancer Centre
Machakos Cancer Centre
Garissa Cancer Centre
Nakuru Cancer Centre
Nyeri Cancer Centre
Machakos Cancer Centre
Nakuru Cancer Centre
Nakuru Cancer Centre
Nyeri Cancer Centre
Kakamega Cancer Centre
Machakos Cancer Centre
Kisumu Cancer Centre
Kisumu Cancer Centre
Makueni Cancer Centre
Garissa Cancer Centre
Taita Taveta Cancer Centre
Taita Taveta Cancer Centre
Nyeri Cancer Centre
Meru Cancer Centre
Machakos Cancer Centre

Kakamega Cancer Centre
Kakamega Cancer Centre
Makueni Cancer Centre
Kisumu Cancer Centre
Kisumu Cancer Centre
Taita Taveta Cancer Centre
Nakuru Cancer Centre
Taita Taveta Cancer Centre
Nakuru Cancer Centre
Nakuru Cancer Centre
Makueni Cancer Centre
Kisumu Cancer Centre
Kisumu Cancer Centre
Machakos Cancer Centre
Kakamega Cancer Centre
Machakos Cancer Centre
Meru Cancer Centre
Taita Taveta Cancer Centre
Taita Taveta Cancer Centre
Garissa Cancer Centre
Mombasa Cancer Centre
Mombasa Cancer Centre
Mombasa Cancer Centre
Mombasa Cancer Centre
Mombasa Cancer Centre
Mombasa Cancer Centre
Mombasa Cancer Centre
Mombasa Cancer Centre
Mombasa Cancer Centre
Mombasa Cancer Centre
Mombasa Cancer Centre
Mombasa Cancer Centre
Mombasa Cancer Centre
Mombasa Cancer Centre
Mombasa Cancer Centre
Mombasa Cancer Centre
Machakos Cancer Centre
Machakos Cancer Centre
Machakos Cancer Centre
Machakos Cancer Centre
Machakos Cancer Centre
Machakos Cancer Centre
Garissa Cancer Centre
Machakos Cancer Centre
Machakos Cancer Centre
Machakos Cancer Centre
Mombasa Cancer Centre
Garissa Cancer Centre
Garissa Cancer Centre
Machakos Cancer Centre

Machakos Cancer Centre
Machakos Cancer Centre
Mombasa Cancer Centre
Mombasa Cancer Centre
Mombasa Cancer Centre
Mombasa Cancer Centre
Mombasa Cancer Centre
Mombasa Cancer Centre
Mombasa Cancer Centre
Mombasa Cancer Centre
Mombasa Cancer Centre
Mombasa Cancer Centre
Mombasa Cancer Centre
Mombasa Cancer Centre
Mombasa Cancer Centre
Mombasa Cancer Centre
Mombasa Cancer Centre
Mombasa Cancer Centre
Machakos Cancer Centre
Machakos Cancer Centre
Machakos Cancer Centre
Mombasa Cancer Centre
Mombasa Cancer Centre
Mombasa Cancer Centre
Mombasa Cancer Centre
Mombasa Cancer Centre
Mombasa Cancer Centre
Mombasa Cancer Centre
Mombasa Cancer Centre
Mombasa Cancer Centre
Mombasa Cancer Centre
Mombasa Cancer Centre
Garissa Cancer Centre
Mombasa Cancer Centre
Mombasa Cancer Centre
Mombasa Cancer Centre
Mombasa Cancer Centre
Mombasa Cancer Centre
Mombasa Cancer Centre
Garissa Cancer Centre
Machakos Cancer Centre
Machakos Cancer Centre
Machakos Cancer Centre
Machakos Cancer Centre
Machakos Cancer Centre
Machakos Cancer Centre
Garissa Cancer Centre
Mombasa Cancer Centre
Mombasa Cancer Centre
Garissa Cancer Centre

Mombasa Cancer Centre
Machakos Cancer Centre
Machakos Cancer Centre
Machakos Cancer Centre
Garissa Cancer Centre
Machakos Cancer Centre
Machakos Cancer Centre
Machakos Cancer Centre
Mombasa Cancer Centre
Machakos Cancer Centre
Machakos Cancer Centre
Mombasa Cancer Centre
Machakos Cancer Centre
Mombasa Cancer Centre
Mombasa Cancer Centre
Garissa Cancer Centre
Garissa Cancer Centre
Machakos Cancer Centre
Machakos Cancer Centre
Machakos Cancer Centre
Garissa Cancer Centre
Kisumu Cancer Centre
Kisumu Cancer Centre
Kisumu Cancer Centre
Kisumu Cancer Centre
Kisumu Cancer Centre
Kisumu Cancer Centre
Kisumu Cancer Centre
Kisumu Cancer Centre
Kisumu Cancer Centre
Kisumu Cancer Centre
Kisumu Cancer Centre
Kisumu Cancer Centre
Kisumu Cancer Centre
Kisumu Cancer Centre
Kisumu Cancer Centre
Kisumu Cancer Centre
Kisumu Cancer Centre
Kisumu Cancer Centre
Kisumu Cancer Centre
Kisumu Cancer Centre
Kisumu Cancer Centre
Kisumu Cancer Centre
Kisumu Cancer Centre
Kisumu Cancer Centre
Kisumu Cancer Centre
Kisumu Cancer Centre
Kisumu Cancer Centre
Kisumu Cancer Centre
Kisumu Cancer Centre

Kisumu Cancer Centre
Kisumu Cancer Centre
Kisumu Cancer Centre
Kisumu Cancer Centre
Kisumu Cancer Centre
Kisumu Cancer Centre
Kisumu Cancer Centre
Kisumu Cancer Centre
Kisumu Cancer Centre
Kisumu Cancer Centre
Kisumu Cancer Centre
Kisumu Cancer Centre
Kisumu Cancer Centre
Kisumu Cancer Centre
Kisumu Cancer Centre
Kisumu Cancer Centre
Kisumu Cancer Centre
Kisumu Cancer Centre
Kisumu Cancer Centre
Kisumu Cancer Centre
Kisumu Cancer Centre
Kisumu Cancer Centre
Kisumu Cancer Centre
Kisumu Cancer Centre
Kisumu Cancer Centre
Kisumu Cancer Centre
Kisumu Cancer Centre
Kisumu Cancer Centre
Kisumu Cancer Centre
Kisumu Cancer Centre
Kisumu Cancer Centre
Kisumu Cancer Centre
Kisumu Cancer Centre
Kisumu Cancer Centre
Kisumu Cancer Centre
Kisumu Cancer Centre
Kisumu Cancer Centre
Nyeri Cancer Centre
Nyeri Cancer Centre
Nyeri Cancer Centre
Nyeri Cancer Centre
Nyeri Cancer Centre
Nyeri Cancer Centre
Nyeri Cancer Centre
Nyeri Cancer Centre
Nyeri Cancer Centre
Nyeri Cancer Centre
Nyeri Cancer Centre
Nyeri Cancer Centre
Nyeri Cancer Centre

Nyeri Cancer Centre
Nyeri Cancer Centre
Nyeri Cancer Centre
Nyeri Cancer Centre
Nyeri Cancer Centre
Nyeri Cancer Centre
Nyeri Cancer Centre
Nyeri Cancer Centre
Nyeri Cancer Centre
Nyeri Cancer Centre
Nyeri Cancer Centre
Nyeri Cancer Centre
Nyeri Cancer Centre
Nyeri Cancer Centre
Nyeri Cancer Centre
Nyeri Cancer Centre
Nyeri Cancer Centre
Nyeri Cancer Centre
Nyeri Cancer Centre
Nyeri Cancer Centre
Nyeri Cancer Centre
Nyeri Cancer Centre
Nyeri Cancer Centre
Nyeri Cancer Centre
Nyeri Cancer Centre
Nyeri Cancer Centre
Nyeri Cancer Centre
Nyeri Cancer Centre
Nyeri Cancer Centre
Nyeri Cancer Centre
Nyeri Cancer Centre
Nyeri Cancer Centre
Nyeri Cancer Centre
Nyeri Cancer Centre
Nyeri Cancer Centre
Nyeri Cancer Centre
Nyeri Cancer Centre
Nyeri Cancer Centre
Nyeri Cancer Centre
Nyeri Cancer Centre
Nyeri Cancer Centre
Nyeri Cancer Centre
Nyeri Cancer Centre
Nyeri Cancer Centre
Nyeri Cancer Centre
Nyeri Cancer Centre
Nyeri Cancer Centre
Nyeri Cancer Centre
Nyeri Cancer Centre
Nyeri Cancer Centre

Nyeri Cancer Centre
Nyeri Cancer Centre
Nyeri Cancer Centre
Nyeri Cancer Centre
Nyeri Cancer Centre
Nyeri Cancer Centre
Nyeri Cancer Centre
Nyeri Cancer Centre
Nyeri Cancer Centre
Nyeri Cancer Centre
Nyeri Cancer Centre
Nyeri Cancer Centre
Nyeri Cancer Centre
Nyeri Cancer Centre
Nyeri Cancer Centre
Nyeri Cancer Centre
Nyeri Cancer Centre
Nyeri Cancer Centre
Nyeri Cancer Centre
Nyeri Cancer Centre
Nyeri Cancer Centre
Nyeri Cancer Centre
Nyeri Cancer Centre
Nyeri Cancer Centre
Kenyatta National Hospital
Kenyatta National Hospital
Kenyatta National Hospital
Kenyatta National Hospital
Kenyatta National Hospital
Kenyatta National Hospital
Kenyatta National Hospital
Kenyatta National Hospital
Kenyatta National Hospital
Kenyatta National Hospital
Kenyatta National Hospital
Kenyatta National Hospital
Kenyatta National Hospital
Kenyatta National Hospital
Kenyatta National Hospital
Kenyatta National Hospital
Kenyatta National Hospital
Kenyatta National Hospital
Kenyatta National Hospital
Kenyatta National Hospital
Kenyatta National Hospital
Kenyatta National Hospital
Kenyatta National Hospital
Kenyatta National Hospital
Kenyatta National Hospital
Kenyatta National Hospital

Kenyatta National Hospital
Kenyatta National Hospital
Kenyatta National Hospital
Kenyatta National Hospital
Kenyatta National Hospital
Kenyatta National Hospital
Kenyatta National Hospital
Kenyatta National Hospital
Kenyatta National Hospital
Kenyatta National Hospital
Kenyatta National Hospital
Kenyatta National Hospital
Kenyatta National Hospital
Kenyatta National Hospital
Kenyatta National Hospital
Kenyatta National Hospital
Kenyatta National Hospital
Kenyatta National Hospital
Kenyatta National Hospital
Kenyatta National Hospital
Kenyatta National Hospital
Kenyatta National Hospital
Kenyatta National Hospital
Kenyatta National Hospital
Kenyatta National Hospital
Kenyatta National Hospital
Kenyatta National Hospital
Kenyatta National Hospital
Kenyatta National Hospital
Kenyatta National Hospital
Kenyatta National Hospital
Kenyatta National Hospital
Kenyatta National Hospital
Kenyatta National Hospital
Kenyatta National Hospital
Kenyatta National Hospital
Kenyatta National Hospital
Kenyatta National Hospital
Kenyatta National Hospital
Kenyatta National Hospital
Kenyatta National Hospital
Kenyatta National Hospital
Kenyatta National Hospital
Kenyatta National Hospital
Kenyatta National Hospital
Kenyatta National Hospital
Kenyatta National Hospital
Kenyatta National Hospital
Kenyatta National Hospital
Kenyatta National Hospital

Kenyatta National Hospital
Kenyatta National Hospital
Kenyatta National Hospital
Kenyatta National Hospital
Kenyatta National Hospital
Kenyatta National Hospital
Kenyatta National Hospital
Kenyatta National Hospital
Kenyatta National Hospital
Kenyatta National Hospital
Kenyatta National Hospital
Kenyatta National Hospital
Kenyatta National Hospital
Kenyatta National Hospital
Kenyatta National Hospital
Kenyatta National Hospital
Kenyatta University Teaching Refferal and Research Hospital
Kenyatta University Teaching Refferal and Research Hospital
Kenyatta University Teaching Refferal and Research Hospital
Kenyatta University Teaching Refferal and Research Hospital
Kenyatta University Teaching Refferal and Research Hospital
Kenyatta University Teaching Refferal and Research Hospital
Kenyatta University Teaching Refferal and Research Hospital
Kenyatta University Teaching Refferal and Research Hospital
Kenyatta University Teaching Refferal and Research Hospital
Kenyatta University Teaching Refferal and Research Hospital
Kenyatta University Teaching Refferal and Research Hospital
Kenyatta University Teaching Refferal and Research Hospital
Kenyatta University Teaching Refferal and Research Hospital
Kenyatta University Teaching Refferal and Research Hospital
Kenyatta University Teaching Refferal and Research Hospital
Kenyatta University Teaching Refferal and Research Hospital
Kenyatta University Teaching Refferal and Research Hospital
Kenyatta University Teaching Refferal and Research Hospital
Kenyatta University Teaching Refferal and Research Hospital
Kenyatta University Teaching Refferal and Research Hospital
Kenyatta University Teaching Refferal and Research Hospital
Kenyatta University Teaching Refferal and Research Hospital
Kenyatta University Teaching Refferal and Research Hospital
Kenyatta University Teaching Refferal and Research Hospital
Kenyatta University Teaching Refferal and Research Hospital
Kenyatta University Teaching Refferal and Research Hospital
Kenyatta University Teaching Refferal and Research Hospital
Kenyatta University Teaching Refferal and Research Hospital
Kenyatta University Teaching Refferal and Research Hospital
Kenyatta University Teaching Refferal and Research Hospital
Kenyatta University Teaching Refferal and Research Hospital
Kenyatta University Teaching Refferal and Research Hospital
Kenyatta University Teaching Refferal and Research Hospital
Kenyatta University Teaching Refferal and Research Hospital

Kenyatta University Teaching Refferal and Research Hospital
Kenyatta University Teaching Refferal and Research Hospital
Kenyatta University Teaching Refferal and Research Hospital
Kenyatta University Teaching Refferal and Research Hospital
Kenyatta University Teaching Refferal and Research Hospital
Kenyatta University Teaching Refferal and Research Hospital
Kenyatta University Teaching Refferal and Research Hospital
Kenyatta University Teaching Refferal and Research Hospital
Kenyatta University Teaching Refferal and Research Hospital
Kenyatta University Teaching Refferal and Research Hospital
Kenyatta University Teaching Refferal and Research Hospital
Kenyatta University Teaching Refferal and Research Hospital
Kenyatta University Teaching Refferal and Research Hospital
Kenyatta University Teaching Refferal and Research Hospital
Kenyatta University Teaching Refferal and Research Hospital
Kenyatta University Teaching Refferal and Research Hospital
Kenyatta University Teaching Refferal and Research Hospital
Kenyatta University Teaching Refferal and Research Hospital
Kenyatta University Teaching Refferal and Research Hospital
Kenyatta University Teaching Refferal and Research Hospital
Kenyatta University Teaching Refferal and Research Hospital
Kenyatta University Teaching Refferal and Research Hospital
Kenyatta University Teaching Refferal and Research Hospital
Kenyatta University Teaching Refferal and Research Hospital
Kenyatta University Teaching Refferal and Research Hospital
Kenyatta University Teaching Refferal and Research Hospital
Kenyatta University Teaching Refferal and Research Hospital
Kenyatta University Teaching Refferal and Research Hospital
Kenyatta University Teaching Refferal and Research Hospital
Kenyatta University Teaching Refferal and Research Hospital
Kenyatta University Teaching Refferal and Research Hospital
Kenyatta University Teaching Refferal and Research Hospital
Kenyatta University Teaching Refferal and Research Hospital
Kenyatta University Teaching Refferal and Research Hospital
Kenyatta University Teaching Refferal and Research Hospital
Kenyatta University Teaching Refferal and Research Hospital
Kenyatta University Teaching Refferal and Research Hospital
Kenyatta University Teaching Refferal and Research Hospital
Kenyatta University Teaching Refferal and Research Hospital
Kenyatta University Teaching Refferal and Research Hospital
Kenyatta University Teaching Refferal and Research Hospital
Kenyatta University Teaching Refferal and Research Hospital
Kenyatta University Teaching Refferal and Research Hospital
Kenyatta University Teaching Refferal and Research Hospital
Kenyatta University Teaching Refferal and Research Hospital
Kenyatta University Teaching Refferal and Research Hospital
Kenyatta University Teaching Refferal and Research Hospital
Kenyatta University Teaching Refferal and Research Hospital
Kenyatta University Teaching Refferal and Research Hospital
Kenyatta University Teaching Refferal and Research Hospital

Kenyatta University Teaching Refferal and Research Hospital
Kenyatta University Teaching Refferal and Research Hospital
Kenyatta University Teaching Refferal and Research Hospital
Kenyatta University Teaching Refferal and Research Hospital
Kenyatta University Teaching Refferal and Research Hospital
Kenyatta University Teaching Refferal and Research Hospital
Kenyatta University Teaching Refferal and Research Hospital
Kenyatta University Teaching Refferal and Research Hospital
Kenyatta University Teaching Refferal and Research Hospital
Meru Cancer Centre
Meru Cancer Centre
Meru Cancer Centre
Meru Cancer Centre
Meru Cancer Centre
Meru Cancer Centre
Meru Cancer Centre
Meru Cancer Centre
Meru Cancer Centre
Meru Cancer Centre
Meru Cancer Centre
Meru Cancer Centre
Meru Cancer Centre
Meru Cancer Centre
Meru Cancer Centre
Meru Cancer Centre
Meru Cancer Centre
Meru Cancer Centre
Meru Cancer Centre
Meru Cancer Centre
Meru Cancer Centre
Meru Cancer Centre
Meru Cancer Centre
Meru Cancer Centre
Meru Cancer Centre
Meru Cancer Centre
Meru Cancer Centre
Meru Cancer Centre
Meru Cancer Centre
Meru Cancer Centre
Meru Cancer Centre
Meru Cancer Centre
Meru Cancer Centre
Meru Cancer Centre
Meru Cancer Centre
Meru Cancer Centre
Meru Cancer Centre
Meru Cancer Centre
Meru Cancer Centre
Meru Cancer Centre
Meru Cancer Centre

Meru Cancer Centre
Meru Cancer Centre
Meru Cancer Centre
Meru Cancer Centre
Meru Cancer Centre
Meru Cancer Centre
Meru Cancer Centre
Meru Cancer Centre
Meru Cancer Centre
Meru Cancer Centre
Meru Cancer Centre
Meru Cancer Centre
Meru Cancer Centre
Meru Cancer Centre
Meru Cancer Centre
Meru Cancer Centre
Meru Cancer Centre
Meru Cancer Centre
Meru Cancer Centre
Meru Cancer Centre
Meru Cancer Centre
Meru Cancer Centre
Meru Cancer Centre
Meru Cancer Centre
Meru Cancer Centre
Meru Cancer Centre
Meru Cancer Centre
Meru Cancer Centre
Meru Cancer Centre
Meru Cancer Centre
Meru Cancer Centre
Meru Cancer Centre
Meru Cancer Centre
Meru Cancer Centre
Meru Cancer Centre
Meru Cancer Centre
Meru Cancer Centre
Meru Cancer Centre
Meru Cancer Centre
Meru Cancer Centre
Meru Cancer Centre
Meru Cancer Centre
Meru Cancer Centre
Meru Cancer Centre
Meru Cancer Centre
Meru Cancer Centre
Meru Cancer Centre
Meru Cancer Centre
Meru Cancer Centre
Meru Cancer Centre

Meru Cancer Centre
Meru Cancer Centre
Meru Cancer Centre
Meru Cancer Centre
Meru Cancer Centre
Kenyatta University Teaching Refferal and Research Hospital
Kenyatta University Teaching Refferal and Research Hospital
Kenyatta University Teaching Refferal and Research Hospital
Kenyatta University Teaching Refferal and Research Hospital
Kenyatta University Teaching Refferal and Research Hospital
Kenyatta University Teaching Refferal and Research Hospital
Kenyatta University Teaching Refferal and Research Hospital
Kenyatta University Teaching Refferal and Research Hospital
Kenyatta University Teaching Refferal and Research Hospital
Kenyatta University Teaching Refferal and Research Hospital
Kenyatta University Teaching Refferal and Research Hospital
Kenyatta University Teaching Refferal and Research Hospital
Kenyatta University Teaching Refferal and Research Hospital
Kenyatta University Teaching Refferal and Research Hospital
Kenyatta University Teaching Refferal and Research Hospital
Kenyatta University Teaching Refferal and Research Hospital
Kenyatta University Teaching Refferal and Research Hospital
Kenyatta University Teaching Refferal and Research Hospital
Kenyatta University Teaching Refferal and Research Hospital
Kenyatta University Teaching Refferal and Research Hospital
Kenyatta University Teaching Refferal and Research Hospital
Kenyatta University Teaching Refferal and Research Hospital
Kenyatta University Teaching Refferal and Research Hospital
Kenyatta University Teaching Refferal and Research Hospital
Kenyatta University Teaching Refferal and Research Hospital
Kenyatta University Teaching Refferal and Research Hospital
Kenyatta University Teaching Refferal and Research Hospital
Kenyatta University Teaching Refferal and Research Hospital
Kenyatta University Teaching Refferal and Research Hospital
Kenyatta University Teaching Refferal and Research Hospital
Kenyatta University Teaching Refferal and Research Hospital
Kenyatta University Teaching Refferal and Research Hospital
Kenyatta University Teaching Refferal and Research Hospital
Kenyatta University Teaching Refferal and Research Hospital
Kenyatta University Teaching Refferal and Research Hospital
Kenyatta University Teaching Refferal and Research Hospital
Kenyatta University Teaching Refferal and Research Hospital
Kenyatta University Teaching Refferal and Research Hospital
Kenyatta University Teaching Refferal and Research Hospital
Kenyatta University Teaching Refferal and Research Hospital
Kenyatta University Teaching Refferal and Research Hospital
Kenyatta University Teaching Refferal and Research Hospital
Kenyatta University Teaching Refferal and Research Hospital
Kenyatta University Teaching Refferal and Research Hospital
Kenyatta University Teaching Refferal and Research Hospital

Kenyatta University Teaching Refferal and Research Hospital
Kenyatta University Teaching Refferal and Research Hospital
Kenyatta University Teaching Refferal and Research Hospital
Kenyatta University Teaching Refferal and Research Hospital
Kenyatta University Teaching Refferal and Research Hospital
Kenyatta University Teaching Refferal and Research Hospital
Kenyatta University Teaching Refferal and Research Hospital
Kenyatta University Teaching Refferal and Research Hospital
Kenyatta University Teaching Refferal and Research Hospital
Kenyatta University Teaching Refferal and Research Hospital
Kenyatta University Teaching Refferal and Research Hospital
Kenyatta University Teaching Refferal and Research Hospital
Kenyatta University Teaching Refferal and Research Hospital
Kenyatta University Teaching Refferal and Research Hospital
Kenyatta University Teaching Refferal and Research Hospital
Kenyatta University Teaching Refferal and Research Hospital
Kenyatta University Teaching Refferal and Research Hospital
Kenyatta University Teaching Refferal and Research Hospital
Kenyatta University Teaching Refferal and Research Hospital
Kenyatta University Teaching Refferal and Research Hospital
Kenyatta University Teaching Refferal and Research Hospital
Kenyatta University Teaching Refferal and Research Hospital
Kenyatta University Teaching Refferal and Research Hospital
Kenyatta University Teaching Refferal and Research Hospital
Kenyatta University Teaching Refferal and Research Hospital
Kenyatta University Teaching Refferal and Research Hospital
Kenyatta University Teaching Refferal and Research Hospital
Kenyatta University Teaching Refferal and Research Hospital
Kenyatta University Teaching Refferal and Research Hospital
Kenyatta University Teaching Refferal and Research Hospital
Kenyatta University Teaching Refferal and Research Hospital
Kenyatta University Teaching Refferal and Research Hospital
Kenyatta University Teaching Refferal and Research Hospital
Kenyatta University Teaching Refferal and Research Hospital
Kenyatta University Teaching Refferal and Research Hospital
Kenyatta University Teaching Refferal and Research Hospital
Kenyatta University Teaching Refferal and Research Hospital
Kenyatta University Teaching Refferal and Research Hospital
Kenyatta University Teaching Refferal and Research Hospital
Kenyatta University Teaching Refferal and Research Hospital
Kenyatta University Teaching Refferal and Research Hospital
Kenyatta University Teaching Refferal and Research Hospital
Kenyatta University Teaching Refferal and Research Hospital
Kenyatta University Teaching Refferal and Research Hospital
Kenyatta University Teaching Refferal and Research Hospital
Kenyatta University Teaching Refferal and Research Hospital
Kenyatta University Teaching Refferal and Research Hospital
Kenyatta University Teaching Refferal and Research Hospital
Kenyatta University Teaching Refferal and Research Hospital
Kenyatta University Teaching Refferal and Research Hospital

Kenyatta University Teaching Refferal and Research Hospital
Kenyatta University Teaching Refferal and Research Hospital
Kenyatta University Teaching Refferal and Research Hospital
Kenyatta University Teaching Refferal and Research Hospital
Kenyatta University Teaching Refferal and Research Hospital
Kenyatta University Teaching Refferal and Research Hospital
Kenyatta University Teaching Refferal and Research Hospital
Kenyatta University Teaching Refferal and Research Hospital
Kenyatta University Teaching Refferal and Research Hospital
Kenyatta University Teaching Refferal and Research Hospital
Kenyatta University Teaching Refferal and Research Hospital
Kenyatta University Teaching Refferal and Research Hospital
Kenyatta University Teaching Refferal and Research Hospital
Kenyatta University Teaching Refferal and Research Hospital
Kenyatta University Teaching Refferal and Research Hospital
Kenyatta University Teaching Refferal and Research Hospital
Kenyatta University Teaching Refferal and Research Hospital
Kenyatta University Teaching Refferal and Research Hospital
Kenyatta University Teaching Refferal and Research Hospital
Kenyatta University Teaching Refferal and Research Hospital
Kenyatta University Teaching Refferal and Research Hospital
Kenyatta University Teaching Refferal and Research Hospital
Kenyatta University Teaching Refferal and Research Hospital
Kenyatta University Teaching Refferal and Research Hospital
Kenyatta University Teaching Refferal and Research Hospital
Kenyatta University Teaching Refferal and Research Hospital
Kenyatta University Teaching Refferal and Research Hospital
Kenyatta University Teaching Refferal and Research Hospital
Kenyatta University Teaching Refferal and Research Hospital
Kenyatta University Teaching Refferal and Research Hospital
Kenyatta University Teaching Refferal and Research Hospital
Kenyatta University Teaching Refferal and Research Hospital
Kenyatta University Teaching Refferal and Research Hospital
Kenyatta University Teaching Refferal and Research Hospital
Kenyatta University Teaching Refferal and Research Hospital
Kenyatta University Teaching Refferal and Research Hospital
Kenyatta University Teaching Refferal and Research Hospital
Kenyatta University Teaching Refferal and Research Hospital
Kenyatta University Teaching Refferal and Research Hospital
Kenyatta University Teaching Refferal and Research Hospital
Mombasa Cancer Centre
Mombasa Cancer Centre
Mombasa Cancer Centre
Mombasa Cancer Centre
Mombasa Cancer Centre
Mombasa Cancer Centre
Mombasa Cancer Centre
Mombasa Cancer Centre
Mombasa Cancer Centre
Mombasa Cancer Centre

Mombasa Cancer Centre
Mombasa Cancer Centre
Mombasa Cancer Centre
Mombasa Cancer Centre
Mombasa Cancer Centre
Mombasa Cancer Centre
Mombasa Cancer Centre
Mombasa Cancer Centre
Mombasa Cancer Centre
Mombasa Cancer Centre
Mombasa Cancer Centre
Mombasa Cancer Centre
Mombasa Cancer Centre
Mombasa Cancer Centre
Mombasa Cancer Centre
Mombasa Cancer Centre
Mombasa Cancer Centre
Mombasa Cancer Centre
Mombasa Cancer Centre
Mombasa Cancer Centre
Mombasa Cancer Centre
Mombasa Cancer Centre
Mombasa Cancer Centre
Mombasa Cancer Centre
Mombasa Cancer Centre
Mombasa Cancer Centre
Mombasa Cancer Centre
Mombasa Cancer Centre
Mombasa Cancer Centre
Mombasa Cancer Centre
Mombasa Cancer Centre
Mombasa Cancer Centre
Mombasa Cancer Centre
Mombasa Cancer Centre
Mombasa Cancer Centre
Mombasa Cancer Centre
Mombasa Cancer Centre
Mombasa Cancer Centre
Mombasa Cancer Centre
Mombasa Cancer Centre
Mombasa Cancer Centre
Mombasa Cancer Centre
Mombasa Cancer Centre
Mombasa Cancer Centre
Mombasa Cancer Centre
Mombasa Cancer Centre
Mombasa Cancer Centre
Mombasa Cancer Centre
Mombasa Cancer Centre
Mombasa Cancer Centre

Mombasa Cancer Centre
Mombasa Cancer Centre
Mombasa Cancer Centre
Mombasa Cancer Centre
Mombasa Cancer Centre
Mombasa Cancer Centre
Mombasa Cancer Centre
Mombasa Cancer Centre
Mombasa Cancer Centre
Mombasa Cancer Centre
Mombasa Cancer Centre
Mombasa Cancer Centre
Mombasa Cancer Centre
Mombasa Cancer Centre
Mombasa Cancer Centre
Mombasa Cancer Centre
Mombasa Cancer Centre
Mombasa Cancer Centre
Mombasa Cancer Centre
Mombasa Cancer Centre
Mombasa Cancer Centre
Mombasa Cancer Centre
Meru Cancer Centre
Meru Cancer Centre
Meru Cancer Centre
Meru Cancer Centre
Meru Cancer Centre
Meru Cancer Centre
Meru Cancer Centre
Meru Cancer Centre
Meru Cancer Centre
Meru Cancer Centre
Meru Cancer Centre
Meru Cancer Centre
Meru Cancer Centre
Meru Cancer Centre
Meru Cancer Centre
Meru Cancer Centre
Meru Cancer Centre
Meru Cancer Centre
Meru Cancer Centre
Meru Cancer Centre
Meru Cancer Centre
Meru Cancer Centre
Meru Cancer Centre
Meru Cancer Centre
Meru Cancer Centre
Meru Cancer Centre
Meru Cancer Centre
Meru Cancer Centre

Meru Cancer Centre
Meru Cancer Centre
Meru Cancer Centre
Meru Cancer Centre
Meru Cancer Centre
Meru Cancer Centre
Meru Cancer Centre
Meru Cancer Centre
Meru Cancer Centre
Meru Cancer Centre
Meru Cancer Centre
Meru Cancer Centre
Meru Cancer Centre
Meru Cancer Centre
Meru Cancer Centre
Meru Cancer Centre
Meru Cancer Centre
Meru Cancer Centre
Meru Cancer Centre
Meru Cancer Centre
Meru Cancer Centre
Meru Cancer Centre
Meru Cancer Centre
Meru Cancer Centre
Meru Cancer Centre
Meru Cancer Centre
Meru Cancer Centre
Meru Cancer Centre
Meru Cancer Centre
Meru Cancer Centre
Meru Cancer Centre
Meru Cancer Centre
Meru Cancer Centre
Meru Cancer Centre
Meru Cancer Centre
Meru Cancer Centre
Machakos Cancer Centre
Taita Taveta Cancer Centre
Taita Taveta Cancer Centre
Kenyatta National Hospital
Kenyatta National Hospital
Kenyatta National Hospital
Kenyatta National Hospital
Kenyatta National Hospital
Kenyatta National Hospital
Kenyatta National Hospital
Kenyatta National Hospital
Kenyatta National Hospital
Kenyatta National Hospital
Kenyatta National Hospital

Kenyatta National Hospital
Kenyatta National Hospital
Kenyatta National Hospital
Machakos Cancer Centre
Kenyatta National Hospital
Machakos Cancer Centre
Taita Taveta Cancer Centre
Taita Taveta Cancer Centre
Machakos Cancer Centre
Taita Taveta Cancer Centre
Kenyatta National Hospital
Kenyatta National Hospital
Kenyatta National Hospital
Taita Taveta Cancer Centre
Kenyatta National Hospital
Kenyatta National Hospital
Kenyatta National Hospital
Kenyatta National Hospital
Kenyatta National Hospital
Kenyatta National Hospital
Machakos Cancer Centre
Kenyatta National Hospital
Kenyatta National Hospital
Kenyatta National Hospital
Kenyatta National Hospital
Kenyatta National Hospital
Taita Taveta Cancer Centre
Machakos Cancer Centre
Taita Taveta Cancer Centre
Machakos Cancer Centre
Machakos Cancer Centre
Taita Taveta Cancer Centre
Machakos Cancer Centre
Kenyatta National Hospital
Machakos Cancer Centre
Taita Taveta Cancer Centre
Machakos Cancer Centre
Kenyatta National Hospital
Taita Taveta Cancer Centre
Kenyatta National Hospital
Meru Cancer Centre
Meru Cancer Centre
Meru Cancer Centre
Meru Cancer Centre
Makueni Cancer Centre
Makueni Cancer Centre
Makueni Cancer Centre
Makueni Cancer Centre
Makueni Cancer Centre
Meru Cancer Centre

Meru Cancer Centre
Meru Cancer Centre
Meru Cancer Centre
Makueni Cancer Centre
Makueni Cancer Centre
Makueni Cancer Centre
Makueni Cancer Centre
Meru Cancer Centre
Meru Cancer Centre
Meru Cancer Centre
Meru Cancer Centre
Meru Cancer Centre
Meru Cancer Centre
Makueni Cancer Centre
Meru Cancer Centre
Meru Cancer Centre
Makueni Cancer Centre
Makueni Cancer Centre
Makueni Cancer Centre
Makueni Cancer Centre
Makueni Cancer Centre
Makueni Cancer Centre
Makueni Cancer Centre
Meru Cancer Centre
Meru Cancer Centre
Meru Cancer Centre
Meru Cancer Centre
Makueni Cancer Centre
Makueni Cancer Centre
Makueni Cancer Centre
Makueni Cancer Centre
Meru Cancer Centre
Meru Cancer Centre
Meru Cancer Centre
Meru Cancer Centre
Meru Cancer Centre
Meru Cancer Centre
Meru Cancer Centre
Meru Cancer Centre
Makueni Cancer Centre
Makueni Cancer Centre
Makueni Cancer Centre
Makueni Cancer Centre
Makueni Cancer Centre
Meru Cancer Centre
Meru Cancer Centre
Makueni Cancer Centre
Meru Cancer Centre
Meru Cancer Centre
Makueni Cancer Centre

Makueni Cancer Centre
Makueni Cancer Centre
Makueni Cancer Centre
Meru Cancer Centre
Makueni Cancer Centre
Makueni Cancer Centre
Makueni Cancer Centre
Makueni Cancer Centre
Meru Cancer Centre
Meru Cancer Centre
Meru Cancer Centre
Meru Cancer Centre
Kenyatta University Teaching Refferal and Research Hospital
Kenyatta University Teaching Refferal and Research Hospital
Kenyatta National Hospital
Kenyatta National Hospital
Kenyatta National Hospital
Kenyatta University Teaching Refferal and Research Hospital
Kenyatta University Teaching Refferal and Research Hospital
Kenyatta University Teaching Refferal and Research Hospital
Kenyatta National Hospital
Kenyatta National Hospital
Kenyatta University Teaching Refferal and Research Hospital
Kenyatta University Teaching Refferal and Research Hospital
Kenyatta University Teaching Refferal and Research Hospital
Kenyatta University Teaching Refferal and Research Hospital
Kenyatta University Teaching Refferal and Research Hospital
Kenyatta University Teaching Refferal and Research Hospital
Makueni Cancer Centre
Makueni Cancer Centre
Makueni Cancer Centre
Makueni Cancer Centre
Makueni Cancer Centre
Makueni Cancer Centre
Makueni Cancer Centre
Makueni Cancer Centre
Makueni Cancer Centre
Makueni Cancer Centre
Makueni Cancer Centre
Makueni Cancer Centre
Makueni Cancer Centre
Makueni Cancer Centre
Makueni Cancer Centre
Makueni Cancer Centre
Makueni Cancer Centre
Makueni Cancer Centre
Makueni Cancer Centre
Makueni Cancer Centre
Makueni Cancer Centre
Makueni Cancer Centre

Makueni Cancer Centre
Makueni Cancer Centre
Makueni Cancer Centre
Makueni Cancer Centre
Makueni Cancer Centre
Makueni Cancer Centre
Makueni Cancer Centre
Makueni Cancer Centre
Makueni Cancer Centre
Makueni Cancer Centre
Makueni Cancer Centre
Makueni Cancer Centre
Makueni Cancer Centre
Makueni Cancer Centre
Makueni Cancer Centre
Makueni Cancer Centre
Makueni Cancer Centre
Makueni Cancer Centre
Makueni Cancer Centre
Makueni Cancer Centre
Makueni Cancer Centre
Makueni Cancer Centre
Makueni Cancer Centre
Makueni Cancer Centre
Makueni Cancer Centre
Makueni Cancer Centre
Makueni Cancer Centre
Makueni Cancer Centre
Makueni Cancer Centre
Makueni Cancer Centre
Makueni Cancer Centre
Makueni Cancer Centre
Makueni Cancer Centre
Makueni Cancer Centre
Makueni Cancer Centre
Makueni Cancer Centre
Makueni Cancer Centre
Makueni Cancer Centre
Makueni Cancer Centre
Makueni Cancer Centre
Makueni Cancer Centre
Makueni Cancer Centre
Makueni Cancer Centre
Makueni Cancer Centre
Makueni Cancer Centre
Makueni Cancer Centre
Makueni Cancer Centre
Makueni Cancer Centre
Makueni Cancer Centre
Makueni Cancer Centre

Makueni Cancer Centre
Makueni Cancer Centre
Makueni Cancer Centre
Makueni Cancer Centre
Makueni Cancer Centre
Makueni Cancer Centre
Makueni Cancer Centre
Makueni Cancer Centre
Makueni Cancer Centre
Makueni Cancer Centre
Makueni Cancer Centre
Makueni Cancer Centre
Makueni Cancer Centre
Makueni Cancer Centre
Makueni Cancer Centre
Makueni Cancer Centre
Makueni Cancer Centre
Makueni Cancer Centre
Makueni Cancer Centre
Makueni Cancer Centre
Makueni Cancer Centre
Makueni Cancer Centre
Makueni Cancer Centre
Meru Cancer Centre
Meru Cancer Centre
Meru Cancer Centre
Meru Cancer Centre
Meru Cancer Centre
Meru Cancer Centre
Meru Cancer Centre
Meru Cancer Centre
Meru Cancer Centre
Meru Cancer Centre
Meru Cancer Centre
Meru Cancer Centre
Meru Cancer Centre
Meru Cancer Centre
Meru Cancer Centre
Meru Cancer Centre
Meru Cancer Centre
Meru Cancer Centre
Meru Cancer Centre
Meru Cancer Centre
Meru Cancer Centre
Meru Cancer Centre
Meru Cancer Centre
Meru Cancer Centre
Meru Cancer Centre
Meru Cancer Centre
Meru Cancer Centre

Meru Cancer Centre
Meru Cancer Centre
Meru Cancer Centre
Meru Cancer Centre
Meru Cancer Centre
Meru Cancer Centre
Meru Cancer Centre
Meru Cancer Centre
Meru Cancer Centre
Meru Cancer Centre
Meru Cancer Centre
Meru Cancer Centre
Meru Cancer Centre
Meru Cancer Centre
Meru Cancer Centre
Meru Cancer Centre
Meru Cancer Centre
Meru Cancer Centre
Meru Cancer Centre
Meru Cancer Centre
Meru Cancer Centre
Meru Cancer Centre
Meru Cancer Centre
Meru Cancer Centre
Meru Cancer Centre
Meru Cancer Centre
Meru Cancer Centre
Meru Cancer Centre
Meru Cancer Centre
Meru Cancer Centre
Meru Cancer Centre
Meru Cancer Centre
Meru Cancer Centre
Meru Cancer Centre
Meru Cancer Centre
Meru Cancer Centre
Meru Cancer Centre
Meru Cancer Centre
Meru Cancer Centre
Meru Cancer Centre
Meru Cancer Centre
Meru Cancer Centre
Meru Cancer Centre
Meru Cancer Centre
Meru Cancer Centre
Meru Cancer Centre
Meru Cancer Centre
Meru Cancer Centre
Meru Cancer Centre
Meru Cancer Centre

Meru Cancer Centre
Meru Cancer Centre
Meru Cancer Centre
Meru Cancer Centre
Meru Cancer Centre
Meru Cancer Centre
Meru Cancer Centre
Meru Cancer Centre
Meru Cancer Centre
Meru Cancer Centre
Meru Cancer Centre
Meru Cancer Centre
Meru Cancer Centre
Meru Cancer Centre
Meru Cancer Centre
Meru Cancer Centre
Meru Cancer Centre
Meru Cancer Centre
Meru Cancer Centre
Meru Cancer Centre
Meru Cancer Centre
Meru Cancer Centre
Meru Cancer Centre
Meru Cancer Centre
Meru Cancer Centre
Meru Cancer Centre
Meru Cancer Centre
Meru Cancer Centre
Meru Cancer Centre
Meru Cancer Centre
Meru Cancer Centre
Meru Cancer Centre
Meru Cancer Centre
Meru Cancer Centre
Meru Cancer Centre
Meru Cancer Centre
Meru Cancer Centre
Meru Cancer Centre
Meru Cancer Centre
Meru Cancer Centre
Meru Cancer Centre
Nyeri Cancer Centre
Kenyatta University Teaching Refferal and Research Hospital
Kenyatta National Hospital
Kenyatta University Teaching Refferal and Research Hospital
Garissa Cancer Centre
Kenyatta University Teaching Refferal and Research Hospital
Mombasa Cancer Centre
Kenyatta University Teaching Refferal and Research Hospital
Kenyatta University Teaching Refferal and Research Hospital

Kenyatta University Teaching Refferal and Research Hospital
Kenyatta University Teaching Refferal and Research Hospital
Nyeri Cancer Centre
Nyeri Cancer Centre
Nyeri Cancer Centre
Nyeri Cancer Centre
Nyeri Cancer Centre
Kenyatta University Teaching Refferal and Research Hospital
Kenyatta University Teaching Refferal and Research Hospital
Kenyatta University Teaching Refferal and Research Hospital
Kenyatta University Teaching Refferal and Research Hospital
Kenyatta University Teaching Refferal and Research Hospital
Mombasa Cancer Centre
Nyeri Cancer Centre
Nyeri Cancer Centre
Nyeri Cancer Centre
Nyeri Cancer Centre
Kenyatta National Hospital
Kenyatta National Hospital
Kenyatta National Hospital
Nyeri Cancer Centre
Nyeri Cancer Centre
Nyeri Cancer Centre
Kenyatta National Hospital
Kenyatta National Hospital
Kenyatta National Hospital
Mombasa Cancer Centre
Mombasa Cancer Centre
Meru Cancer Centre
Meru Cancer Centre
Meru Cancer Centre
Kenyatta National Hospital
Kenyatta National Hospital
Kenyatta National Hospital
Kisumu Cancer Centre
Nyeri Cancer Centre
Nyeri Cancer Centre
Nyeri Cancer Centre
Nyeri Cancer Centre
Nyeri Cancer Centre
Nyeri Cancer Centre
Mombasa Cancer Centre
Kenyatta University Teaching Refferal and Research Hospital
Kenyatta University Teaching Refferal and Research Hospital
Kenyatta University Teaching Refferal and Research Hospital
Kenyatta National Hospital
Kenyatta National Hospital
Nyeri Cancer Centre
Kisumu Cancer Centre
Kisumu Cancer Centre

Kisumu Cancer Centre
Kisumu Cancer Centre
Kenyatta University Teaching Refferal and Research Hospital
Nyeri Cancer Centre
Kenyatta University Teaching Refferal and Research Hospital
Nyeri Cancer Centre
Kenyatta National Hospital
Kenyatta National Hospital
Nyeri Cancer Centre
Mombasa Cancer Centre
Nyeri Cancer Centre
Meru Cancer Centre
Meru Cancer Centre
Kenyatta National Hospital
Kenyatta National Hospital
Kenyatta National Hospital
Nyeri Cancer Centre
Kenyatta National Hospital
Meru Cancer Centre
Meru Cancer Centre
Meru Cancer Centre
Kenyatta University Teaching Refferal and Research Hospital
Kenyatta University Teaching Refferal and Research Hospital
Kenyatta University Teaching Refferal and Research Hospital
Machakos Cancer Centre
Nyeri Cancer Centre
Meru Cancer Centre
Mombasa Cancer Centre
Meru Cancer Centre
Meru Cancer Centre
Meru Cancer Centre
Nyeri Cancer Centre
Mombasa Cancer Centre
Nyeri Cancer Centre
Makueni Cancer Centre
Makueni Cancer Centre
Kenyatta University Teaching Refferal and Research Hospital
Kenyatta University Teaching Refferal and Research Hospital
Meru Cancer Centre
Kenyatta University Teaching Refferal and Research Hospital
Kenyatta National Hospital
Kenyatta National Hospital
Kenyatta National Hospital
Kenyatta National Hospital
Kenyatta University Teaching Refferal and Research Hospital
Kenyatta National Hospital
Kenyatta University Teaching Refferal and Research Hospital
Kenyatta University Teaching Refferal and Research Hospital
Kenyatta National Hospital
Meru Cancer Centre

Meru Cancer Centre
Meru Cancer Centre
Mombasa Cancer Centre
Mombasa Cancer Centre
Mombasa Cancer Centre
Makueni Cancer Centre
Makueni Cancer Centre
Makueni Cancer Centre
Kenyatta National Hospital
Kenyatta National Hospital
Kenyatta National Hospital
Makueni Cancer Centre
Makueni Cancer Centre
Makueni Cancer Centre
Makueni Cancer Centre
Makueni Cancer Centre
Makueni Cancer Centre
Makueni Cancer Centre
Kenyatta National Hospital
Meru Cancer Centre
Kenyatta National Hospital
Kenyatta National Hospital
Kenyatta National Hospital
Meru Cancer Centre
Meru Cancer Centre
Meru Cancer Centre
Meru Cancer Centre
Meru Cancer Centre
Kenyatta National Hospital
Taita Taveta Cancer Centre
Kenyatta National Hospital
Kenyatta National Hospital
Meru Cancer Centre
Meru Cancer Centre
Meru Cancer Centre
Kenyatta National Hospital
Makueni Cancer Centre
Makueni Cancer Centre
Kenyatta National Hospital
Kenyatta National Hospital
Kenyatta National Hospital
Kenyatta University Teaching Refferal and Research Hospital
Kenyatta University Teaching Refferal and Research Hospital
Kenyatta National Hospital
Garissa Cancer Centre
Nyeri Cancer Centre
Nakuru Cancer Centre
Nyeri Cancer Centre
Nyeri Cancer Centre
Nyeri Cancer Centre

Kakamega Cancer Centre
Kakamega Cancer Centre
Taita Taveta Cancer Centre
Kakamega Cancer Centre
Makueni Cancer Centre
Taita Taveta Cancer Centre
Makueni Cancer Centre
Meru Cancer Centre
Nyeri Cancer Centre
Nyeri Cancer Centre
Garissa Cancer Centre
Nakuru Cancer Centre
Nakuru Cancer Centre
Kakamega Cancer Centre
Nakuru Cancer Centre
Nakuru Cancer Centre
Kakamega Cancer Centre
Makueni Cancer Centre
Makueni Cancer Centre
Makueni Cancer Centre
Kakamega Cancer Centre
Kakamega Cancer Centre
Nakuru Cancer Centre
Taita Taveta Cancer Centre
Garissa Cancer Centre
Garissa Cancer Centre
Nyeri Cancer Centre
Makueni Cancer Centre
Kakamega Cancer Centre
Nakuru Cancer Centre
Makueni Cancer Centre
Makueni Cancer Centre
Makueni Cancer Centre
Makueni Cancer Centre
Nakuru Cancer Centre
Nakuru Cancer Centre
Nyeri Cancer Centre
Kakamega Cancer Centre
Kakamega Cancer Centre
Kakamega Cancer Centre
Garissa Cancer Centre
Kakamega Cancer Centre
Meru Cancer Centre
Bomet Cancer Centre
Meru Cancer Centre
Meru Cancer Centre
Nakuru Cancer Centre
Kakamega Cancer Centre
Kakamega Cancer Centre
Nyeri Cancer Centre

Mombasa Cancer Centre
Kakamega Cancer Centre
Machakos Cancer Centre
Makueni Cancer Centre
Makueni Cancer Centre
Nyeri Cancer Centre
Nyeri Cancer Centre
Mombasa Cancer Centre
Meru Cancer Centre
Meru Cancer Centre
Garissa Cancer Centre
Kakamega Cancer Centre
Kakamega Cancer Centre
Machakos Cancer Centre
Bomet Cancer Centre
Garissa Cancer Centre
Bomet Cancer Centre
Garissa Cancer Centre
Nakuru Cancer Centre
Nyeri Cancer Centre
Nyeri Cancer Centre
Nyeri Cancer Centre
Nyeri Cancer Centre
Taita Taveta Cancer Centre
Taita Taveta Cancer Centre
Taita Taveta Cancer Centre
Kisumu Cancer Centre
Kisumu Cancer Centre
Makueni Cancer Centre
Kisumu Cancer Centre
Kakamega Cancer Centre
Kisumu Cancer Centre
Machakos Cancer Centre
Machakos Cancer Centre
Machakos Cancer Centre
Meru Cancer Centre
Meru Cancer Centre
Meru Cancer Centre
Meru Cancer Centre
Taita Taveta Cancer Centre
Machakos Cancer Centre
Nakuru Cancer Centre
Kakamega Cancer Centre
Mombasa Cancer Centre
Makueni Cancer Centre
Bomet Cancer Centre
Makueni Cancer Centre
Bomet Cancer Centre
Makueni Cancer Centre
Mombasa Cancer Centre

Kakamega Cancer Centre
Nakuru Cancer Centre
Kakamega Cancer Centre
Kakamega Cancer Centre
Meru Cancer Centre
Meru Cancer Centre
Garissa Cancer Centre
Kakamega Cancer Centre
Kakamega Cancer Centre
Nyeri Cancer Centre
Meru Cancer Centre
Garissa Cancer Centre
Garissa Cancer Centre
Meru Cancer Centre
Meru Cancer Centre
Machakos Cancer Centre
Machakos Cancer Centre
Kakamega Cancer Centre
Makueni Cancer Centre
Nakuru Cancer Centre
Makueni Cancer Centre
Makueni Cancer Centre
Makueni Cancer Centre
Kakamega Cancer Centre
Nyeri Cancer Centre
Machakos Cancer Centre
Machakos Cancer Centre
Nyeri Cancer Centre
Nyeri Cancer Centre
Mombasa Cancer Centre
Kakamega Cancer Centre
Meru Cancer Centre
Taita Taveta Cancer Centre
Meru Cancer Centre
Meru Cancer Centre
Meru Cancer Centre
Garissa Cancer Centre
Nyeri Cancer Centre
Nyeri Cancer Centre
Makueni Cancer Centre
Makueni Cancer Centre
Makueni Cancer Centre
Nyeri Cancer Centre
Nyeri Cancer Centre
Nyeri Cancer Centre
Machakos Cancer Centre
Meru Cancer Centre
Meru Cancer Centre
Meru Cancer Centre
Meru Cancer Centre

Meru Cancer Centre
Meru Cancer Centre
Meru Cancer Centre
Meru Cancer Centre
Meru Cancer Centre
Meru Cancer Centre
Meru Cancer Centre
Meru Cancer Centre
Meru Cancer Centre
Nyeri Cancer Centre
Nyeri Cancer Centre
Makueni Cancer Centre
Makueni Cancer Centre
Makueni Cancer Centre
Makueni Cancer Centre
Makueni Cancer Centre
Makueni Cancer Centre
Makueni Cancer Centre
Nyeri Cancer Centre
Nyeri Cancer Centre
Nyeri Cancer Centre
Nyeri Cancer Centre
Kisumu Cancer Centre
Makueni Cancer Centre
Makueni Cancer Centre
Makueni Cancer Centre
Nyeri Cancer Centre
Nyeri Cancer Centre
Nyeri Cancer Centre
Nyeri Cancer Centre
Meru Cancer Centre
Meru Cancer Centre
Meru Cancer Centre
Meru Cancer Centre
Meru Cancer Centre
Meru Cancer Centre
Meru Cancer Centre
Meru Cancer Centre
Meru Cancer Centre
Meru Cancer Centre
Meru Cancer Centre
Meru Cancer Centre
Meru Cancer Centre
Meru Cancer Centre
Meru Cancer Centre
Meru Cancer Centre
Meru Cancer Centre
Meru Cancer Centre
Meru Cancer Centre
Makueni Cancer Centre

Makueni Cancer Centre
Makueni Cancer Centre
Makueni Cancer Centre
Makueni Cancer Centre
Makueni Cancer Centre
Makueni Cancer Centre
Makueni Cancer Centre
Makueni Cancer Centre
Makueni Cancer Centre
Makueni Cancer Centre
Machakos Cancer Centre
Machakos Cancer Centre
Nyeri Cancer Centre
Nyeri Cancer Centre
Kenyatta University Teaching Refferal and Research Hospital
Kenyatta University Teaching Refferal and Research Hospital
Kenyatta University Teaching Refferal and Research Hospital
Kenyatta University Teaching Refferal and Research Hospital
Kenyatta University Teaching Refferal and Research Hospital
Kenyatta University Teaching Refferal and Research Hospital
Kenyatta University Teaching Refferal and Research Hospital
Kenyatta University Teaching Refferal and Research Hospital
Kenyatta University Teaching Refferal and Research Hospital
Kenyatta University Teaching Refferal and Research Hospital
Kenyatta University Teaching Refferal and Research Hospital
Kenyatta University Teaching Refferal and Research Hospital
Kenyatta University Teaching Refferal and Research Hospital
Kenyatta University Teaching Refferal and Research Hospital
Kenyatta University Teaching Refferal and Research Hospital
Kenyatta University Teaching Refferal and Research Hospital
Kenyatta University Teaching Refferal and Research Hospital
Kenyatta University Teaching Refferal and Research Hospital
Kenyatta University Teaching Refferal and Research Hospital
Kenyatta University Teaching Refferal and Research Hospital
Kenyatta University Teaching Refferal and Research Hospital
Kenyatta University Teaching Refferal and Research Hospital
Kenyatta University Teaching Refferal and Research Hospital
Kenyatta University Teaching Refferal and Research Hospital
Kenyatta University Teaching Refferal and Research Hospital
Kenyatta University Teaching Refferal and Research Hospital
Kenyatta University Teaching Refferal and Research Hospital
Kenyatta University Teaching Refferal and Research Hospital
Kenyatta University Teaching Refferal and Research Hospital
Kenyatta University Teaching Refferal and Research Hospital
Kenyatta University Teaching Refferal and Research Hospital
Kenyatta University Teaching Refferal and Research Hospital
Kenyatta University Teaching Refferal and Research Hospital
Kenyatta University Teaching Refferal and Research Hospital
Kenyatta University Teaching Refferal and Research Hospital
Kenyatta University Teaching Refferal and Research Hospital

Kenyatta University Teaching Refferal and Research Hospital
Kenyatta University Teaching Refferal and Research Hospital
Kenyatta University Teaching Refferal and Research Hospital
Kenyatta University Teaching Refferal and Research Hospital
Kenyatta University Teaching Refferal and Research Hospital
Kenyatta University Teaching Refferal and Research Hospital
Kenyatta University Teaching Refferal and Research Hospital
Kenyatta University Teaching Refferal and Research Hospital
Kenyatta University Teaching Refferal and Research Hospital
Kenyatta University Teaching Refferal and Research Hospital
Kenyatta University Teaching Refferal and Research Hospital
Kenyatta University Teaching Refferal and Research Hospital
Kenyatta University Teaching Refferal and Research Hospital
Kenyatta University Teaching Refferal and Research Hospital
Kenyatta University Teaching Refferal and Research Hospital
Kenyatta University Teaching Refferal and Research Hospital
Kenyatta University Teaching Refferal and Research Hospital
Kenyatta University Teaching Refferal and Research Hospital
Kenyatta University Teaching Refferal and Research Hospital
Kenyatta University Teaching Refferal and Research Hospital
Kenyatta University Teaching Refferal and Research Hospital
Kenyatta University Teaching Refferal and Research Hospital
Kenyatta University Teaching Refferal and Research Hospital
Kenyatta University Teaching Refferal and Research Hospital
Kenyatta University Teaching Refferal and Research Hospital
Kenyatta University Teaching Refferal and Research Hospital
Kenyatta University Teaching Refferal and Research Hospital
Kenyatta University Teaching Refferal and Research Hospital
Kenyatta University Teaching Refferal and Research Hospital
Kenyatta University Teaching Refferal and Research Hospital
Kenyatta University Teaching Refferal and Research Hospital
Kenyatta University Teaching Refferal and Research Hospital
Kenyatta University Teaching Refferal and Research Hospital
Mombasa Cancer Centre
Mombasa Cancer Centre
Mombasa Cancer Centre
Mombasa Cancer Centre
Mombasa Cancer Centre
Mombasa Cancer Centre
Mombasa Cancer Centre
Mombasa Cancer Centre
Mombasa Cancer Centre
Mombasa Cancer Centre
Mombasa Cancer Centre
Mombasa Cancer Centre
Mombasa Cancer Centre
Mombasa Cancer Centre
Mombasa Cancer Centre
Mombasa Cancer Centre
Mombasa Cancer Centre

Mombasa Cancer Centre
Mombasa Cancer Centre
Mombasa Cancer Centre
Mombasa Cancer Centre
Mombasa Cancer Centre
Mombasa Cancer Centre
Mombasa Cancer Centre
Mombasa Cancer Centre
Mombasa Cancer Centre
Mombasa Cancer Centre
Mombasa Cancer Centre
Mombasa Cancer Centre
Mombasa Cancer Centre
Mombasa Cancer Centre
Mombasa Cancer Centre
Mombasa Cancer Centre
Mombasa Cancer Centre
Mombasa Cancer Centre
Mombasa Cancer Centre
Mombasa Cancer Centre
Mombasa Cancer Centre
Mombasa Cancer Centre
Mombasa Cancer Centre
Mombasa Cancer Centre
Mombasa Cancer Centre
Mombasa Cancer Centre
Mombasa Cancer Centre
Mombasa Cancer Centre
Mombasa Cancer Centre
Mombasa Cancer Centre
Mombasa Cancer Centre
Mombasa Cancer Centre
Mombasa Cancer Centre
Mombasa Cancer Centre
Mombasa Cancer Centre
Mombasa Cancer Centre
Mombasa Cancer Centre
Mombasa Cancer Centre
Mombasa Cancer Centre
Mombasa Cancer Centre
Mombasa Cancer Centre
Mombasa Cancer Centre
Mombasa Cancer Centre
Mombasa Cancer Centre
Mombasa Cancer Centre
Mombasa Cancer Centre
Mombasa Cancer Centre
Mombasa Cancer Centre
Mombasa Cancer Centre
Mombasa Cancer Centre

Mombasa Cancer Centre
Mombasa Cancer Centre
Mombasa Cancer Centre
Mombasa Cancer Centre
Mombasa Cancer Centre
Mombasa Cancer Centre
Mombasa Cancer Centre
Mombasa Cancer Centre
Mombasa Cancer Centre
Mombasa Cancer Centre
Mombasa Cancer Centre
Mombasa Cancer Centre
Mombasa Cancer Centre
Mombasa Cancer Centre
Mombasa Cancer Centre
Mombasa Cancer Centre
Mombasa Cancer Centre
Mombasa Cancer Centre
Mombasa Cancer Centre
Mombasa Cancer Centre
Mombasa Cancer Centre
Mombasa Cancer Centre
Mombasa Cancer Centre
Mombasa Cancer Centre
Mombasa Cancer Centre
Mombasa Cancer Centre
Mombasa Cancer Centre
Mombasa Cancer Centre
Mombasa Cancer Centre
Mombasa Cancer Centre
Mombasa Cancer Centre
Mombasa Cancer Centre
Mombasa Cancer Centre
Mombasa Cancer Centre
Mombasa Cancer Centre
Mombasa Cancer Centre
Mombasa Cancer Centre
Mombasa Cancer Centre
Mombasa Cancer Centre
Mombasa Cancer Centre
Mombasa Cancer Centre
Mombasa Cancer Centre
Mombasa Cancer Centre
Mombasa Cancer Centre
Mombasa Cancer Centre
Mombasa Cancer Centre
Mombasa Cancer Centre
Mombasa Cancer Centre
Mombasa Cancer Centre
Mombasa Cancer Centre

Mombasa Cancer Centre
Mombasa Cancer Centre
Mombasa Cancer Centre
Mombasa Cancer Centre
Mombasa Cancer Centre
Mombasa Cancer Centre
Mombasa Cancer Centre
Mombasa Cancer Centre
Mombasa Cancer Centre
Mombasa Cancer Centre
Mombasa Cancer Centre
Mombasa Cancer Centre
Mombasa Cancer Centre
Mombasa Cancer Centre
Mombasa Cancer Centre
Mombasa Cancer Centre
Mombasa Cancer Centre
Mombasa Cancer Centre
Mombasa Cancer Centre
Mombasa Cancer Centre
Mombasa Cancer Centre
Kenyatta National Hospital
Kenyatta National Hospital
Kenyatta National Hospital
Kenyatta National Hospital
Kenyatta National Hospital
Kenyatta National Hospital
Kenyatta National Hospital
Kenyatta National Hospital
Kenyatta National Hospital
Kenyatta National Hospital
Kenyatta National Hospital
Kenyatta National Hospital
Kenyatta National Hospital
Kenyatta National Hospital
Kenyatta National Hospital
Kenyatta National Hospital
Kenyatta National Hospital
Kenyatta National Hospital
Kenyatta National Hospital
Kenyatta National Hospital
Kenyatta National Hospital
Kenyatta National Hospital
Kenyatta National Hospital
Kenyatta National Hospital
Kenyatta National Hospital
Kenyatta National Hospital
Kenyatta National Hospital
Kenyatta National Hospital
Kenyatta National Hospital

Kenyatta National Hospital
Kenyatta National Hospital
Kenyatta National Hospital
Kenyatta National Hospital
Kenyatta National Hospital
Kenyatta National Hospital
Kenyatta National Hospital
Kenyatta National Hospital
Kenyatta National Hospital
Kenyatta National Hospital
Kenyatta National Hospital
Kenyatta National Hospital
Kenyatta National Hospital
Kenyatta National Hospital
Kenyatta National Hospital
Kenyatta National Hospital
Kenyatta National Hospital
Kenyatta National Hospital
Kenyatta National Hospital
Kenyatta National Hospital
Kenyatta National Hospital
Kenyatta National Hospital
Kenyatta National Hospital
Kenyatta National Hospital
Kenyatta National Hospital
Kenyatta National Hospital
Kenyatta National Hospital
Kenyatta National Hospital
Kenyatta National Hospital
Kenyatta National Hospital
Kenyatta National Hospital
Kenyatta National Hospital
Kenyatta National Hospital
Kenyatta National Hospital
Kenyatta National Hospital
Kenyatta National Hospital
Kenyatta National Hospital
Kenyatta National Hospital
Kenyatta National Hospital
Kenyatta National Hospital
Kenyatta National Hospital
Kenyatta National Hospital
Kenyatta National Hospital
Kenyatta National Hospital
Kenyatta National Hospital
Kenyatta National Hospital
Kenyatta National Hospital
Kenyatta National Hospital
Kenyatta National Hospital
Kenyatta National Hospital

Kenyatta National Hospital
Kenyatta National Hospital
Kenyatta National Hospital
Kenyatta National Hospital
Kenyatta National Hospital
Kenyatta National Hospital
Kenyatta National Hospital
Kenyatta National Hospital
Kenyatta National Hospital
Kenyatta National Hospital
Kenyatta National Hospital
Kenyatta National Hospital
Kenyatta National Hospital
Kenyatta National Hospital
Kenyatta National Hospital
Kenyatta National Hospital
Kenyatta National Hospital
Kenyatta National Hospital
Kenyatta National Hospital
Kenyatta National Hospital
Kenyatta National Hospital
Kenyatta National Hospital
Kenyatta National Hospital
Kenyatta National Hospital
Kenyatta National Hospital
Kenyatta National Hospital
Kenyatta National Hospital
Kenyatta National Hospital
Kenyatta National Hospital
Kenyatta National Hospital
Kenyatta National Hospital
Kenyatta National Hospital
Kenyatta National Hospital
Kenyatta National Hospital
Kenyatta National Hospital
Kenyatta National Hospital
Kenyatta National Hospital
Kenyatta National Hospital
Kenyatta National Hospital
Kenyatta National Hospital
Kenyatta National Hospital
Kenyatta National Hospital
Kenyatta National Hospital
Kenyatta National Hospital
Kenyatta National Hospital
Kenyatta National Hospital
Kenyatta National Hospital
Kenyatta National Hospital
Kenyatta National Hospital
Kenyatta National Hospital

Kenyatta National Hospital
Kenyatta National Hospital
Kenyatta National Hospital
Kenyatta National Hospital
Kenyatta National Hospital
Kenyatta National Hospital
Kenyatta National Hospital
Kenyatta National Hospital
Kenyatta National Hospital
Kenyatta National Hospital
Kenyatta National Hospital
Kenyatta National Hospital
Kenyatta National Hospital
Kenyatta National Hospital
Kenyatta National Hospital
Kenyatta National Hospital
Kenyatta National Hospital
Kenyatta National Hospital
Kenyatta National Hospital
Kenyatta National Hospital
Kenyatta National Hospital
Kenyatta National Hospital
Kenyatta National Hospital
Kenyatta National Hospital
Kenyatta National Hospital
Kenyatta National Hospital
Kenyatta National Hospital
Kenyatta National Hospital
Kenyatta National Hospital
Kenyatta National Hospital
Kenyatta National Hospital
Kenyatta National Hospital
Kenyatta National Hospital
Kenyatta National Hospital
Kenyatta National Hospital
Kenyatta National Hospital
Kenyatta National Hospital
Kenyatta National Hospital
Kenyatta National Hospital
Kenyatta National Hospital
Kenyatta National Hospital
Kenyatta National Hospital
Kenyatta National Hospital
Kenyatta National Hospital
Kenyatta National Hospital
Kenyatta National Hospital
Kenyatta National Hospital
Kenyatta National Hospital
Kenyatta National Hospital
Kenyatta National Hospital

Kenyatta National Hospital
Kenyatta National Hospital
Kenyatta National Hospital
Kenyatta National Hospital
Kenyatta National Hospital
Kenyatta National Hospital
Kenyatta National Hospital
Kenyatta National Hospital
Kenyatta National Hospital
Kenyatta National Hospital
Kenyatta National Hospital
Kenyatta National Hospital
Kenyatta National Hospital
Kenyatta National Hospital
Kenyatta National Hospital
Kenyatta National Hospital
Kenyatta National Hospital
Kenyatta National Hospital
Kenyatta National Hospital
Kenyatta National Hospital
Kenyatta National Hospital
Kenyatta National Hospital
Kenyatta National Hospital
Kenyatta National Hospital
Kenyatta National Hospital
Kenyatta National Hospital
Kenyatta National Hospital
Kenyatta National Hospital
Kenyatta National Hospital
Kenyatta National Hospital
Kenyatta National Hospital
Kenyatta National Hospital
Kenyatta National Hospital
Kenyatta National Hospital
Kenyatta National Hospital
Kenyatta National Hospital
Kenyatta National Hospital
Nyeri Cancer Centre
Kakamega Cancer Centre
Nakuru Cancer Centre
Kakamega Cancer Centre
Nakuru Cancer Centre
Nakuru Cancer Centre
Kakamega Cancer Centre
Nakuru Cancer Centre
Nyeri Cancer Centre
Nyeri Cancer Centre
Kakamega Cancer Centre
Garissa Cancer Centre
Machakos Cancer Centre

Meru Cancer Centre
Machakos Cancer Centre
Machakos Cancer Centre
Meru Cancer Centre
Nyeri Cancer Centre
Nyeri Cancer Centre
Mombasa Cancer Centre
Kakamega Cancer Centre
Mombasa Cancer Centre
Nyeri Cancer Centre
Nyeri Cancer Centre
Garissa Cancer Centre
Mombasa Cancer Centre
Kakamega Cancer Centre
Nakuru Cancer Centre
Kakamega Cancer Centre
Kakamega Cancer Centre
Kakamega Cancer Centre
Mombasa Cancer Centre
Nakuru Cancer Centre
Mombasa Cancer Centre
Mombasa Cancer Centre
Mombasa Cancer Centre
Mombasa Cancer Centre
Makueni Cancer Centre
Kakamega Cancer Centre
Kakamega Cancer Centre
Kakamega Cancer Centre
Nakuru Cancer Centre
Nakuru Cancer Centre
Nakuru Cancer Centre
Nakuru Cancer Centre
Nakuru Cancer Centre
Nakuru Cancer Centre
Mombasa Cancer Centre
Mombasa Cancer Centre
Mombasa Cancer Centre
Mombasa Cancer Centre
Mombasa Cancer Centre
Mombasa Cancer Centre
Mombasa Cancer Centre
Mombasa Cancer Centre
Mombasa Cancer Centre
Garissa Cancer Centre
Nyeri Cancer Centre
Garissa Cancer Centre
Kakamega Cancer Centre
Kakamega Cancer Centre
Nakuru Cancer Centre
Nakuru Cancer Centre

Nakuru Cancer Centre
Kakamega Cancer Centre
Nakuru Cancer Centre
Kakamega Cancer Centre
Nakuru Cancer Centre
Nakuru Cancer Centre
Nyeri Cancer Centre
Nyeri Cancer Centre
Nyeri Cancer Centre
Nakuru Cancer Centre
Nyeri Cancer Centre
Nakuru Cancer Centre
Machakos Cancer Centre
Nyeri Cancer Centre
Kakamega Cancer Centre
Kakamega Cancer Centre
Garissa Cancer Centre
Makueni Cancer Centre
Taita Taveta Cancer Centre
Kakamega Cancer Centre
Garissa Cancer Centre
Meru Cancer Centre
Kakamega Cancer Centre
Machakos Cancer Centre
Mombasa Cancer Centre
Mombasa Cancer Centre
Mombasa Cancer Centre
Mombasa Cancer Centre
Nyeri Cancer Centre
Mombasa Cancer Centre
Mombasa Cancer Centre
Nyeri Cancer Centre
Nakuru Cancer Centre
Nakuru Cancer Centre
Nakuru Cancer Centre
Nakuru Cancer Centre
Mombasa Cancer Centre
Kakamega Cancer Centre
Nyeri Cancer Centre
Nyeri Cancer Centre
Makueni Cancer Centre
Mombasa Cancer Centre
Mombasa Cancer Centre
Mombasa Cancer Centre
Mombasa Cancer Centre
Mombasa Cancer Centre
Mombasa Cancer Centre
Nyeri Cancer Centre
Mombasa Cancer Centre
Mombasa Cancer Centre

Nyeri Cancer Centre
Mombasa Cancer Centre
Mombasa Cancer Centre
Meru Cancer Centre
Meru Cancer Centre
Meru Cancer Centre
Garissa Cancer Centre
Garissa Cancer Centre
Nakuru Cancer Centre
Mombasa Cancer Centre
Mombasa Cancer Centre
Mombasa Cancer Centre
Mombasa Cancer Centre
Mombasa Cancer Centre
Mombasa Cancer Centre
Nakuru Cancer Centre
Nakuru Cancer Centre
Mombasa Cancer Centre
Mombasa Cancer Centre
Mombasa Cancer Centre
Kakamega Cancer Centre
Machakos Cancer Centre
Kakamega Cancer Centre
Kakamega Cancer Centre
Meru Cancer Centre
Kakamega Cancer Centre
Kakamega Cancer Centre
Kakamega Cancer Centre
Mombasa Cancer Centre
Machakos Cancer Centre
Kakamega Cancer Centre
Garissa Cancer Centre
Mombasa Cancer Centre
Nyeri Cancer Centre
Makueni Cancer Centre
Mombasa Cancer Centre
Mombasa Cancer Centre
Nyeri Cancer Centre
Makueni Cancer Centre
Mombasa Cancer Centre
Nyeri Cancer Centre
Nakuru Cancer Centre
Kakamega Cancer Centre
Nakuru Cancer Centre
Mombasa Cancer Centre
Taita Taveta Cancer Centre
Mombasa Cancer Centre
Mombasa Cancer Centre
Mombasa Cancer Centre
Mombasa Cancer Centre

Mombasa Cancer Centre
Mombasa Cancer Centre
Kakamega Cancer Centre
Mombasa Cancer Centre
Meru Cancer Centre
Meru Cancer Centre
Meru Cancer Centre
Makueni Cancer Centre
Garissa Cancer Centre
Kakamega Cancer Centre
Kakamega Cancer Centre
Meru Cancer Centre
Makueni Cancer Centre
Meru Cancer Centre
Mombasa Cancer Centre
Mombasa Cancer Centre
Mombasa Cancer Centre
Meru Cancer Centre
Garissa Cancer Centre
Meru Cancer Centre
Meru Cancer Centre
Meru Cancer Centre
Meru Cancer Centre
Kakamega Cancer Centre
Machakos Cancer Centre
Machakos Cancer Centre
Makueni Cancer Centre
Kakamega Cancer Centre
Kakamega Cancer Centre
Nakuru Cancer Centre
Nakuru Cancer Centre
Nakuru Cancer Centre
Mombasa Cancer Centre
Nakuru Cancer Centre
Mombasa Cancer Centre
Mombasa Cancer Centre
Mombasa Cancer Centre
Nakuru Cancer Centre
Mombasa Cancer Centre
Mombasa Cancer Centre
Mombasa Cancer Centre
Nakuru Cancer Centre
Mombasa Cancer Centre
Nyeri Cancer Centre
Nyeri Cancer Centre
Kakamega Cancer Centre
Mombasa Cancer Centre
Mombasa Cancer Centre
Mombasa Cancer Centre
Mombasa Cancer Centre

Mombasa Cancer Centre
Nyeri Cancer Centre
Mombasa Cancer Centre
Mombasa Cancer Centre
Mombasa Cancer Centre
Kakamega Cancer Centre
Mombasa Cancer Centre
Meru Cancer Centre
Garissa Cancer Centre
Kakamega Cancer Centre
Nakuru Cancer Centre
Machakos Cancer Centre
Taita Taveta Cancer Centre
Makueni Cancer Centre
Machakos Cancer Centre
Meru Cancer Centre
Meru Cancer Centre
Makueni Cancer Centre
Nyeri Cancer Centre
Kakamega Cancer Centre
Nakuru Cancer Centre
Nakuru Cancer Centre
Nyeri Cancer Centre
Mombasa Cancer Centre
Taita Taveta Cancer Centre
Nyeri Cancer Centre
Nyeri Cancer Centre
Nyeri Cancer Centre
Nakuru Cancer Centre
Nakuru Cancer Centre
Nakuru Cancer Centre
Nyeri Cancer Centre
Nakuru Cancer Centre
Kakamega Cancer Centre
Makueni Cancer Centre
Taita Taveta Cancer Centre
Garissa Cancer Centre
Meru Cancer Centre
Meru Cancer Centre
Meru Cancer Centre
Meru Cancer Centre
Kakamega Cancer Centre
Nakuru Cancer Centre
Machakos Cancer Centre
Nyeri Cancer Centre
Kakamega Cancer Centre
Kakamega Cancer Centre
Kakamega Cancer Centre
Bomet Cancer Centre
Kakamega Cancer Centre

Nyeri Cancer Centre
Kakamega Cancer Centre
Makueni Cancer Centre
Mombasa Cancer Centre
Nyeri Cancer Centre
Nakuru Cancer Centre
Nakuru Cancer Centre
Meru Cancer Centre
Nyeri Cancer Centre
Nyeri Cancer Centre
Meru Cancer Centre
Meru Cancer Centre
Meru Cancer Centre
Kakamega Cancer Centre
Nyeri Cancer Centre
Nyeri Cancer Centre
Meru Cancer Centre
Nyeri Cancer Centre
Nakuru Cancer Centre
Nakuru Cancer Centre
Nyeri Cancer Centre
Kakamega Cancer Centre
Machakos Cancer Centre
Garissa Cancer Centre
Taita Taveta Cancer Centre
Makueni Cancer Centre
Machakos Cancer Centre
Machakos Cancer Centre
Machakos Cancer Centre
Meru Cancer Centre
Nyeri Cancer Centre
Nakuru Cancer Centre
Bomet Cancer Centre
Makueni Cancer Centre
Makueni Cancer Centre
Bomet Cancer Centre
Nyeri Cancer Centre
Nyeri Cancer Centre
Nyeri Cancer Centre
Makueni Cancer Centre
Nyeri Cancer Centre
Nyeri Cancer Centre
Garissa Cancer Centre
Nakuru Cancer Centre
Kakamega Cancer Centre
Makueni Cancer Centre
Makueni Cancer Centre
Nyeri Cancer Centre
Nakuru Cancer Centre
Kakamega Cancer Centre

Nakuru Cancer Centre
Makueni Cancer Centre
Nyeri Cancer Centre
Nyeri Cancer Centre
Mombasa Cancer Centre
Makueni Cancer Centre
Makueni Cancer Centre
Makueni Cancer Centre
Mombasa Cancer Centre
Nyeri Cancer Centre
Nyeri Cancer Centre
Nyeri Cancer Centre
Meru Cancer Centre
Taita Taveta Cancer Centre
Kakamega Cancer Centre
Bomet Cancer Centre
Machakos Cancer Centre
Nakuru Cancer Centre
Makueni Cancer Centre
Kisumu Cancer Centre
Kakamega Cancer Centre
Kakamega Cancer Centre
Kakamega Cancer Centre
Meru Cancer Centre
Machakos Cancer Centre
Mombasa Cancer Centre
Machakos Cancer Centre
Nakuru Cancer Centre
Mombasa Cancer Centre
Mombasa Cancer Centre
Makueni Cancer Centre
Kakamega Cancer Centre
Meru Cancer Centre
Makueni Cancer Centre
Meru Cancer Centre
Nyeri Cancer Centre
Nyeri Cancer Centre
Machakos Cancer Centre
Machakos Cancer Centre
Nakuru Cancer Centre
Bomet Cancer Centre
Kakamega Cancer Centre
Mombasa Cancer Centre
Kakamega Cancer Centre
Nakuru Cancer Centre
Kenyatta University Teaching Refferal and Research Hospital
Nakuru Cancer Centre
Nakuru Cancer Centre
Meru Cancer Centre
Meru Cancer Centre

Mombasa Cancer Centre
Makueni Cancer Centre
Mombasa Cancer Centre
Nakuru Cancer Centre
Nakuru Cancer Centre
Nakuru Cancer Centre
Meru Cancer Centre
Meru Cancer Centre
Kenyatta University Teaching Refferal and Research Hospital
Kenyatta University Teaching Refferal and Research Hospital
Mombasa Cancer Centre
Mombasa Cancer Centre
Mombasa Cancer Centre
Kenyatta University Teaching Refferal and Research Hospital
Kenyatta University Teaching Refferal and Research Hospital
Mombasa Cancer Centre
Mombasa Cancer Centre
Kenyatta National Hospital
Nakuru Cancer Centre
Mombasa Cancer Centre
Mombasa Cancer Centre
Nakuru Cancer Centre
Mombasa Cancer Centre
Mombasa Cancer Centre
Mombasa Cancer Centre
Kenyatta University Teaching Refferal and Research Hospital
Kenyatta University Teaching Refferal and Research Hospital
Kenyatta University Teaching Refferal and Research Hospital
Mombasa Cancer Centre
Mombasa Cancer Centre
Meru Cancer Centre
Mombasa Cancer Centre
Mombasa Cancer Centre
Nakuru Cancer Centre
Meru Cancer Centre
Embu Cancer Centre
Kenyatta National Hospital
Mombasa Cancer Centre
Machakos Cancer Centre
Nakuru Cancer Centre
Kenyatta University Teaching Refferal and Research Hospital
Kenyatta University Teaching Refferal and Research Hospital
Nakuru Cancer Centre
Kenyatta University Teaching Refferal and Research Hospital
Kenyatta University Teaching Refferal and Research Hospital
Meru Cancer Centre
Mombasa Cancer Centre
Meru Cancer Centre
Mombasa Cancer Centre
Nakuru Cancer Centre

Mombasa Cancer Centre
Mombasa Cancer Centre
Nakuru Cancer Centre
Nakuru Cancer Centre
Meru Cancer Centre
Mombasa Cancer Centre
Mombasa Cancer Centre
Kenyatta University Teaching Refferal and Research Hospital
Kenyatta University Teaching Refferal and Research Hospital
Mombasa Cancer Centre
Mombasa Cancer Centre
Kenyatta University Teaching Refferal and Research Hospital
Kenyatta University Teaching Refferal and Research Hospital
Machakos Cancer Centre
Mombasa Cancer Centre
Mombasa Cancer Centre
Kakamega Cancer Centre
Nakuru Cancer Centre
Embu Cancer Centre
Kakamega Cancer Centre
Kenyatta University Teaching Refferal and Research Hospital
Nakuru Cancer Centre
Kenyatta University Teaching Refferal and Research Hospital
Mombasa Cancer Centre
Machakos Cancer Centre
Machakos Cancer Centre
Meru Cancer Centre
Mombasa Cancer Centre
Kenyatta University Teaching Refferal and Research Hospital
Mombasa Cancer Centre
Mombasa Cancer Centre
Kenyatta University Teaching Refferal and Research Hospital
Meru Cancer Centre
Kenyatta University Teaching Refferal and Research Hospital
Kenyatta University Teaching Refferal and Research Hospital
Mombasa Cancer Centre
Mombasa Cancer Centre
Meru Cancer Centre
Meru Cancer Centre
Kenyatta University Teaching Refferal and Research Hospital
Kenyatta University Teaching Refferal and Research Hospital
Mombasa Cancer Centre
Mombasa Cancer Centre
Kenyatta National Hospital
Meru Cancer Centre
Kenyatta National Hospital
Nyeri Cancer Centre
Machakos Cancer Centre
Nakuru Cancer Centre
Nakuru Cancer Centre

Embu Cancer Centre
Kisumu Cancer Centre
Kenyatta University Teaching Refferal and Research Hospital
Kenyatta University Teaching Refferal and Research Hospital
Kisumu Cancer Centre
Nakuru Cancer Centre
Kenyatta University Teaching Refferal and Research Hospital
Kenyatta University Teaching Refferal and Research Hospital
Kakamega Cancer Centre
Kakamega Cancer Centre
Meru Cancer Centre
Mombasa Cancer Centre
Mombasa Cancer Centre
Meru Cancer Centre
Kenyatta University Teaching Refferal and Research Hospital
Kenyatta University Teaching Refferal and Research Hospital
Kakamega Cancer Centre
Kakamega Cancer Centre
Machakos Cancer Centre
Kenyatta National Hospital
Nyeri Cancer Centre
Nakuru Cancer Centre
Mombasa Cancer Centre
Mombasa Cancer Centre
Meru Cancer Centre
Kenyatta National Hospital
Kisumu Cancer Centre
Nakuru Cancer Centre
Nakuru Cancer Centre
Garissa Cancer Centre
Kakamega Cancer Centre
Nyeri Cancer Centre
Machakos Cancer Centre
Nyeri Cancer Centre
Nakuru Cancer Centre
Nakuru Cancer Centre
Kenyatta National Hospital
Embu Cancer Centre
Nakuru Cancer Centre
Kisumu Cancer Centre
Meru Cancer Centre
Meru Cancer Centre
Mombasa Cancer Centre
Mombasa Cancer Centre
Kakamega Cancer Centre
Kenyatta University Teaching Refferal and Research Hospital
Kenyatta University Teaching Refferal and Research Hospital
Kakamega Cancer Centre
Kisumu Cancer Centre
Meru Cancer Centre

Meru Cancer Centre
Mombasa Cancer Centre
Mombasa Cancer Centre
Kenyatta University Teaching Refferal and Research Hospital
Kenyatta National Hospital
Kenyatta University Teaching Refferal and Research Hospital
Kenyatta National Hospital
Kenyatta National Hospital
Kenyatta National Hospital
Mombasa Cancer Centre
Mombasa Cancer Centre
Nyeri Cancer Centre
Nakuru Cancer Centre
Nakuru Cancer Centre
Embu Cancer Centre
Machakos Cancer Centre
Kakamega Cancer Centre
Kenyatta University Teaching Refferal and Research Hospital
Embu Cancer Centre
Kenyatta University Teaching Refferal and Research Hospital
Nakuru Cancer Centre
Nakuru Cancer Centre
Kisumu Cancer Centre
Nyeri Cancer Centre
Meru Cancer Centre
Meru Cancer Centre
Machakos Cancer Centre
Mombasa Cancer Centre
Mombasa Cancer Centre
Nyeri Cancer Centre
Kenyatta University Teaching Refferal and Research Hospital
Mombasa Cancer Centre
Kenyatta National Hospital
Mombasa Cancer Centre
Kenyatta National Hospital
Meru Cancer Centre
Taita Taveta Cancer Centre
Kenyatta University Teaching Refferal and Research Hospital
Kenyatta University Teaching Refferal and Research Hospital
Mombasa Cancer Centre
Kenyatta University Teaching Refferal and Research Hospital
Mombasa Cancer Centre
Kenyatta National Hospital
Kenyatta National Hospital
Kisumu Cancer Centre
Kakamega Cancer Centre
Meru Cancer Centre
Meru Cancer Centre
Nakuru Cancer Centre
Nyeri Cancer Centre

Meru Cancer Centre
Kenyatta University Teaching Refferal and Research Hospital
Machakos Cancer Centre
Meru Cancer Centre
Kakamega Cancer Centre
Kenyatta University Teaching Refferal and Research Hospital
Kakamega Cancer Centre
Embu Cancer Centre
Kenyatta National Hospital
Kenyatta National Hospital
Kisumu Cancer Centre
Mombasa Cancer Centre
Mombasa Cancer Centre
Nyeri Cancer Centre
Kenyatta National Hospital
Kenyatta National Hospital
Machakos Cancer Centre
Machakos Cancer Centre
Nakuru Cancer Centre
Nakuru Cancer Centre
Kisumu Cancer Centre
Kenyatta University Teaching Refferal and Research Hospital
Kenyatta University Teaching Refferal and Research Hospital
Nakuru Cancer Centre
Nakuru Cancer Centre
Kenyatta University Teaching Refferal and Research Hospital
Kenyatta University Teaching Refferal and Research Hospital
Kakamega Cancer Centre
Makueni Cancer Centre
Mombasa Cancer Centre
Mombasa Cancer Centre
Kisumu Cancer Centre
Meru Cancer Centre
Meru Cancer Centre
Machakos Cancer Centre
Kenyatta University Teaching Refferal and Research Hospital
Kenyatta University Teaching Refferal and Research Hospital
Kenyatta National Hospital
Makueni Cancer Centre
Kakamega Cancer Centre
Machakos Cancer Centre
Kenyatta National Hospital
Machakos Cancer Centre
Nyeri Cancer Centre
Nyeri Cancer Centre
Nakuru Cancer Centre
Embu Cancer Centre
Nakuru Cancer Centre
Mombasa Cancer Centre
Mombasa Cancer Centre

Kenyatta National Hospital
Kenyatta National Hospital
Kisumu Cancer Centre
Makueni Cancer Centre
Nakuru Cancer Centre
Nakuru Cancer Centre
Nyeri Cancer Centre
Machakos Cancer Centre
Nyeri Cancer Centre
Nakuru Cancer Centre
Nakuru Cancer Centre
Machakos Cancer Centre
Makueni Cancer Centre
Kakamega Cancer Centre
Kenyatta National Hospital
Kenyatta National Hospital
Kisumu Cancer Centre
Embu Cancer Centre
Nakuru Cancer Centre
Nakuru Cancer Centre
Kisumu Cancer Centre
Meru Cancer Centre
Meru Cancer Centre
Makueni Cancer Centre
Kakamega Cancer Centre
Mombasa Cancer Centre
Kakamega Cancer Centre
Mombasa Cancer Centre
Kenyatta University Teaching Refferal and Research Hospital
Kenyatta University Teaching Refferal and Research Hospital
Kakamega Cancer Centre
Makueni Cancer Centre
Meru Cancer Centre
Makueni Cancer Centre
Kisumu Cancer Centre
Mombasa Cancer Centre
Mombasa Cancer Centre
Embu Cancer Centre
Kenyatta National Hospital
Machakos Cancer Centre
Kenyatta University Teaching Refferal and Research Hospital
Machakos Cancer Centre
Kenyatta National Hospital
Kenyatta University Teaching Refferal and Research Hospital
Mombasa Cancer Centre
Mombasa Cancer Centre
Kenyatta National Hospital
Kenyatta National Hospital
Nakuru Cancer Centre
Nakuru Cancer Centre

Nyeri Cancer Centre
Embu Cancer Centre
Kakamega Cancer Centre
Machakos Cancer Centre
Machakos Cancer Centre
Makueni Cancer Centre
Mombasa Cancer Centre
Mombasa Cancer Centre
Embu Cancer Centre
Nakuru Cancer Centre
Nakuru Cancer Centre
Nyeri Cancer Centre
Kenyatta National Hospital
Machakos Cancer Centre
Kenyatta University Teaching Refferal and Research Hospital
Meru Cancer Centre
Meru Cancer Centre
Kisumu Cancer Centre
Kenyatta National Hospital
Kenyatta University Teaching Refferal and Research Hospital
Kakamega Cancer Centre
Nyeri Cancer Centre
Nyeri Cancer Centre
Makueni Cancer Centre
Mombasa Cancer Centre
Mombasa Cancer Centre
Kenyatta National Hospital
Kenyatta University Teaching Refferal and Research Hospital
Kenyatta National Hospital
Embu Cancer Centre
Meru Cancer Centre
Kenyatta University Teaching Refferal and Research Hospital
Meru Cancer Centre
Kenyatta University Teaching Refferal and Research Hospital
Kenyatta University Teaching Refferal and Research Hospital
Mombasa Cancer Centre
Mombasa Cancer Centre
Kenyatta National Hospital
Kenyatta National Hospital
Kakamega Cancer Centre
Meru Cancer Centre
Meru Cancer Centre
Kisumu Cancer Centre
Embu Cancer Centre
Nyeri Cancer Centre
Nyeri Cancer Centre
Nakuru Cancer Centre
Nakuru Cancer Centre
Meru Cancer Centre
Meru Cancer Centre

Kenyatta University Teaching Refferal and Research Hospital
Machakos Cancer Centre
Kenyatta University Teaching Refferal and Research Hospital
Kakamega Cancer Centre
Kakamega Cancer Centre
Nyeri Cancer Centre
Nyeri Cancer Centre
Mombasa Cancer Centre
Embu Cancer Centre
Embu Cancer Centre
Mombasa Cancer Centre
Kenyatta National Hospital
Kisumu Cancer Centre
Kenyatta National Hospital
Nyeri Cancer Centre
Nyeri Cancer Centre
Kisumu Cancer Centre
Machakos Cancer Centre
Kenyatta National Hospital
Kenyatta National Hospital
Embu Cancer Centre
Embu Cancer Centre
Makueni Cancer Centre
Kakamega Cancer Centre
Machakos Cancer Centre
Nakuru Cancer Centre
Nakuru Cancer Centre
Kakamega Cancer Centre
Kenyatta University Teaching Refferal and Research Hospital
Kenyatta University Teaching Refferal and Research Hospital
Meru Cancer Centre
Kenyatta University Teaching Refferal and Research Hospital
Kisumu Cancer Centre
Kenyatta University Teaching Refferal and Research Hospital
Kakamega Cancer Centre
Makueni Cancer Centre
Mombasa Cancer Centre
Mombasa Cancer Centre
Nakuru Cancer Centre
Nakuru Cancer Centre
Machakos Cancer Centre
Machakos Cancer Centre
Meru Cancer Centre
Bomet Cancer Centre
Kenyatta National Hospital
Kenyatta National Hospital
Kenyatta University Teaching Refferal and Research Hospital
Kakamega Cancer Centre
Kakamega Cancer Centre
Machakos Cancer Centre

Machakos Cancer Centre
Nakuru Cancer Centre
Nakuru Cancer Centre
Nyeri Cancer Centre
Nyeri Cancer Centre
Nyeri Cancer Centre
Kisumu Cancer Centre
Nyeri Cancer Centre
Kenyatta National Hospital
Kenyatta National Hospital
Nakuru Cancer Centre
Nakuru Cancer Centre
Embu Cancer Centre
Makueni Cancer Centre
Kakamega Cancer Centre
Makueni Cancer Centre
Makueni Cancer Centre
Nyeri Cancer Centre
Nyeri Cancer Centre
Nakuru Cancer Centre
Nakuru Cancer Centre
Machakos Cancer Centre
Kenyatta National Hospital
Kenyatta National Hospital
Kisumu Cancer Centre
Embu Cancer Centre
Nakuru Cancer Centre
Nakuru Cancer Centre
Meru Cancer Centre
Meru Cancer Centre
Kisumu Cancer Centre
Embu Cancer Centre
Embu Cancer Centre
Kenyatta University Teaching Refferal and Research Hospital
Makueni Cancer Centre
Kakamega Cancer Centre
Machakos Cancer Centre
Machakos Cancer Centre
Meru Cancer Centre
Makueni Cancer Centre
Kakamega Cancer Centre
Meru Cancer Centre
Kisumu Cancer Centre
Mombasa Cancer Centre
Mombasa Cancer Centre
Embu Cancer Centre
Kenyatta University Teaching Refferal and Research Hospital
Kenyatta National Hospital
Kenyatta National Hospital
Machakos Cancer Centre

Machakos Cancer Centre
Kenyatta National Hospital
Meru Cancer Centre
Nakuru Cancer Centre
Nakuru Cancer Centre
Nyeri Cancer Centre
Nyeri Cancer Centre
Embu Cancer Centre
Embu Cancer Centre
Mombasa Cancer Centre
Mombasa Cancer Centre
Kenyatta National Hospital
Machakos Cancer Centre
Kakamega Cancer Centre
Kakamega Cancer Centre
Makueni Cancer Centre
Makueni Cancer Centre
Machakos Cancer Centre
Kenyatta National Hospital
Nakuru Cancer Centre
Nakuru Cancer Centre
Kenyatta National Hospital
Makueni Cancer Centre
Embu Cancer Centre
Mombasa Cancer Centre
Machakos Cancer Centre
Embu Cancer Centre
Meru Cancer Centre
Nyeri Cancer Centre
Nyeri Cancer Centre
Kisumu Cancer Centre
Meru Cancer Centre
Kakamega Cancer Centre
Nyeri Cancer Centre
Nyeri Cancer Centre
Meru Cancer Centre
Meru Cancer Centre
Kenyatta University Teaching Refferal and Research Hospital
Kenyatta University Teaching Refferal and Research Hospital
Mombasa Cancer Centre
Kenyatta National Hospital
Embu Cancer Centre
Kenyatta National Hospital
Mombasa Cancer Centre
Mombasa Cancer Centre
Kenyatta University Teaching Refferal and Research Hospital
Kenyatta University Teaching Refferal and Research Hospital
Bomet Cancer Centre
Kenyatta National Hospital
Kenyatta National Hospital

Meru Cancer Centre
Kisumu Cancer Centre
Meru Cancer Centre
Nakuru Cancer Centre
Nyeri Cancer Centre
Nakuru Cancer Centre
Nyeri Cancer Centre
Kisumu Cancer Centre
Kisumu Cancer Centre
Nyeri Cancer Centre
Nyeri Cancer Centre
Machakos Cancer Centre
Machakos Cancer Centre
Kenyatta National Hospital
Kenyatta National Hospital
Mombasa Cancer Centre
Embu Cancer Centre
Embu Cancer Centre
Meru Cancer Centre
Meru Cancer Centre
Nyeri Cancer Centre
Nyeri Cancer Centre
Kenyatta National Hospital
Machakos Cancer Centre
Machakos Cancer Centre
Kisumu Cancer Centre
Kenyatta National Hospital
Kisumu Cancer Centre
Embu Cancer Centre
Nakuru Cancer Centre
Nakuru Cancer Centre
Kakamega Cancer Centre
Kakamega Cancer Centre
Kenyatta University Teaching Refferal and Research Hospital
Kenyatta University Teaching Refferal and Research Hospital
Nakuru Cancer Centre
Nakuru Cancer Centre
Kenyatta University Teaching Refferal and Research Hospital
Kakamega Cancer Centre
Kenyatta University Teaching Refferal and Research Hospital
Kisumu Cancer Centre
Meru Cancer Centre
Meru Cancer Centre
Mombasa Cancer Centre
Kisumu Cancer Centre
Mombasa Cancer Centre
Machakos Cancer Centre
Machakos Cancer Centre
Kenyatta National Hospital
Kenyatta University Teaching Refferal and Research Hospital

Kenyatta University Teaching Refferal and Research Hospital
Kenyatta National Hospital
Kakamega Cancer Centre
Machakos Cancer Centre
Machakos Cancer Centre
Nakuru Cancer Centre
Nyeri Cancer Centre
Nyeri Cancer Centre
Nakuru Cancer Centre
Mombasa Cancer Centre
Mombasa Cancer Centre
Kisumu Cancer Centre
Kenyatta National Hospital
Nyeri Cancer Centre
Kenyatta National Hospital
Kisumu Cancer Centre
Kakamega Cancer Centre
Kakamega Cancer Centre
Nakuru Cancer Centre
Nakuru Cancer Centre
Embu Cancer Centre
Machakos Cancer Centre
Machakos Cancer Centre
Makueni Cancer Centre
Nyeri Cancer Centre
Nyeri Cancer Centre
Nakuru Cancer Centre
Nakuru Cancer Centre
Machakos Cancer Centre
Kisumu Cancer Centre
Kakamega Cancer Centre
Embu Cancer Centre
Kisumu Cancer Centre
Kenyatta National Hospital
Kakamega Cancer Centre
Kenyatta National Hospital
Nakuru Cancer Centre
Nakuru Cancer Centre
Meru Cancer Centre
Meru Cancer Centre
Embu Cancer Centre
Embu Cancer Centre
Kisumu Cancer Centre
Kisumu Cancer Centre
Makueni Cancer Centre
Mombasa Cancer Centre
Mombasa Cancer Centre
Kakamega Cancer Centre
Kenyatta University Teaching Refferal and Research Hospital
Kakamega Cancer Centre

Kenyatta University Teaching Refferal and Research Hospital
Machakos Cancer Centre
Machakos Cancer Centre
Meru Cancer Centre
Kakamega Cancer Centre
Kisumu Cancer Centre
Meru Cancer Centre
Kisumu Cancer Centre
Kakamega Cancer Centre
Mombasa Cancer Centre
Mombasa Cancer Centre
Embu Cancer Centre
Embu Cancer Centre
Kenyatta National Hospital
Machakos Cancer Centre
Kenyatta National Hospital
Machakos Cancer Centre
Kenyatta University Teaching Refferal and Research Hospital
Kenyatta University Teaching Refferal and Research Hospital
Mombasa Cancer Centre
Mombasa Cancer Centre
Kenyatta National Hospital
Kenyatta National Hospital
Nakuru Cancer Centre
Nakuru Cancer Centre
Nyeri Cancer Centre
Nyeri Cancer Centre
Embu Cancer Centre
Makueni Cancer Centre
Kakamega Cancer Centre
Kakamega Cancer Centre
Machakos Cancer Centre
Nakuru Cancer Centre
Kakamega Cancer Centre
Mombasa Cancer Centre
Mombasa Cancer Centre
Kakamega Cancer Centre
Embu Cancer Centre
Nakuru Cancer Centre
Makueni Cancer Centre
Nyeri Cancer Centre
Kisumu Cancer Centre
Kenyatta University Teaching Refferal and Research Hospital
Nyeri Cancer Centre
Kenyatta University Teaching Refferal and Research Hospital
Meru Cancer Centre
Meru Cancer Centre
Machakos Cancer Centre
Kenyatta National Hospital
Kenyatta National Hospital

Kakamega Cancer Centre
Kakamega Cancer Centre
Makueni Cancer Centre
Nyeri Cancer Centre
Nyeri Cancer Centre
Kenyatta National Hospital
Kenyatta University Teaching Refferal and Research Hospital
Mombasa Cancer Centre
Kenyatta National Hospital
Meru Cancer Centre
Kenyatta University Teaching Refferal and Research Hospital
Embu Cancer Centre
Embu Cancer Centre
Mombasa Cancer Centre
Meru Cancer Centre
Kenyatta University Teaching Refferal and Research Hospital
Kenyatta University Teaching Refferal and Research Hospital
Mombasa Cancer Centre
Mombasa Cancer Centre
Kenyatta National Hospital
Kenyatta National Hospital
Embu Cancer Centre
Kakamega Cancer Centre
Kisumu Cancer Centre
Meru Cancer Centre
Meru Cancer Centre
Kisumu Cancer Centre
Nyeri Cancer Centre
Nyeri Cancer Centre
Nakuru Cancer Centre
Nakuru Cancer Centre
Kisumu Cancer Centre
Machakos Cancer Centre
Kenyatta University Teaching Refferal and Research Hospital
Kakamega Cancer Centre
Nyeri Cancer Centre
Kakamega Cancer Centre
Nyeri Cancer Centre
Machakos Cancer Centre
Meru Cancer Centre
Mombasa Cancer Centre
Mombasa Cancer Centre
Embu Cancer Centre
Embu Cancer Centre
Kenyatta University Teaching Refferal and Research Hospital
Meru Cancer Centre
Kenyatta National Hospital
Kenyatta National Hospital
Nyeri Cancer Centre
Nyeri Cancer Centre

Machakos Cancer Centre
Machakos Cancer Centre
Kisumu Cancer Centre
Kenyatta National Hospital
Kenyatta National Hospital
Embu Cancer Centre
Embu Cancer Centre
Nakuru Cancer Centre
Nakuru Cancer Centre
Kakamega Cancer Centre
Kakamega Cancer Centre
Kenyatta University Teaching Refferal and Research Hospital
Kenyatta University Teaching Refferal and Research Hospital
Kakamega Cancer Centre
Kakamega Cancer Centre
Kenyatta University Teaching Refferal and Research Hospital
Kenyatta University Teaching Refferal and Research Hospital
Makueni Cancer Centre
Nakuru Cancer Centre
Meru Cancer Centre
Kisumu Cancer Centre
Mombasa Cancer Centre
Mombasa Cancer Centre
Machakos Cancer Centre
Nakuru Cancer Centre
Machakos Cancer Centre
Meru Cancer Centre
Kenyatta National Hospital
Kenyatta National Hospital
Kenyatta University Teaching Refferal and Research Hospital
Kenyatta University Teaching Refferal and Research Hospital
Kakamega Cancer Centre
Machakos Cancer Centre
Machakos Cancer Centre
Nakuru Cancer Centre
Nakuru Cancer Centre
Nyeri Cancer Centre
Nyeri Cancer Centre
Taita Taveta Cancer Centre
Mombasa Cancer Centre
Kisumu Cancer Centre
Kisumu Cancer Centre
Nyeri Cancer Centre
Nyeri Cancer Centre
Kenyatta National Hospital
Kenyatta National Hospital
Kakamega Cancer Centre
Kakamega Cancer Centre
Nakuru Cancer Centre
Nakuru Cancer Centre

Makueni Cancer Centre
Embu Cancer Centre
Embu Cancer Centre
Makueni Cancer Centre
Makueni Cancer Centre
Nyeri Cancer Centre
Nyeri Cancer Centre
Machakos Cancer Centre
Nakuru Cancer Centre
Nakuru Cancer Centre
Machakos Cancer Centre
Kisumu Cancer Centre
Kisumu Cancer Centre
Kenyatta National Hospital
Embu Cancer Centre
Embu Cancer Centre
Kakamega Cancer Centre
Kakamega Cancer Centre
Kenyatta National Hospital
Nakuru Cancer Centre
Nakuru Cancer Centre
Meru Cancer Centre
Meru Cancer Centre
Garissa Cancer Centre
Bomet Cancer Centre
Embu Cancer Centre
Embu Cancer Centre
Kisumu Cancer Centre
Kenyatta University Teaching Refferal and Research Hospital
Kenyatta University Teaching Refferal and Research Hospital
Mombasa Cancer Centre
Mombasa Cancer Centre
Makueni Cancer Centre
Kakamega Cancer Centre
Kakamega Cancer Centre
Machakos Cancer Centre
Machakos Cancer Centre
Kakamega Cancer Centre
Kisumu Cancer Centre
Meru Cancer Centre
Kakamega Cancer Centre
Meru Cancer Centre
Mombasa Cancer Centre
Mombasa Cancer Centre
Embu Cancer Centre
Embu Cancer Centre
Kenyatta University Teaching Refferal and Research Hospital
Kenyatta National Hospital
Machakos Cancer Centre
Machakos Cancer Centre

Kenyatta National Hospital
Kenyatta University Teaching Refferal and Research Hospital
Kenyatta National Hospital
Kenyatta National Hospital
Nakuru Cancer Centre
Nakuru Cancer Centre
Nyeri Cancer Centre
Nyeri Cancer Centre
Embu Cancer Centre
Embu Cancer Centre
Mombasa Cancer Centre
Mombasa Cancer Centre
Machakos Cancer Centre
Kakamega Cancer Centre
Kakamega Cancer Centre
Makueni Cancer Centre
Makueni Cancer Centre
Nakuru Cancer Centre
Nakuru Cancer Centre
Kenyatta National Hospital
Kenyatta National Hospital
Machakos Cancer Centre
Embu Cancer Centre
Mombasa Cancer Centre
Mombasa Cancer Centre
Embu Cancer Centre
Kenyatta University Teaching Refferal and Research Hospital
Kenyatta University Teaching Refferal and Research Hospital
Kakamega Cancer Centre
Meru Cancer Centre
Meru Cancer Centre
Nyeri Cancer Centre
Nyeri Cancer Centre
Kakamega Cancer Centre
Nyeri Cancer Centre
Nyeri Cancer Centre
Kakamega Cancer Centre
Kenyatta National Hospital
Kenyatta National Hospital
Meru Cancer Centre
Meru Cancer Centre
Bomet Cancer Centre
Kenyatta University Teaching Refferal and Research Hospital
Kenyatta University Teaching Refferal and Research Hospital
Embu Cancer Centre
Embu Cancer Centre
Taita Taveta Cancer Centre
Mombasa Cancer Centre
Mombasa Cancer Centre
Kenyatta University Teaching Refferal and Research Hospital

Mombasa Cancer Centre
Mombasa Cancer Centre
Kenyatta University Teaching Refferal and Research Hospital
Kenyatta National Hospital
Bomet Cancer Centre
Kenyatta National Hospital
Meru Cancer Centre
Meru Cancer Centre
Nakuru Cancer Centre
Nyeri Cancer Centre
Nakuru Cancer Centre
Nyeri Cancer Centre
Kisumu Cancer Centre
Kisumu Cancer Centre
Nyeri Cancer Centre
Nyeri Cancer Centre
Kakamega Cancer Centre
Machakos Cancer Centre
Machakos Cancer Centre
Mombasa Cancer Centre
Kenyatta National Hospital
Taita Taveta Cancer Centre
Embu Cancer Centre
Taita Taveta Cancer Centre
Kenyatta National Hospital
Mombasa Cancer Centre
Embu Cancer Centre
Kenyatta University Teaching Refferal and Research Hospital
Meru Cancer Centre
Meru Cancer Centre
Kenyatta University Teaching Refferal and Research Hospital
Nyeri Cancer Centre
Kenyatta National Hospital
Meru Cancer Centre
Kenyatta National Hospital
Meru Cancer Centre
Garissa Cancer Centre
Makueni Cancer Centre
Taita Taveta Cancer Centre
Makueni Cancer Centre
Kakamega Cancer Centre
Makueni Cancer Centre
Makueni Cancer Centre
Makueni Cancer Centre
Kenyatta University Teaching Refferal and Research Hospital
Kakamega Cancer Centre
Garissa Cancer Centre
Nakuru Cancer Centre
Makueni Cancer Centre
Makueni Cancer Centre

Makueni Cancer Centre
Machakos Cancer Centre
Mombasa Cancer Centre
Kenyatta University Teaching Refferal and Research Hospital
Makueni Cancer Centre
Makueni Cancer Centre
Makueni Cancer Centre
Kakamega Cancer Centre
Kakamega Cancer Centre
Makueni Cancer Centre
Makueni Cancer Centre
Kenyatta National Hospital
Taita Taveta Cancer Centre
Nakuru Cancer Centre
Nakuru Cancer Centre
Taita Taveta Cancer Centre
Mombasa Cancer Centre
Mombasa Cancer Centre
Meru Cancer Centre
Nyeri Cancer Centre
Kenyatta National Hospital
Kenyatta National Hospital
Meru Cancer Centre
Embu Cancer Centre
Nakuru Cancer Centre
Embu Cancer Centre
Kakamega Cancer Centre
Makueni Cancer Centre
Makueni Cancer Centre
Taita Taveta Cancer Centre
Kenyatta National Hospital
Machakos Cancer Centre
Machakos Cancer Centre
Machakos Cancer Centre
Makueni Cancer Centre
Nakuru Cancer Centre
Makueni Cancer Centre
Garissa Cancer Centre
Garissa Cancer Centre
Kenyatta National Hospital
Garissa Cancer Centre
Kenyatta National Hospital
Embu Cancer Centre
Kenyatta National Hospital
Kisumu Cancer Centre
Kisumu Cancer Centre
Machakos Cancer Centre
Nakuru Cancer Centre
Machakos Cancer Centre
Embu Cancer Centre

Makueni Cancer Centre
Mombasa Cancer Centre
Makueni Cancer Centre
Mombasa Cancer Centre
Kakamega Cancer Centre
Kakamega Cancer Centre
Makueni Cancer Centre
Meru Cancer Centre
Makueni Cancer Centre
Kakamega Cancer Centre
Mombasa Cancer Centre
Kisumu Cancer Centre
Kenyatta National Hospital
Garissa Cancer Centre
Mombasa Cancer Centre
Bomet Cancer Centre
Meru Cancer Centre
Garissa Cancer Centre
Nakuru Cancer Centre
Machakos Cancer Centre
Makueni Cancer Centre
Machakos Cancer Centre
Makueni Cancer Centre
Embu Cancer Centre
Kakamega Cancer Centre
Taita Taveta Cancer Centre
Makueni Cancer Centre
Makueni Cancer Centre
Kenyatta National Hospital
Mombasa Cancer Centre
Kenyatta National Hospital
Kenyatta National Hospital
Meru Cancer Centre
Makueni Cancer Centre
Kakamega Cancer Centre
Kakamega Cancer Centre
Bomet Cancer Centre
Bomet Cancer Centre
Bomet Cancer Centre
Mombasa Cancer Centre
Taita Taveta Cancer Centre
Kenyatta National Hospital
Makueni Cancer Centre
Meru Cancer Centre
Taita Taveta Cancer Centre
Mombasa Cancer Centre
Meru Cancer Centre
Makueni Cancer Centre
Garissa Cancer Centre
Kenyatta National Hospital

Mombasa Cancer Centre
Bomet Cancer Centre
Garissa Cancer Centre
Embu Cancer Centre
Taita Taveta Cancer Centre
Taita Taveta Cancer Centre
Makueni Cancer Centre
Kakamega Cancer Centre
Kisumu Cancer Centre
Meru Cancer Centre
Nakuru Cancer Centre
Kenyatta National Hospital
Meru Cancer Centre
Machakos Cancer Centre
Garissa Cancer Centre
Garissa Cancer Centre
Mombasa Cancer Centre
Bomet Cancer Centre
Kisumu Cancer Centre
Kisumu Cancer Centre
Bomet Cancer Centre
Kenyatta National Hospital
Meru Cancer Centre
Machakos Cancer Centre
Kenyatta National Hospital
Kenyatta National Hospital
Bomet Cancer Centre
Kenyatta National Hospital
Embu Cancer Centre
Kakamega Cancer Centre
Garissa Cancer Centre
Kisumu Cancer Centre
Kakamega Cancer Centre
Embu Cancer Centre
Embu Cancer Centre
Nakuru Cancer Centre
Embu Cancer Centre
Meru Cancer Centre
Kenyatta University Teaching Refferal and Research Hospital
Kisumu Cancer Centre
Meru Cancer Centre
Makueni Cancer Centre
Kisumu Cancer Centre
Kakamega Cancer Centre
Kakamega Cancer Centre
Kisumu Cancer Centre
Garissa Cancer Centre
Garissa Cancer Centre
Garissa Cancer Centre
Makueni Cancer Centre

Taita Taveta Cancer Centre
Bomet Cancer Centre
Garissa Cancer Centre
Bomet Cancer Centre
Mombasa Cancer Centre
Kisumu Cancer Centre
Garissa Cancer Centre
Kisumu Cancer Centre
Kenyatta National Hospital
Bomet Cancer Centre
Bomet Cancer Centre
Kenyatta National Hospital
Kenyatta University Teaching Refferal and Research Hospital
Makueni Cancer Centre
Makueni Cancer Centre
Makueni Cancer Centre
Kenyatta National Hospital
Bomet Cancer Centre
Bomet Cancer Centre
Bomet Cancer Centre
Kenyatta National Hospital
Bomet Cancer Centre
Nyeri Cancer Centre
Nyeri Cancer Centre
Embu Cancer Centre
Taita Taveta Cancer Centre
Embu Cancer Centre
Embu Cancer Centre
Embu Cancer Centre
Embu Cancer Centre
Mombasa Cancer Centre
Mombasa Cancer Centre
Mombasa Cancer Centre
Garissa Cancer Centre
Garissa Cancer Centre
Garissa Cancer Centre
Meru Cancer Centre
Kenyatta National Hospital
Kisumu Cancer Centre
Meru Cancer Centre
Meru Cancer Centre
Kisumu Cancer Centre
Kenyatta National Hospital
Makueni Cancer Centre
Makueni Cancer Centre
Kakamega Cancer Centre
Garissa Cancer Centre
Embu Cancer Centre
Embu Cancer Centre
Kakamega Cancer Centre

Garissa Cancer Centre
Makueni Cancer Centre
Kenyatta National Hospital
Kenyatta National Hospital
Machakos Cancer Centre
Taita Taveta Cancer Centre
Machakos Cancer Centre
Machakos Cancer Centre
Makueni Cancer Centre
Makueni Cancer Centre
Kenyatta National Hospital
Kenyatta National Hospital
Garissa Cancer Centre
Embu Cancer Centre
Kenyatta National Hospital
Embu Cancer Centre
Embu Cancer Centre
Kakamega Cancer Centre
Kakamega Cancer Centre
Nakuru Cancer Centre
Machakos Cancer Centre
Machakos Cancer Centre
Embu Cancer Centre
Garissa Cancer Centre
Bomet Cancer Centre
Bomet Cancer Centre
Kisumu Cancer Centre
Kisumu Cancer Centre
Meru Cancer Centre
Garissa Cancer Centre
Mombasa Cancer Centre
Kenyatta University Teaching Refferal and Research Hospital
Mombasa Cancer Centre
Kakamega Cancer Centre
Makueni Cancer Centre
Kakamega Cancer Centre
Kakamega Cancer Centre
Kisumu Cancer Centre
Meru Cancer Centre
Kakamega Cancer Centre
Makueni Cancer Centre
Kisumu Cancer Centre
Kisumu Cancer Centre
Embu Cancer Centre
Embu Cancer Centre
Bomet Cancer Centre
Kenyatta National Hospital
Kenyatta University Teaching Refferal and Research Hospital
Machakos Cancer Centre
Kenyatta National Hospital

Kenyatta National Hospital
Machakos Cancer Centre
Garissa Cancer Centre
Garissa Cancer Centre
Garissa Cancer Centre
Machakos Cancer Centre
Garissa Cancer Centre
Kenyatta National Hospital
Nyeri Cancer Centre
Taita Taveta Cancer Centre
Taita Taveta Cancer Centre
Embu Cancer Centre
Kenyatta National Hospital
Embu Cancer Centre
Nakuru Cancer Centre
Machakos Cancer Centre
Kenyatta National Hospital
Kenyatta National Hospital
Kakamega Cancer Centre
Kakamega Cancer Centre
Bomet Cancer Centre
Garissa Cancer Centre
Makueni Cancer Centre
Machakos Cancer Centre
Nakuru Cancer Centre
Taita Taveta Cancer Centre
Taita Taveta Cancer Centre
Embu Cancer Centre
Embu Cancer Centre
Embu Cancer Centre
Kenyatta National Hospital
Machakos Cancer Centre
Taita Taveta Cancer Centre
Kakamega Cancer Centre
Machakos Cancer Centre
Kenyatta National Hospital
Kenyatta National Hospital
Meru Cancer Centre
Kisumu Cancer Centre
Kisumu Cancer Centre
Kisumu Cancer Centre
Kenyatta National Hospital
Bomet Cancer Centre
Kenyatta University Teaching Refferal and Research Hospital
Kenyatta University Teaching Refferal and Research Hospital
Kakamega Cancer Centre
Kakamega Cancer Centre
Nyeri Cancer Centre
Kakamega Cancer Centre
Garissa Cancer Centre

Mombasa Cancer Centre
Makueni Cancer Centre
Kakamega Cancer Centre
Embu Cancer Centre
Embu Cancer Centre
Kenyatta National Hospital
Mombasa Cancer Centre
Bomet Cancer Centre
Garissa Cancer Centre
Bomet Cancer Centre
Kisumu Cancer Centre
Kenyatta National Hospital
Embu Cancer Centre
Garissa Cancer Centre
Kenyatta National Hospital
Kakamega Cancer Centre
Kakamega Cancer Centre
Kakamega Cancer Centre
Kakamega Cancer Centre
Kakamega Cancer Centre
Makueni Cancer Centre
Kisumu Cancer Centre
Kenyatta National Hospital
Machakos Cancer Centre
Makueni Cancer Centre
Makueni Cancer Centre
Makueni Cancer Centre
Kakamega Cancer Centre
Kakamega Cancer Centre
Kakamega Cancer Centre
Makueni Cancer Centre
Makueni Cancer Centre
Garissa Cancer Centre
Taita Taveta Cancer Centre
Mombasa Cancer Centre
Taita Taveta Cancer Centre
Mombasa Cancer Centre
Kisumu Cancer Centre
Mombasa Cancer Centre
Kenyatta National Hospital
Bomet Cancer Centre
Kenyatta University Teaching Refferal and Research Hospital
Kenyatta University Teaching Refferal and Research Hospital
Bomet Cancer Centre
Bomet Cancer Centre
Bomet Cancer Centre
Bomet Cancer Centre
Machakos Cancer Centre
Garissa Cancer Centre
Meru Cancer Centre

Bomet Cancer Centre
Taita Taveta Cancer Centre
Taita Taveta Cancer Centre
Taita Taveta Cancer Centre
Taita Taveta Cancer Centre
Garissa Cancer Centre
Garissa Cancer Centre
Garissa Cancer Centre
Garissa Cancer Centre
Garissa Cancer Centre
Garissa Cancer Centre
Garissa Cancer Centre
Makueni Cancer Centre
Nakuru Cancer Centre
Embu Cancer Centre
Kakamega Cancer Centre
Meru Cancer Centre
Kenyatta University Teaching Refferal and Research Hospital
Garissa Cancer Centre
Kisumu Cancer Centre
Taita Taveta Cancer Centre
Makueni Cancer Centre
Taita Taveta Cancer Centre
Taita Taveta Cancer Centre
Taita Taveta Cancer Centre
Garissa Cancer Centre
Kakamega Cancer Centre
Taita Taveta Cancer Centre
Garissa Cancer Centre
Bomet Cancer Centre
Bomet Cancer Centre
Mombasa Cancer Centre
Kisumu Cancer Centre
Kisumu Cancer Centre
Bomet Cancer Centre
Kenyatta National Hospital
Kenyatta University Teaching Refferal and Research Hospital
Bomet Cancer Centre
Kakamega Cancer Centre
Kakamega Cancer Centre
Makueni Cancer Centre
Kakamega Cancer Centre
Bomet Cancer Centre
Taita Taveta Cancer Centre
Taita Taveta Cancer Centre
Embu Cancer Centre
Embu Cancer Centre
Garissa Cancer Centre
Nakuru Cancer Centre
Embu Cancer Centre

Mombasa Cancer Centre
Garissa Cancer Centre
Garissa Cancer Centre
Bomet Cancer Centre
Nyeri Cancer Centre
Bomet Cancer Centre
Bomet Cancer Centre
Kisumu Cancer Centre
Kisumu Cancer Centre
Kisumu Cancer Centre
Kisumu Cancer Centre
Meru Cancer Centre
Meru Cancer Centre
Nakuru Cancer Centre
Nakuru Cancer Centre
Embu Cancer Centre
Nakuru Cancer Centre
Garissa Cancer Centre
Kakamega Cancer Centre
Embu Cancer Centre
Kakamega Cancer Centre
Garissa Cancer Centre
Kakamega Cancer Centre
Taita Taveta Cancer Centre
Makueni Cancer Centre
Garissa Cancer Centre
Kakamega Cancer Centre
Embu Cancer Centre
Embu Cancer Centre
Taita Taveta Cancer Centre
Taita Taveta Cancer Centre
Garissa Cancer Centre
Machakos Cancer Centre
Taita Taveta Cancer Centre
Taita Taveta Cancer Centre
Taita Taveta Cancer Centre
Taita Taveta Cancer Centre
Machakos Cancer Centre
Nakuru Cancer Centre
Embu Cancer Centre
Embu Cancer Centre
Kakamega Cancer Centre
Garissa Cancer Centre
Garissa Cancer Centre
Garissa Cancer Centre
Kakamega Cancer Centre
Embu Cancer Centre
Kenyatta National Hospital
Embu Cancer Centre
Garissa Cancer Centre

Embu Cancer Centre
Embu Cancer Centre
Nakuru Cancer Centre
Embu Cancer Centre
Garissa Cancer Centre
Garissa Cancer Centre
Kisumu Cancer Centre
Bomet Cancer Centre
Garissa Cancer Centre
Kisumu Cancer Centre
Meru Cancer Centre
Bomet Cancer Centre
Bomet Cancer Centre
Garissa Cancer Centre
Taita Taveta Cancer Centre
Taita Taveta Cancer Centre
Taita Taveta Cancer Centre
Garissa Cancer Centre
Garissa Cancer Centre
Makueni Cancer Centre
Makueni Cancer Centre
Kisumu Cancer Centre
Kisumu Cancer Centre
Meru Cancer Centre
Meru Cancer Centre
Kakamega Cancer Centre
Kisumu Cancer Centre
Makueni Cancer Centre
Makueni Cancer Centre
Garissa Cancer Centre
Garissa Cancer Centre
Garissa Cancer Centre
Garissa Cancer Centre
Taita Taveta Cancer Centre
Taita Taveta Cancer Centre
Embu Cancer Centre
Taita Taveta Cancer Centre
Taita Taveta Cancer Centre
Embu Cancer Centre
Embu Cancer Centre
Embu Cancer Centre
Bomet Cancer Centre
Bomet Cancer Centre
Kenyatta National Hospital
Kenyatta National Hospital
Machakos Cancer Centre
Kenyatta National Hospital
Bomet Cancer Centre
Meru Cancer Centre
Meru Cancer Centre

Bomet Cancer Centre
Garissa Cancer Centre
Meru Cancer Centre
Meru Cancer Centre
Bomet Cancer Centre
Garissa Cancer Centre
Meru Cancer Centre
Bomet Cancer Centre
Garissa Cancer Centre
Taita Taveta Cancer Centre
Taita Taveta Cancer Centre
Garissa Cancer Centre
Garissa Cancer Centre
Embu Cancer Centre
Taita Taveta Cancer Centre
Embu Cancer Centre
Makueni Cancer Centre
Kakamega Cancer Centre
Makueni Cancer Centre
Kakamega Cancer Centre
Garissa Cancer Centre
Garissa Cancer Centre
Garissa Cancer Centre
Garissa Cancer Centre
Kakamega Cancer Centre
Taita Taveta Cancer Centre
Makueni Cancer Centre
Makueni Cancer Centre
Kakamega Cancer Centre
Kisumu Cancer Centre
Nyeri Cancer Centre
Kisumu Cancer Centre
Meru Cancer Centre
Meru Cancer Centre
Kakamega Cancer Centre
Kisumu Cancer Centre
Bomet Cancer Centre
Kisumu Cancer Centre
Kenyatta National Hospital
Kenyatta University Teaching Refferal and Research Hospital
Bomet Cancer Centre
Garissa Cancer Centre
Garissa Cancer Centre
Bomet Cancer Centre
Bomet Cancer Centre
Embu Cancer Centre
Kakamega Cancer Centre
Makueni Cancer Centre
Makueni Cancer Centre
Makueni Cancer Centre

Makueni Cancer Centre
Nyeri Cancer Centre
Nyeri Cancer Centre
Kenyatta University Teaching Refferal and Research Hospital
Garissa Cancer Centre
Kenyatta National Hospital
Garissa Cancer Centre
Garissa Cancer Centre
Garissa Cancer Centre
Garissa Cancer Centre
Bomet Cancer Centre
Meru Cancer Centre
Bomet Cancer Centre
Taita Taveta Cancer Centre
Kakamega Cancer Centre
Kenyatta National Hospital
Taita Taveta Cancer Centre
Embu Cancer Centre
Taita Taveta Cancer Centre
Kenyatta National Hospital
Kenyatta University Teaching Refferal and Research Hospital
Bomet Cancer Centre
Bomet Cancer Centre
Bomet Cancer Centre
Garissa Cancer Centre
Embu Cancer Centre
Taita Taveta Cancer Centre
Taita Taveta Cancer Centre
Embu Cancer Centre
Taita Taveta Cancer Centre
Garissa Cancer Centre
Garissa Cancer Centre
Garissa Cancer Centre
Makueni Cancer Centre
Kakamega Cancer Centre
Kakamega Cancer Centre
Makueni Cancer Centre
Kakamega Cancer Centre
Kakamega Cancer Centre
Kenyatta National Hospital
Meru Cancer Centre
Kenyatta National Hospital
Kisumu Cancer Centre
Kisumu Cancer Centre
Kenyatta National Hospital
Nakuru Cancer Centre
Embu Cancer Centre
Kenyatta National Hospital
Kenyatta National Hospital
Makueni Cancer Centre

Kakamega Cancer Centre
Kakamega Cancer Centre
Makueni Cancer Centre
Makueni Cancer Centre
Garissa Cancer Centre
Garissa Cancer Centre
Taita Taveta Cancer Centre
Embu Cancer Centre
Taita Taveta Cancer Centre
Taita Taveta Cancer Centre
Kenyatta National Hospital
Kisumu Cancer Centre
Bomet Cancer Centre
Meru Cancer Centre
Meru Cancer Centre
Bomet Cancer Centre
Bomet Cancer Centre
Garissa Cancer Centre
Bomet Cancer Centre

Centre Type
Regional Cancer Centre
Regional Cancer Centre
Regional Cancer Centre
Regional Cancer Centre
Regional Cancer Centre
Regional Cancer Centre
Regional Cancer Centre
Regional Cancer Centre
Regional Cancer Centre
Regional Cancer Centre
Regional Cancer Centre
Regional Cancer Centre
Regional Cancer Centre
Regional Cancer Centre
Regional Cancer Centre
Regional Cancer Centre
Regional Cancer Centre
Regional Cancer Centre
National Cancer Centre
National Cancer Centre
Regional Cancer Centre
Regional Cancer Centre
Regional Cancer Centre
Regional Cancer Centre
Regional Cancer Centre
Regional Cancer Centre
Regional Cancer Centre
Regional Cancer Centre
Regional Cancer Centre
Regional Cancer Centre
Regional Cancer Centre
Regional Cancer Centre
Regional Cancer Centre
National Cancer Centre
National Cancer Centre
Regional Cancer Centre
Regional Cancer Centre
Regional Cancer Centre
Regional Cancer Centre
Regional Cancer Centre
Regional Cancer Centre
Regional Cancer Centre
Regional Cancer Centre
National Cancer Centre
Regional Cancer Centre
National Cancer Centre
Regional Cancer Centre
Regional Cancer Centre
Regional Cancer Centre

Regional Cancer Centre
Regional Cancer Centre
Regional Cancer Centre
Regional Cancer Centre
Regional Cancer Centre
Regional Cancer Centre
Regional Cancer Centre
Regional Cancer Centre
Regional Cancer Centre
Regional Cancer Centre
Regional Cancer Centre
National Cancer Centre
National Cancer Centre
Regional Cancer Centre
Regional Cancer Centre
Regional Cancer Centre
Regional Cancer Centre
Regional Cancer Centre
Regional Cancer Centre
Regional Cancer Centre
Regional Cancer Centre
Regional Cancer Centre
Regional Cancer Centre
Regional Cancer Centre
Regional Cancer Centre
Regional Cancer Centre
Regional Cancer Centre
Regional Cancer Centre
Regional Cancer Centre
Regional Cancer Centre
Regional Cancer Centre
Regional Cancer Centre
Regional Cancer Centre
Regional Cancer Centre
National Cancer Centre
National Cancer Centre
Regional Cancer Centre
Regional Cancer Centre
Regional Cancer Centre
Regional Cancer Centre
Regional Cancer Centre
Regional Cancer Centre
Regional Cancer Centre
Regional Cancer Centre
Regional Cancer Centre
Regional Cancer Centre
Regional Cancer Centre
Regional Cancer Centre
Regional Cancer Centre
Regional Cancer Centre

Regional Cancer Centre
Regional Cancer Centre
Regional Cancer Centre
Regional Cancer Centre
Regional Cancer Centre
Regional Cancer Centre
Regional Cancer Centre
Regional Cancer Centre
Regional Cancer Centre
Regional Cancer Centre
Regional Cancer Centre
Regional Cancer Centre
Regional Cancer Centre
Regional Cancer Centre
Regional Cancer Centre
Regional Cancer Centre
Regional Cancer Centre
Regional Cancer Centre
Regional Cancer Centre
Regional Cancer Centre
Regional Cancer Centre
Regional Cancer Centre
Regional Cancer Centre
Regional Cancer Centre
Regional Cancer Centre
Regional Cancer Centre
Regional Cancer Centre
National Cancer Centre
Regional Cancer Centre
National Cancer Centre
National Cancer Centre
Regional Cancer Centre
Regional Cancer Centre
Regional Cancer Centre
Regional Cancer Centre
National Cancer Centre
National Cancer Centre
Regional Cancer Centre
Regional Cancer Centre
Regional Cancer Centre
Regional Cancer Centre
Regional Cancer Centre
National Cancer Centre
National Cancer Centre
Regional Cancer Centre
Regional Cancer Centre
Regional Cancer Centre
Regional Cancer Centre
Regional Cancer Centre
Regional Cancer Centre

Regional Cancer Centre
Regional Cancer Centre
Regional Cancer Centre
Regional Cancer Centre
National Cancer Centre
National Cancer Centre
Regional Cancer Centre
Regional Cancer Centre
Regional Cancer Centre
Regional Cancer Centre
National Cancer Centre
National Cancer Centre
Regional Cancer Centre
Regional Cancer Centre
Regional Cancer Centre
Regional Cancer Centre
Regional Cancer Centre
Regional Cancer Centre
Regional Cancer Centre
Regional Cancer Centre
Regional Cancer Centre
Regional Cancer Centre
National Cancer Centre
Regional Cancer Centre
National Cancer Centre
National Cancer Centre
National Cancer Centre
Regional Cancer Centre
Regional Cancer Centre
Regional Cancer Centre
Regional Cancer Centre
Regional Cancer Centre
Regional Cancer Centre
National Cancer Centre
National Cancer Centre
Regional Cancer Centre
National Cancer Centre
Regional Cancer Centre
Regional Cancer Centre
Regional Cancer Centre
National Cancer Centre
Regional Cancer Centre
Regional Cancer Centre
Regional Cancer Centre
Regional Cancer Centre
National Cancer Centre
Regional Cancer Centre
Regional Cancer Centre
Regional Cancer Centre
Regional Cancer Centre

National Cancer Centre
Regional Cancer Centre
National Cancer Centre
Regional Cancer Centre
Regional Cancer Centre
Regional Cancer Centre
Regional Cancer Centre
National Cancer Centre
National Cancer Centre
Regional Cancer Centre
Regional Cancer Centre
Regional Cancer Centre
Regional Cancer Centre
Regional Cancer Centre
Regional Cancer Centre
Regional Cancer Centre
Regional Cancer Centre
National Cancer Centre
Regional Cancer Centre
Regional Cancer Centre
Regional Cancer Centre
Regional Cancer Centre
Regional Cancer Centre
Regional Cancer Centre
National Cancer Centre
Regional Cancer Centre
Regional Cancer Centre
Regional Cancer Centre
Regional Cancer Centre
Regional Cancer Centre
Regional Cancer Centre
Regional Cancer Centre
Regional Cancer Centre
Regional Cancer Centre
Regional Cancer Centre
Regional Cancer Centre
Regional Cancer Centre
National Cancer Centre
Regional Cancer Centre
Regional Cancer Centre
Regional Cancer Centre
Regional Cancer Centre
Regional Cancer Centre
Regional Cancer Centre
National Cancer Centre
National Cancer Centre
Regional Cancer Centre
Regional Cancer Centre
National Cancer Centre
National Cancer Centre

Regional Cancer Centre
National Cancer Centre
Regional Cancer Centre
Regional Cancer Centre
Regional Cancer Centre
Regional Cancer Centre
Regional Cancer Centre
Regional Cancer Centre
Regional Cancer Centre
National Cancer Centre
National Cancer Centre
Regional Cancer Centre
Regional Cancer Centre
Regional Cancer Centre
Regional Cancer Centre
Regional Cancer Centre
Regional Cancer Centre
Regional Cancer Centre
National Cancer Centre
National Cancer Centre
Regional Cancer Centre
Regional Cancer Centre
Regional Cancer Centre
Regional Cancer Centre
Regional Cancer Centre
Regional Cancer Centre
Regional Cancer Centre
Regional Cancer Centre
National Cancer Centre
Regional Cancer Centre
National Cancer Centre
Regional Cancer Centre
Regional Cancer Centre
Regional Cancer Centre
Regional Cancer Centre
Regional Cancer Centre
Regional Cancer Centre
Regional Cancer Centre
Regional Cancer Centre
Regional Cancer Centre
Regional Cancer Centre
Regional Cancer Centre
Regional Cancer Centre
Regional Cancer Centre
Regional Cancer Centre
Regional Cancer Centre
Regional Cancer Centre
Regional Cancer Centre
Regional Cancer Centre
Regional Cancer Centre

Regional Cancer Centre
National Cancer Centre
Regional Cancer Centre
National Cancer Centre
Regional Cancer Centre
Regional Cancer Centre
Regional Cancer Centre
Regional Cancer Centre
Regional Cancer Centre
Regional Cancer Centre
Regional Cancer Centre
Regional Cancer Centre
Regional Cancer Centre
Regional Cancer Centre
Regional Cancer Centre
Regional Cancer Centre
Regional Cancer Centre
Regional Cancer Centre
Regional Cancer Centre
Regional Cancer Centre
National Cancer Centre
Regional Cancer Centre
Regional Cancer Centre
Regional Cancer Centre
Regional Cancer Centre
Regional Cancer Centre
Regional Cancer Centre
Regional Cancer Centre
Regional Cancer Centre
Regional Cancer Centre
Regional Cancer Centre
Regional Cancer Centre
Regional Cancer Centre
National Cancer Centre
National Cancer Centre
Regional Cancer Centre
Regional Cancer Centre
Regional Cancer Centre
Regional Cancer Centre
Regional Cancer Centre
Regional Cancer Centre
Regional Cancer Centre
Regional Cancer Centre
Regional Cancer Centre
Regional Cancer Centre
Regional Cancer Centre
Regional Cancer Centre
Regional Cancer Centre
Regional Cancer Centre
Regional Cancer Centre
Regional Cancer Centre

Regional Cancer Centre
Regional Cancer Centre
National Cancer Centre
National Cancer Centre
Regional Cancer Centre
Regional Cancer Centre
Regional Cancer Centre
Regional Cancer Centre
Regional Cancer Centre
Regional Cancer Centre
Regional Cancer Centre
Regional Cancer Centre
Regional Cancer Centre
Regional Cancer Centre
Regional Cancer Centre
Regional Cancer Centre
Regional Cancer Centre
Regional Cancer Centre
Regional Cancer Centre
National Cancer Centre
National Cancer Centre
Regional Cancer Centre
Regional Cancer Centre
Regional Cancer Centre
Regional Cancer Centre
Regional Cancer Centre
Regional Cancer Centre
National Cancer Centre
National Cancer Centre
Regional Cancer Centre
Regional Cancer Centre
Regional Cancer Centre
Regional Cancer Centre
Regional Cancer Centre
Regional Cancer Centre
Regional Cancer Centre
Regional Cancer Centre
Regional Cancer Centre
Regional Cancer Centre
Regional Cancer Centre
Regional Cancer Centre
Regional Cancer Centre
National Cancer Centre
Regional Cancer Centre
Regional Cancer Centre
Regional Cancer Centre
National Cancer Centre
Regional Cancer Centre
Regional Cancer Centre
Regional Cancer Centre

Regional Cancer Centre
Regional Cancer Centre
Regional Cancer Centre
Regional Cancer Centre
Regional Cancer Centre
Regional Cancer Centre
Regional Cancer Centre
Regional Cancer Centre
Regional Cancer Centre
Regional Cancer Centre
Regional Cancer Centre
Regional Cancer Centre
Regional Cancer Centre
Regional Cancer Centre
Regional Cancer Centre
National Cancer Centre
Regional Cancer Centre
National Cancer Centre
Regional Cancer Centre
Regional Cancer Centre
Regional Cancer Centre
Regional Cancer Centre
Regional Cancer Centre
Regional Cancer Centre
Regional Cancer Centre
Regional Cancer Centre
Regional Cancer Centre
Regional Cancer Centre
Regional Cancer Centre
Regional Cancer Centre
Regional Cancer Centre
Regional Cancer Centre
Regional Cancer Centre
Regional Cancer Centre
Regional Cancer Centre
Regional Cancer Centre
Regional Cancer Centre
Regional Cancer Centre
Regional Cancer Centre
Regional Cancer Centre
Regional Cancer Centre
Regional Cancer Centre
Regional Cancer Centre
Regional Cancer Centre
Regional Cancer Centre
Regional Cancer Centre
Regional Cancer Centre
Regional Cancer Centre
Regional Cancer Centre
Regional Cancer Centre

Regional Cancer Centre
Regional Cancer Centre
Regional Cancer Centre
Regional Cancer Centre
Regional Cancer Centre
Regional Cancer Centre
Regional Cancer Centre
Regional Cancer Centre
Regional Cancer Centre
Regional Cancer Centre
Regional Cancer Centre
Regional Cancer Centre
Regional Cancer Centre
Regional Cancer Centre
Regional Cancer Centre
Regional Cancer Centre
Regional Cancer Centre
Regional Cancer Centre
National Cancer Centre
National Cancer Centre
Regional Cancer Centre
Regional Cancer Centre
Regional Cancer Centre
Regional Cancer Centre
Regional Cancer Centre
Regional Cancer Centre
Regional Cancer Centre
Regional Cancer Centre
Regional Cancer Centre
Regional Cancer Centre
Regional Cancer Centre
Regional Cancer Centre
Regional Cancer Centre
Regional Cancer Centre
National Cancer Centre
National Cancer Centre
Regional Cancer Centre
Regional Cancer Centre
Regional Cancer Centre
Regional Cancer Centre
Regional Cancer Centre
Regional Cancer Centre
Regional Cancer Centre
Regional Cancer Centre
Regional Cancer Centre
National Cancer Centre
National Cancer Centre
Regional Cancer Centre
Regional Cancer Centre
Regional Cancer Centre

Regional Cancer Centre
Regional Cancer Centre
Regional Cancer Centre
Regional Cancer Centre
Regional Cancer Centre
Regional Cancer Centre
National Cancer Centre
National Cancer Centre
Regional Cancer Centre
Regional Cancer Centre
Regional Cancer Centre
Regional Cancer Centre
Regional Cancer Centre
Regional Cancer Centre
Regional Cancer Centre
Regional Cancer Centre
Regional Cancer Centre
Regional Cancer Centre
Regional Cancer Centre
Regional Cancer Centre
Regional Cancer Centre
Regional Cancer Centre
Regional Cancer Centre
Regional Cancer Centre
National Cancer Centre
Regional Cancer Centre
Regional Cancer Centre
National Cancer Centre
Regional Cancer Centre
Regional Cancer Centre
Regional Cancer Centre
Regional Cancer Centre
Regional Cancer Centre
Regional Cancer Centre
Regional Cancer Centre
Regional Cancer Centre
Regional Cancer Centre
Regional Cancer Centre
Regional Cancer Centre
Regional Cancer Centre
Regional Cancer Centre
Regional Cancer Centre
Regional Cancer Centre
Regional Cancer Centre
Regional Cancer Centre
Regional Cancer Centre
Regional Cancer Centre
Regional Cancer Centre
Regional Cancer Centre
Regional Cancer Centre

Regional Cancer Centre
Regional Cancer Centre
Regional Cancer Centre
Regional Cancer Centre
Regional Cancer Centre
Regional Cancer Centre
Regional Cancer Centre
Regional Cancer Centre
Regional Cancer Centre
Regional Cancer Centre
Regional Cancer Centre
Regional Cancer Centre
Regional Cancer Centre
Regional Cancer Centre
Regional Cancer Centre
National Cancer Centre
National Cancer Centre
Regional Cancer Centre
Regional Cancer Centre
Regional Cancer Centre
Regional Cancer Centre
Regional Cancer Centre
Regional Cancer Centre
Regional Cancer Centre
Regional Cancer Centre
Regional Cancer Centre
Regional Cancer Centre
Regional Cancer Centre
National Cancer Centre
National Cancer Centre
Regional Cancer Centre
Regional Cancer Centre
Regional Cancer Centre
Regional Cancer Centre
Regional Cancer Centre
Regional Cancer Centre
National Cancer Centre
National Cancer Centre
Regional Cancer Centre
Regional Cancer Centre
Regional Cancer Centre
Regional Cancer Centre
Regional Cancer Centre
National Cancer Centre
Regional Cancer Centre
National Cancer Centre
Regional Cancer Centre
Regional Cancer Centre
Regional Cancer Centre
Regional Cancer Centre

Regional Cancer Centre
Regional Cancer Centre
Regional Cancer Centre
Regional Cancer Centre
Regional Cancer Centre
Regional Cancer Centre
Regional Cancer Centre
Regional Cancer Centre
National Cancer Centre
Regional Cancer Centre
Regional Cancer Centre
Regional Cancer Centre
Regional Cancer Centre
Regional Cancer Centre
Regional Cancer Centre
National Cancer Centre
Regional Cancer Centre
Regional Cancer Centre
Regional Cancer Centre
Regional Cancer Centre
Regional Cancer Centre
Regional Cancer Centre
Regional Cancer Centre
Regional Cancer Centre
Regional Cancer Centre
Regional Cancer Centre
Regional Cancer Centre
Regional Cancer Centre
Regional Cancer Centre
Regional Cancer Centre
Regional Cancer Centre
Regional Cancer Centre
Regional Cancer Centre
Regional Cancer Centre
Regional Cancer Centre
Regional Cancer Centre
Regional Cancer Centre
Regional Cancer Centre
Regional Cancer Centre
Regional Cancer Centre
Regional Cancer Centre
Regional Cancer Centre
Regional Cancer Centre
Regional Cancer Centre
Regional Cancer Centre
Regional Cancer Centre
National Cancer Centre
National Cancer Centre
Regional Cancer Centre
Regional Cancer Centre

Regional Cancer Centre
Regional Cancer Centre
Regional Cancer Centre
Regional Cancer Centre
Regional Cancer Centre
Regional Cancer Centre
Regional Cancer Centre
Regional Cancer Centre
National Cancer Centre
National Cancer Centre
Regional Cancer Centre
Regional Cancer Centre
Regional Cancer Centre
Regional Cancer Centre
Regional Cancer Centre
Regional Cancer Centre
Regional Cancer Centre
Regional Cancer Centre
National Cancer Centre
National Cancer Centre
Regional Cancer Centre
Regional Cancer Centre
Regional Cancer Centre
Regional Cancer Centre
National Cancer Centre
Regional Cancer Centre
National Cancer Centre
Regional Cancer Centre
Regional Cancer Centre
Regional Cancer Centre
Regional Cancer Centre
Regional Cancer Centre
Regional Cancer Centre
Regional Cancer Centre
Regional Cancer Centre
Regional Cancer Centre
Regional Cancer Centre
Regional Cancer Centre
Regional Cancer Centre
Regional Cancer Centre
National Cancer Centre
National Cancer Centre
Regional Cancer Centre
Regional Cancer Centre
Regional Cancer Centre
Regional Cancer Centre
Regional Cancer Centre
Regional Cancer Centre
Regional Cancer Centre
Regional Cancer Centre
Regional Cancer Centre

Regional Cancer Centre
Regional Cancer Centre
Regional Cancer Centre
Regional Cancer Centre
Regional Cancer Centre
Regional Cancer Centre
Regional Cancer Centre
Regional Cancer Centre
Regional Cancer Centre
Regional Cancer Centre
Regional Cancer Centre
Regional Cancer Centre
Regional Cancer Centre
Regional Cancer Centre
Regional Cancer Centre
Regional Cancer Centre
Regional Cancer Centre
National Cancer Centre
National Cancer Centre
Regional Cancer Centre
Regional Cancer Centre
Regional Cancer Centre
Regional Cancer Centre
Regional Cancer Centre
Regional Cancer Centre
Regional Cancer Centre
Regional Cancer Centre
National Cancer Centre
National Cancer Centre
Regional Cancer Centre
Regional Cancer Centre
Regional Cancer Centre
Regional Cancer Centre
National Cancer Centre
National Cancer Centre
Regional Cancer Centre
Regional Cancer Centre
Regional Cancer Centre
Regional Cancer Centre
Regional Cancer Centre
Regional Cancer Centre
Regional Cancer Centre
Regional Cancer Centre
National Cancer Centre
National Cancer Centre
Regional Cancer Centre
Regional Cancer Centre
Regional Cancer Centre
Regional Cancer Centre
Regional Cancer Centre

Regional Cancer Centre
Regional Cancer Centre
Regional Cancer Centre
Regional Cancer Centre
Regional Cancer Centre
Regional Cancer Centre
Regional Cancer Centre
Regional Cancer Centre
Regional Cancer Centre
Regional Cancer Centre
National Cancer Centre
National Cancer Centre
Regional Cancer Centre
Regional Cancer Centre
Regional Cancer Centre
Regional Cancer Centre
Regional Cancer Centre
Regional Cancer Centre
Regional Cancer Centre
Regional Cancer Centre
Regional Cancer Centre
Regional Cancer Centre
Regional Cancer Centre
Regional Cancer Centre
Regional Cancer Centre
Regional Cancer Centre
Regional Cancer Centre
Regional Cancer Centre
Regional Cancer Centre
Regional Cancer Centre
Regional Cancer Centre
Regional Cancer Centre
Regional Cancer Centre
Regional Cancer Centre
Regional Cancer Centre
Regional Cancer Centre
Regional Cancer Centre
Regional Cancer Centre
Regional Cancer Centre
Regional Cancer Centre
Regional Cancer Centre
Regional Cancer Centre
Regional Cancer Centre
Regional Cancer Centre
Regional Cancer Centre
Regional Cancer Centre
Regional Cancer Centre
Regional Cancer Centre
Regional Cancer Centre
Regional Cancer Centre

Regional Cancer Centre
Regional Cancer Centre
Regional Cancer Centre
Regional Cancer Centre
Regional Cancer Centre
Regional Cancer Centre
Regional Cancer Centre
Regional Cancer Centre
Regional Cancer Centre
Regional Cancer Centre
Regional Cancer Centre
Regional Cancer Centre
Regional Cancer Centre
Regional Cancer Centre
Regional Cancer Centre
Regional Cancer Centre
Regional Cancer Centre
Regional Cancer Centre
Regional Cancer Centre
Regional Cancer Centre
Regional Cancer Centre
Regional Cancer Centre
Regional Cancer Centre
Regional Cancer Centre
Regional Cancer Centre
Regional Cancer Centre
Regional Cancer Centre
Regional Cancer Centre
Regional Cancer Centre
Regional Cancer Centre
Regional Cancer Centre
Regional Cancer Centre
Regional Cancer Centre
Regional Cancer Centre
Regional Cancer Centre
Regional Cancer Centre
Regional Cancer Centre
Regional Cancer Centre
Regional Cancer Centre
Regional Cancer Centre
Regional Cancer Centre
Regional Cancer Centre
Regional Cancer Centre
Regional Cancer Centre
Regional Cancer Centre
Regional Cancer Centre
Regional Cancer Centre
Regional Cancer Centre
Regional Cancer Centre
Regional Cancer Centre

Regional Cancer Centre
Regional Cancer Centre
Regional Cancer Centre
Regional Cancer Centre
Regional Cancer Centre
Regional Cancer Centre
Regional Cancer Centre
Regional Cancer Centre
Regional Cancer Centre
Regional Cancer Centre
Regional Cancer Centre
Regional Cancer Centre
Regional Cancer Centre
Regional Cancer Centre
Regional Cancer Centre
Regional Cancer Centre
Regional Cancer Centre
Regional Cancer Centre
Regional Cancer Centre
Regional Cancer Centre
Regional Cancer Centre
Regional Cancer Centre
Regional Cancer Centre
Regional Cancer Centre
Regional Cancer Centre
Regional Cancer Centre
Regional Cancer Centre
Regional Cancer Centre
Regional Cancer Centre
Regional Cancer Centre
Regional Cancer Centre
Regional Cancer Centre
Regional Cancer Centre
Regional Cancer Centre
Regional Cancer Centre
Regional Cancer Centre
Regional Cancer Centre
Regional Cancer Centre
Regional Cancer Centre
Regional Cancer Centre
Regional Cancer Centre
Regional Cancer Centre
Regional Cancer Centre
Regional Cancer Centre
Regional Cancer Centre
Regional Cancer Centre
Regional Cancer Centre
Regional Cancer Centre
Regional Cancer Centre
Regional Cancer Centre

Regional Cancer Centre
Regional Cancer Centre
Regional Cancer Centre
Regional Cancer Centre
Regional Cancer Centre
Regional Cancer Centre
Regional Cancer Centre
Regional Cancer Centre
Regional Cancer Centre
Regional Cancer Centre
Regional Cancer Centre
Regional Cancer Centre
Regional Cancer Centre
Regional Cancer Centre
Regional Cancer Centre
Regional Cancer Centre
Regional Cancer Centre
Regional Cancer Centre
Regional Cancer Centre
Regional Cancer Centre
Regional Cancer Centre
Regional Cancer Centre
Regional Cancer Centre
Regional Cancer Centre
Regional Cancer Centre
Regional Cancer Centre
Regional Cancer Centre
Regional Cancer Centre
Regional Cancer Centre
Regional Cancer Centre
Regional Cancer Centre
Regional Cancer Centre
Regional Cancer Centre
Regional Cancer Centre
Regional Cancer Centre
Regional Cancer Centre
Regional Cancer Centre
Regional Cancer Centre
Regional Cancer Centre
Regional Cancer Centre
Regional Cancer Centre
Regional Cancer Centre
Regional Cancer Centre
Regional Cancer Centre
Regional Cancer Centre
Regional Cancer Centre
Regional Cancer Centre
Regional Cancer Centre
Regional Cancer Centre
Regional Cancer Centre

Regional Cancer Centre
Regional Cancer Centre
Regional Cancer Centre
Regional Cancer Centre
Regional Cancer Centre
Regional Cancer Centre
Regional Cancer Centre
Regional Cancer Centre
Regional Cancer Centre
Regional Cancer Centre
Regional Cancer Centre
Regional Cancer Centre
Regional Cancer Centre
Regional Cancer Centre
Regional Cancer Centre
Regional Cancer Centre
Regional Cancer Centre
Regional Cancer Centre
Regional Cancer Centre
Regional Cancer Centre
Regional Cancer Centre
Regional Cancer Centre
Regional Cancer Centre
Regional Cancer Centre
Regional Cancer Centre
Regional Cancer Centre
Regional Cancer Centre
Regional Cancer Centre
Regional Cancer Centre
Regional Cancer Centre
Regional Cancer Centre
Regional Cancer Centre
Regional Cancer Centre
Regional Cancer Centre
Regional Cancer Centre
Regional Cancer Centre
Regional Cancer Centre
Regional Cancer Centre
Regional Cancer Centre
Regional Cancer Centre
Regional Cancer Centre
Regional Cancer Centre
Regional Cancer Centre
Regional Cancer Centre
Regional Cancer Centre
Regional Cancer Centre
Regional Cancer Centre
Regional Cancer Centre
Regional Cancer Centre
Regional Cancer Centre

Regional Cancer Centre
Regional Cancer Centre
Regional Cancer Centre
Regional Cancer Centre
Regional Cancer Centre
Regional Cancer Centre
Regional Cancer Centre
Regional Cancer Centre
Regional Cancer Centre
Regional Cancer Centre
Regional Cancer Centre
Regional Cancer Centre
Regional Cancer Centre
Regional Cancer Centre
Regional Cancer Centre
Regional Cancer Centre
Regional Cancer Centre
Regional Cancer Centre
Regional Cancer Centre
Regional Cancer Centre
Regional Cancer Centre
Regional Cancer Centre
Regional Cancer Centre
Regional Cancer Centre
Regional Cancer Centre
Regional Cancer Centre
Regional Cancer Centre
Regional Cancer Centre
Regional Cancer Centre
Regional Cancer Centre
Regional Cancer Centre
Regional Cancer Centre
Regional Cancer Centre
Regional Cancer Centre
Regional Cancer Centre
Regional Cancer Centre
Regional Cancer Centre
Regional Cancer Centre
Regional Cancer Centre
Regional Cancer Centre
Regional Cancer Centre
Regional Cancer Centre
Regional Cancer Centre
Regional Cancer Centre
Regional Cancer Centre
Regional Cancer Centre
Regional Cancer Centre
Regional Cancer Centre
Regional Cancer Centre
Regional Cancer Centre

Regional Cancer Centre
Regional Cancer Centre
Regional Cancer Centre
Regional Cancer Centre
Regional Cancer Centre
Regional Cancer Centre
Regional Cancer Centre
Regional Cancer Centre
Regional Cancer Centre
Regional Cancer Centre
Regional Cancer Centre
Regional Cancer Centre
Regional Cancer Centre
Regional Cancer Centre
Regional Cancer Centre
Regional Cancer Centre
Regional Cancer Centre
Regional Cancer Centre
Regional Cancer Centre
Regional Cancer Centre
Regional Cancer Centre
Regional Cancer Centre
Regional Cancer Centre
Regional Cancer Centre
Regional Cancer Centre
Regional Cancer Centre
Regional Cancer Centre
Regional Cancer Centre
Regional Cancer Centre
Regional Cancer Centre
Regional Cancer Centre
Regional Cancer Centre
Regional Cancer Centre
Regional Cancer Centre
Regional Cancer Centre
Regional Cancer Centre
Regional Cancer Centre
Regional Cancer Centre
Regional Cancer Centre
Regional Cancer Centre
Regional Cancer Centre
Regional Cancer Centre
Regional Cancer Centre
Regional Cancer Centre
Regional Cancer Centre
Regional Cancer Centre
Regional Cancer Centre
Regional Cancer Centre
Regional Cancer Centre
Regional Cancer Centre

Regional Cancer Centre
Regional Cancer Centre
Regional Cancer Centre
Regional Cancer Centre
Regional Cancer Centre
Regional Cancer Centre
Regional Cancer Centre
Regional Cancer Centre
Regional Cancer Centre
Regional Cancer Centre
Regional Cancer Centre
Regional Cancer Centre
Regional Cancer Centre
Regional Cancer Centre
Regional Cancer Centre
Regional Cancer Centre
Regional Cancer Centre
Regional Cancer Centre
Regional Cancer Centre
Regional Cancer Centre
Regional Cancer Centre
Regional Cancer Centre
Regional Cancer Centre
Regional Cancer Centre
Regional Cancer Centre
Regional Cancer Centre
Regional Cancer Centre
Regional Cancer Centre
Regional Cancer Centre
Regional Cancer Centre
Regional Cancer Centre
Regional Cancer Centre
Regional Cancer Centre
Regional Cancer Centre
Regional Cancer Centre
Regional Cancer Centre
Regional Cancer Centre
Regional Cancer Centre
Regional Cancer Centre
Regional Cancer Centre
Regional Cancer Centre
Regional Cancer Centre
Regional Cancer Centre
Regional Cancer Centre
Regional Cancer Centre
Regional Cancer Centre
Regional Cancer Centre
Regional Cancer Centre
Regional Cancer Centre
Regional Cancer Centre

Regional Cancer Centre
Regional Cancer Centre
Regional Cancer Centre
Regional Cancer Centre
Regional Cancer Centre
Regional Cancer Centre
Regional Cancer Centre
Regional Cancer Centre
Regional Cancer Centre
Regional Cancer Centre
Regional Cancer Centre
Regional Cancer Centre
Regional Cancer Centre
Regional Cancer Centre
Regional Cancer Centre
Regional Cancer Centre
Regional Cancer Centre
Regional Cancer Centre
Regional Cancer Centre
Regional Cancer Centre
Regional Cancer Centre
Regional Cancer Centre
Regional Cancer Centre
Regional Cancer Centre
Regional Cancer Centre
Regional Cancer Centre
Regional Cancer Centre
Regional Cancer Centre
Regional Cancer Centre
Regional Cancer Centre
Regional Cancer Centre
Regional Cancer Centre
Regional Cancer Centre
Regional Cancer Centre
Regional Cancer Centre
Regional Cancer Centre
Regional Cancer Centre
Regional Cancer Centre
Regional Cancer Centre
Regional Cancer Centre
Regional Cancer Centre
Regional Cancer Centre
Regional Cancer Centre
Regional Cancer Centre
Regional Cancer Centre
Regional Cancer Centre
Regional Cancer Centre
Regional Cancer Centre
Regional Cancer Centre
Regional Cancer Centre

Regional Cancer Centre
Regional Cancer Centre
Regional Cancer Centre
Regional Cancer Centre
Regional Cancer Centre
Regional Cancer Centre
Regional Cancer Centre
Regional Cancer Centre
Regional Cancer Centre
Regional Cancer Centre
Regional Cancer Centre
Regional Cancer Centre
Regional Cancer Centre
Regional Cancer Centre
Regional Cancer Centre
Regional Cancer Centre
Regional Cancer Centre
Regional Cancer Centre
Regional Cancer Centre
Regional Cancer Centre
Regional Cancer Centre
Regional Cancer Centre
Regional Cancer Centre
Regional Cancer Centre
Regional Cancer Centre
Regional Cancer Centre
Regional Cancer Centre
Regional Cancer Centre
Regional Cancer Centre
Regional Cancer Centre
Regional Cancer Centre
Regional Cancer Centre
Regional Cancer Centre
Regional Cancer Centre
Regional Cancer Centre
Regional Cancer Centre
Regional Cancer Centre
Regional Cancer Centre
Regional Cancer Centre
Regional Cancer Centre
Regional Cancer Centre
Regional Cancer Centre
Regional Cancer Centre
Regional Cancer Centre
Regional Cancer Centre
Regional Cancer Centre
Regional Cancer Centre
Regional Cancer Centre
Regional Cancer Centre
Regional Cancer Centre

Regional Cancer Centre
Regional Cancer Centre
Regional Cancer Centre
Regional Cancer Centre
Regional Cancer Centre
Regional Cancer Centre
Regional Cancer Centre
Regional Cancer Centre
Regional Cancer Centre
Regional Cancer Centre
Regional Cancer Centre
Regional Cancer Centre
Regional Cancer Centre
Regional Cancer Centre
Regional Cancer Centre
Regional Cancer Centre
Regional Cancer Centre
Regional Cancer Centre
Regional Cancer Centre
Regional Cancer Centre
Regional Cancer Centre
Regional Cancer Centre
Regional Cancer Centre
Regional Cancer Centre
Regional Cancer Centre
Regional Cancer Centre
Regional Cancer Centre
Regional Cancer Centre
Regional Cancer Centre
Regional Cancer Centre
Regional Cancer Centre
Regional Cancer Centre
Regional Cancer Centre
Regional Cancer Centre
Regional Cancer Centre
Regional Cancer Centre
Regional Cancer Centre
Regional Cancer Centre
Regional Cancer Centre
Regional Cancer Centre
Regional Cancer Centre
Regional Cancer Centre
Regional Cancer Centre
Regional Cancer Centre
Regional Cancer Centre
Regional Cancer Centre
Regional Cancer Centre
Regional Cancer Centre
Regional Cancer Centre
Regional Cancer Centre

Regional Cancer Centre
Regional Cancer Centre
Regional Cancer Centre
Regional Cancer Centre
Regional Cancer Centre
Regional Cancer Centre
Regional Cancer Centre
Regional Cancer Centre
Regional Cancer Centre
Regional Cancer Centre
Regional Cancer Centre
Regional Cancer Centre
Regional Cancer Centre
Regional Cancer Centre
Regional Cancer Centre
Regional Cancer Centre
Regional Cancer Centre
Regional Cancer Centre
Regional Cancer Centre
Regional Cancer Centre
Regional Cancer Centre
Regional Cancer Centre
Regional Cancer Centre
Regional Cancer Centre
Regional Cancer Centre
Regional Cancer Centre
Regional Cancer Centre
Regional Cancer Centre
Regional Cancer Centre
Regional Cancer Centre
Regional Cancer Centre
Regional Cancer Centre
Regional Cancer Centre
Regional Cancer Centre
Regional Cancer Centre
Regional Cancer Centre
Regional Cancer Centre
Regional Cancer Centre
Regional Cancer Centre
Regional Cancer Centre
Regional Cancer Centre
Regional Cancer Centre
Regional Cancer Centre
Regional Cancer Centre
Regional Cancer Centre
Regional Cancer Centre
Regional Cancer Centre
Regional Cancer Centre
Regional Cancer Centre
Regional Cancer Centre

Regional Cancer Centre
Regional Cancer Centre
Regional Cancer Centre
Regional Cancer Centre
Regional Cancer Centre
Regional Cancer Centre
Regional Cancer Centre
Regional Cancer Centre
Regional Cancer Centre
Regional Cancer Centre
Regional Cancer Centre
Regional Cancer Centre
Regional Cancer Centre
Regional Cancer Centre
Regional Cancer Centre
Regional Cancer Centre
Regional Cancer Centre
Regional Cancer Centre
Regional Cancer Centre
Regional Cancer Centre
Regional Cancer Centre
Regional Cancer Centre
Regional Cancer Centre
Regional Cancer Centre
Regional Cancer Centre
Regional Cancer Centre
Regional Cancer Centre
Regional Cancer Centre
Regional Cancer Centre
Regional Cancer Centre
Regional Cancer Centre
Regional Cancer Centre
Regional Cancer Centre
Regional Cancer Centre
Regional Cancer Centre
Regional Cancer Centre
Regional Cancer Centre
Regional Cancer Centre
Regional Cancer Centre
Regional Cancer Centre
Regional Cancer Centre
Regional Cancer Centre
Regional Cancer Centre
Regional Cancer Centre
Regional Cancer Centre
Regional Cancer Centre
Regional Cancer Centre
Regional Cancer Centre
Regional Cancer Centre
Regional Cancer Centre

Regional Cancer Centre
Regional Cancer Centre
Regional Cancer Centre
Regional Cancer Centre
Regional Cancer Centre
Regional Cancer Centre
Regional Cancer Centre
Regional Cancer Centre
Regional Cancer Centre
Regional Cancer Centre
Regional Cancer Centre
Regional Cancer Centre
Regional Cancer Centre
Regional Cancer Centre
Regional Cancer Centre
Regional Cancer Centre
Regional Cancer Centre
Regional Cancer Centre
Regional Cancer Centre
Regional Cancer Centre
Regional Cancer Centre
Regional Cancer Centre
Regional Cancer Centre
Regional Cancer Centre
Regional Cancer Centre
Regional Cancer Centre
Regional Cancer Centre
Regional Cancer Centre
Regional Cancer Centre
Regional Cancer Centre
Regional Cancer Centre
Regional Cancer Centre
Regional Cancer Centre
Regional Cancer Centre
Regional Cancer Centre
Regional Cancer Centre
Regional Cancer Centre
Regional Cancer Centre
Regional Cancer Centre
Regional Cancer Centre
Regional Cancer Centre
Regional Cancer Centre
Regional Cancer Centre
Regional Cancer Centre
Regional Cancer Centre
Regional Cancer Centre
Regional Cancer Centre
Regional Cancer Centre
Regional Cancer Centre
Regional Cancer Centre

Regional Cancer Centre
Regional Cancer Centre
Regional Cancer Centre
Regional Cancer Centre
Regional Cancer Centre
Regional Cancer Centre
Regional Cancer Centre
Regional Cancer Centre
Regional Cancer Centre
Regional Cancer Centre
Regional Cancer Centre
Regional Cancer Centre
Regional Cancer Centre
Regional Cancer Centre
Regional Cancer Centre
Regional Cancer Centre
Regional Cancer Centre
Regional Cancer Centre
Regional Cancer Centre
Regional Cancer Centre
Regional Cancer Centre
Regional Cancer Centre
Regional Cancer Centre
Regional Cancer Centre
Regional Cancer Centre
Regional Cancer Centre
Regional Cancer Centre
Regional Cancer Centre
Regional Cancer Centre
Regional Cancer Centre
Regional Cancer Centre
Regional Cancer Centre
Regional Cancer Centre
Regional Cancer Centre
Regional Cancer Centre
Regional Cancer Centre
Regional Cancer Centre
Regional Cancer Centre
Regional Cancer Centre
Regional Cancer Centre
Regional Cancer Centre
Regional Cancer Centre
Regional Cancer Centre
Regional Cancer Centre
Regional Cancer Centre
Regional Cancer Centre
Regional Cancer Centre
Regional Cancer Centre
Regional Cancer Centre
Regional Cancer Centre

Regional Cancer Centre
Regional Cancer Centre
Regional Cancer Centre
Regional Cancer Centre
Regional Cancer Centre
Regional Cancer Centre
Regional Cancer Centre
Regional Cancer Centre
Regional Cancer Centre
Regional Cancer Centre
Regional Cancer Centre
Regional Cancer Centre
Regional Cancer Centre
Regional Cancer Centre
Regional Cancer Centre
Regional Cancer Centre
Regional Cancer Centre
Regional Cancer Centre
Regional Cancer Centre
Regional Cancer Centre
Regional Cancer Centre
Regional Cancer Centre
Regional Cancer Centre
Regional Cancer Centre
Regional Cancer Centre
Regional Cancer Centre
Regional Cancer Centre
Regional Cancer Centre
Regional Cancer Centre
Regional Cancer Centre
Regional Cancer Centre
Regional Cancer Centre
Regional Cancer Centre
Regional Cancer Centre
Regional Cancer Centre
Regional Cancer Centre
Regional Cancer Centre
Regional Cancer Centre
Regional Cancer Centre
Regional Cancer Centre
Regional Cancer Centre
Regional Cancer Centre
Regional Cancer Centre
Regional Cancer Centre
Regional Cancer Centre
Regional Cancer Centre
Regional Cancer Centre
Regional Cancer Centre
Regional Cancer Centre
Regional Cancer Centre

Regional Cancer Centre
Regional Cancer Centre
Regional Cancer Centre
Regional Cancer Centre
Regional Cancer Centre
Regional Cancer Centre
Regional Cancer Centre
Regional Cancer Centre
Regional Cancer Centre
Regional Cancer Centre
Regional Cancer Centre
Regional Cancer Centre
Regional Cancer Centre
Regional Cancer Centre
Regional Cancer Centre
Regional Cancer Centre
Regional Cancer Centre
Regional Cancer Centre
Regional Cancer Centre
Regional Cancer Centre
Regional Cancer Centre
Regional Cancer Centre
Regional Cancer Centre
Regional Cancer Centre
Regional Cancer Centre
Regional Cancer Centre
Regional Cancer Centre
Regional Cancer Centre
Regional Cancer Centre
Regional Cancer Centre
Regional Cancer Centre
Regional Cancer Centre
Regional Cancer Centre
Regional Cancer Centre
Regional Cancer Centre
Regional Cancer Centre
Regional Cancer Centre
Regional Cancer Centre
Regional Cancer Centre
Regional Cancer Centre
Regional Cancer Centre
Regional Cancer Centre
Regional Cancer Centre
Regional Cancer Centre
Regional Cancer Centre
Regional Cancer Centre
Regional Cancer Centre
Regional Cancer Centre
Regional Cancer Centre
Regional Cancer Centre

Regional Cancer Centre
Regional Cancer Centre
Regional Cancer Centre
Regional Cancer Centre
Regional Cancer Centre
Regional Cancer Centre
Regional Cancer Centre
Regional Cancer Centre
Regional Cancer Centre
Regional Cancer Centre
Regional Cancer Centre
Regional Cancer Centre
Regional Cancer Centre
Regional Cancer Centre
Regional Cancer Centre
Regional Cancer Centre
Regional Cancer Centre
Regional Cancer Centre
Regional Cancer Centre
Regional Cancer Centre
Regional Cancer Centre
Regional Cancer Centre
Regional Cancer Centre
Regional Cancer Centre
Regional Cancer Centre
Regional Cancer Centre
Regional Cancer Centre
Regional Cancer Centre
Regional Cancer Centre
Regional Cancer Centre
Regional Cancer Centre
Regional Cancer Centre
Regional Cancer Centre
Regional Cancer Centre
Regional Cancer Centre
Regional Cancer Centre
Regional Cancer Centre
Regional Cancer Centre
Regional Cancer Centre
Regional Cancer Centre
Regional Cancer Centre
Regional Cancer Centre
Regional Cancer Centre
Regional Cancer Centre
Regional Cancer Centre
Regional Cancer Centre
Regional Cancer Centre
Regional Cancer Centre
Regional Cancer Centre
Regional Cancer Centre

Regional Cancer Centre
Regional Cancer Centre
Regional Cancer Centre
Regional Cancer Centre
Regional Cancer Centre
Regional Cancer Centre
Regional Cancer Centre
Regional Cancer Centre
Regional Cancer Centre
Regional Cancer Centre
Regional Cancer Centre
Regional Cancer Centre
Regional Cancer Centre
Regional Cancer Centre
Regional Cancer Centre
Regional Cancer Centre
Regional Cancer Centre
Regional Cancer Centre
Regional Cancer Centre
Regional Cancer Centre
Regional Cancer Centre
Regional Cancer Centre
Regional Cancer Centre
Regional Cancer Centre
National Cancer Centre
National Cancer Centre
National Cancer Centre
National Cancer Centre
National Cancer Centre
National Cancer Centre
National Cancer Centre
National Cancer Centre
National Cancer Centre
National Cancer Centre
National Cancer Centre
National Cancer Centre
National Cancer Centre
National Cancer Centre
National Cancer Centre
National Cancer Centre
National Cancer Centre
National Cancer Centre
National Cancer Centre
National Cancer Centre
National Cancer Centre
National Cancer Centre
National Cancer Centre
National Cancer Centre
National Cancer Centre
National Cancer Centre

National Cancer Centre
National Cancer Centre
National Cancer Centre
National Cancer Centre
National Cancer Centre
National Cancer Centre
National Cancer Centre
National Cancer Centre
National Cancer Centre
National Cancer Centre
National Cancer Centre
National Cancer Centre
National Cancer Centre
National Cancer Centre
National Cancer Centre
National Cancer Centre
National Cancer Centre
National Cancer Centre
National Cancer Centre
National Cancer Centre
National Cancer Centre
National Cancer Centre
National Cancer Centre
National Cancer Centre
National Cancer Centre
National Cancer Centre
National Cancer Centre
National Cancer Centre
National Cancer Centre
National Cancer Centre
National Cancer Centre
National Cancer Centre
National Cancer Centre
National Cancer Centre
National Cancer Centre
National Cancer Centre
National Cancer Centre
National Cancer Centre
National Cancer Centre
National Cancer Centre
National Cancer Centre
National Cancer Centre
National Cancer Centre
National Cancer Centre
National Cancer Centre
National Cancer Centre
National Cancer Centre
National Cancer Centre
National Cancer Centre
National Cancer Centre

National Cancer Centre
National Cancer Centre
National Cancer Centre
National Cancer Centre
National Cancer Centre
National Cancer Centre
National Cancer Centre
National Cancer Centre
National Cancer Centre
National Cancer Centre
National Cancer Centre
National Cancer Centre
National Cancer Centre
National Cancer Centre
National Cancer Centre
National Cancer Centre
National Cancer Centre
National Cancer Centre
National Cancer Centre
National Cancer Centre
National Cancer Centre
National Cancer Centre
National Cancer Centre
National Cancer Centre
National Cancer Centre
National Cancer Centre
National Cancer Centre
National Cancer Centre
National Cancer Centre
National Cancer Centre
National Cancer Centre
National Cancer Centre
National Cancer Centre
National Cancer Centre
National Cancer Centre
National Cancer Centre
National Cancer Centre
National Cancer Centre
National Cancer Centre
National Cancer Centre
National Cancer Centre
National Cancer Centre
National Cancer Centre
National Cancer Centre
National Cancer Centre
National Cancer Centre
National Cancer Centre
National Cancer Centre
National Cancer Centre
National Cancer Centre

National Cancer Centre
National Cancer Centre
National Cancer Centre
National Cancer Centre
National Cancer Centre
National Cancer Centre
National Cancer Centre
National Cancer Centre
National Cancer Centre
National Cancer Centre
National Cancer Centre
National Cancer Centre
National Cancer Centre
National Cancer Centre
National Cancer Centre
National Cancer Centre
National Cancer Centre
National Cancer Centre
National Cancer Centre
National Cancer Centre
National Cancer Centre
National Cancer Centre
National Cancer Centre
National Cancer Centre
National Cancer Centre
National Cancer Centre
National Cancer Centre
National Cancer Centre
National Cancer Centre
National Cancer Centre
National Cancer Centre
National Cancer Centre
National Cancer Centre
National Cancer Centre
National Cancer Centre
National Cancer Centre
National Cancer Centre
National Cancer Centre
National Cancer Centre
National Cancer Centre
National Cancer Centre
National Cancer Centre
National Cancer Centre
National Cancer Centre
National Cancer Centre
National Cancer Centre
National Cancer Centre
National Cancer Centre
National Cancer Centre
National Cancer Centre

National Cancer Centre
National Cancer Centre
National Cancer Centre
National Cancer Centre
National Cancer Centre
National Cancer Centre
National Cancer Centre
National Cancer Centre
National Cancer Centre
Regional Cancer Centre
Regional Cancer Centre
Regional Cancer Centre
Regional Cancer Centre
Regional Cancer Centre
Regional Cancer Centre
Regional Cancer Centre
Regional Cancer Centre
Regional Cancer Centre
Regional Cancer Centre
Regional Cancer Centre
Regional Cancer Centre
Regional Cancer Centre
Regional Cancer Centre
Regional Cancer Centre
Regional Cancer Centre
Regional Cancer Centre
Regional Cancer Centre
Regional Cancer Centre
Regional Cancer Centre
Regional Cancer Centre
Regional Cancer Centre
Regional Cancer Centre
Regional Cancer Centre
Regional Cancer Centre
Regional Cancer Centre
Regional Cancer Centre
Regional Cancer Centre
Regional Cancer Centre
Regional Cancer Centre
Regional Cancer Centre
Regional Cancer Centre
Regional Cancer Centre
Regional Cancer Centre
Regional Cancer Centre
Regional Cancer Centre
Regional Cancer Centre
Regional Cancer Centre
Regional Cancer Centre
Regional Cancer Centre
Regional Cancer Centre

Regional Cancer Centre
Regional Cancer Centre
Regional Cancer Centre
Regional Cancer Centre
Regional Cancer Centre
Regional Cancer Centre
Regional Cancer Centre
Regional Cancer Centre
Regional Cancer Centre
Regional Cancer Centre
Regional Cancer Centre
Regional Cancer Centre
Regional Cancer Centre
Regional Cancer Centre
Regional Cancer Centre
Regional Cancer Centre
Regional Cancer Centre
Regional Cancer Centre
Regional Cancer Centre
Regional Cancer Centre
Regional Cancer Centre
Regional Cancer Centre
Regional Cancer Centre
Regional Cancer Centre
Regional Cancer Centre
Regional Cancer Centre
Regional Cancer Centre
Regional Cancer Centre
Regional Cancer Centre
Regional Cancer Centre
Regional Cancer Centre
Regional Cancer Centre
Regional Cancer Centre
Regional Cancer Centre
Regional Cancer Centre
Regional Cancer Centre
Regional Cancer Centre
Regional Cancer Centre
Regional Cancer Centre
Regional Cancer Centre
Regional Cancer Centre
Regional Cancer Centre
Regional Cancer Centre
Regional Cancer Centre
Regional Cancer Centre
Regional Cancer Centre
Regional Cancer Centre
Regional Cancer Centre
Regional Cancer Centre
Regional Cancer Centre

Regional Cancer Centre
Regional Cancer Centre
Regional Cancer Centre
Regional Cancer Centre
Regional Cancer Centre
National Cancer Centre
National Cancer Centre
National Cancer Centre
National Cancer Centre
National Cancer Centre
National Cancer Centre
National Cancer Centre
National Cancer Centre
National Cancer Centre
National Cancer Centre
National Cancer Centre
National Cancer Centre
National Cancer Centre
National Cancer Centre
National Cancer Centre
National Cancer Centre
National Cancer Centre
National Cancer Centre
National Cancer Centre
National Cancer Centre
National Cancer Centre
National Cancer Centre
National Cancer Centre
National Cancer Centre
National Cancer Centre
National Cancer Centre
National Cancer Centre
National Cancer Centre
National Cancer Centre
National Cancer Centre
National Cancer Centre
National Cancer Centre
National Cancer Centre
National Cancer Centre
National Cancer Centre
National Cancer Centre
National Cancer Centre
National Cancer Centre
National Cancer Centre
National Cancer Centre
National Cancer Centre
National Cancer Centre
National Cancer Centre
National Cancer Centre
National Cancer Centre

National Cancer Centre
National Cancer Centre
National Cancer Centre
National Cancer Centre
National Cancer Centre
National Cancer Centre
National Cancer Centre
National Cancer Centre
National Cancer Centre
National Cancer Centre
National Cancer Centre
National Cancer Centre
National Cancer Centre
National Cancer Centre
National Cancer Centre
National Cancer Centre
National Cancer Centre
National Cancer Centre
National Cancer Centre
National Cancer Centre
National Cancer Centre
National Cancer Centre
National Cancer Centre
National Cancer Centre
National Cancer Centre
National Cancer Centre
National Cancer Centre
National Cancer Centre
National Cancer Centre
National Cancer Centre
National Cancer Centre
National Cancer Centre
National Cancer Centre
National Cancer Centre
National Cancer Centre
National Cancer Centre
National Cancer Centre
National Cancer Centre
National Cancer Centre
National Cancer Centre
National Cancer Centre
National Cancer Centre
National Cancer Centre
National Cancer Centre
National Cancer Centre
National Cancer Centre
National Cancer Centre
National Cancer Centre
National Cancer Centre
National Cancer Centre

National Cancer Centre
National Cancer Centre
National Cancer Centre
National Cancer Centre
National Cancer Centre
National Cancer Centre
National Cancer Centre
National Cancer Centre
National Cancer Centre
National Cancer Centre
National Cancer Centre
National Cancer Centre
National Cancer Centre
National Cancer Centre
National Cancer Centre
National Cancer Centre
National Cancer Centre
National Cancer Centre
National Cancer Centre
National Cancer Centre
National Cancer Centre
National Cancer Centre
National Cancer Centre
National Cancer Centre
National Cancer Centre
National Cancer Centre
National Cancer Centre
National Cancer Centre
National Cancer Centre
National Cancer Centre
National Cancer Centre
National Cancer Centre
National Cancer Centre
National Cancer Centre
National Cancer Centre
National Cancer Centre
National Cancer Centre
National Cancer Centre
National Cancer Centre
National Cancer Centre
Regional Cancer Centre
Regional Cancer Centre
Regional Cancer Centre
Regional Cancer Centre
Regional Cancer Centre
Regional Cancer Centre
Regional Cancer Centre
Regional Cancer Centre
Regional Cancer Centre
Regional Cancer Centre

Regional Cancer Centre
Regional Cancer Centre
Regional Cancer Centre
Regional Cancer Centre
Regional Cancer Centre
Regional Cancer Centre
Regional Cancer Centre
Regional Cancer Centre
Regional Cancer Centre
Regional Cancer Centre
Regional Cancer Centre
Regional Cancer Centre
Regional Cancer Centre
Regional Cancer Centre
Regional Cancer Centre
Regional Cancer Centre
Regional Cancer Centre
Regional Cancer Centre
Regional Cancer Centre
Regional Cancer Centre
Regional Cancer Centre
Regional Cancer Centre
Regional Cancer Centre
Regional Cancer Centre
Regional Cancer Centre
Regional Cancer Centre
Regional Cancer Centre
Regional Cancer Centre
Regional Cancer Centre
Regional Cancer Centre
Regional Cancer Centre
Regional Cancer Centre
Regional Cancer Centre
Regional Cancer Centre
Regional Cancer Centre
Regional Cancer Centre
Regional Cancer Centre
Regional Cancer Centre
Regional Cancer Centre
Regional Cancer Centre
Regional Cancer Centre
Regional Cancer Centre
Regional Cancer Centre
Regional Cancer Centre
Regional Cancer Centre
Regional Cancer Centre
Regional Cancer Centre
Regional Cancer Centre
Regional Cancer Centre
Regional Cancer Centre

Regional Cancer Centre
Regional Cancer Centre
Regional Cancer Centre
Regional Cancer Centre
Regional Cancer Centre
Regional Cancer Centre
Regional Cancer Centre
Regional Cancer Centre
Regional Cancer Centre
Regional Cancer Centre
Regional Cancer Centre
Regional Cancer Centre
Regional Cancer Centre
Regional Cancer Centre
Regional Cancer Centre
Regional Cancer Centre
Regional Cancer Centre
Regional Cancer Centre
Regional Cancer Centre
Regional Cancer Centre
Regional Cancer Centre
Regional Cancer Centre
Regional Cancer Centre
Regional Cancer Centre
Regional Cancer Centre
Regional Cancer Centre
Regional Cancer Centre
Regional Cancer Centre
Regional Cancer Centre
Regional Cancer Centre
Regional Cancer Centre
Regional Cancer Centre
Regional Cancer Centre
Regional Cancer Centre
Regional Cancer Centre
Regional Cancer Centre
Regional Cancer Centre
Regional Cancer Centre
Regional Cancer Centre
Regional Cancer Centre
Regional Cancer Centre
Regional Cancer Centre
Regional Cancer Centre
Regional Cancer Centre
Regional Cancer Centre
Regional Cancer Centre
Regional Cancer Centre
Regional Cancer Centre
Regional Cancer Centre
Regional Cancer Centre

Regional Cancer Centre
Regional Cancer Centre
Regional Cancer Centre
Regional Cancer Centre
Regional Cancer Centre
Regional Cancer Centre
Regional Cancer Centre
Regional Cancer Centre
Regional Cancer Centre
Regional Cancer Centre
Regional Cancer Centre
Regional Cancer Centre
Regional Cancer Centre
Regional Cancer Centre
Regional Cancer Centre
Regional Cancer Centre
Regional Cancer Centre
Regional Cancer Centre
Regional Cancer Centre
Regional Cancer Centre
Regional Cancer Centre
Regional Cancer Centre
Regional Cancer Centre
Regional Cancer Centre
Regional Cancer Centre
Regional Cancer Centre
Regional Cancer Centre
Regional Cancer Centre
Regional Cancer Centre
Regional Cancer Centre
Regional Cancer Centre
Regional Cancer Centre
Regional Cancer Centre
Regional Cancer Centre
Regional Cancer Centre
Regional Cancer Centre
Regional Cancer Centre
Regional Cancer Centre
Regional Cancer Centre
National Cancer Centre
National Cancer Centre
National Cancer Centre
National Cancer Centre
National Cancer Centre
National Cancer Centre
National Cancer Centre
National Cancer Centre
National Cancer Centre
National Cancer Centre
National Cancer Centre

National Cancer Centre
National Cancer Centre
National Cancer Centre
Regional Cancer Centre
National Cancer Centre
Regional Cancer Centre
Regional Cancer Centre
Regional Cancer Centre
Regional Cancer Centre
Regional Cancer Centre
National Cancer Centre
National Cancer Centre
National Cancer Centre
Regional Cancer Centre
National Cancer Centre
National Cancer Centre
National Cancer Centre
National Cancer Centre
National Cancer Centre
National Cancer Centre
Regional Cancer Centre
National Cancer Centre
National Cancer Centre
National Cancer Centre
National Cancer Centre
National Cancer Centre
Regional Cancer Centre
Regional Cancer Centre
Regional Cancer Centre
Regional Cancer Centre
Regional Cancer Centre
Regional Cancer Centre
Regional Cancer Centre
National Cancer Centre
Regional Cancer Centre
Regional Cancer Centre
Regional Cancer Centre
National Cancer Centre
Regional Cancer Centre
National Cancer Centre
Regional Cancer Centre
Regional Cancer Centre
Regional Cancer Centre
Regional Cancer Centre
Regional Cancer Centre
Regional Cancer Centre
Regional Cancer Centre
Regional Cancer Centre
Regional Cancer Centre
Regional Cancer Centre

Regional Cancer Centre
Regional Cancer Centre
Regional Cancer Centre
Regional Cancer Centre
Regional Cancer Centre
Regional Cancer Centre
Regional Cancer Centre
Regional Cancer Centre
Regional Cancer Centre
Regional Cancer Centre
Regional Cancer Centre
Regional Cancer Centre
Regional Cancer Centre
Regional Cancer Centre
Regional Cancer Centre
Regional Cancer Centre
Regional Cancer Centre
Regional Cancer Centre
Regional Cancer Centre
Regional Cancer Centre
Regional Cancer Centre
Regional Cancer Centre
Regional Cancer Centre
Regional Cancer Centre
Regional Cancer Centre
Regional Cancer Centre
Regional Cancer Centre
Regional Cancer Centre
Regional Cancer Centre
Regional Cancer Centre
Regional Cancer Centre
Regional Cancer Centre
Regional Cancer Centre
Regional Cancer Centre
Regional Cancer Centre
Regional Cancer Centre
Regional Cancer Centre
Regional Cancer Centre
Regional Cancer Centre
Regional Cancer Centre
Regional Cancer Centre
Regional Cancer Centre
Regional Cancer Centre
Regional Cancer Centre
Regional Cancer Centre
Regional Cancer Centre
Regional Cancer Centre
Regional Cancer Centre
Regional Cancer Centre
Regional Cancer Centre

Regional Cancer Centre
Regional Cancer Centre
Regional Cancer Centre
Regional Cancer Centre
Regional Cancer Centre
Regional Cancer Centre
Regional Cancer Centre
Regional Cancer Centre
Regional Cancer Centre
Regional Cancer Centre
Regional Cancer Centre
Regional Cancer Centre
National Cancer Centre
National Cancer Centre
National Cancer Centre
National Cancer Centre
National Cancer Centre
National Cancer Centre
National Cancer Centre
National Cancer Centre
National Cancer Centre
National Cancer Centre
National Cancer Centre
National Cancer Centre
National Cancer Centre
National Cancer Centre
National Cancer Centre
National Cancer Centre
Regional Cancer Centre
Regional Cancer Centre
Regional Cancer Centre
Regional Cancer Centre
Regional Cancer Centre
Regional Cancer Centre
Regional Cancer Centre
Regional Cancer Centre
Regional Cancer Centre
Regional Cancer Centre
Regional Cancer Centre
Regional Cancer Centre
Regional Cancer Centre
Regional Cancer Centre
Regional Cancer Centre
Regional Cancer Centre
Regional Cancer Centre
Regional Cancer Centre
Regional Cancer Centre
Regional Cancer Centre
Regional Cancer Centre
Regional Cancer Centre

Regional Cancer Centre
Regional Cancer Centre
Regional Cancer Centre
Regional Cancer Centre
Regional Cancer Centre
Regional Cancer Centre
Regional Cancer Centre
Regional Cancer Centre
Regional Cancer Centre
Regional Cancer Centre
Regional Cancer Centre
Regional Cancer Centre
Regional Cancer Centre
Regional Cancer Centre
Regional Cancer Centre
Regional Cancer Centre
Regional Cancer Centre
Regional Cancer Centre
Regional Cancer Centre
Regional Cancer Centre
Regional Cancer Centre
Regional Cancer Centre
Regional Cancer Centre
Regional Cancer Centre
Regional Cancer Centre
Regional Cancer Centre
Regional Cancer Centre
Regional Cancer Centre
Regional Cancer Centre
Regional Cancer Centre
Regional Cancer Centre
Regional Cancer Centre
Regional Cancer Centre
Regional Cancer Centre
Regional Cancer Centre
Regional Cancer Centre
Regional Cancer Centre
Regional Cancer Centre
Regional Cancer Centre
Regional Cancer Centre
Regional Cancer Centre
Regional Cancer Centre
Regional Cancer Centre
Regional Cancer Centre
Regional Cancer Centre
Regional Cancer Centre
Regional Cancer Centre
Regional Cancer Centre
Regional Cancer Centre
Regional Cancer Centre

Regional Cancer Centre
Regional Cancer Centre
Regional Cancer Centre
Regional Cancer Centre
Regional Cancer Centre
Regional Cancer Centre
Regional Cancer Centre
Regional Cancer Centre
Regional Cancer Centre
Regional Cancer Centre
Regional Cancer Centre
Regional Cancer Centre
Regional Cancer Centre
Regional Cancer Centre
Regional Cancer Centre
Regional Cancer Centre
Regional Cancer Centre
Regional Cancer Centre
Regional Cancer Centre
Regional Cancer Centre
Regional Cancer Centre
Regional Cancer Centre
Regional Cancer Centre
Regional Cancer Centre
Regional Cancer Centre
Regional Cancer Centre
Regional Cancer Centre
Regional Cancer Centre
Regional Cancer Centre
Regional Cancer Centre
Regional Cancer Centre
Regional Cancer Centre
Regional Cancer Centre
Regional Cancer Centre
Regional Cancer Centre
Regional Cancer Centre
Regional Cancer Centre
Regional Cancer Centre
Regional Cancer Centre
Regional Cancer Centre
Regional Cancer Centre
Regional Cancer Centre
Regional Cancer Centre
Regional Cancer Centre
Regional Cancer Centre
Regional Cancer Centre
Regional Cancer Centre
Regional Cancer Centre
Regional Cancer Centre
Regional Cancer Centre

Regional Cancer Centre
Regional Cancer Centre
Regional Cancer Centre
Regional Cancer Centre
Regional Cancer Centre
Regional Cancer Centre
Regional Cancer Centre
Regional Cancer Centre
Regional Cancer Centre
Regional Cancer Centre
Regional Cancer Centre
Regional Cancer Centre
Regional Cancer Centre
Regional Cancer Centre
Regional Cancer Centre
Regional Cancer Centre
Regional Cancer Centre
Regional Cancer Centre
Regional Cancer Centre
Regional Cancer Centre
Regional Cancer Centre
Regional Cancer Centre
Regional Cancer Centre
Regional Cancer Centre
Regional Cancer Centre
Regional Cancer Centre
Regional Cancer Centre
Regional Cancer Centre
Regional Cancer Centre
Regional Cancer Centre
Regional Cancer Centre
Regional Cancer Centre
Regional Cancer Centre
Regional Cancer Centre
Regional Cancer Centre
Regional Cancer Centre
Regional Cancer Centre
Regional Cancer Centre
Regional Cancer Centre
Regional Cancer Centre
Regional Cancer Centre
Regional Cancer Centre
Regional Cancer Centre
Regional Cancer Centre
Regional Cancer Centre
Regional Cancer Centre
Regional Cancer Centre
Regional Cancer Centre
Regional Cancer Centre
Regional Cancer Centre

Regional Cancer Centre
Regional Cancer Centre
Regional Cancer Centre
Regional Cancer Centre
Regional Cancer Centre
Regional Cancer Centre
Regional Cancer Centre
Regional Cancer Centre
Regional Cancer Centre
Regional Cancer Centre
Regional Cancer Centre
Regional Cancer Centre
Regional Cancer Centre
Regional Cancer Centre
Regional Cancer Centre
Regional Cancer Centre
Regional Cancer Centre
Regional Cancer Centre
Regional Cancer Centre
Regional Cancer Centre
Regional Cancer Centre
Regional Cancer Centre
Regional Cancer Centre
Regional Cancer Centre
Regional Cancer Centre
Regional Cancer Centre
Regional Cancer Centre
Regional Cancer Centre
Regional Cancer Centre
Regional Cancer Centre
Regional Cancer Centre
Regional Cancer Centre
Regional Cancer Centre
Regional Cancer Centre
Regional Cancer Centre
Regional Cancer Centre
Regional Cancer Centre
Regional Cancer Centre
Regional Cancer Centre
Regional Cancer Centre
Regional Cancer Centre
Regional Cancer Centre
National Cancer Centre
National Cancer Centre
National Cancer Centre
Regional Cancer Centre
National Cancer Centre
Regional Cancer Centre
National Cancer Centre
National Cancer Centre

National Cancer Centre
National Cancer Centre
Regional Cancer Centre
Regional Cancer Centre
Regional Cancer Centre
Regional Cancer Centre
Regional Cancer Centre
National Cancer Centre
National Cancer Centre
National Cancer Centre
National Cancer Centre
National Cancer Centre
Regional Cancer Centre
Regional Cancer Centre
Regional Cancer Centre
Regional Cancer Centre
Regional Cancer Centre
National Cancer Centre
National Cancer Centre
National Cancer Centre
Regional Cancer Centre
Regional Cancer Centre
Regional Cancer Centre
National Cancer Centre
National Cancer Centre
National Cancer Centre
Regional Cancer Centre
Regional Cancer Centre
Regional Cancer Centre
Regional Cancer Centre
Regional Cancer Centre
National Cancer Centre
National Cancer Centre
National Cancer Centre
Regional Cancer Centre
Regional Cancer Centre
Regional Cancer Centre
Regional Cancer Centre
Regional Cancer Centre
Regional Cancer Centre
Regional Cancer Centre
Regional Cancer Centre
National Cancer Centre
National Cancer Centre
National Cancer Centre
National Cancer Centre
National Cancer Centre
Regional Cancer Centre
Regional Cancer Centre
Regional Cancer Centre

Regional Cancer Centre
Regional Cancer Centre
National Cancer Centre
Regional Cancer Centre
National Cancer Centre
Regional Cancer Centre
National Cancer Centre
National Cancer Centre
Regional Cancer Centre
Regional Cancer Centre
Regional Cancer Centre
Regional Cancer Centre
Regional Cancer Centre
National Cancer Centre
National Cancer Centre
National Cancer Centre
Regional Cancer Centre
National Cancer Centre
Regional Cancer Centre
Regional Cancer Centre
Regional Cancer Centre
National Cancer Centre
National Cancer Centre
National Cancer Centre
Regional Cancer Centre
Regional Cancer Centre
Regional Cancer Centre
Regional Cancer Centre
Regional Cancer Centre
Regional Cancer Centre
Regional Cancer Centre
Regional Cancer Centre
Regional Cancer Centre
Regional Cancer Centre
Regional Cancer Centre
Regional Cancer Centre
National Cancer Centre
National Cancer Centre
Regional Cancer Centre
National Cancer Centre
National Cancer Centre
National Cancer Centre
National Cancer Centre
National Cancer Centre
National Cancer Centre
National Cancer Centre
National Cancer Centre
National Cancer Centre
National Cancer Centre
Regional Cancer Centre

Regional Cancer Centre
Regional Cancer Centre
Regional Cancer Centre
Regional Cancer Centre
Regional Cancer Centre
Regional Cancer Centre
Regional Cancer Centre
Regional Cancer Centre
National Cancer Centre
National Cancer Centre
National Cancer Centre
Regional Cancer Centre
Regional Cancer Centre
Regional Cancer Centre
Regional Cancer Centre
Regional Cancer Centre
Regional Cancer Centre
Regional Cancer Centre
National Cancer Centre
Regional Cancer Centre
National Cancer Centre
National Cancer Centre
National Cancer Centre
Regional Cancer Centre
Regional Cancer Centre
Regional Cancer Centre
Regional Cancer Centre
Regional Cancer Centre
National Cancer Centre
Regional Cancer Centre
National Cancer Centre
National Cancer Centre
Regional Cancer Centre
Regional Cancer Centre
Regional Cancer Centre
National Cancer Centre
Regional Cancer Centre
Regional Cancer Centre
National Cancer Centre
National Cancer Centre
National Cancer Centre
National Cancer Centre
National Cancer Centre
National Cancer Centre
Regional Cancer Centre
Regional Cancer Centre
Regional Cancer Centre
Regional Cancer Centre
Regional Cancer Centre
Regional Cancer Centre

Regional Cancer Centre
Regional Cancer Centre
Regional Cancer Centre
Regional Cancer Centre
Regional Cancer Centre
Regional Cancer Centre
Regional Cancer Centre
Regional Cancer Centre
Regional Cancer Centre
Regional Cancer Centre
Regional Cancer Centre
Regional Cancer Centre
Regional Cancer Centre
Regional Cancer Centre
Regional Cancer Centre
Regional Cancer Centre
Regional Cancer Centre
Regional Cancer Centre
Regional Cancer Centre
Regional Cancer Centre
Regional Cancer Centre
Regional Cancer Centre
Regional Cancer Centre
Regional Cancer Centre
Regional Cancer Centre
Regional Cancer Centre
Regional Cancer Centre
Regional Cancer Centre
Regional Cancer Centre
Regional Cancer Centre
Regional Cancer Centre
Regional Cancer Centre
Regional Cancer Centre
Regional Cancer Centre
Regional Cancer Centre
Regional Cancer Centre
Regional Cancer Centre
Regional Cancer Centre
Regional Cancer Centre
Regional Cancer Centre
Regional Cancer Centre
Regional Cancer Centre
Regional Cancer Centre
Regional Cancer Centre
Regional Cancer Centre
Regional Cancer Centre
Regional Cancer Centre
Regional Cancer Centre
Regional Cancer Centre
Regional Cancer Centre

Regional Cancer Centre
Regional Cancer Centre
Regional Cancer Centre
Regional Cancer Centre
Regional Cancer Centre
Regional Cancer Centre
Regional Cancer Centre
Regional Cancer Centre
Regional Cancer Centre
Regional Cancer Centre
Regional Cancer Centre
Regional Cancer Centre
Regional Cancer Centre
Regional Cancer Centre
Regional Cancer Centre
Regional Cancer Centre
Regional Cancer Centre
Regional Cancer Centre
Regional Cancer Centre
Regional Cancer Centre
Regional Cancer Centre
Regional Cancer Centre
Regional Cancer Centre
Regional Cancer Centre
Regional Cancer Centre
Regional Cancer Centre
Regional Cancer Centre
Regional Cancer Centre
Regional Cancer Centre
Regional Cancer Centre
Regional Cancer Centre
Regional Cancer Centre
Regional Cancer Centre
Regional Cancer Centre
Regional Cancer Centre
Regional Cancer Centre
Regional Cancer Centre
Regional Cancer Centre
Regional Cancer Centre
Regional Cancer Centre
Regional Cancer Centre
Regional Cancer Centre
Regional Cancer Centre
Regional Cancer Centre
Regional Cancer Centre
Regional Cancer Centre
Regional Cancer Centre
Regional Cancer Centre
Regional Cancer Centre
Regional Cancer Centre

Regional Cancer Centre
Regional Cancer Centre
Regional Cancer Centre
Regional Cancer Centre
Regional Cancer Centre
Regional Cancer Centre
Regional Cancer Centre
Regional Cancer Centre
Regional Cancer Centre
Regional Cancer Centre
Regional Cancer Centre
Regional Cancer Centre
Regional Cancer Centre
Regional Cancer Centre
Regional Cancer Centre
Regional Cancer Centre
Regional Cancer Centre
Regional Cancer Centre
Regional Cancer Centre
Regional Cancer Centre
Regional Cancer Centre
Regional Cancer Centre
Regional Cancer Centre
Regional Cancer Centre
Regional Cancer Centre
Regional Cancer Centre
Regional Cancer Centre
Regional Cancer Centre
Regional Cancer Centre
Regional Cancer Centre
Regional Cancer Centre
Regional Cancer Centre
Regional Cancer Centre
Regional Cancer Centre
Regional Cancer Centre
Regional Cancer Centre
Regional Cancer Centre
Regional Cancer Centre
Regional Cancer Centre
Regional Cancer Centre
Regional Cancer Centre
Regional Cancer Centre
Regional Cancer Centre
Regional Cancer Centre
Regional Cancer Centre
Regional Cancer Centre
Regional Cancer Centre
Regional Cancer Centre
Regional Cancer Centre
Regional Cancer Centre

Regional Cancer Centre
Regional Cancer Centre
Regional Cancer Centre
Regional Cancer Centre
Regional Cancer Centre
Regional Cancer Centre
Regional Cancer Centre
Regional Cancer Centre
Regional Cancer Centre
Regional Cancer Centre
Regional Cancer Centre
Regional Cancer Centre
Regional Cancer Centre
Regional Cancer Centre
Regional Cancer Centre
Regional Cancer Centre
Regional Cancer Centre
Regional Cancer Centre
Regional Cancer Centre
Regional Cancer Centre
Regional Cancer Centre
Regional Cancer Centre
Regional Cancer Centre
Regional Cancer Centre
Regional Cancer Centre
Regional Cancer Centre
Regional Cancer Centre
Regional Cancer Centre
Regional Cancer Centre
Regional Cancer Centre
Regional Cancer Centre
Regional Cancer Centre
Regional Cancer Centre
Regional Cancer Centre
Regional Cancer Centre
Regional Cancer Centre
Regional Cancer Centre
Regional Cancer Centre
Regional Cancer Centre
Regional Cancer Centre
Regional Cancer Centre
Regional Cancer Centre
Regional Cancer Centre
Regional Cancer Centre
Regional Cancer Centre
Regional Cancer Centre
Regional Cancer Centre
Regional Cancer Centre
Regional Cancer Centre
Regional Cancer Centre

Regional Cancer Centre
Regional Cancer Centre
Regional Cancer Centre
Regional Cancer Centre
Regional Cancer Centre
Regional Cancer Centre
Regional Cancer Centre
Regional Cancer Centre
Regional Cancer Centre
Regional Cancer Centre
Regional Cancer Centre
Regional Cancer Centre
Regional Cancer Centre
Regional Cancer Centre
National Cancer Centre
National Cancer Centre
National Cancer Centre
National Cancer Centre
National Cancer Centre
National Cancer Centre
National Cancer Centre
National Cancer Centre
National Cancer Centre
National Cancer Centre
National Cancer Centre
National Cancer Centre
National Cancer Centre
National Cancer Centre
National Cancer Centre
National Cancer Centre
National Cancer Centre
National Cancer Centre
National Cancer Centre
National Cancer Centre
National Cancer Centre
National Cancer Centre
National Cancer Centre
National Cancer Centre
National Cancer Centre
National Cancer Centre
National Cancer Centre
National Cancer Centre
National Cancer Centre
National Cancer Centre
National Cancer Centre
National Cancer Centre
National Cancer Centre
National Cancer Centre
National Cancer Centre
National Cancer Centre

National Cancer Centre
National Cancer Centre
National Cancer Centre
National Cancer Centre
National Cancer Centre
National Cancer Centre
National Cancer Centre
National Cancer Centre
National Cancer Centre
National Cancer Centre
National Cancer Centre
National Cancer Centre
National Cancer Centre
National Cancer Centre
National Cancer Centre
National Cancer Centre
National Cancer Centre
National Cancer Centre
National Cancer Centre
National Cancer Centre
National Cancer Centre
National Cancer Centre
National Cancer Centre
National Cancer Centre
National Cancer Centre
National Cancer Centre
National Cancer Centre
National Cancer Centre
National Cancer Centre
National Cancer Centre
National Cancer Centre
National Cancer Centre
National Cancer Centre
Regional Cancer Centre
Regional Cancer Centre
Regional Cancer Centre
Regional Cancer Centre
Regional Cancer Centre
Regional Cancer Centre
Regional Cancer Centre
Regional Cancer Centre
Regional Cancer Centre
Regional Cancer Centre
Regional Cancer Centre
Regional Cancer Centre
Regional Cancer Centre
Regional Cancer Centre
Regional Cancer Centre
Regional Cancer Centre
Regional Cancer Centre

Regional Cancer Centre
Regional Cancer Centre
Regional Cancer Centre
Regional Cancer Centre
Regional Cancer Centre
Regional Cancer Centre
Regional Cancer Centre
Regional Cancer Centre
Regional Cancer Centre
Regional Cancer Centre
Regional Cancer Centre
Regional Cancer Centre
Regional Cancer Centre
Regional Cancer Centre
Regional Cancer Centre
Regional Cancer Centre
Regional Cancer Centre
Regional Cancer Centre
Regional Cancer Centre
Regional Cancer Centre
Regional Cancer Centre
Regional Cancer Centre
Regional Cancer Centre
Regional Cancer Centre
Regional Cancer Centre
Regional Cancer Centre
Regional Cancer Centre
Regional Cancer Centre
Regional Cancer Centre
Regional Cancer Centre
Regional Cancer Centre
Regional Cancer Centre
Regional Cancer Centre
Regional Cancer Centre
Regional Cancer Centre
Regional Cancer Centre
Regional Cancer Centre
Regional Cancer Centre
Regional Cancer Centre
Regional Cancer Centre
Regional Cancer Centre
Regional Cancer Centre
Regional Cancer Centre
Regional Cancer Centre
Regional Cancer Centre
Regional Cancer Centre
Regional Cancer Centre
Regional Cancer Centre
Regional Cancer Centre
Regional Cancer Centre

Regional Cancer Centre
Regional Cancer Centre
Regional Cancer Centre
Regional Cancer Centre
Regional Cancer Centre
Regional Cancer Centre
Regional Cancer Centre
Regional Cancer Centre
Regional Cancer Centre
Regional Cancer Centre
Regional Cancer Centre
Regional Cancer Centre
Regional Cancer Centre
Regional Cancer Centre
Regional Cancer Centre
Regional Cancer Centre
Regional Cancer Centre
Regional Cancer Centre
Regional Cancer Centre
Regional Cancer Centre
Regional Cancer Centre
Regional Cancer Centre
Regional Cancer Centre
Regional Cancer Centre
Regional Cancer Centre
Regional Cancer Centre
Regional Cancer Centre
Regional Cancer Centre
Regional Cancer Centre
Regional Cancer Centre
Regional Cancer Centre
Regional Cancer Centre
Regional Cancer Centre
Regional Cancer Centre
Regional Cancer Centre
Regional Cancer Centre
Regional Cancer Centre
Regional Cancer Centre
Regional Cancer Centre
Regional Cancer Centre
Regional Cancer Centre
Regional Cancer Centre
Regional Cancer Centre
Regional Cancer Centre
Regional Cancer Centre
Regional Cancer Centre
Regional Cancer Centre
Regional Cancer Centre
Regional Cancer Centre
Regional Cancer Centre

Regional Cancer Centre
Regional Cancer Centre
Regional Cancer Centre
Regional Cancer Centre
Regional Cancer Centre
Regional Cancer Centre
Regional Cancer Centre
Regional Cancer Centre
Regional Cancer Centre
Regional Cancer Centre
Regional Cancer Centre
Regional Cancer Centre
Regional Cancer Centre
Regional Cancer Centre
Regional Cancer Centre
Regional Cancer Centre
Regional Cancer Centre
Regional Cancer Centre
Regional Cancer Centre
Regional Cancer Centre
Regional Cancer Centre
National Cancer Centre
National Cancer Centre
National Cancer Centre
National Cancer Centre
National Cancer Centre
National Cancer Centre
National Cancer Centre
National Cancer Centre
National Cancer Centre
National Cancer Centre
National Cancer Centre
National Cancer Centre
National Cancer Centre
National Cancer Centre
National Cancer Centre
National Cancer Centre
National Cancer Centre
National Cancer Centre
National Cancer Centre
National Cancer Centre
National Cancer Centre
National Cancer Centre
National Cancer Centre
National Cancer Centre
National Cancer Centre
National Cancer Centre
National Cancer Centre
National Cancer Centre
National Cancer Centre

National Cancer Centre
National Cancer Centre
National Cancer Centre
National Cancer Centre
National Cancer Centre
National Cancer Centre
National Cancer Centre
National Cancer Centre
National Cancer Centre
National Cancer Centre
National Cancer Centre
National Cancer Centre
National Cancer Centre
National Cancer Centre
National Cancer Centre
National Cancer Centre
National Cancer Centre
National Cancer Centre
National Cancer Centre
National Cancer Centre
National Cancer Centre
National Cancer Centre
National Cancer Centre
National Cancer Centre
National Cancer Centre
National Cancer Centre
National Cancer Centre
National Cancer Centre
National Cancer Centre
National Cancer Centre
National Cancer Centre
National Cancer Centre
National Cancer Centre
National Cancer Centre
National Cancer Centre
National Cancer Centre
National Cancer Centre
National Cancer Centre
National Cancer Centre
National Cancer Centre
National Cancer Centre
National Cancer Centre
National Cancer Centre
National Cancer Centre
National Cancer Centre
National Cancer Centre
National Cancer Centre
National Cancer Centre
National Cancer Centre
National Cancer Centre

National Cancer Centre
National Cancer Centre
National Cancer Centre
National Cancer Centre
National Cancer Centre
National Cancer Centre
National Cancer Centre
National Cancer Centre
National Cancer Centre
National Cancer Centre
National Cancer Centre
National Cancer Centre
National Cancer Centre
National Cancer Centre
National Cancer Centre
National Cancer Centre
National Cancer Centre
National Cancer Centre
National Cancer Centre
National Cancer Centre
National Cancer Centre
National Cancer Centre
National Cancer Centre
National Cancer Centre
National Cancer Centre
National Cancer Centre
National Cancer Centre
National Cancer Centre
National Cancer Centre
National Cancer Centre
National Cancer Centre
National Cancer Centre
National Cancer Centre
National Cancer Centre
National Cancer Centre
National Cancer Centre
National Cancer Centre
National Cancer Centre
National Cancer Centre
National Cancer Centre
National Cancer Centre
National Cancer Centre
National Cancer Centre
National Cancer Centre
National Cancer Centre
National Cancer Centre
National Cancer Centre
National Cancer Centre
National Cancer Centre
National Cancer Centre

National Cancer Centre
National Cancer Centre
National Cancer Centre
National Cancer Centre
National Cancer Centre
National Cancer Centre
National Cancer Centre
National Cancer Centre
National Cancer Centre
National Cancer Centre
National Cancer Centre
National Cancer Centre
National Cancer Centre
National Cancer Centre
National Cancer Centre
National Cancer Centre
National Cancer Centre
National Cancer Centre
National Cancer Centre
National Cancer Centre
National Cancer Centre
National Cancer Centre
National Cancer Centre
National Cancer Centre
National Cancer Centre
National Cancer Centre
National Cancer Centre
National Cancer Centre
National Cancer Centre
National Cancer Centre
National Cancer Centre
National Cancer Centre
National Cancer Centre
National Cancer Centre
National Cancer Centre
National Cancer Centre
National Cancer Centre
National Cancer Centre
National Cancer Centre
National Cancer Centre
National Cancer Centre
National Cancer Centre
National Cancer Centre
National Cancer Centre
National Cancer Centre
National Cancer Centre
National Cancer Centre
National Cancer Centre
National Cancer Centre
National Cancer Centre

National Cancer Centre
National Cancer Centre
National Cancer Centre
National Cancer Centre
National Cancer Centre
National Cancer Centre
National Cancer Centre
National Cancer Centre
National Cancer Centre
National Cancer Centre
National Cancer Centre
National Cancer Centre
National Cancer Centre
National Cancer Centre
National Cancer Centre
National Cancer Centre
National Cancer Centre
National Cancer Centre
National Cancer Centre
National Cancer Centre
National Cancer Centre
National Cancer Centre
National Cancer Centre
National Cancer Centre
National Cancer Centre
National Cancer Centre
National Cancer Centre
National Cancer Centre
National Cancer Centre
National Cancer Centre
National Cancer Centre
National Cancer Centre
National Cancer Centre
National Cancer Centre
National Cancer Centre
National Cancer Centre
National Cancer Centre
Regional Cancer Centre
Regional Cancer Centre
Regional Cancer Centre
Regional Cancer Centre
Regional Cancer Centre
Regional Cancer Centre
Regional Cancer Centre
Regional Cancer Centre
Regional Cancer Centre
Regional Cancer Centre
Regional Cancer Centre
Regional Cancer Centre
Regional Cancer Centre

Regional Cancer Centre
Regional Cancer Centre
Regional Cancer Centre
Regional Cancer Centre
Regional Cancer Centre
Regional Cancer Centre
Regional Cancer Centre
Regional Cancer Centre
Regional Cancer Centre
Regional Cancer Centre
Regional Cancer Centre
Regional Cancer Centre
Regional Cancer Centre
Regional Cancer Centre
Regional Cancer Centre
Regional Cancer Centre
Regional Cancer Centre
Regional Cancer Centre
Regional Cancer Centre
Regional Cancer Centre
Regional Cancer Centre
Regional Cancer Centre
Regional Cancer Centre
Regional Cancer Centre
Regional Cancer Centre
Regional Cancer Centre
Regional Cancer Centre
Regional Cancer Centre
Regional Cancer Centre
Regional Cancer Centre
Regional Cancer Centre
Regional Cancer Centre
Regional Cancer Centre
Regional Cancer Centre
Regional Cancer Centre
Regional Cancer Centre
Regional Cancer Centre
Regional Cancer Centre
Regional Cancer Centre
Regional Cancer Centre
Regional Cancer Centre
Regional Cancer Centre
Regional Cancer Centre
Regional Cancer Centre
Regional Cancer Centre
Regional Cancer Centre
Regional Cancer Centre
Regional Cancer Centre
Regional Cancer Centre
Regional Cancer Centre

Regional Cancer Centre
Regional Cancer Centre
Regional Cancer Centre
Regional Cancer Centre
Regional Cancer Centre
Regional Cancer Centre
Regional Cancer Centre
Regional Cancer Centre
Regional Cancer Centre
Regional Cancer Centre
Regional Cancer Centre
Regional Cancer Centre
Regional Cancer Centre
Regional Cancer Centre
Regional Cancer Centre
Regional Cancer Centre
Regional Cancer Centre
Regional Cancer Centre
Regional Cancer Centre
Regional Cancer Centre
Regional Cancer Centre
Regional Cancer Centre
Regional Cancer Centre
Regional Cancer Centre
Regional Cancer Centre
Regional Cancer Centre
Regional Cancer Centre
Regional Cancer Centre
Regional Cancer Centre
Regional Cancer Centre
Regional Cancer Centre
Regional Cancer Centre
Regional Cancer Centre
Regional Cancer Centre
Regional Cancer Centre
Regional Cancer Centre
Regional Cancer Centre
Regional Cancer Centre
Regional Cancer Centre
Regional Cancer Centre
Regional Cancer Centre
Regional Cancer Centre
Regional Cancer Centre
Regional Cancer Centre
Regional Cancer Centre
Regional Cancer Centre
Regional Cancer Centre
Regional Cancer Centre
Regional Cancer Centre
Regional Cancer Centre

Regional Cancer Centre
Regional Cancer Centre
Regional Cancer Centre
Regional Cancer Centre
Regional Cancer Centre
Regional Cancer Centre
Regional Cancer Centre
Regional Cancer Centre
Regional Cancer Centre
Regional Cancer Centre
Regional Cancer Centre
Regional Cancer Centre
Regional Cancer Centre
Regional Cancer Centre
Regional Cancer Centre
Regional Cancer Centre
Regional Cancer Centre
Regional Cancer Centre
Regional Cancer Centre
Regional Cancer Centre
Regional Cancer Centre
Regional Cancer Centre
Regional Cancer Centre
Regional Cancer Centre
Regional Cancer Centre
Regional Cancer Centre
Regional Cancer Centre
Regional Cancer Centre
Regional Cancer Centre
Regional Cancer Centre
Regional Cancer Centre
Regional Cancer Centre
Regional Cancer Centre
Regional Cancer Centre
Regional Cancer Centre
Regional Cancer Centre
Regional Cancer Centre
Regional Cancer Centre
Regional Cancer Centre
Regional Cancer Centre
Regional Cancer Centre
Regional Cancer Centre
Regional Cancer Centre
Regional Cancer Centre
Regional Cancer Centre
Regional Cancer Centre
Regional Cancer Centre
Regional Cancer Centre
Regional Cancer Centre
Regional Cancer Centre

Regional Cancer Centre
Regional Cancer Centre
Regional Cancer Centre
Regional Cancer Centre
Regional Cancer Centre
Regional Cancer Centre
Regional Cancer Centre
Regional Cancer Centre
Regional Cancer Centre
Regional Cancer Centre
Regional Cancer Centre
Regional Cancer Centre
Regional Cancer Centre
Regional Cancer Centre
Regional Cancer Centre
Regional Cancer Centre
Regional Cancer Centre
Regional Cancer Centre
Regional Cancer Centre
Regional Cancer Centre
Regional Cancer Centre
Regional Cancer Centre
Regional Cancer Centre
Regional Cancer Centre
Regional Cancer Centre
Regional Cancer Centre
Regional Cancer Centre
Regional Cancer Centre
Regional Cancer Centre
Regional Cancer Centre
Regional Cancer Centre
Regional Cancer Centre
Regional Cancer Centre
Regional Cancer Centre
Regional Cancer Centre
Regional Cancer Centre
Regional Cancer Centre
Regional Cancer Centre
Regional Cancer Centre
Regional Cancer Centre
Regional Cancer Centre
Regional Cancer Centre
Regional Cancer Centre
Regional Cancer Centre
Regional Cancer Centre
Regional Cancer Centre
Regional Cancer Centre
Regional Cancer Centre
Regional Cancer Centre
Regional Cancer Centre

Regional Cancer Centre
Regional Cancer Centre
Regional Cancer Centre
Regional Cancer Centre
Regional Cancer Centre
Regional Cancer Centre
Regional Cancer Centre
Regional Cancer Centre
Regional Cancer Centre
Regional Cancer Centre
Regional Cancer Centre
Regional Cancer Centre
Regional Cancer Centre
Regional Cancer Centre
Regional Cancer Centre
Regional Cancer Centre
Regional Cancer Centre
Regional Cancer Centre
Regional Cancer Centre
Regional Cancer Centre
Regional Cancer Centre
Regional Cancer Centre
Regional Cancer Centre
Regional Cancer Centre
Regional Cancer Centre
Regional Cancer Centre
Regional Cancer Centre
Regional Cancer Centre
Regional Cancer Centre
Regional Cancer Centre
Regional Cancer Centre
Regional Cancer Centre
Regional Cancer Centre
Regional Cancer Centre
Regional Cancer Centre
Regional Cancer Centre
Regional Cancer Centre
Regional Cancer Centre
Regional Cancer Centre
Regional Cancer Centre
Regional Cancer Centre
Regional Cancer Centre
Regional Cancer Centre
Regional Cancer Centre
Regional Cancer Centre
Regional Cancer Centre
Regional Cancer Centre
Regional Cancer Centre
Regional Cancer Centre
Regional Cancer Centre

Regional Cancer Centre
Regional Cancer Centre
Regional Cancer Centre
Regional Cancer Centre
Regional Cancer Centre
Regional Cancer Centre
Regional Cancer Centre
Regional Cancer Centre
Regional Cancer Centre
Regional Cancer Centre
Regional Cancer Centre
Regional Cancer Centre
Regional Cancer Centre
Regional Cancer Centre
Regional Cancer Centre
Regional Cancer Centre
Regional Cancer Centre
Regional Cancer Centre
Regional Cancer Centre
Regional Cancer Centre
Regional Cancer Centre
Regional Cancer Centre
Regional Cancer Centre
Regional Cancer Centre
Regional Cancer Centre
Regional Cancer Centre
Regional Cancer Centre
Regional Cancer Centre
Regional Cancer Centre
Regional Cancer Centre
Regional Cancer Centre
Regional Cancer Centre
Regional Cancer Centre
Regional Cancer Centre
Regional Cancer Centre
Regional Cancer Centre
Regional Cancer Centre
Regional Cancer Centre
Regional Cancer Centre
Regional Cancer Centre
Regional Cancer Centre
Regional Cancer Centre
Regional Cancer Centre
Regional Cancer Centre
Regional Cancer Centre
Regional Cancer Centre
Regional Cancer Centre
Regional Cancer Centre
Regional Cancer Centre
Regional Cancer Centre

Regional Cancer Centre
Regional Cancer Centre
Regional Cancer Centre
Regional Cancer Centre
Regional Cancer Centre
Regional Cancer Centre
Regional Cancer Centre
Regional Cancer Centre
Regional Cancer Centre
Regional Cancer Centre
Regional Cancer Centre
Regional Cancer Centre
Regional Cancer Centre
Regional Cancer Centre
Regional Cancer Centre
Regional Cancer Centre
Regional Cancer Centre
Regional Cancer Centre
Regional Cancer Centre
Regional Cancer Centre
Regional Cancer Centre
Regional Cancer Centre
Regional Cancer Centre
Regional Cancer Centre
Regional Cancer Centre
Regional Cancer Centre
Regional Cancer Centre
Regional Cancer Centre
Regional Cancer Centre
Regional Cancer Centre
Regional Cancer Centre
Regional Cancer Centre
Regional Cancer Centre
Regional Cancer Centre
Regional Cancer Centre
Regional Cancer Centre
Regional Cancer Centre
Regional Cancer Centre
Regional Cancer Centre
Regional Cancer Centre
Regional Cancer Centre
Regional Cancer Centre
Regional Cancer Centre
Regional Cancer Centre
Regional Cancer Centre
National Cancer Centre
Regional Cancer Centre
Regional Cancer Centre
Regional Cancer Centre
Regional Cancer Centre

Regional Cancer Centre
Regional Cancer Centre
Regional Cancer Centre
Regional Cancer Centre
Regional Cancer Centre
Regional Cancer Centre
Regional Cancer Centre
Regional Cancer Centre
National Cancer Centre
National Cancer Centre
Regional Cancer Centre
Regional Cancer Centre
Regional Cancer Centre
National Cancer Centre
National Cancer Centre
Regional Cancer Centre
Regional Cancer Centre
National Cancer Centre
Regional Cancer Centre
Regional Cancer Centre
Regional Cancer Centre
Regional Cancer Centre
Regional Cancer Centre
Regional Cancer Centre
Regional Cancer Centre
National Cancer Centre
National Cancer Centre
National Cancer Centre
Regional Cancer Centre
Regional Cancer Centre
Regional Cancer Centre
Regional Cancer Centre
Regional Cancer Centre
Regional Cancer Centre
Regional Cancer Centre
Regional Cancer Centre
National Cancer Centre
Regional Cancer Centre
Regional Cancer Centre
Regional Cancer Centre
National Cancer Centre
National Cancer Centre
Regional Cancer Centre
National Cancer Centre
National Cancer Centre
Regional Cancer Centre
Regional Cancer Centre
Regional Cancer Centre
Regional Cancer Centre
Regional Cancer Centre

Regional Cancer Centre
Regional Cancer Centre
Regional Cancer Centre
Regional Cancer Centre
Regional Cancer Centre
Regional Cancer Centre
Regional Cancer Centre
National Cancer Centre
National Cancer Centre
Regional Cancer Centre
Regional Cancer Centre
National Cancer Centre
National Cancer Centre
Regional Cancer Centre
Regional Cancer Centre
Regional Cancer Centre
Regional Cancer Centre
Regional Cancer Centre
Regional Cancer Centre
Regional Cancer Centre
National Cancer Centre
Regional Cancer Centre
National Cancer Centre
Regional Cancer Centre
Regional Cancer Centre
Regional Cancer Centre
Regional Cancer Centre
Regional Cancer Centre
National Cancer Centre
Regional Cancer Centre
Regional Cancer Centre
National Cancer Centre
Regional Cancer Centre
National Cancer Centre
National Cancer Centre
Regional Cancer Centre
Regional Cancer Centre
Regional Cancer Centre
Regional Cancer Centre
National Cancer Centre
National Cancer Centre
Regional Cancer Centre
Regional Cancer Centre
National Cancer Centre
Regional Cancer Centre
National Cancer Centre
Regional Cancer Centre
Regional Cancer Centre
Regional Cancer Centre
Regional Cancer Centre

Regional Cancer Centre
Regional Cancer Centre
National Cancer Centre
National Cancer Centre
Regional Cancer Centre
Regional Cancer Centre
National Cancer Centre
National Cancer Centre
Regional Cancer Centre
Regional Cancer Centre
Regional Cancer Centre
Regional Cancer Centre
Regional Cancer Centre
Regional Cancer Centre
National Cancer Centre
National Cancer Centre
Regional Cancer Centre
Regional Cancer Centre
Regional Cancer Centre
National Cancer Centre
Regional Cancer Centre
Regional Cancer Centre
Regional Cancer Centre
Regional Cancer Centre
Regional Cancer Centre
National Cancer Centre
Regional Cancer Centre
Regional Cancer Centre
Regional Cancer Centre
Regional Cancer Centre
Regional Cancer Centre
Regional Cancer Centre
Regional Cancer Centre
Regional Cancer Centre
Regional Cancer Centre
Regional Cancer Centre
National Cancer Centre
Regional Cancer Centre
Regional Cancer Centre
Regional Cancer Centre
Regional Cancer Centre
Regional Cancer Centre
Regional Cancer Centre
Regional Cancer Centre
Regional Cancer Centre
National Cancer Centre
National Cancer Centre
Regional Cancer Centre
Regional Cancer Centre
Regional Cancer Centre

Regional Cancer Centre
Regional Cancer Centre
Regional Cancer Centre
National Cancer Centre
National Cancer Centre
National Cancer Centre
National Cancer Centre
National Cancer Centre
National Cancer Centre
Regional Cancer Centre
Regional Cancer Centre
Regional Cancer Centre
Regional Cancer Centre
Regional Cancer Centre
Regional Cancer Centre
Regional Cancer Centre
Regional Cancer Centre
National Cancer Centre
Regional Cancer Centre
National Cancer Centre
Regional Cancer Centre
Regional Cancer Centre
Regional Cancer Centre
Regional Cancer Centre
Regional Cancer Centre
Regional Cancer Centre
Regional Cancer Centre
Regional Cancer Centre
Regional Cancer Centre
Regional Cancer Centre
National Cancer Centre
Regional Cancer Centre
National Cancer Centre
Regional Cancer Centre
National Cancer Centre
Regional Cancer Centre
Regional Cancer Centre
National Cancer Centre
National Cancer Centre
Regional Cancer Centre
National Cancer Centre
Regional Cancer Centre
National Cancer Centre
National Cancer Centre
Regional Cancer Centre
Regional Cancer Centre
Regional Cancer Centre
Regional Cancer Centre
Regional Cancer Centre
Regional Cancer Centre

Regional Cancer Centre
National Cancer Centre
Regional Cancer Centre
Regional Cancer Centre
Regional Cancer Centre
National Cancer Centre
Regional Cancer Centre
Regional Cancer Centre
National Cancer Centre
National Cancer Centre
Regional Cancer Centre
Regional Cancer Centre
Regional Cancer Centre
Regional Cancer Centre
National Cancer Centre
National Cancer Centre
Regional Cancer Centre
Regional Cancer Centre
Regional Cancer Centre
Regional Cancer Centre
Regional Cancer Centre
National Cancer Centre
National Cancer Centre
Regional Cancer Centre
Regional Cancer Centre
National Cancer Centre
National Cancer Centre
Regional Cancer Centre
Regional Cancer Centre
Regional Cancer Centre
Regional Cancer Centre
Regional Cancer Centre
Regional Cancer Centre
Regional Cancer Centre
Regional Cancer Centre
National Cancer Centre
National Cancer Centre
National Cancer Centre
Regional Cancer Centre
Regional Cancer Centre
Regional Cancer Centre
National Cancer Centre
Regional Cancer Centre
Regional Cancer Centre
Regional Cancer Centre
Regional Cancer Centre
Regional Cancer Centre
Regional Cancer Centre
Regional Cancer Centre
Regional Cancer Centre

National Cancer Centre
National Cancer Centre
Regional Cancer Centre
Regional Cancer Centre
Regional Cancer Centre
Regional Cancer Centre
Regional Cancer Centre
Regional Cancer Centre
Regional Cancer Centre
Regional Cancer Centre
Regional Cancer Centre
Regional Cancer Centre
Regional Cancer Centre
Regional Cancer Centre
National Cancer Centre
National Cancer Centre
Regional Cancer Centre
Regional Cancer Centre
Regional Cancer Centre
Regional Cancer Centre
Regional Cancer Centre
Regional Cancer Centre
Regional Cancer Centre
Regional Cancer Centre
Regional Cancer Centre
Regional Cancer Centre
Regional Cancer Centre
Regional Cancer Centre
National Cancer Centre
National Cancer Centre
Regional Cancer Centre
Regional Cancer Centre
Regional Cancer Centre
Regional Cancer Centre
Regional Cancer Centre
Regional Cancer Centre
Regional Cancer Centre
Regional Cancer Centre
National Cancer Centre
Regional Cancer Centre
National Cancer Centre
Regional Cancer Centre
National Cancer Centre
National Cancer Centre
Regional Cancer Centre
Regional Cancer Centre
National Cancer Centre
National Cancer Centre
Regional Cancer Centre
Regional Cancer Centre

Regional Cancer Centre
Regional Cancer Centre
Regional Cancer Centre
Regional Cancer Centre
Regional Cancer Centre
Regional Cancer Centre
Regional Cancer Centre
Regional Cancer Centre
Regional Cancer Centre
Regional Cancer Centre
Regional Cancer Centre
Regional Cancer Centre
National Cancer Centre
Regional Cancer Centre
National Cancer Centre
Regional Cancer Centre
Regional Cancer Centre
Regional Cancer Centre
National Cancer Centre
National Cancer Centre
Regional Cancer Centre
Regional Cancer Centre
Regional Cancer Centre
Regional Cancer Centre
Regional Cancer Centre
Regional Cancer Centre
National Cancer Centre
National Cancer Centre
National Cancer Centre
Regional Cancer Centre
Regional Cancer Centre
National Cancer Centre
Regional Cancer Centre
National Cancer Centre
National Cancer Centre
Regional Cancer Centre
Regional Cancer Centre
National Cancer Centre
National Cancer Centre
Regional Cancer Centre
Regional Cancer Centre
Regional Cancer Centre
Regional Cancer Centre
Regional Cancer Centre
Regional Cancer Centre
Regional Cancer Centre
Regional Cancer Centre
Regional Cancer Centre
Regional Cancer Centre
Regional Cancer Centre

National Cancer Centre
Regional Cancer Centre
National Cancer Centre
Regional Cancer Centre
Regional Cancer Centre
Regional Cancer Centre
Regional Cancer Centre
Regional Cancer Centre
Regional Cancer Centre
Regional Cancer Centre
Regional Cancer Centre
National Cancer Centre
Regional Cancer Centre
National Cancer Centre
Regional Cancer Centre
Regional Cancer Centre
Regional Cancer Centre
Regional Cancer Centre
National Cancer Centre
National Cancer Centre
Regional Cancer Centre
Regional Cancer Centre
Regional Cancer Centre
Regional Cancer Centre
Regional Cancer Centre
Regional Cancer Centre
Regional Cancer Centre
Regional Cancer Centre
National Cancer Centre
National Cancer Centre
Regional Cancer Centre
National Cancer Centre
Regional Cancer Centre
National Cancer Centre
Regional Cancer Centre
Regional Cancer Centre
Regional Cancer Centre
Regional Cancer Centre
Regional Cancer Centre
Regional Cancer Centre
Regional Cancer Centre
Regional Cancer Centre
Regional Cancer Centre
Regional Cancer Centre
National Cancer Centre
National Cancer Centre
National Cancer Centre
Regional Cancer Centre
Regional Cancer Centre
Regional Cancer Centre

Regional Cancer Centre
Regional Cancer Centre
Regional Cancer Centre
Regional Cancer Centre
Regional Cancer Centre
Regional Cancer Centre
Regional Cancer Centre
Regional Cancer Centre
National Cancer Centre
National Cancer Centre
Regional Cancer Centre
Regional Cancer Centre
Regional Cancer Centre
Regional Cancer Centre
Regional Cancer Centre
Regional Cancer Centre
Regional Cancer Centre
Regional Cancer Centre
Regional Cancer Centre
Regional Cancer Centre
Regional Cancer Centre
Regional Cancer Centre
National Cancer Centre
National Cancer Centre
Regional Cancer Centre
Regional Cancer Centre
Regional Cancer Centre
Regional Cancer Centre
Regional Cancer Centre
Regional Cancer Centre
Regional Cancer Centre
Regional Cancer Centre
Regional Cancer Centre
National Cancer Centre
Regional Cancer Centre
Regional Cancer Centre
Regional Cancer Centre
Regional Cancer Centre
Regional Cancer Centre
Regional Cancer Centre
Regional Cancer Centre
Regional Cancer Centre
Regional Cancer Centre
Regional Cancer Centre
Regional Cancer Centre
Regional Cancer Centre
National Cancer Centre
National Cancer Centre
National Cancer Centre
Regional Cancer Centre

Regional Cancer Centre
National Cancer Centre
Regional Cancer Centre
Regional Cancer Centre
Regional Cancer Centre
Regional Cancer Centre
Regional Cancer Centre
Regional Cancer Centre
Regional Cancer Centre
Regional Cancer Centre
Regional Cancer Centre
National Cancer Centre
Regional Cancer Centre
Regional Cancer Centre
Regional Cancer Centre
Regional Cancer Centre
Regional Cancer Centre
Regional Cancer Centre
National Cancer Centre
Regional Cancer Centre
Regional Cancer Centre
National Cancer Centre
Regional Cancer Centre
Regional Cancer Centre
Regional Cancer Centre
Regional Cancer Centre
Regional Cancer Centre
Regional Cancer Centre
Regional Cancer Centre
Regional Cancer Centre
Regional Cancer Centre
Regional Cancer Centre
Regional Cancer Centre
Regional Cancer Centre
Regional Cancer Centre
Regional Cancer Centre
Regional Cancer Centre
National Cancer Centre
National Cancer Centre
Regional Cancer Centre
National Cancer Centre
Regional Cancer Centre
National Cancer Centre
Regional Cancer Centre
Regional Cancer Centre
National Cancer Centre
National Cancer Centre
Regional Cancer Centre
National Cancer Centre
National Cancer Centre

Regional Cancer Centre
Regional Cancer Centre
Regional Cancer Centre
Regional Cancer Centre
Regional Cancer Centre
Regional Cancer Centre
Regional Cancer Centre
Regional Cancer Centre
Regional Cancer Centre
Regional Cancer Centre
Regional Cancer Centre
Regional Cancer Centre
Regional Cancer Centre
National Cancer Centre
National Cancer Centre
Regional Cancer Centre
Regional Cancer Centre
Regional Cancer Centre
Regional Cancer Centre
Regional Cancer Centre
Regional Cancer Centre
Regional Cancer Centre
National Cancer Centre
Regional Cancer Centre
Regional Cancer Centre
Regional Cancer Centre
National Cancer Centre
Regional Cancer Centre
Regional Cancer Centre
Regional Cancer Centre
Regional Cancer Centre
Regional Cancer Centre
Regional Cancer Centre
National Cancer Centre
National Cancer Centre
Regional Cancer Centre
Regional Cancer Centre
National Cancer Centre
Regional Cancer Centre
National Cancer Centre
Regional Cancer Centre
Regional Cancer Centre
Regional Cancer Centre
Regional Cancer Centre
Regional Cancer Centre
Regional Cancer Centre
Regional Cancer Centre
Regional Cancer Centre
National Cancer Centre
National Cancer Centre

National Cancer Centre
National Cancer Centre
Regional Cancer Centre
Regional Cancer Centre
Regional Cancer Centre
Regional Cancer Centre
Regional Cancer Centre
Regional Cancer Centre
Regional Cancer Centre
Regional Cancer Centre
Regional Cancer Centre
Regional Cancer Centre
National Cancer Centre
Regional Cancer Centre
National Cancer Centre
Regional Cancer Centre
Regional Cancer Centre
Regional Cancer Centre
Regional Cancer Centre
Regional Cancer Centre
Regional Cancer Centre
Regional Cancer Centre
Regional Cancer Centre
Regional Cancer Centre
Regional Cancer Centre
Regional Cancer Centre
Regional Cancer Centre
Regional Cancer Centre
Regional Cancer Centre
Regional Cancer Centre
Regional Cancer Centre
Regional Cancer Centre
Regional Cancer Centre
National Cancer Centre
Regional Cancer Centre
National Cancer Centre
Regional Cancer Centre
Regional Cancer Centre
Regional Cancer Centre
Regional Cancer Centre
Regional Cancer Centre
Regional Cancer Centre
Regional Cancer Centre
Regional Cancer Centre
Regional Cancer Centre
Regional Cancer Centre
Regional Cancer Centre
Regional Cancer Centre
National Cancer Centre
Regional Cancer Centre

National Cancer Centre
Regional Cancer Centre
Regional Cancer Centre
Regional Cancer Centre
Regional Cancer Centre
Regional Cancer Centre
Regional Cancer Centre
Regional Cancer Centre
Regional Cancer Centre
Regional Cancer Centre
Regional Cancer Centre
Regional Cancer Centre
Regional Cancer Centre
National Cancer Centre
Regional Cancer Centre
National Cancer Centre
Regional Cancer Centre
National Cancer Centre
National Cancer Centre
Regional Cancer Centre
Regional Cancer Centre
National Cancer Centre
National Cancer Centre
Regional Cancer Centre
Regional Cancer Centre
Regional Cancer Centre
Regional Cancer Centre
Regional Cancer Centre
Regional Cancer Centre
Regional Cancer Centre
Regional Cancer Centre
Regional Cancer Centre
Regional Cancer Centre
Regional Cancer Centre
Regional Cancer Centre
Regional Cancer Centre
Regional Cancer Centre
Regional Cancer Centre
Regional Cancer Centre
Regional Cancer Centre
Regional Cancer Centre
Regional Cancer Centre
National Cancer Centre
Regional Cancer Centre
National Cancer Centre
Regional Cancer Centre
Regional Cancer Centre
Regional Cancer Centre
National Cancer Centre
National Cancer Centre

Regional Cancer Centre
Regional Cancer Centre
Regional Cancer Centre
Regional Cancer Centre
Regional Cancer Centre
National Cancer Centre
National Cancer Centre
Regional Cancer Centre
National Cancer Centre
Regional Cancer Centre
National Cancer Centre
Regional Cancer Centre
Regional Cancer Centre
Regional Cancer Centre
Regional Cancer Centre
National Cancer Centre
National Cancer Centre
Regional Cancer Centre
Regional Cancer Centre
National Cancer Centre
National Cancer Centre
Regional Cancer Centre
Regional Cancer Centre
Regional Cancer Centre
Regional Cancer Centre
Regional Cancer Centre
Regional Cancer Centre
Regional Cancer Centre
Regional Cancer Centre
Regional Cancer Centre
Regional Cancer Centre
Regional Cancer Centre
Regional Cancer Centre
National Cancer Centre
Regional Cancer Centre
Regional Cancer Centre
Regional Cancer Centre
Regional Cancer Centre
Regional Cancer Centre
Regional Cancer Centre
Regional Cancer Centre
Regional Cancer Centre
Regional Cancer Centre
Regional Cancer Centre
National Cancer Centre
Regional Cancer Centre
National Cancer Centre
National Cancer Centre
Regional Cancer Centre
Regional Cancer Centre

Regional Cancer Centre
Regional Cancer Centre
Regional Cancer Centre
National Cancer Centre
National Cancer Centre
Regional Cancer Centre
Regional Cancer Centre
Regional Cancer Centre
Regional Cancer Centre
Regional Cancer Centre
Regional Cancer Centre
National Cancer Centre
National Cancer Centre
Regional Cancer Centre
Regional Cancer Centre
National Cancer Centre
National Cancer Centre
Regional Cancer Centre
Regional Cancer Centre
Regional Cancer Centre
Regional Cancer Centre
Regional Cancer Centre
Regional Cancer Centre
Regional Cancer Centre
Regional Cancer Centre
Regional Cancer Centre
Regional Cancer Centre
National Cancer Centre
National Cancer Centre
National Cancer Centre
National Cancer Centre
Regional Cancer Centre
Regional Cancer Centre
Regional Cancer Centre
Regional Cancer Centre
Regional Cancer Centre
Regional Cancer Centre
Regional Cancer Centre
Regional Cancer Centre
Regional Cancer Centre
Regional Cancer Centre
Regional Cancer Centre
Regional Cancer Centre
Regional Cancer Centre
National Cancer Centre
National Cancer Centre
Regional Cancer Centre
Regional Cancer Centre
Regional Cancer Centre
Regional Cancer Centre

Regional Cancer Centre
Regional Cancer Centre
Regional Cancer Centre
Regional Cancer Centre
Regional Cancer Centre
Regional Cancer Centre
Regional Cancer Centre
Regional Cancer Centre
Regional Cancer Centre
Regional Cancer Centre
Regional Cancer Centre
Regional Cancer Centre
Regional Cancer Centre
National Cancer Centre
Regional Cancer Centre
Regional Cancer Centre
Regional Cancer Centre
Regional Cancer Centre
National Cancer Centre
Regional Cancer Centre
Regional Cancer Centre
Regional Cancer Centre
Regional Cancer Centre
Regional Cancer Centre
Regional Cancer Centre
Regional Cancer Centre
Regional Cancer Centre
Regional Cancer Centre
National Cancer Centre
National Cancer Centre
Regional Cancer Centre
Regional Cancer Centre
Regional Cancer Centre
Regional Cancer Centre
Regional Cancer Centre
Regional Cancer Centre
Regional Cancer Centre
Regional Cancer Centre
Regional Cancer Centre
Regional Cancer Centre
Regional Cancer Centre
Regional Cancer Centre
Regional Cancer Centre
Regional Cancer Centre
Regional Cancer Centre
Regional Cancer Centre
National Cancer Centre
National Cancer Centre
Regional Cancer Centre
Regional Cancer Centre

National Cancer Centre
National Cancer Centre
National Cancer Centre
National Cancer Centre
Regional Cancer Centre
Regional Cancer Centre
Regional Cancer Centre
Regional Cancer Centre
Regional Cancer Centre
Regional Cancer Centre
Regional Cancer Centre
Regional Cancer Centre
Regional Cancer Centre
Regional Cancer Centre
Regional Cancer Centre
Regional Cancer Centre
Regional Cancer Centre
Regional Cancer Centre
Regional Cancer Centre
National Cancer Centre
National Cancer Centre
Regional Cancer Centre
Regional Cancer Centre
Regional Cancer Centre
Regional Cancer Centre
Regional Cancer Centre
National Cancer Centre
National Cancer Centre
Regional Cancer Centre
Regional Cancer Centre
Regional Cancer Centre
Regional Cancer Centre
Regional Cancer Centre
Regional Cancer Centre
Regional Cancer Centre
Regional Cancer Centre
Regional Cancer Centre
National Cancer Centre
National Cancer Centre
Regional Cancer Centre
Regional Cancer Centre
Regional Cancer Centre
National Cancer Centre
National Cancer Centre
Regional Cancer Centre
Regional Cancer Centre
Regional Cancer Centre
Regional Cancer Centre
Regional Cancer Centre
National Cancer Centre

Regional Cancer Centre
Regional Cancer Centre
National Cancer Centre
National Cancer Centre
Regional Cancer Centre
National Cancer Centre
Regional Cancer Centre
Regional Cancer Centre
Regional Cancer Centre
Regional Cancer Centre
Regional Cancer Centre
Regional Cancer Centre
Regional Cancer Centre
Regional Cancer Centre
Regional Cancer Centre
Regional Cancer Centre
Regional Cancer Centre
Regional Cancer Centre
Regional Cancer Centre
Regional Cancer Centre
National Cancer Centre
Regional Cancer Centre
Regional Cancer Centre
Regional Cancer Centre
National Cancer Centre
Regional Cancer Centre
Regional Cancer Centre
National Cancer Centre
Regional Cancer Centre
Regional Cancer Centre
National Cancer Centre
Regional Cancer Centre
National Cancer Centre
Regional Cancer Centre
National Cancer Centre
Regional Cancer Centre
Regional Cancer Centre
Regional Cancer Centre
Regional Cancer Centre
Regional Cancer Centre
Regional Cancer Centre
Regional Cancer Centre
Regional Cancer Centre
Regional Cancer Centre
National Cancer Centre
Regional Cancer Centre
Regional Cancer Centre
Regional Cancer Centre
Regional Cancer Centre
Regional Cancer Centre

Regional Cancer Centre
Regional Cancer Centre
Regional Cancer Centre
National Cancer Centre
Regional Cancer Centre
Regional Cancer Centre
Regional Cancer Centre
Regional Cancer Centre
Regional Cancer Centre
Regional Cancer Centre
Regional Cancer Centre
National Cancer Centre
Regional Cancer Centre
Regional Cancer Centre
Regional Cancer Centre
Regional Cancer Centre
Regional Cancer Centre
Regional Cancer Centre
Regional Cancer Centre
Regional Cancer Centre
National Cancer Centre
National Cancer Centre
Regional Cancer Centre
Regional Cancer Centre
Regional Cancer Centre
Regional Cancer Centre
Regional Cancer Centre
Regional Cancer Centre
Regional Cancer Centre
Regional Cancer Centre
National Cancer Centre
Regional Cancer Centre
Regional Cancer Centre
Regional Cancer Centre
Regional Cancer Centre
Regional Cancer Centre
Regional Cancer Centre
Regional Cancer Centre
Regional Cancer Centre
National Cancer Centre
Regional Cancer Centre
National Cancer Centre
Regional Cancer Centre
National Cancer Centre
Regional Cancer Centre
Regional Cancer Centre
Regional Cancer Centre
Regional Cancer Centre
Regional Cancer Centre
Regional Cancer Centre

Regional Cancer Centre
Regional Cancer Centre
Regional Cancer Centre
Regional Cancer Centre
Regional Cancer Centre
Regional Cancer Centre
Regional Cancer Centre
Regional Cancer Centre
Regional Cancer Centre
Regional Cancer Centre
Regional Cancer Centre
Regional Cancer Centre
National Cancer Centre
Regional Cancer Centre
Regional Cancer Centre
Regional Cancer Centre
Regional Cancer Centre
Regional Cancer Centre
Regional Cancer Centre
Regional Cancer Centre
Regional Cancer Centre
Regional Cancer Centre
Regional Cancer Centre
Regional Cancer Centre
Regional Cancer Centre
Regional Cancer Centre
Regional Cancer Centre
Regional Cancer Centre
National Cancer Centre
Regional Cancer Centre
National Cancer Centre
National Cancer Centre
Regional Cancer Centre
Regional Cancer Centre
Regional Cancer Centre
Regional Cancer Centre
Regional Cancer Centre
Regional Cancer Centre
Regional Cancer Centre
Regional Cancer Centre
Regional Cancer Centre
National Cancer Centre
Regional Cancer Centre
Regional Cancer Centre
Regional Cancer Centre
Regional Cancer Centre
Regional Cancer Centre
Regional Cancer Centre
Regional Cancer Centre
National Cancer Centre

Regional Cancer Centre
Regional Cancer Centre
Regional Cancer Centre
Regional Cancer Centre
Regional Cancer Centre
Regional Cancer Centre
Regional Cancer Centre
Regional Cancer Centre
Regional Cancer Centre
Regional Cancer Centre
Regional Cancer Centre
National Cancer Centre
Regional Cancer Centre
Regional Cancer Centre
Regional Cancer Centre
Regional Cancer Centre
Regional Cancer Centre
Regional Cancer Centre
Regional Cancer Centre
Regional Cancer Centre
Regional Cancer Centre
National Cancer Centre
Regional Cancer Centre
Regional Cancer Centre
National Cancer Centre
National Cancer Centre
Regional Cancer Centre
National Cancer Centre
Regional Cancer Centre
Regional Cancer Centre
Regional Cancer Centre
Regional Cancer Centre
Regional Cancer Centre
Regional Cancer Centre
Regional Cancer Centre
Regional Cancer Centre
Regional Cancer Centre
Regional Cancer Centre
National Cancer Centre
Regional Cancer Centre
Regional Cancer Centre
Regional Cancer Centre
Regional Cancer Centre
Regional Cancer Centre
Regional Cancer Centre
Regional Cancer Centre
Regional Cancer Centre
Regional Cancer Centre
Regional Cancer Centre
Regional Cancer Centre

Regional Cancer Centre
Regional Cancer Centre
Regional Cancer Centre
Regional Cancer Centre
Regional Cancer Centre
Regional Cancer Centre
Regional Cancer Centre
Regional Cancer Centre
National Cancer Centre
Regional Cancer Centre
Regional Cancer Centre
National Cancer Centre
National Cancer Centre
Regional Cancer Centre
Regional Cancer Centre
Regional Cancer Centre
National Cancer Centre
Regional Cancer Centre
Regional Cancer Centre
Regional Cancer Centre
National Cancer Centre
Regional Cancer Centre
Regional Cancer Centre
Regional Cancer Centre
Regional Cancer Centre
Regional Cancer Centre
Regional Cancer Centre
Regional Cancer Centre
Regional Cancer Centre
Regional Cancer Centre
Regional Cancer Centre
Regional Cancer Centre
Regional Cancer Centre
Regional Cancer Centre
Regional Cancer Centre
Regional Cancer Centre
Regional Cancer Centre
National Cancer Centre
Regional Cancer Centre
Regional Cancer Centre
Regional Cancer Centre
Regional Cancer Centre
National Cancer Centre
Regional Cancer Centre
Regional Cancer Centre
Regional Cancer Centre
Regional Cancer Centre
Regional Cancer Centre
Regional Cancer Centre
Regional Cancer Centre

Regional Cancer Centre
Regional Cancer Centre
National Cancer Centre
National Cancer Centre
Regional Cancer Centre
Regional Cancer Centre
Regional Cancer Centre
Regional Cancer Centre
Regional Cancer Centre
Regional Cancer Centre
National Cancer Centre
National Cancer Centre
Regional Cancer Centre
Regional Cancer Centre
National Cancer Centre
Regional Cancer Centre
Regional Cancer Centre
Regional Cancer Centre
Regional Cancer Centre
Regional Cancer Centre
Regional Cancer Centre
Regional Cancer Centre
Regional Cancer Centre
Regional Cancer Centre
Regional Cancer Centre
Regional Cancer Centre
Regional Cancer Centre
Regional Cancer Centre
Regional Cancer Centre
Regional Cancer Centre
Regional Cancer Centre
National Cancer Centre
Regional Cancer Centre
Regional Cancer Centre
Regional Cancer Centre
Regional Cancer Centre
Regional Cancer Centre
Regional Cancer Centre
Regional Cancer Centre
Regional Cancer Centre
Regional Cancer Centre
Regional Cancer Centre
Regional Cancer Centre
Regional Cancer Centre
Regional Cancer Centre
Regional Cancer Centre
Regional Cancer Centre
National Cancer Centre
National Cancer Centre
Regional Cancer Centre
National Cancer Centre

National Cancer Centre
Regional Cancer Centre
Regional Cancer Centre
Regional Cancer Centre
Regional Cancer Centre
Regional Cancer Centre
Regional Cancer Centre
National Cancer Centre
Regional Cancer Centre
Regional Cancer Centre
Regional Cancer Centre
Regional Cancer Centre
National Cancer Centre
Regional Cancer Centre
Regional Cancer Centre
Regional Cancer Centre
National Cancer Centre
National Cancer Centre
Regional Cancer Centre
Regional Cancer Centre
Regional Cancer Centre
Regional Cancer Centre
Regional Cancer Centre
Regional Cancer Centre
Regional Cancer Centre
Regional Cancer Centre
Regional Cancer Centre
Regional Cancer Centre
Regional Cancer Centre
Regional Cancer Centre
National Cancer Centre
Regional Cancer Centre
Regional Cancer Centre
Regional Cancer Centre
Regional Cancer Centre
National Cancer Centre
National Cancer Centre
Regional Cancer Centre
Regional Cancer Centre
Regional Cancer Centre
Regional Cancer Centre
National Cancer Centre
Regional Cancer Centre
National Cancer Centre
National Cancer Centre
Regional Cancer Centre
Regional Cancer Centre
Regional Cancer Centre
Regional Cancer Centre
Regional Cancer Centre

Regional Cancer Centre
Regional Cancer Centre
Regional Cancer Centre
Regional Cancer Centre
Regional Cancer Centre
National Cancer Centre
Regional Cancer Centre
Regional Cancer Centre
Regional Cancer Centre
Regional Cancer Centre
Regional Cancer Centre
National Cancer Centre
Regional Cancer Centre
Regional Cancer Centre
National Cancer Centre
Regional Cancer Centre
Regional Cancer Centre
Regional Cancer Centre
Regional Cancer Centre
Regional Cancer Centre
Regional Cancer Centre
Regional Cancer Centre
National Cancer Centre
Regional Cancer Centre
Regional Cancer Centre
Regional Cancer Centre
Regional Cancer Centre
Regional Cancer Centre
Regional Cancer Centre
Regional Cancer Centre
Regional Cancer Centre
Regional Cancer Centre
Regional Cancer Centre
Regional Cancer Centre
Regional Cancer Centre
Regional Cancer Centre
Regional Cancer Centre
Regional Cancer Centre
Regional Cancer Centre
National Cancer Centre
Regional Cancer Centre
National Cancer Centre
National Cancer Centre
Regional Cancer Centre
Regional Cancer Centre
Regional Cancer Centre
Regional Cancer Centre
Regional Cancer Centre
Regional Cancer Centre
Regional Cancer Centre

Regional Cancer Centre
Regional Cancer Centre
Regional Cancer Centre
Regional Cancer Centre
Regional Cancer Centre
Regional Cancer Centre
Regional Cancer Centre
Regional Cancer Centre
Regional Cancer Centre
Regional Cancer Centre
Regional Cancer Centre
Regional Cancer Centre
Regional Cancer Centre
Regional Cancer Centre
Regional Cancer Centre
Regional Cancer Centre
Regional Cancer Centre
National Cancer Centre
Regional Cancer Centre
Regional Cancer Centre
Regional Cancer Centre
Regional Cancer Centre
Regional Cancer Centre
Regional Cancer Centre
Regional Cancer Centre
Regional Cancer Centre
Regional Cancer Centre
Regional Cancer Centre
Regional Cancer Centre
Regional Cancer Centre
Regional Cancer Centre
Regional Cancer Centre
Regional Cancer Centre
Regional Cancer Centre
Regional Cancer Centre
National Cancer Centre
National Cancer Centre
Regional Cancer Centre
Regional Cancer Centre
Regional Cancer Centre
Regional Cancer Centre
Regional Cancer Centre
Regional Cancer Centre
Regional Cancer Centre
Regional Cancer Centre
Regional Cancer Centre
Regional Cancer Centre
Regional Cancer Centre
Regional Cancer Centre
Regional Cancer Centre

Regional Cancer Centre
Regional Cancer Centre
Regional Cancer Centre
Regional Cancer Centre
Regional Cancer Centre
Regional Cancer Centre
Regional Cancer Centre
Regional Cancer Centre
Regional Cancer Centre
Regional Cancer Centre
Regional Cancer Centre
Regional Cancer Centre
Regional Cancer Centre
Regional Cancer Centre
Regional Cancer Centre
Regional Cancer Centre
Regional Cancer Centre
Regional Cancer Centre
Regional Cancer Centre
Regional Cancer Centre
Regional Cancer Centre
Regional Cancer Centre
Regional Cancer Centre
Regional Cancer Centre
Regional Cancer Centre
Regional Cancer Centre
Regional Cancer Centre
Regional Cancer Centre
Regional Cancer Centre
Regional Cancer Centre
Regional Cancer Centre
Regional Cancer Centre
Regional Cancer Centre
Regional Cancer Centre
Regional Cancer Centre
Regional Cancer Centre
Regional Cancer Centre
Regional Cancer Centre
Regional Cancer Centre
Regional Cancer Centre
Regional Cancer Centre
Regional Cancer Centre
Regional Cancer Centre
Regional Cancer Centre
Regional Cancer Centre
Regional Cancer Centre
Regional Cancer Centre
National Cancer Centre
Regional Cancer Centre
Regional Cancer Centre

Regional Cancer Centre
Regional Cancer Centre
Regional Cancer Centre
Regional Cancer Centre
Regional Cancer Centre
Regional Cancer Centre
Regional Cancer Centre
Regional Cancer Centre
Regional Cancer Centre
Regional Cancer Centre
Regional Cancer Centre
Regional Cancer Centre
Regional Cancer Centre
Regional Cancer Centre
Regional Cancer Centre
Regional Cancer Centre
Regional Cancer Centre
Regional Cancer Centre
Regional Cancer Centre
Regional Cancer Centre
Regional Cancer Centre
Regional Cancer Centre
Regional Cancer Centre
Regional Cancer Centre
Regional Cancer Centre
Regional Cancer Centre
Regional Cancer Centre
Regional Cancer Centre
Regional Cancer Centre
Regional Cancer Centre
Regional Cancer Centre
Regional Cancer Centre
Regional Cancer Centre
Regional Cancer Centre
Regional Cancer Centre
Regional Cancer Centre
Regional Cancer Centre
Regional Cancer Centre
Regional Cancer Centre
Regional Cancer Centre
Regional Cancer Centre
Regional Cancer Centre
Regional Cancer Centre
National Cancer Centre
National Cancer Centre
Regional Cancer Centre
National Cancer Centre
Regional Cancer Centre
Regional Cancer Centre
Regional Cancer Centre

Regional Cancer Centre
Regional Cancer Centre
Regional Cancer Centre
Regional Cancer Centre
Regional Cancer Centre
Regional Cancer Centre
Regional Cancer Centre
Regional Cancer Centre
Regional Cancer Centre
Regional Cancer Centre
Regional Cancer Centre
Regional Cancer Centre
Regional Cancer Centre
Regional Cancer Centre
Regional Cancer Centre
Regional Cancer Centre
Regional Cancer Centre
Regional Cancer Centre
Regional Cancer Centre
Regional Cancer Centre
Regional Cancer Centre
Regional Cancer Centre
Regional Cancer Centre
Regional Cancer Centre
Regional Cancer Centre
Regional Cancer Centre
Regional Cancer Centre
Regional Cancer Centre
Regional Cancer Centre
Regional Cancer Centre
Regional Cancer Centre
Regional Cancer Centre
Regional Cancer Centre
Regional Cancer Centre
Regional Cancer Centre
Regional Cancer Centre
Regional Cancer Centre
Regional Cancer Centre
National Cancer Centre
National Cancer Centre
Regional Cancer Centre
Regional Cancer Centre
Regional Cancer Centre
Regional Cancer Centre
Regional Cancer Centre
Regional Cancer Centre
Regional Cancer Centre
Regional Cancer Centre
Regional Cancer Centre
Regional Cancer Centre

Regional Cancer Centre
Regional Cancer Centre
Regional Cancer Centre
National Cancer Centre
Regional Cancer Centre
National Cancer Centre
Regional Cancer Centre
Regional Cancer Centre
Regional Cancer Centre
Regional Cancer Centre
Regional Cancer Centre
Regional Cancer Centre
Regional Cancer Centre
Regional Cancer Centre
Regional Cancer Centre
National Cancer Centre
Regional Cancer Centre
Regional Cancer Centre
Regional Cancer Centre
National Cancer Centre
National Cancer Centre
Regional Cancer Centre
Regional Cancer Centre
Regional Cancer Centre
Regional Cancer Centre
Regional Cancer Centre
Regional Cancer Centre
Regional Cancer Centre
Regional Cancer Centre
Regional Cancer Centre
Regional Cancer Centre
Regional Cancer Centre
Regional Cancer Centre
Regional Cancer Centre
Regional Cancer Centre
Regional Cancer Centre
Regional Cancer Centre
Regional Cancer Centre
Regional Cancer Centre
National Cancer Centre
Regional Cancer Centre
National Cancer Centre
Regional Cancer Centre
Regional Cancer Centre
National Cancer Centre
Regional Cancer Centre
Regional Cancer Centre
National Cancer Centre
National Cancer Centre
Regional Cancer Centre

Regional Cancer Centre
Regional Cancer Centre
Regional Cancer Centre
Regional Cancer Centre
Regional Cancer Centre
Regional Cancer Centre
Regional Cancer Centre
Regional Cancer Centre
Regional Cancer Centre
Regional Cancer Centre
National Cancer Centre
Regional Cancer Centre
Regional Cancer Centre
Regional Cancer Centre
Regional Cancer Centre
Regional Cancer Centre
Regional Cancer Centre
Regional Cancer Centre
Regional Cancer Centre

Treatments	Sex	Month
Chemotherapy	Male	April
Chemotherapy	Male	April
Chemotherapy	Male	April
Chemotherapy	Male	April
Chemotherapy	Male	April
Chemotherapy	Male	April
Chemotherapy	Male	April
Chemotherapy	Male	April
Chemotherapy	Female	April
Chemotherapy	Male	April
Chemotherapy	Male	August
Chemotherapy	Male	August
Chemotherapy	Female	August
Chemotherapy	Male	August
Chemotherapy	Female	August
Chemotherapy	Female	August
Chemotherapy	Male	August
Chemotherapy	Male	August
Chemotherapy	Female	August
Chemotherapy	Male	August
Chemotherapy	Female	August
Chemotherapy	Female	August
Chemotherapy	Female	December
Chemotherapy	Male	December
Chemotherapy	Female	December
Chemotherapy	Male	December
Chemotherapy	Male	December
Chemotherapy	Male	December
Chemotherapy	Male	December
Chemotherapy	Male	December
Chemotherapy	Male	December
Chemotherapy	Male	February
Chemotherapy	Female	February
Chemotherapy	Female	February
Chemotherapy	Male	February
Chemotherapy	Male	February
Chemotherapy	Female	February
Chemotherapy	Male	February
Chemotherapy	Male	February
Chemotherapy	Male	February
Chemotherapy	Female	February
Chemotherapy	Male	February
Chemotherapy	Male	February
Chemotherapy	Female	January
Chemotherapy	Male	January
Chemotherapy	Male	January
Chemotherapy	Female	January
Chemotherapy	Male	January
Chemotherapy	Female	January

Chemotherapy	Male	January
Chemotherapy	Male	January
Chemotherapy	Female	January
Chemotherapy	Male	January
Chemotherapy	Male	January
Chemotherapy	Female	January
Chemotherapy	Male	January
Chemotherapy	Male	January
Chemotherapy	Female	January
Chemotherapy	Male	January
Chemotherapy	Male	July
Chemotherapy	Female	July
Chemotherapy	Male	July
Chemotherapy	Female	July
Chemotherapy	Male	July
Chemotherapy	Male	July
Chemotherapy	Female	July
Chemotherapy	Male	July
Chemotherapy	Female	July
Chemotherapy	Male	June
Chemotherapy	Male	June
Chemotherapy	Female	June
Chemotherapy	Male	June
Chemotherapy	Female	June
Chemotherapy	Male	June
Chemotherapy	Female	June
Chemotherapy	Male	June
Chemotherapy	Female	June
Chemotherapy	Female	March
Chemotherapy	Male	March
Chemotherapy	Female	March
Chemotherapy	Male	March
Chemotherapy	Male	March
Chemotherapy	Male	March
Chemotherapy	Male	March
Chemotherapy	Female	March
Chemotherapy	Male	May
Chemotherapy	Male	May
Chemotherapy	Female	May
Chemotherapy	Male	May
Chemotherapy	Male	May
Chemotherapy	Male	May
Chemotherapy	Male	May
Chemotherapy	Female	November
Chemotherapy	Male	November
Chemotherapy	Female	November
Chemotherapy	Female	November
Chemotherapy	Female	November
Chemotherapy	Female	November
Chemotherapy	Male	November

Chemotherapy	Male	November
Chemotherapy	Male	November
Chemotherapy	Male	November
Chemotherapy	Female	November
Chemotherapy	Male	October
Chemotherapy	Male	October
Chemotherapy	Female	October
Chemotherapy	Male	October
Chemotherapy	Male	October
Chemotherapy	Female	October
Chemotherapy	Male	October
Chemotherapy	Male	October
Chemotherapy	Female	October
Chemotherapy	Male	September
Chemotherapy	Female	September
Chemotherapy	Male	September
Chemotherapy	Female	September
Chemotherapy	Female	September
Chemotherapy	Male	September
Chemotherapy	Male	September
Chemotherapy	Male	April
Chemotherapy	Female	April
Chemotherapy	Male	April
Chemotherapy	Male	April
Chemotherapy	Female	April
Chemotherapy	Male	April
Chemotherapy	Female	April
Chemotherapy	Female	April
Chemotherapy	Male	April
Chemotherapy	Female	August
Chemotherapy	Male	August
Chemotherapy	Female	August
Chemotherapy	Male	August
Chemotherapy	Male	August
Chemotherapy	Male	August
Chemotherapy	Male	August
Chemotherapy	Female	August
Chemotherapy	Female	August
Chemotherapy	Male	August
Chemotherapy	Male	August
Chemotherapy	Female	December
Chemotherapy	Male	December
Chemotherapy	Female	December
Chemotherapy	Male	December
Chemotherapy	Male	December
Chemotherapy	Female	December
Chemotherapy	Male	December
Chemotherapy	Male	December
Chemotherapy	Male	December
Chemotherapy	Male	February

Chemotherapy	Female	February
Chemotherapy	Female	February
Chemotherapy	Male	February
Chemotherapy	Male	February
Chemotherapy	Male	February
Chemotherapy	Female	February
Chemotherapy	Male	February
Chemotherapy	Female	February
Chemotherapy	Male	February
Chemotherapy	Female	February
Chemotherapy	Female	January
Chemotherapy	Male	January
Chemotherapy	Male	January
Chemotherapy	Male	January
Chemotherapy	Male	January
Chemotherapy	Female	January
Chemotherapy	Female	January
Chemotherapy	Male	January
Chemotherapy	Female	January
Chemotherapy	Female	January
Chemotherapy	Male	July
Chemotherapy	Male	July
Chemotherapy	Male	July
Chemotherapy	Male	July
Chemotherapy	Female	July
Chemotherapy	Female	July
Chemotherapy	Male	July
Chemotherapy	Female	July
Chemotherapy	Male	July
Chemotherapy	Male	June
Chemotherapy	Female	June
Chemotherapy	Male	June
Chemotherapy	Female	June
Chemotherapy	Female	June
Chemotherapy	Male	June
Chemotherapy	Male	June
Chemotherapy	Male	March
Chemotherapy	Female	March
Chemotherapy	Male	March
Chemotherapy	Male	March
Chemotherapy	Female	March
Chemotherapy	Female	March
Chemotherapy	Male	March
Chemotherapy	Male	March
Chemotherapy	Female	May
Chemotherapy	Male	May
Chemotherapy	Male	May
Chemotherapy	Male	May
Chemotherapy	Male	May
Chemotherapy	Female	November

Chemotherapy	Female	November
Chemotherapy	Female	November
Chemotherapy	Male	November
Chemotherapy	Male	November
Chemotherapy	Male	November
Chemotherapy	Male	November
Chemotherapy	Male	November
Chemotherapy	Male	October
Chemotherapy	Female	October
Chemotherapy	Male	October
Chemotherapy	Female	October
Chemotherapy	Male	October
Chemotherapy	Male	October
Chemotherapy	Male	October
Chemotherapy	Female	October
Chemotherapy	Female	October
Chemotherapy	Male	October
Chemotherapy	Male	September
Chemotherapy	Male	September
Chemotherapy	Male	September
Chemotherapy	Male	September
Chemotherapy	Female	September
Chemotherapy	Female	September
Chemotherapy	Male	September
Chemotherapy	Female	September
Chemotherapy	Male	April
Chemotherapy	Male	April
Chemotherapy	Male	April
Chemotherapy	Female	April
Chemotherapy	Male	April
Chemotherapy	Female	April
Chemotherapy	Female	April
Chemotherapy	Male	April
Chemotherapy	Male	April
Chemotherapy	Female	April
Chemotherapy	Male	April
Chemotherapy	Female	April
Chemotherapy	Male	August
Chemotherapy	Female	August
Chemotherapy	Female	August
Chemotherapy	Male	August
Chemotherapy	Male	August
Chemotherapy	Male	August
Chemotherapy	Male	August
Chemotherapy	Male	August
Chemotherapy	Female	August
Chemotherapy	Male	December
Chemotherapy	Female	December
Chemotherapy	Male	December
Chemotherapy	Female	December

Chemotherapy	Male	December
Chemotherapy	Male	December
Chemotherapy	Male	December
Chemotherapy	Female	December
Chemotherapy	Female	December
Chemotherapy	Male	December
Chemotherapy	Female	February
Chemotherapy	Male	February
Chemotherapy	Female	February
Chemotherapy	Male	February
Chemotherapy	Female	February
Chemotherapy	Female	February
Chemotherapy	Male	February
Chemotherapy	Male	February
Chemotherapy	Male	February
Chemotherapy	Male	February
Chemotherapy	Male	January
Chemotherapy	Female	January
Chemotherapy	Male	January
Chemotherapy	Female	January
Chemotherapy	Male	January
Chemotherapy	Male	January
Chemotherapy	Female	January
Chemotherapy	Male	January
Chemotherapy	Male	January
Chemotherapy	Female	January
Chemotherapy	Male	January
Chemotherapy	Female	January
Chemotherapy	Female	July
Chemotherapy	Male	July
Chemotherapy	Male	July
Chemotherapy	Male	July
Chemotherapy	Male	July
Chemotherapy	Male	July
Chemotherapy	Female	July
Chemotherapy	Male	July
Chemotherapy	Female	June
Chemotherapy	Female	June
Chemotherapy	Male	June
Chemotherapy	Male	June
Chemotherapy	Male	June
Chemotherapy	Male	June
Chemotherapy	Female	June
Chemotherapy	Male	June
Chemotherapy	Male	June
Chemotherapy	Female	June
Chemotherapy	Male	March
Chemotherapy	Female	March
Chemotherapy	Male	March
Chemotherapy	Male	March

Chemotherapy	Male	March
Chemotherapy	Male	March
Chemotherapy	Female	March
Chemotherapy	Female	March
Chemotherapy	Male	March
Chemotherapy	Female	March
Chemotherapy	Male	March
Chemotherapy	Female	May
Chemotherapy	Male	May
Chemotherapy	Female	May
Chemotherapy	Female	May
Chemotherapy	Male	May
Chemotherapy	Male	May
Chemotherapy	Female	November
Chemotherapy	Male	November
Chemotherapy	Male	November
Chemotherapy	Female	November
Chemotherapy	Male	November
Chemotherapy	Female	November
Chemotherapy	Male	November
Chemotherapy	Male	October
Chemotherapy	Male	October
Chemotherapy	Female	October
Chemotherapy	Male	October
Chemotherapy	Male	October
Chemotherapy	Female	October
Chemotherapy	Male	October
Chemotherapy	Female	October
Chemotherapy	Female	October
Chemotherapy	Male	October
Chemotherapy	Male	September
Chemotherapy	Male	September
Chemotherapy	Male	September
Chemotherapy	Female	September
Chemotherapy	Male	September
Chemotherapy	Male	September
Chemotherapy	Female	September
Chemotherapy	Male	September
Chemotherapy	Male	September
Chemotherapy	Male	April
Chemotherapy	Female	April
Chemotherapy	Female	April
Chemotherapy	Female	April
Chemotherapy	Male	April
Chemotherapy	Female	April
Chemotherapy	Male	April
Chemotherapy	Female	April
Chemotherapy	Male	April
Chemotherapy	Male	August
Chemotherapy	Female	August

Chemotherapy	Female	August
Chemotherapy	Female	August
Chemotherapy	Female	August
Chemotherapy	Male	August
Chemotherapy	Male	August
Chemotherapy	Female	August
Chemotherapy	Female	August
Chemotherapy	Male	December
Chemotherapy	Female	December
Chemotherapy	Male	December
Chemotherapy	Female	December
Chemotherapy	Male	December
Chemotherapy	Female	December
Chemotherapy	Female	December
Chemotherapy	Female	December
Chemotherapy	Female	December
Chemotherapy	Male	December
Chemotherapy	Male	December
Chemotherapy	Male	February
Chemotherapy	Male	February
Chemotherapy	Female	February
Chemotherapy	Male	February
Chemotherapy	Female	February
Chemotherapy	Male	February
Chemotherapy	Male	February
Chemotherapy	Female	February
Chemotherapy	Male	February
Chemotherapy	Male	January
Chemotherapy	Female	January
Chemotherapy	Female	January
Chemotherapy	Male	January
Chemotherapy	Male	January
Chemotherapy	Female	January
Chemotherapy	Male	January
Chemotherapy	Female	January
Chemotherapy	Male	January
Chemotherapy	Female	January
Chemotherapy	Male	January
Chemotherapy	Female	January
Chemotherapy	Male	January
Chemotherapy	Female	January
Chemotherapy	Male	January
Chemotherapy	Male	July
Chemotherapy	Male	July
Chemotherapy	Male	July
Chemotherapy	Female	July
Chemotherapy	Female	July
Chemotherapy	Male	July
Chemotherapy	Female	July
Chemotherapy	Female	July

Chemotherapy	Male	June
Chemotherapy	Male	June
Chemotherapy	Female	June
Chemotherapy	Female	June
Chemotherapy	Female	June
Chemotherapy	Male	June
Chemotherapy	Male	June
Chemotherapy	Female	June
Chemotherapy	Male	June
Chemotherapy	Female	March
Chemotherapy	Male	March
Chemotherapy	Female	March
Chemotherapy	Female	March
Chemotherapy	Female	March
Chemotherapy	Male	March
Chemotherapy	Female	March
Chemotherapy	Female	March
Chemotherapy	Male	March
Chemotherapy	Female	May
Chemotherapy	Male	May
Chemotherapy	Female	May
Chemotherapy	Female	May
Chemotherapy	Male	May
Chemotherapy	Female	November
Chemotherapy	Male	November
Chemotherapy	Female	November
Chemotherapy	Female	November
Chemotherapy	Male	November
Chemotherapy	Male	November
Chemotherapy	Female	November
Chemotherapy	Male	November
Chemotherapy	Female	November
Chemotherapy	Female	November
Chemotherapy	Female	November
Chemotherapy	Male	November
Chemotherapy	Female	October
Chemotherapy	Male	October
Chemotherapy	Female	October
Chemotherapy	Female	October
Chemotherapy	Female	October
Chemotherapy	Male	October
Chemotherapy	Female	October
Chemotherapy	Female	October
Chemotherapy	Female	October
Chemotherapy	Male	October
Chemotherapy	Male	September
Chemotherapy	Female	September
Chemotherapy	Female	September
Chemotherapy	Female	September
Chemotherapy	Female	September

Chemotherapy	Female	September
Chemotherapy	Male	September
Chemotherapy	Male	September
Chemotherapy	Female	September
Chemotherapy	Female	September
Chemotherapy	Male	September
Chemotherapy	Male	April
Chemotherapy	Female	April
Chemotherapy	Male	April
Chemotherapy	Female	April
Chemotherapy	Female	April
Chemotherapy	Female	April
Chemotherapy	Female	August
Chemotherapy	Male	August
Chemotherapy	Male	August
Chemotherapy	Female	August
Chemotherapy	Female	August
Chemotherapy	Male	August
Chemotherapy	Male	August
Chemotherapy	Female	August
Chemotherapy	Male	August
Chemotherapy	Female	August
Chemotherapy	Female	December
Chemotherapy	Female	December
Chemotherapy	Male	December
Chemotherapy	Male	December
Chemotherapy	Female	December
Chemotherapy	Female	December
Chemotherapy	Female	December
Chemotherapy	Male	December
Chemotherapy	Male	February
Chemotherapy	Female	February
Chemotherapy	Female	February
Chemotherapy	Male	February
Chemotherapy	Male	February
Chemotherapy	Female	February
Chemotherapy	Female	February
Chemotherapy	Female	February
Chemotherapy	Male	February
Chemotherapy	Female	February
Chemotherapy	Female	February
Chemotherapy	Male	February
Chemotherapy	Female	January
Chemotherapy	Male	January
Chemotherapy	Male	January
Chemotherapy	Female	January
Chemotherapy	Male	January
Chemotherapy	Female	January
Chemotherapy	Male	January
Chemotherapy	Female	January

Chemotherapy	Male	January
Chemotherapy	Female	January
Chemotherapy	Female	January
Chemotherapy	Male	January
Chemotherapy	Male	July
Chemotherapy	Female	July
Chemotherapy	Female	July
Chemotherapy	Male	July
Chemotherapy	Male	July
Chemotherapy	Female	July
Chemotherapy	Male	July
Chemotherapy	Male	June
Chemotherapy	Female	June
Chemotherapy	Male	June
Chemotherapy	Female	June
Chemotherapy	Male	June
Chemotherapy	Female	June
Chemotherapy	Male	June
Chemotherapy	Male	June
Chemotherapy	Female	June
Chemotherapy	Male	June
Chemotherapy	Female	June
Chemotherapy	Female	March
Chemotherapy	Female	March
Chemotherapy	Female	March
Chemotherapy	Male	March
Chemotherapy	Female	March
Chemotherapy	Male	March
Chemotherapy	Female	March
Chemotherapy	Male	March
Chemotherapy	Male	March
Chemotherapy	Female	March
Chemotherapy	Female	May
Chemotherapy	Female	May
Chemotherapy	Male	May
Chemotherapy	Female	May
Chemotherapy	Female	May
Chemotherapy	Male	May
Chemotherapy	Female	November
Chemotherapy	Male	November
Chemotherapy	Male	November
Chemotherapy	Female	November
Chemotherapy	Male	November
Chemotherapy	Female	November
Chemotherapy	Male	November
Chemotherapy	Female	November
Chemotherapy	Female	October
Chemotherapy	Female	October
Chemotherapy	Male	October
Chemotherapy	Female	October

Chemotherapy	Male	October
Chemotherapy	Female	October
Chemotherapy	Female	September
Chemotherapy	Male	September
Chemotherapy	Female	September
Chemotherapy	Female	September
Chemotherapy	Male	April
Chemotherapy	Female	April
Chemotherapy	Male	April
Chemotherapy	Female	April
Chemotherapy	Male	April
Chemotherapy	Male	August
Chemotherapy	Male	August
Chemotherapy	Female	August
Chemotherapy	Male	August
Chemotherapy	Male	August
Chemotherapy	Female	August
Chemotherapy	Female	August
Chemotherapy	Male	August
Chemotherapy	Female	December
Chemotherapy	Male	December
Chemotherapy	Female	December
Chemotherapy	Male	December
Chemotherapy	Male	December
Chemotherapy	Female	December
Chemotherapy	Male	December
Chemotherapy	Female	December
Chemotherapy	Male	February
Chemotherapy	Male	February
Chemotherapy	Female	February
Chemotherapy	Male	February
Chemotherapy	Female	February
Chemotherapy	Female	February
Chemotherapy	Female	February
Chemotherapy	Male	January
Chemotherapy	Female	January
Chemotherapy	Female	January
Chemotherapy	Male	January
Chemotherapy	Female	January
Chemotherapy	Male	January
Chemotherapy	Female	January
Chemotherapy	Female	January
Chemotherapy	Male	January
Chemotherapy	Male	July
Chemotherapy	Female	July
Chemotherapy	Female	July
Chemotherapy	Female	July
Chemotherapy	Male	July
Chemotherapy	Female	July
Chemotherapy	Male	July

Chemotherapy	Female	June
Chemotherapy	Male	June
Chemotherapy	Female	June
Chemotherapy	Female	June
Chemotherapy	Male	June
Chemotherapy	Male	June
Chemotherapy	Male	June
Chemotherapy	Female	March
Chemotherapy	Male	March
Chemotherapy	Male	March
Chemotherapy	Female	March
Chemotherapy	Male	March
Chemotherapy	Male	March
Chemotherapy	Female	March
Chemotherapy	Male	March
Chemotherapy	Female	March
Chemotherapy	Male	March
Chemotherapy	Male	May
Chemotherapy	Female	November
Chemotherapy	Male	November
Chemotherapy	Female	November
Chemotherapy	Male	November
Chemotherapy	Female	November
Chemotherapy	Male	November
Chemotherapy	Male	November
Chemotherapy	Female	November
Chemotherapy	Male	October
Chemotherapy	Male	October
Chemotherapy	Female	October
Chemotherapy	Male	October
Chemotherapy	Female	October
Chemotherapy	Female	September
Chemotherapy	Female	September
Chemotherapy	Male	September
Chemotherapy	Male	September
Chemotherapy	Male	September
Chemotherapy	Female	September
Chemotherapy	Male	September
Chemotherapy	Male	April
Chemotherapy	Male	April
Chemotherapy	Female	April
Chemotherapy	Male	April
Chemotherapy	Female	April
Chemotherapy	Male	August
Chemotherapy	Female	August
Chemotherapy	Male	August
Chemotherapy	Male	August
Chemotherapy	Female	August
Chemotherapy	Male	August
Chemotherapy	Female	August

Chemotherapy	Male	August
Chemotherapy	Female	December
Chemotherapy	Female	December
Chemotherapy	Male	December
Chemotherapy	Male	December
Chemotherapy	Female	December
Chemotherapy	Male	February
Chemotherapy	Female	February
Chemotherapy	Male	February
Chemotherapy	Female	February
Chemotherapy	Female	February
Chemotherapy	Female	February
Chemotherapy	Male	February
Chemotherapy	Female	February
Chemotherapy	Male	January
Chemotherapy	Male	January
Chemotherapy	Female	January
Chemotherapy	Male	January
Chemotherapy	Male	January
Chemotherapy	Female	January
Chemotherapy	Female	January
Chemotherapy	Female	January
Chemotherapy	Male	January
Chemotherapy	Male	July
Chemotherapy	Female	July
Chemotherapy	Male	July
Chemotherapy	Male	July
Chemotherapy	Male	July
Chemotherapy	Male	July
Chemotherapy	Female	July
Chemotherapy	Female	June
Chemotherapy	Male	June
Chemotherapy	Male	June
Chemotherapy	Female	June
Chemotherapy	Male	June
Chemotherapy	Female	June
Chemotherapy	Female	June
Chemotherapy	Male	March
Chemotherapy	Female	March
Chemotherapy	Male	March
Chemotherapy	Female	March
Chemotherapy	Male	March
Chemotherapy	Female	May
Chemotherapy	Male	May
Chemotherapy	Female	November
Chemotherapy	Male	November
Chemotherapy	Male	November
Chemotherapy	Female	November
Chemotherapy	Female	November
Chemotherapy	Male	November

Chemotherapy	Female	October
Chemotherapy	Male	October
Chemotherapy	Female	October
Chemotherapy	Female	October
Chemotherapy	Male	October
Chemotherapy	Female	October
Chemotherapy	Male	September
Chemotherapy	Male	September
Chemotherapy	Female	September
Chemotherapy	Male	April
Chemotherapy	Male	April
Chemotherapy	Male	April
Chemotherapy	Female	April
Chemotherapy	Male	April
Chemotherapy	Female	August
Chemotherapy	Female	August
Chemotherapy	Male	August
Chemotherapy	Male	August
Chemotherapy	Female	August
Chemotherapy	Female	August
Chemotherapy	Male	August
Chemotherapy	Male	December
Chemotherapy	Male	December
Chemotherapy	Female	December
Chemotherapy	Female	December
Chemotherapy	Male	February
Chemotherapy	Female	February
Chemotherapy	Male	February
Chemotherapy	Female	February
Chemotherapy	Female	February
Chemotherapy	Male	February
Chemotherapy	Male	February
Chemotherapy	Male	February
Chemotherapy	Female	January
Chemotherapy	Male	January
Chemotherapy	Male	January
Chemotherapy	Female	January
Chemotherapy	Female	January
Chemotherapy	Male	January
Chemotherapy	Male	January
Chemotherapy	Female	January
Chemotherapy	Male	January
Chemotherapy	Male	July
Chemotherapy	Female	July
Chemotherapy	Male	July
Chemotherapy	Male	July
Chemotherapy	Male	July
Chemotherapy	Female	July
Chemotherapy	Male	June
Chemotherapy	Female	June

Chemotherapy	Male	June
Chemotherapy	Male	June
Chemotherapy	Male	June
Chemotherapy	Female	June
Chemotherapy	Female	June
Chemotherapy	Male	June
Chemotherapy	Male	March
Chemotherapy	Male	March
Chemotherapy	Female	March
Chemotherapy	Male	March
Chemotherapy	Male	March
Chemotherapy	Female	March
Chemotherapy	Male	May
Chemotherapy	Male	May
Chemotherapy	Male	May
Chemotherapy	Female	November
Chemotherapy	Male	November
Chemotherapy	Female	November
Chemotherapy	Female	November
Chemotherapy	Female	November
Chemotherapy	Male	November
Chemotherapy	Male	November
Chemotherapy	Male	November
Chemotherapy	Male	October
Chemotherapy	Female	October
Chemotherapy	Male	October
Chemotherapy	Female	October
Chemotherapy	Male	September
Chemotherapy	Male	September
Chemotherapy	Female	September
Hormonal or Targeted therapies	Male	April
Hormonal or Targeted therapies	Male	August
Hormonal or Targeted therapies	Male	December
Hormonal or Targeted therapies	Male	February
Hormonal or Targeted therapies	Male	January
Hormonal or Targeted therapies	Male	July
Hormonal or Targeted therapies	Male	June
Hormonal or Targeted therapies	Male	March
Hormonal or Targeted therapies	Male	May
Hormonal or Targeted therapies	Male	November
Hormonal or Targeted therapies	Male	September
Hormonal or Targeted therapies	Female	April
Hormonal or Targeted therapies	Female	April
Hormonal or Targeted therapies	Male	April
Hormonal or Targeted therapies	Male	April
Hormonal or Targeted therapies	Female	April
Hormonal or Targeted therapies	Female	April
Hormonal or Targeted therapies	Male	April
Hormonal or Targeted therapies	Male	April
Hormonal or Targeted therapies	Female	April

Hormonal or Targeted therapies	Female	April
Hormonal or Targeted therapies	Female	August
Hormonal or Targeted therapies	Female	August
Hormonal or Targeted therapies	Female	December
Hormonal or Targeted therapies	Male	December
Hormonal or Targeted therapies	Female	December
Hormonal or Targeted therapies	Female	December
Hormonal or Targeted therapies	Female	December
Hormonal or Targeted therapies	Male	December
Hormonal or Targeted therapies	Female	December
Hormonal or Targeted therapies	Female	February
Hormonal or Targeted therapies	Female	February
Hormonal or Targeted therapies	Female	February
Hormonal or Targeted therapies	Male	February
Hormonal or Targeted therapies	Male	February
Hormonal or Targeted therapies	Female	February
Hormonal or Targeted therapies	Female	February
Hormonal or Targeted therapies	Male	February
Hormonal or Targeted therapies	Male	February
Hormonal or Targeted therapies	Male	February
Hormonal or Targeted therapies	Female	January
Hormonal or Targeted therapies	Female	January
Hormonal or Targeted therapies	Male	January
Hormonal or Targeted therapies	Male	January
Hormonal or Targeted therapies	Female	January
Hormonal or Targeted therapies	Female	January
Hormonal or Targeted therapies	Male	January
Hormonal or Targeted therapies	Male	January
Hormonal or Targeted therapies	Female	January
Hormonal or Targeted therapies	Female	July
Hormonal or Targeted therapies	Male	July
Hormonal or Targeted therapies	Female	July
Hormonal or Targeted therapies	Male	July
Hormonal or Targeted therapies	Male	June
Hormonal or Targeted therapies	Female	June
Hormonal or Targeted therapies	Female	June
Hormonal or Targeted therapies	Male	June
Hormonal or Targeted therapies	Female	June
Hormonal or Targeted therapies	Female	June
Hormonal or Targeted therapies	Female	March
Hormonal or Targeted therapies	Female	March
Hormonal or Targeted therapies	Male	March
Hormonal or Targeted therapies	Male	March
Hormonal or Targeted therapies	Female	March
Hormonal or Targeted therapies	Female	March
Hormonal or Targeted therapies	Female	March
Hormonal or Targeted therapies	Female	May
Hormonal or Targeted therapies	Male	May
Hormonal or Targeted therapies	Female	May
Hormonal or Targeted therapies	Female	May

Hormonal or Targeted therapies	Female	May
Hormonal or Targeted therapies	Male	November
Hormonal or Targeted therapies	Male	November
Hormonal or Targeted therapies	Female	November
Hormonal or Targeted therapies	Female	November
Hormonal or Targeted therapies	Female	November
Hormonal or Targeted therapies	Male	November
Hormonal or Targeted therapies	Male	November
Hormonal or Targeted therapies	Female	November
Hormonal or Targeted therapies	Female	November
Hormonal or Targeted therapies	Female	October
Hormonal or Targeted therapies	Male	October
Hormonal or Targeted therapies	Female	October
Hormonal or Targeted therapies	Female	October
Hormonal or Targeted therapies	Male	October
Hormonal or Targeted therapies	Male	September
Hormonal or Targeted therapies	Female	September
Hormonal or Targeted therapies	Female	September
Hormonal or Targeted therapies	Female	September
Hormonal or Targeted therapies	Female	September
Hormonal or Targeted therapies	Male	April
Hormonal or Targeted therapies	Male	April
Hormonal or Targeted therapies	Female	April
Hormonal or Targeted therapies	Male	April
Hormonal or Targeted therapies	Male	April
Hormonal or Targeted therapies	Female	April
Hormonal or Targeted therapies	Female	April
Hormonal or Targeted therapies	Male	April
Hormonal or Targeted therapies	Female	August
Hormonal or Targeted therapies	Male	August
Hormonal or Targeted therapies	Female	August
Hormonal or Targeted therapies	Male	August
Hormonal or Targeted therapies	Male	August
Hormonal or Targeted therapies	Male	August
Hormonal or Targeted therapies	Male	August
Hormonal or Targeted therapies	Female	August
Hormonal or Targeted therapies	Female	December
Hormonal or Targeted therapies	Male	December
Hormonal or Targeted therapies	Male	December
Hormonal or Targeted therapies	Male	December
Hormonal or Targeted therapies	Female	December
Hormonal or Targeted therapies	Male	December
Hormonal or Targeted therapies	Male	December
Hormonal or Targeted therapies	Female	December
Hormonal or Targeted therapies	Female	December
Hormonal or Targeted therapies	Female	February
Hormonal or Targeted therapies	Male	February
Hormonal or Targeted therapies	Female	February
Hormonal or Targeted therapies	Female	February
Hormonal or Targeted therapies	Male	February

Hormonal or Targeted therapies	Male	February
Hormonal or Targeted therapies	Female	February
Hormonal or Targeted therapies	Male	January
Hormonal or Targeted therapies	Male	January
Hormonal or Targeted therapies	Male	January
Hormonal or Targeted therapies	Female	January
Hormonal or Targeted therapies	Female	January
Hormonal or Targeted therapies	Male	January
Hormonal or Targeted therapies	Female	January
Hormonal or Targeted therapies	Male	January
Hormonal or Targeted therapies	Male	July
Hormonal or Targeted therapies	Female	July
Hormonal or Targeted therapies	Female	July
Hormonal or Targeted therapies	Male	July
Hormonal or Targeted therapies	Female	July
Hormonal or Targeted therapies	Male	July
Hormonal or Targeted therapies	Male	July
Hormonal or Targeted therapies	Male	July
Hormonal or Targeted therapies	Female	July
Hormonal or Targeted therapies	Female	June
Hormonal or Targeted therapies	Male	June
Hormonal or Targeted therapies	Female	June
Hormonal or Targeted therapies	Female	June
Hormonal or Targeted therapies	Male	June
Hormonal or Targeted therapies	Male	June
Hormonal or Targeted therapies	Male	June
Hormonal or Targeted therapies	Male	March
Hormonal or Targeted therapies	Female	March
Hormonal or Targeted therapies	Female	March
Hormonal or Targeted therapies	Male	March
Hormonal or Targeted therapies	Male	March
Hormonal or Targeted therapies	Female	March
Hormonal or Targeted therapies	Male	March
Hormonal or Targeted therapies	Female	March
Hormonal or Targeted therapies	Male	March
Hormonal or Targeted therapies	Male	May
Hormonal or Targeted therapies	Male	May
Hormonal or Targeted therapies	Male	May
Hormonal or Targeted therapies	Female	May
Hormonal or Targeted therapies	Female	May
Hormonal or Targeted therapies	Male	November
Hormonal or Targeted therapies	Male	November
Hormonal or Targeted therapies	Male	November
Hormonal or Targeted therapies	Female	November
Hormonal or Targeted therapies	Male	November
Hormonal or Targeted therapies	Female	November
Hormonal or Targeted therapies	Male	November
Hormonal or Targeted therapies	Male	October
Hormonal or Targeted therapies	Female	October
Hormonal or Targeted therapies	Male	October

Hormonal or Targeted therapies	Female	October
Hormonal or Targeted therapies	Male	October
Hormonal or Targeted therapies	Male	October
Hormonal or Targeted therapies	Female	October
Hormonal or Targeted therapies	Male	October
Hormonal or Targeted therapies	Female	September
Hormonal or Targeted therapies	Male	September
Hormonal or Targeted therapies	Male	September
Hormonal or Targeted therapies	Female	September
Hormonal or Targeted therapies	Male	September
Hormonal or Targeted therapies	Female	September
Hormonal or Targeted therapies	Male	September
Hormonal or Targeted therapies	Female	April
Hormonal or Targeted therapies	Male	April
Hormonal or Targeted therapies	Male	April
Hormonal or Targeted therapies	Female	April
Hormonal or Targeted therapies	Female	April
Hormonal or Targeted therapies	Male	April
Hormonal or Targeted therapies	Female	April
Hormonal or Targeted therapies	Female	April
Hormonal or Targeted therapies	Female	April
Hormonal or Targeted therapies	Male	April
Hormonal or Targeted therapies	Female	April
Hormonal or Targeted therapies	Male	April
Hormonal or Targeted therapies	Female	August
Hormonal or Targeted therapies	Male	August
Hormonal or Targeted therapies	Female	August
Hormonal or Targeted therapies	Female	August
Hormonal or Targeted therapies	Female	August
Hormonal or Targeted therapies	Male	August
Hormonal or Targeted therapies	Female	December
Hormonal or Targeted therapies	Male	December
Hormonal or Targeted therapies	Male	December
Hormonal or Targeted therapies	Female	December
Hormonal or Targeted therapies	Female	December
Hormonal or Targeted therapies	Male	December
Hormonal or Targeted therapies	Female	December
Hormonal or Targeted therapies	Female	December
Hormonal or Targeted therapies	Female	December
Hormonal or Targeted therapies	Male	December
Hormonal or Targeted therapies	Female	December
Hormonal or Targeted therapies	Male	February
Hormonal or Targeted therapies	Male	February
Hormonal or Targeted therapies	Female	February
Hormonal or Targeted therapies	Female	February
Hormonal or Targeted therapies	Female	February
Hormonal or Targeted therapies	Male	February
Hormonal or Targeted therapies	Female	February
Hormonal or Targeted therapies	Male	February
Hormonal or Targeted therapies	Female	February

Hormonal or Targeted therapies	Male	February
Hormonal or Targeted therapies	Female	February
Hormonal or Targeted therapies	Male	February
Hormonal or Targeted therapies	Male	January
Hormonal or Targeted therapies	Female	January
Hormonal or Targeted therapies	Male	January
Hormonal or Targeted therapies	Male	January
Hormonal or Targeted therapies	Female	January
Hormonal or Targeted therapies	Male	January
Hormonal or Targeted therapies	Female	January
Hormonal or Targeted therapies	Male	January
Hormonal or Targeted therapies	Female	January
Hormonal or Targeted therapies	Male	January
Hormonal or Targeted therapies	Female	January
Hormonal or Targeted therapies	Female	January
Hormonal or Targeted therapies	Male	January
Hormonal or Targeted therapies	Female	July
Hormonal or Targeted therapies	Male	July
Hormonal or Targeted therapies	Female	July
Hormonal or Targeted therapies	Male	July
Hormonal or Targeted therapies	Female	July
Hormonal or Targeted therapies	Female	July
Hormonal or Targeted therapies	Female	June
Hormonal or Targeted therapies	Female	June
Hormonal or Targeted therapies	Female	June
Hormonal or Targeted therapies	Male	June
Hormonal or Targeted therapies	Female	June
Hormonal or Targeted therapies	Male	June
Hormonal or Targeted therapies	Female	June
Hormonal or Targeted therapies	Female	March
Hormonal or Targeted therapies	Male	March
Hormonal or Targeted therapies	Male	March
Hormonal or Targeted therapies	Male	March
Hormonal or Targeted therapies	Female	March
Hormonal or Targeted therapies	Female	March
Hormonal or Targeted therapies	Female	May
Hormonal or Targeted therapies	Female	May
Hormonal or Targeted therapies	Male	May
Hormonal or Targeted therapies	Female	May
Hormonal or Targeted therapies	Female	May
Hormonal or Targeted therapies	Male	May
Hormonal or Targeted therapies	Female	May
Hormonal or Targeted therapies	Female	May
Hormonal or Targeted therapies	Male	May
Hormonal or Targeted therapies	Female	November
Hormonal or Targeted therapies	Female	November
Hormonal or Targeted therapies	Male	November
Hormonal or Targeted therapies	Female	November
Hormonal or Targeted therapies	Male	November
Hormonal or Targeted therapies	Male	November

Hormonal or Targeted therapies	Male	November
Hormonal or Targeted therapies	Female	November
Hormonal or Targeted therapies	Male	November
Hormonal or Targeted therapies	Female	November
Hormonal or Targeted therapies	Female	November
Hormonal or Targeted therapies	Female	October
Hormonal or Targeted therapies	Male	October
Hormonal or Targeted therapies	Female	October
Hormonal or Targeted therapies	Male	October
Hormonal or Targeted therapies	Male	October
Hormonal or Targeted therapies	Female	October
Hormonal or Targeted therapies	Female	September
Hormonal or Targeted therapies	Female	September
Hormonal or Targeted therapies	Male	September
Hormonal or Targeted therapies	Male	September
Hormonal or Targeted therapies	Female	September
Hormonal or Targeted therapies	Female	September
Hormonal or Targeted therapies	Female	September
Hormonal or Targeted therapies	Female	September
Hormonal or Targeted therapies	Male	September
Hormonal or Targeted therapies	Male	September
Hormonal or Targeted therapies	Female	September
Hormonal or Targeted therapies	Male	April
Hormonal or Targeted therapies	Female	April
Hormonal or Targeted therapies	Male	April
Hormonal or Targeted therapies	Male	April
Hormonal or Targeted therapies	Male	April
Hormonal or Targeted therapies	Male	April
Hormonal or Targeted therapies	Male	April
Hormonal or Targeted therapies	Female	April
Hormonal or Targeted therapies	Female	April
Hormonal or Targeted therapies	Female	April
Hormonal or Targeted therapies	Male	April
Hormonal or Targeted therapies	Male	August
Hormonal or Targeted therapies	Male	August
Hormonal or Targeted therapies	Female	August
Hormonal or Targeted therapies	Male	August
Hormonal or Targeted therapies	Male	December
Hormonal or Targeted therapies	Male	December
Hormonal or Targeted therapies	Male	December
Hormonal or Targeted therapies	Male	December
Hormonal or Targeted therapies	Female	December
Hormonal or Targeted therapies	Male	December
Hormonal or Targeted therapies	Male	December
Hormonal or Targeted therapies	Female	December
Hormonal or Targeted therapies	Male	December
Hormonal or Targeted therapies	Female	February
Hormonal or Targeted therapies	Male	February
Hormonal or Targeted therapies	Male	February
Hormonal or Targeted therapies	Male	February

Hormonal or Targeted therapies	Female	February
Hormonal or Targeted therapies	Male	February
Hormonal or Targeted therapies	Female	February
Hormonal or Targeted therapies	Female	February
Hormonal or Targeted therapies	Male	February
Hormonal or Targeted therapies	Male	February
Hormonal or Targeted therapies	Male	February
Hormonal or Targeted therapies	Male	February
Hormonal or Targeted therapies	Male	January
Hormonal or Targeted therapies	Male	January
Hormonal or Targeted therapies	Female	January
Hormonal or Targeted therapies	Female	January
Hormonal or Targeted therapies	Female	January
Hormonal or Targeted therapies	Male	January
Hormonal or Targeted therapies	Male	January
Hormonal or Targeted therapies	Male	January
Hormonal or Targeted therapies	Male	January
Hormonal or Targeted therapies	Female	January
Hormonal or Targeted therapies	Female	July
Hormonal or Targeted therapies	Male	July
Hormonal or Targeted therapies	Male	July
Hormonal or Targeted therapies	Male	July
Hormonal or Targeted therapies	Female	July
Hormonal or Targeted therapies	Male	July
Hormonal or Targeted therapies	Female	July
Hormonal or Targeted therapies	Male	June
Hormonal or Targeted therapies	Male	June
Hormonal or Targeted therapies	Female	June
Hormonal or Targeted therapies	Male	June
Hormonal or Targeted therapies	Female	June
Hormonal or Targeted therapies	Male	June
Hormonal or Targeted therapies	Male	June
Hormonal or Targeted therapies	Male	March
Hormonal or Targeted therapies	Female	March
Hormonal or Targeted therapies	Male	March
Hormonal or Targeted therapies	Male	March
Hormonal or Targeted therapies	Male	March
Hormonal or Targeted therapies	Male	March
Hormonal or Targeted therapies	Female	March
Hormonal or Targeted therapies	Male	March
Hormonal or Targeted therapies	Female	May
Hormonal or Targeted therapies	Male	May
Hormonal or Targeted therapies	Male	May
Hormonal or Targeted therapies	Male	May
Hormonal or Targeted therapies	Male	May
Hormonal or Targeted therapies	Female	May
Hormonal or Targeted therapies	Male	May
Hormonal or Targeted therapies	Female	November
Hormonal or Targeted therapies	Male	November
Hormonal or Targeted therapies	Female	November

Hormonal or Targeted therapies	Female	November
Hormonal or Targeted therapies	Male	November
Hormonal or Targeted therapies	Male	November
Hormonal or Targeted therapies	Male	November
Hormonal or Targeted therapies	Male	November
Hormonal or Targeted therapies	Female	November
Hormonal or Targeted therapies	Male	November
Hormonal or Targeted therapies	Male	November
Hormonal or Targeted therapies	Male	October
Hormonal or Targeted therapies	Male	October
Hormonal or Targeted therapies	Male	October
Hormonal or Targeted therapies	Male	October
Hormonal or Targeted therapies	Male	October
Hormonal or Targeted therapies	Female	October
Hormonal or Targeted therapies	Male	October
Hormonal or Targeted therapies	Male	October
Hormonal or Targeted therapies	Male	September
Hormonal or Targeted therapies	Female	September
Hormonal or Targeted therapies	Male	September
Hormonal or Targeted therapies	Female	September
Hormonal or Targeted therapies	Male	September
Hormonal or Targeted therapies	Female	September
Hormonal or Targeted therapies	Male	September
Hormonal or Targeted therapies	Male	September
Hormonal or Targeted therapies	Male	September
Hormonal or Targeted therapies	Female	April
Hormonal or Targeted therapies	Female	April
Hormonal or Targeted therapies	Male	April
Hormonal or Targeted therapies	Female	April
Hormonal or Targeted therapies	Male	April
Hormonal or Targeted therapies	Male	April
Hormonal or Targeted therapies	Female	April
Hormonal or Targeted therapies	Female	April
Hormonal or Targeted therapies	Female	August
Hormonal or Targeted therapies	Female	August
Hormonal or Targeted therapies	Male	August
Hormonal or Targeted therapies	Female	August
Hormonal or Targeted therapies	Male	August
Hormonal or Targeted therapies	Female	August
Hormonal or Targeted therapies	Male	August
Hormonal or Targeted therapies	Male	August
Hormonal or Targeted therapies	Female	August
Hormonal or Targeted therapies	Female	December
Hormonal or Targeted therapies	Female	December
Hormonal or Targeted therapies	Male	December
Hormonal or Targeted therapies	Male	December
Hormonal or Targeted therapies	Male	December
Hormonal or Targeted therapies	Female	December
Hormonal or Targeted therapies	Female	December
Hormonal or Targeted therapies	Male	December

Hormonal or Targeted therapies	Male	February
Hormonal or Targeted therapies	Female	February
Hormonal or Targeted therapies	Female	February
Hormonal or Targeted therapies	Female	February
Hormonal or Targeted therapies	Male	February
Hormonal or Targeted therapies	Female	February
Hormonal or Targeted therapies	Male	February
Hormonal or Targeted therapies	Male	February
Hormonal or Targeted therapies	Female	February
Hormonal or Targeted therapies	Male	January
Hormonal or Targeted therapies	Male	January
Hormonal or Targeted therapies	Male	January
Hormonal or Targeted therapies	Female	January
Hormonal or Targeted therapies	Female	January
Hormonal or Targeted therapies	Male	January
Hormonal or Targeted therapies	Female	January
Hormonal or Targeted therapies	Female	January
Hormonal or Targeted therapies	Female	January
Hormonal or Targeted therapies	Male	January
Hormonal or Targeted therapies	Female	July
Hormonal or Targeted therapies	Female	July
Hormonal or Targeted therapies	Male	July
Hormonal or Targeted therapies	Male	July
Hormonal or Targeted therapies	Male	July
Hormonal or Targeted therapies	Male	July
Hormonal or Targeted therapies	Male	July
Hormonal or Targeted therapies	Female	June
Hormonal or Targeted therapies	Female	June
Hormonal or Targeted therapies	Male	June
Hormonal or Targeted therapies	Female	June
Hormonal or Targeted therapies	Female	June
Hormonal or Targeted therapies	Male	June
Hormonal or Targeted therapies	Male	June
Hormonal or Targeted therapies	Male	March
Hormonal or Targeted therapies	Female	March
Hormonal or Targeted therapies	Female	March
Hormonal or Targeted therapies	Male	March
Hormonal or Targeted therapies	Male	March
Hormonal or Targeted therapies	Male	March
Hormonal or Targeted therapies	Male	March
Hormonal or Targeted therapies	Female	March
Hormonal or Targeted therapies	Female	May
Hormonal or Targeted therapies	Male	May
Hormonal or Targeted therapies	Female	May
Hormonal or Targeted therapies	Male	May
Hormonal or Targeted therapies	Female	May
Hormonal or Targeted therapies	Female	May
Hormonal or Targeted therapies	Female	May
Hormonal or Targeted therapies	Male	May
Hormonal or Targeted therapies	Female	May

Hormonal or Targeted therapies	Male	November
Hormonal or Targeted therapies	Female	November
Hormonal or Targeted therapies	Female	November
Hormonal or Targeted therapies	Male	November
Hormonal or Targeted therapies	Male	November
Hormonal or Targeted therapies	Female	November
Hormonal or Targeted therapies	Male	November
Hormonal or Targeted therapies	Female	November
Hormonal or Targeted therapies	Male	October
Hormonal or Targeted therapies	Female	October
Hormonal or Targeted therapies	Female	October
Hormonal or Targeted therapies	Male	October
Hormonal or Targeted therapies	Male	October
Hormonal or Targeted therapies	Female	October
Hormonal or Targeted therapies	Female	October
Hormonal or Targeted therapies	Female	September
Hormonal or Targeted therapies	Female	September
Hormonal or Targeted therapies	Male	September
Hormonal or Targeted therapies	Female	September
Hormonal or Targeted therapies	Male	September
Hormonal or Targeted therapies	Male	September
Hormonal or Targeted therapies	Male	September
Hormonal or Targeted therapies	Female	September
Hormonal or Targeted therapies	Male	April
Hormonal or Targeted therapies	Female	April
Hormonal or Targeted therapies	Male	April
Hormonal or Targeted therapies	Female	April
Hormonal or Targeted therapies	Male	April
Hormonal or Targeted therapies	Female	April
Hormonal or Targeted therapies	Male	April
Hormonal or Targeted therapies	Female	April
Hormonal or Targeted therapies	Male	April
Hormonal or Targeted therapies	Male	April
Hormonal or Targeted therapies	Female	April
Hormonal or Targeted therapies	Male	April
Hormonal or Targeted therapies	Female	April
Hormonal or Targeted therapies	Female	April
Hormonal or Targeted therapies	Male	August
Hormonal or Targeted therapies	Female	August
Hormonal or Targeted therapies	Female	August
Hormonal or Targeted therapies	Female	August
Hormonal or Targeted therapies	Female	August
Hormonal or Targeted therapies	Male	August
Hormonal or Targeted therapies	Male	August
Hormonal or Targeted therapies	Male	August
Hormonal or Targeted therapies	Female	August
Hormonal or Targeted therapies	Male	August
Hormonal or Targeted therapies	Male	December
Hormonal or Targeted therapies	Female	December
Hormonal or Targeted therapies	Male	December

Hormonal or Targeted therapies	Female	December
Hormonal or Targeted therapies	Female	December
Hormonal or Targeted therapies	Male	December
Hormonal or Targeted therapies	Female	December
Hormonal or Targeted therapies	Male	December
Hormonal or Targeted therapies	Female	December
Hormonal or Targeted therapies	Male	December
Hormonal or Targeted therapies	Female	December
Hormonal or Targeted therapies	Female	February
Hormonal or Targeted therapies	Male	February
Hormonal or Targeted therapies	Female	February
Hormonal or Targeted therapies	Male	February
Hormonal or Targeted therapies	Female	February
Hormonal or Targeted therapies	Male	February
Hormonal or Targeted therapies	Female	February
Hormonal or Targeted therapies	Male	February
Hormonal or Targeted therapies	Male	February
Hormonal or Targeted therapies	Female	February
Hormonal or Targeted therapies	Male	February
Hormonal or Targeted therapies	Female	February
Hormonal or Targeted therapies	Male	February
Hormonal or Targeted therapies	Female	February
Hormonal or Targeted therapies	Male	February
Hormonal or Targeted therapies	Male	January
Hormonal or Targeted therapies	Male	January
Hormonal or Targeted therapies	Female	January
Hormonal or Targeted therapies	Female	January
Hormonal or Targeted therapies	Male	January
Hormonal or Targeted therapies	Female	January
Hormonal or Targeted therapies	Male	January
Hormonal or Targeted therapies	Female	January
Hormonal or Targeted therapies	Male	January
Hormonal or Targeted therapies	Female	January
Hormonal or Targeted therapies	Male	January
Hormonal or Targeted therapies	Male	January
Hormonal or Targeted therapies	Male	January
Hormonal or Targeted therapies	Female	January
Hormonal or Targeted therapies	Female	January
Hormonal or Targeted therapies	Female	July
Hormonal or Targeted therapies	Male	July
Hormonal or Targeted therapies	Male	July
Hormonal or Targeted therapies	Female	July
Hormonal or Targeted therapies	Male	July
Hormonal or Targeted therapies	Female	July
Hormonal or Targeted therapies	Female	July
Hormonal or Targeted therapies	Male	July
Hormonal or Targeted therapies	Male	July
Hormonal or Targeted therapies	Female	July
Hormonal or Targeted therapies	Male	July
Hormonal or Targeted therapies	Male	July

Hormonal or Targeted therapies	Female	June
Hormonal or Targeted therapies	Male	June
Hormonal or Targeted therapies	Male	June
Hormonal or Targeted therapies	Female	June
Hormonal or Targeted therapies	Female	June
Hormonal or Targeted therapies	Male	June
Hormonal or Targeted therapies	Female	June
Hormonal or Targeted therapies	Male	June
Hormonal or Targeted therapies	Female	June
Hormonal or Targeted therapies	Male	June
Hormonal or Targeted therapies	Female	June
Hormonal or Targeted therapies	Male	June
Hormonal or Targeted therapies	Female	March
Hormonal or Targeted therapies	Male	March
Hormonal or Targeted therapies	Female	March
Hormonal or Targeted therapies	Male	March
Hormonal or Targeted therapies	Male	March
Hormonal or Targeted therapies	Female	March
Hormonal or Targeted therapies	Male	March
Hormonal or Targeted therapies	Male	March
Hormonal or Targeted therapies	Male	March
Hormonal or Targeted therapies	Female	March
Hormonal or Targeted therapies	Female	May
Hormonal or Targeted therapies	Male	May
Hormonal or Targeted therapies	Male	May
Hormonal or Targeted therapies	Male	May
Hormonal or Targeted therapies	Female	May
Hormonal or Targeted therapies	Female	May
Hormonal or Targeted therapies	Male	May
Hormonal or Targeted therapies	Male	May
Hormonal or Targeted therapies	Male	May
Hormonal or Targeted therapies	Male	May
Hormonal or Targeted therapies	Female	May
Hormonal or Targeted therapies	Male	May
Hormonal or Targeted therapies	Female	November
Hormonal or Targeted therapies	Female	November
Hormonal or Targeted therapies	Male	November
Hormonal or Targeted therapies	Female	November
Hormonal or Targeted therapies	Male	November
Hormonal or Targeted therapies	Male	November
Hormonal or Targeted therapies	Male	November
Hormonal or Targeted therapies	Female	November
Hormonal or Targeted therapies	Male	November
Hormonal or Targeted therapies	Female	November
Hormonal or Targeted therapies	Female	November
Hormonal or Targeted therapies	Male	November
Hormonal or Targeted therapies	Female	November
Hormonal or Targeted therapies	Male	October
Hormonal or Targeted therapies	Male	October
Hormonal or Targeted therapies	Male	October

Hormonal or Targeted therapies	Male	October
Hormonal or Targeted therapies	Female	October
Hormonal or Targeted therapies	Female	October
Hormonal or Targeted therapies	Male	October
Hormonal or Targeted therapies	Female	October
Hormonal or Targeted therapies	Male	October
Hormonal or Targeted therapies	Male	October
Hormonal or Targeted therapies	Female	October
Hormonal or Targeted therapies	Female	September
Hormonal or Targeted therapies	Male	September
Hormonal or Targeted therapies	Female	September
Hormonal or Targeted therapies	Male	September
Hormonal or Targeted therapies	Female	September
Hormonal or Targeted therapies	Female	September
Hormonal or Targeted therapies	Male	September
Hormonal or Targeted therapies	Male	September
Hormonal or Targeted therapies	Male	September
Hormonal or Targeted therapies	Female	September
Hormonal or Targeted therapies	Male	September
Hormonal or Targeted therapies	Female	September
Surgery	Female	April
Surgery	Female	April
Surgery	Male	April
Surgery	Female	April
Surgery	Male	April
Surgery	Male	April
Surgery	Female	April
Surgery	Male	April
Surgery	Male	April
Surgery	Female	April
Surgery	Male	April
Surgery	Female	April
Surgery	Female	April
Surgery	Male	April
Surgery	Female	April
Surgery	Male	April
Surgery	Female	April
Surgery	Female	April
Surgery	Male	April
Surgery	Female	August
Surgery	Male	August
Surgery	Female	August
Surgery	Male	August
Surgery	Male	December
Surgery	Female	December
Surgery	Female	December
Surgery	Male	December
Surgery	Male	December
Surgery	Male	February
Surgery	Male	February

Surgery	Female	February
Surgery	Female	February
Surgery	Male	February
Surgery	Female	February
Surgery	Male	February
Surgery	Male	February
Surgery	Female	February
Surgery	Male	February
Surgery	Female	February
Surgery	Male	February
Surgery	Female	February
Surgery	Female	February
Surgery	Male	February
Surgery	Female	February
Surgery	Female	February
Surgery	Male	February
Surgery	Male	February
Surgery	Female	February
Surgery	Female	January
Surgery	Male	January
Surgery	Female	January
Surgery	Male	January
Surgery	Male	January
Surgery	Female	January
Surgery	Male	January
Surgery	Male	January
Surgery	Female	January
Surgery	Female	January
Surgery	Male	January
Surgery	Female	January
Surgery	Male	January
Surgery	Female	January
Surgery	Male	January
Surgery	Male	January
Surgery	Female	January
Surgery	Female	January
Surgery	Female	January
Surgery	Male	January
Surgery	Male	July
Surgery	Male	July
Surgery	Male	July
Surgery	Female	July
Surgery	Female	July
Surgery	Female	June
Surgery	Female	June
Surgery	Male	June
Surgery	Male	June
Surgery	Female	June
Surgery	Male	June
Surgery	Male	March

Surgery	Male	March
Surgery	Male	March
Surgery	Female	March
Surgery	Female	March
Surgery	Male	May
Surgery	Female	May
Surgery	Male	May
Surgery	Female	May
Surgery	Female	November
Surgery	Female	November
Surgery	Male	November
Surgery	Female	November
Surgery	Female	November
Surgery	Male	November
Surgery	Female	November
Surgery	Male	November
Surgery	Male	October
Surgery	Female	October
Surgery	Female	October
Surgery	Male	October
Surgery	Male	September
Surgery	Female	April
Surgery	Female	April
Surgery	Female	April
Surgery	Male	April
Surgery	Female	April
Surgery	Male	April
Surgery	Female	August
Surgery	Female	August
Surgery	Male	August
Surgery	Female	August
Surgery	Male	August
Surgery	Female	August
Surgery	Female	December
Surgery	Female	December
Surgery	Male	December
Surgery	Male	December
Surgery	Female	December
Surgery	Female	December
Surgery	Female	February
Surgery	Male	February
Surgery	Male	February
Surgery	Female	February
Surgery	Female	February
Surgery	Female	February
Surgery	Female	January
Surgery	Male	January
Surgery	Male	January
Surgery	Female	January
Surgery	Female	January

Surgery	Female	January
Surgery	Female	July
Surgery	Male	July
Surgery	Female	July
Surgery	Female	July
Surgery	Male	July
Surgery	Male	June
Surgery	Female	June
Surgery	Female	June
Surgery	Female	June
Surgery	Male	June
Surgery	Female	June
Surgery	Female	March
Surgery	Female	May
Surgery	Male	May
Surgery	Male	May
Surgery	Female	May
Surgery	Female	May
Surgery	Female	May
Surgery	Female	November
Surgery	Female	November
Surgery	Female	November
Surgery	Male	November
Surgery	Male	November
Surgery	Female	November
Surgery	Female	October
Surgery	Male	October
Surgery	Female	October
Surgery	Male	October
Surgery	Female	October
Surgery	Female	October
Surgery	Female	September
Surgery	Female	September
Surgery	Female	September
Surgery	Male	September
Surgery	Male	September
Surgery	Female	September
Surgery	Male	April
Surgery	Male	April
Surgery	Female	April
Surgery	Female	April
Surgery	Male	April
Surgery	Female	April
Surgery	Female	April
Surgery	Female	April
Surgery	Female	August
Surgery	Female	August
Surgery	Female	August
Surgery	Male	August
Surgery	Female	August

Surgery	Male	August
Surgery	Male	December
Surgery	Female	December
Surgery	Female	December
Surgery	Male	December
Surgery	Female	December
Surgery	Male	December
Surgery	Female	December
Surgery	Female	December
Surgery	Male	February
Surgery	Female	February
Surgery	Female	February
Surgery	Female	February
Surgery	Female	February
Surgery	Male	February
Surgery	Female	February
Surgery	Male	January
Surgery	Female	January
Surgery	Female	January
Surgery	Female	January
Surgery	Female	January
Surgery	Male	January
Surgery	Male	January
Surgery	Male	January
Surgery	Female	July
Surgery	Female	July
Surgery	Male	July
Surgery	Female	July
Surgery	Female	July
Surgery	Male	July
Surgery	Male	June
Surgery	Female	June
Surgery	Male	June
Surgery	Male	June
Surgery	Female	June
Surgery	Female	June
Surgery	Female	June
Surgery	Female	March
Surgery	Female	March
Surgery	Female	March
Surgery	Female	March
Surgery	Female	March
Surgery	Male	March
Surgery	Female	March
Surgery	Female	May
Surgery	Female	May
Surgery	Male	May
Surgery	Male	May
Surgery	Female	May
Surgery	Male	May

Surgery	Female	May
Surgery	Female	May
Surgery	Female	November
Surgery	Male	November
Surgery	Female	November
Surgery	Female	November
Surgery	Female	November
Surgery	Female	November
Surgery	Male	October
Surgery	Male	October
Surgery	Female	October
Surgery	Female	October
Surgery	Female	October
Surgery	Male	October
Surgery	Female	October
Surgery	Female	October
Surgery	Female	October
Surgery	Female	October
Surgery	Female	September
Surgery	Male	September
Surgery	Female	September
Surgery	Female	September
Surgery	Female	September
Surgery	Female	September
Surgery	Female	April
Surgery	Male	April
Surgery	Male	April
Surgery	Female	April
Surgery	Male	April
Surgery	Female	August
Surgery	Male	August
Surgery	Male	August
Surgery	Female	August
Surgery	Male	August
Surgery	Female	August
Surgery	Female	August
Surgery	Female	August
Surgery	Male	August
Surgery	Female	August
Surgery	Male	August
Surgery	Male	August
Surgery	Male	August
Surgery	Female	August
Surgery	Female	August
Surgery	Male	August
Surgery	Male	December
Surgery	Female	December
Surgery	Male	December
Surgery	Female	December
Surgery	Female	December

Surgery	Male	December
Surgery	Female	December
Surgery	Male	December
Surgery	Female	December
Surgery	Male	December
Surgery	Female	December
Surgery	Male	December
Surgery	Female	December
Surgery	Male	December
Surgery	Female	December
Surgery	Male	December
Surgery	Male	July
Surgery	Female	July
Surgery	Female	June
Surgery	Female	June
Surgery	Female	March
Surgery	Male	March
Surgery	Female	March
Surgery	Female	March
Surgery	Male	March
Surgery	Male	March
Surgery	Female	March
Surgery	Male	March
Surgery	Female	March
Surgery	Male	March
Surgery	Female	March
Surgery	Male	March
Surgery	Female	March
Surgery	Male	March
Surgery	Male	March
Surgery	Female	March
Surgery	Male	May
Surgery	Male	November
Surgery	Female	November
Surgery	Male	November
Surgery	Female	November
Surgery	Male	November
Surgery	Female	November
Surgery	Male	November
Surgery	Male	November
Surgery	Female	November
Surgery	Male	November
Surgery	Female	November
Surgery	Male	November
Surgery	Female	November
Surgery	Male	November
Surgery	Female	November
Surgery	Female	November
Surgery	Male	October
Surgery	Female	October

Surgery	Male	September
Surgery	Female	September
Surgery	Female	September
Surgery	Male	September
Surgery	Female	September
Surgery	Male	September
Surgery	Male	September
Surgery	Male	September
Surgery	Female	September
Surgery	Female	September
Surgery	Male	September
Surgery	Female	September
Surgery	Male	September
Surgery	Female	September
Surgery	Female	September
Surgery	Male	September
Surgery	Female	April
Surgery	Male	April
Surgery	Female	August
Surgery	Female	August
Surgery	Male	December
Surgery	Female	December
Surgery	Male	December
Surgery	Female	December
Surgery	Female	February
Surgery	Male	February
Surgery	Female	February
Surgery	Male	February
Surgery	Male	February
Surgery	Female	February
Surgery	Male	February
Surgery	Female	February
Surgery	Female	February
Surgery	Male	February
Surgery	Male	February
Surgery	Female	February
Surgery	Female	February
Surgery	Male	January
Surgery	Female	January
Surgery	Male	January
Surgery	Female	January
Surgery	Female	January
Surgery	Male	January
Surgery	Male	January
Surgery	Male	January
Surgery	Female	January
Surgery	Female	January
Surgery	Female	January
Surgery	Female	January
Surgery	Male	January

Surgery	Male	July
Surgery	Male	July
Surgery	Male	July
Surgery	Male	July
Surgery	Female	July
Surgery	Female	July
Surgery	Female	July
Surgery	Female	July
Surgery	Male	July
Surgery	Female	July
Surgery	Female	July
Surgery	Female	July
Surgery	Male	July
Surgery	Male	June
Surgery	Female	June
Surgery	Female	March
Surgery	Male	March
Surgery	Female	March
Surgery	Male	March
Surgery	Male	March
Surgery	Female	March
Surgery	Female	March
Surgery	Female	March
Surgery	Male	March
Surgery	Female	March
Surgery	Male	March
Surgery	Female	March
Surgery	Male	March
Surgery	Male	May
Surgery	Female	May
Surgery	Female	May
Surgery	Male	November
Surgery	Male	November
Surgery	Female	November
Surgery	Female	November
Surgery	Male	November
Surgery	Female	November
Surgery	Male	November
Surgery	Female	November
Surgery	Male	November
Surgery	Female	November
Surgery	Female	November
Surgery	Female	November
Surgery	Male	November
Surgery	Male	October
Surgery	Female	October
Surgery	Female	September
Surgery	Female	September
Surgery	Male	September
Surgery	Female	September

Surgery	Male	September
Surgery	Female	September
Surgery	Male	September
Surgery	Female	September
Surgery	Male	September
Surgery	Female	September
Surgery	Male	September
Surgery	Male	September
Surgery	Female	September
Surgery	Male	April
Surgery	Female	April
Surgery	Male	April
Surgery	Female	April
Surgery	Male	April
Surgery	Female	April
Surgery	Female	April
Surgery	Female	April
Surgery	Male	April
Surgery	Female	August
Surgery	Female	August
Surgery	Female	August
Surgery	Male	August
Surgery	Female	August
Surgery	Male	August
Surgery	Male	August
Surgery	Male	August
Surgery	Female	August
Surgery	Male	December
Surgery	Female	December
Surgery	Male	December
Surgery	Female	December
Surgery	Male	December
Surgery	Female	December
Surgery	Female	December
Surgery	Female	December
Surgery	Male	December
Surgery	Female	February
Surgery	Male	February
Surgery	Female	February
Surgery	Male	February
Surgery	Male	February
Surgery	Male	February
Surgery	Female	February
Surgery	Female	February
Surgery	Female	February
Surgery	Male	January
Surgery	Female	January
Surgery	Female	January
Surgery	Female	January
Surgery	Female	January

Surgery	Male	January
Surgery	Female	January
Surgery	Male	January
Surgery	Male	January
Surgery	Male	July
Surgery	Female	July
Surgery	Male	July
Surgery	Female	July
Surgery	Female	July
Surgery	Male	July
Surgery	Female	June
Surgery	Male	June
Surgery	Female	June
Surgery	Male	June
Surgery	Female	June
Surgery	Female	June
Surgery	Male	June
Surgery	Female	June
Surgery	Male	June
Surgery	Female	March
Surgery	Male	March
Surgery	Male	March
Surgery	Female	March
Surgery	Female	March
Surgery	Female	March
Surgery	Male	March
Surgery	Female	March
Surgery	Male	March
Surgery	Male	May
Surgery	Male	May
Surgery	Female	May
Surgery	Female	May
Surgery	Female	May
Surgery	Female	May
Surgery	Male	May
Surgery	Female	May
Surgery	Male	May
Surgery	Male	November
Surgery	Female	November
Surgery	Male	November
Surgery	Male	November
Surgery	Female	November
Surgery	Female	November
Surgery	Male	November
Surgery	Female	October
Surgery	Male	October
Surgery	Female	October
Surgery	Male	October
Surgery	Female	October
Surgery	Female	October

Surgery	Female	September
Surgery	Female	September
Surgery	Female	September
Surgery	Female	September
Surgery	Male	September
Radiotherapy	Male	April
Radiotherapy	Female	April
Radiotherapy	Male	April
Radiotherapy	Female	April
Radiotherapy	Male	April
Radiotherapy	Female	April
Radiotherapy	Female	April
Radiotherapy	Male	April
Radiotherapy	Male	April
Radiotherapy	Female	April
Radiotherapy	Female	April
Radiotherapy	Male	April
Radiotherapy	Female	April
Radiotherapy	Male	April
Radiotherapy	Female	February
Radiotherapy	Male	February
Radiotherapy	Female	February
Radiotherapy	Male	February
Radiotherapy	Male	February
Radiotherapy	Female	February
Radiotherapy	Male	February
Radiotherapy	Female	February
Radiotherapy	Male	February
Radiotherapy	Female	February
Radiotherapy	Male	February
Radiotherapy	Male	February
Radiotherapy	Female	February
Radiotherapy	Female	February
Radiotherapy	Female	January
Radiotherapy	Male	January
Radiotherapy	Male	January
Radiotherapy	Female	January
Radiotherapy	Male	January
Radiotherapy	Female	January
Radiotherapy	Female	January
Radiotherapy	Male	January
Radiotherapy	Male	January
Radiotherapy	Female	January
Radiotherapy	Male	January
Radiotherapy	Female	January
Radiotherapy	Male	January
Radiotherapy	Female	January
Radiotherapy	Female	July
Radiotherapy	Female	July
Radiotherapy	Male	July

Radiotherapy	Female	July
Radiotherapy	Male	July
Radiotherapy	Male	July
Radiotherapy	Male	July
Radiotherapy	Female	July
Radiotherapy	Male	July
Radiotherapy	Female	July
Radiotherapy	Female	July
Radiotherapy	Male	July
Radiotherapy	Male	July
Radiotherapy	Female	July
Radiotherapy	Female	June
Radiotherapy	Female	June
Radiotherapy	Male	June
Radiotherapy	Male	June
Radiotherapy	Female	June
Radiotherapy	Female	June
Radiotherapy	Male	June
Radiotherapy	Female	June
Radiotherapy	Male	June
Radiotherapy	Male	June
Radiotherapy	Female	June
Radiotherapy	Female	June
Radiotherapy	Male	June
Radiotherapy	Male	June
Radiotherapy	Female	March
Radiotherapy	Male	March
Radiotherapy	Female	March
Radiotherapy	Female	March
Radiotherapy	Female	March
Radiotherapy	Male	March
Radiotherapy	Male	March
Radiotherapy	Male	March
Radiotherapy	Male	March
Radiotherapy	Male	March
Radiotherapy	Female	March
Radiotherapy	Female	March
Radiotherapy	Female	March
Radiotherapy	Male	March
Radiotherapy	Male	May
Radiotherapy	Male	May
Radiotherapy	Female	May
Radiotherapy	Female	May
Radiotherapy	Female	May
Radiotherapy	Male	May
Radiotherapy	Female	May
Radiotherapy	Male	May
Radiotherapy	Male	May
Radiotherapy	Female	May
Radiotherapy	Male	May

Radiotherapy	Male	May
Radiotherapy	Female	May
Radiotherapy	Female	May
Radiotherapy	Male	November
Radiotherapy	Female	November
Radiotherapy	Female	November
Radiotherapy	Male	November
Radiotherapy	Male	November
Radiotherapy	Male	November
Radiotherapy	Male	November
Radiotherapy	Female	November
Radiotherapy	Female	November
Radiotherapy	Male	November
Radiotherapy	Female	November
Radiotherapy	Female	November
Radiotherapy	Male	November
Radiotherapy	Female	November
Radiotherapy	Female	October
Radiotherapy	Male	October
Radiotherapy	Male	October
Radiotherapy	Male	October
Radiotherapy	Female	October
Radiotherapy	Female	October
Radiotherapy	Male	October
Radiotherapy	Female	October
Radiotherapy	Male	October
Radiotherapy	Female	October
Radiotherapy	Female	October
Radiotherapy	Male	October
Radiotherapy	Female	October
Radiotherapy	Male	October
Radiotherapy	Female	September
Radiotherapy	Female	September
Radiotherapy	Male	September
Radiotherapy	Female	September
Radiotherapy	Male	September
Radiotherapy	Male	September
Radiotherapy	Female	September
Radiotherapy	Female	September
Radiotherapy	Male	September
Radiotherapy	Female	April
Radiotherapy	Female	April
Radiotherapy	Male	April
Radiotherapy	Female	April
Radiotherapy	Male	April
Radiotherapy	Female	April
Radiotherapy	Male	April
Radiotherapy	Male	April
Radiotherapy	Male	August
Radiotherapy	Female	August

Radiotherapy	Female	December
Radiotherapy	Female	December
Radiotherapy	Male	December
Radiotherapy	Female	December
Radiotherapy	Male	December
Radiotherapy	Female	December
Radiotherapy	Male	December
Radiotherapy	Male	December
Radiotherapy	Male	February
Radiotherapy	Female	February
Radiotherapy	Male	February
Radiotherapy	Female	February
Radiotherapy	Female	February
Radiotherapy	Male	February
Radiotherapy	Female	February
Radiotherapy	Male	February
Radiotherapy	Male	January
Radiotherapy	Female	January
Radiotherapy	Male	January
Radiotherapy	Female	January
Radiotherapy	Male	January
Radiotherapy	Female	January
Radiotherapy	Female	January
Radiotherapy	Male	January
Radiotherapy	Female	June
Radiotherapy	Male	June
Radiotherapy	Female	June
Radiotherapy	Male	June
Radiotherapy	Female	June
Radiotherapy	Female	June
Radiotherapy	Male	June
Radiotherapy	Male	June
Radiotherapy	Male	March
Radiotherapy	Male	March
Radiotherapy	Male	March
Radiotherapy	Female	March
Radiotherapy	Female	March
Radiotherapy	Female	March
Radiotherapy	Male	March
Radiotherapy	Female	March
Radiotherapy	Female	May
Radiotherapy	Male	May
Radiotherapy	Male	May
Radiotherapy	Female	May
Radiotherapy	Female	May
Radiotherapy	Male	May
Radiotherapy	Female	May
Radiotherapy	Male	May
Radiotherapy	Female	November
Radiotherapy	Male	November

Radiotherapy	Male	November
Radiotherapy	Female	November
Radiotherapy	Female	November
Radiotherapy	Male	November
Radiotherapy	Male	November
Radiotherapy	Female	November
Radiotherapy	Female	October
Radiotherapy	Male	October
Radiotherapy	Female	October
Radiotherapy	Male	October
Radiotherapy	Male	October
Radiotherapy	Female	October
Radiotherapy	Female	October
Radiotherapy	Male	October
Radiotherapy	Male	September
Radiotherapy	Female	September
Radiotherapy	Male	September
Radiotherapy	Female	September
Radiotherapy	Female	September
Radiotherapy	Male	September
Radiotherapy	Female	September
Radiotherapy	Male	September
Radiotherapy	Male	April
Radiotherapy	Female	April
Radiotherapy	Male	April
Radiotherapy	Male	April
Radiotherapy	Female	April
Radiotherapy	Female	April
Radiotherapy	Male	August
Radiotherapy	Male	August
Radiotherapy	Female	August
Radiotherapy	Female	August
Radiotherapy	Male	August
Radiotherapy	Female	December
Radiotherapy	Male	December
Radiotherapy	Female	December
Radiotherapy	Male	December
Radiotherapy	Male	December
Radiotherapy	Female	December
Radiotherapy	Male	February
Radiotherapy	Male	February
Radiotherapy	Female	February
Radiotherapy	Female	February
Radiotherapy	Female	February
Radiotherapy	Male	February
Radiotherapy	Female	January
Radiotherapy	Female	January
Radiotherapy	Female	January
Radiotherapy	Male	January
Radiotherapy	Male	January

Radiotherapy	Male	January
Radiotherapy	Female	July
Radiotherapy	Male	July
Radiotherapy	Male	July
Radiotherapy	Female	July
Radiotherapy	Male	July
Radiotherapy	Male	June
Radiotherapy	Female	June
Radiotherapy	Female	June
Radiotherapy	Male	June
Radiotherapy	Male	June
Radiotherapy	Female	June
Radiotherapy	Female	March
Radiotherapy	Female	March
Radiotherapy	Male	March
Radiotherapy	Male	March
Radiotherapy	Male	March
Radiotherapy	Female	March
Radiotherapy	Male	May
Radiotherapy	Female	May
Radiotherapy	Female	May
Radiotherapy	Male	May
Radiotherapy	Male	May
Radiotherapy	Female	November
Radiotherapy	Female	November
Radiotherapy	Male	November
Radiotherapy	Female	October
Radiotherapy	Male	October
Radiotherapy	Female	October
Radiotherapy	Male	October
Radiotherapy	Male	September
Radiotherapy	Female	September
Radiotherapy	Female	September
Radiotherapy	Male	September
Radiotherapy	Female	September
Radiotherapy	Male	September
Radiotherapy	Male	April
Radiotherapy	Female	April
Radiotherapy	Female	August
Radiotherapy	Male	August
Radiotherapy	Female	August
Radiotherapy	Male	August
Radiotherapy	Female	August
Radiotherapy	Male	August
Radiotherapy	Male	August
Radiotherapy	Female	August
Radiotherapy	Male	August
Radiotherapy	Female	August
Radiotherapy	Female	August
Radiotherapy	Male	August

Radiotherapy	Female	August
Radiotherapy	Male	August
Radiotherapy	Female	August
Radiotherapy	Male	August
Radiotherapy	Male	December
Radiotherapy	Male	December
Radiotherapy	Female	December
Radiotherapy	Female	February
Radiotherapy	Male	January
Radiotherapy	Female	January
Radiotherapy	Male	July
Radiotherapy	Female	July
Radiotherapy	Female	July
Radiotherapy	Female	July
Radiotherapy	Female	July
Radiotherapy	Female	July
Radiotherapy	Male	July
Radiotherapy	Male	July
Radiotherapy	Male	July
Radiotherapy	Female	July
Radiotherapy	Male	July
Radiotherapy	Female	July
Radiotherapy	Male	July
Radiotherapy	Male	July
Radiotherapy	Female	July
Radiotherapy	Male	July
Radiotherapy	Female	June
Radiotherapy	Male	June
Radiotherapy	Female	March
Radiotherapy	Male	March
Radiotherapy	Male	May
Radiotherapy	Female	May
Radiotherapy	Male	November
Radiotherapy	Female	October
Radiotherapy	Male	October
Radiotherapy	Female	October
Radiotherapy	Male	September
Radiotherapy	Female	September
Radiotherapy	Female	September
Radiotherapy	Male	September
Nuclear medicine or Radioiodine treatment	Male	April
Nuclear medicine or Radioiodine treatment	Female	April
Nuclear medicine or Radioiodine treatment	Male	April
Nuclear medicine or Radioiodine treatment	Female	April
Nuclear medicine or Radioiodine treatment	Female	April
Nuclear medicine or Radioiodine treatment	Female	August
Nuclear medicine or Radioiodine treatment	Male	August
Nuclear medicine or Radioiodine treatment	Female	August
Nuclear medicine or Radioiodine treatment	Male	August
Nuclear medicine or Radioiodine treatment	Female	August

Nuclear medicine or Radioiodine treatment	Male	August
Nuclear medicine or Radioiodine treatment	Male	August
Nuclear medicine or Radioiodine treatment	Female	August
Nuclear medicine or Radioiodine treatment	Male	December
Nuclear medicine or Radioiodine treatment	Female	December
Nuclear medicine or Radioiodine treatment	Male	December
Nuclear medicine or Radioiodine treatment	Female	December
Nuclear medicine or Radioiodine treatment	Female	December
Nuclear medicine or Radioiodine treatment	Male	December
Nuclear medicine or Radioiodine treatment	Female	December
Nuclear medicine or Radioiodine treatment	Male	December
Nuclear medicine or Radioiodine treatment	Male	February
Nuclear medicine or Radioiodine treatment	Female	February
Nuclear medicine or Radioiodine treatment	Male	February
Nuclear medicine or Radioiodine treatment	Female	February
Nuclear medicine or Radioiodine treatment	Male	February
Nuclear medicine or Radioiodine treatment	Male	February
Nuclear medicine or Radioiodine treatment	Female	February
Nuclear medicine or Radioiodine treatment	Female	February
Nuclear medicine or Radioiodine treatment	Female	January
Nuclear medicine or Radioiodine treatment	Male	January
Nuclear medicine or Radioiodine treatment	Female	January
Nuclear medicine or Radioiodine treatment	Male	January
Nuclear medicine or Radioiodine treatment	Female	January
Nuclear medicine or Radioiodine treatment	Male	January
Nuclear medicine or Radioiodine treatment	Female	January
Nuclear medicine or Radioiodine treatment	Male	January
Nuclear medicine or Radioiodine treatment	Male	July
Nuclear medicine or Radioiodine treatment	Female	July
Nuclear medicine or Radioiodine treatment	Female	July
Nuclear medicine or Radioiodine treatment	Male	July
Nuclear medicine or Radioiodine treatment	Male	June
Nuclear medicine or Radioiodine treatment	Female	June
Nuclear medicine or Radioiodine treatment	Male	June
Nuclear medicine or Radioiodine treatment	Female	June
Nuclear medicine or Radioiodine treatment	Female	March
Nuclear medicine or Radioiodine treatment	Male	March
Nuclear medicine or Radioiodine treatment	Male	March
Nuclear medicine or Radioiodine treatment	Female	March
Nuclear medicine or Radioiodine treatment	Female	March
Nuclear medicine or Radioiodine treatment	Male	March
Nuclear medicine or Radioiodine treatment	Female	May
Nuclear medicine or Radioiodine treatment	Male	May
Nuclear medicine or Radioiodine treatment	Female	May
Nuclear medicine or Radioiodine treatment	Male	May
Nuclear medicine or Radioiodine treatment	Female	May
Nuclear medicine or Radioiodine treatment	Male	May
Nuclear medicine or Radioiodine treatment	Female	May
Nuclear medicine or Radioiodine treatment	Male	May
Nuclear medicine or Radioiodine treatment	Female	November

Nuclear medicine or Radioiodine treatment	Male	November
Nuclear medicine or Radioiodine treatment	Male	November
Nuclear medicine or Radioiodine treatment	Female	November
Nuclear medicine or Radioiodine treatment	Male	November
Nuclear medicine or Radioiodine treatment	Female	September
Nuclear medicine or Radioiodine treatment	Male	September
Nuclear medicine or Radioiodine treatment	Male	September
Nuclear medicine or Radioiodine treatment	Female	September
Nuclear medicine or Radioiodine treatment	Male	September
Nuclear medicine or Radioiodine treatment	Female	September
Nuclear medicine or Radioiodine treatment	Male	September
Nuclear medicine or Radioiodine treatment	Female	September
Radiotherapy	Female	April
Radiotherapy	Female	August
Radiotherapy	Male	August
Radiotherapy	Female	August
Radiotherapy	Male	December
Radiotherapy	Female	February
Radiotherapy	Female	January
Radiotherapy	Female	July
Radiotherapy	Female	July
Radiotherapy	Male	July
Radiotherapy	Female	June
Radiotherapy	Female	March
Radiotherapy	Female	May
Radiotherapy	Female	November
Radiotherapy	Female	October
Radiotherapy	Female	September
Nuclear medicine or Radioiodine treatment	Female	April
Nuclear medicine or Radioiodine treatment	Male	April
Nuclear medicine or Radioiodine treatment	Female	April
Nuclear medicine or Radioiodine treatment	Male	April
Nuclear medicine or Radioiodine treatment	Female	April
Nuclear medicine or Radioiodine treatment	Male	April
Nuclear medicine or Radioiodine treatment	Female	April
Nuclear medicine or Radioiodine treatment	Male	April
Nuclear medicine or Radioiodine treatment	Female	August
Nuclear medicine or Radioiodine treatment	Male	August
Nuclear medicine or Radioiodine treatment	Male	August
Nuclear medicine or Radioiodine treatment	Female	August
Nuclear medicine or Radioiodine treatment	Female	August
Nuclear medicine or Radioiodine treatment	Male	August
Nuclear medicine or Radioiodine treatment	Female	August
Nuclear medicine or Radioiodine treatment	Female	August
Nuclear medicine or Radioiodine treatment	Male	August
Nuclear medicine or Radioiodine treatment	Male	August
Nuclear medicine or Radioiodine treatment	Male	December
Nuclear medicine or Radioiodine treatment	Female	December
Nuclear medicine or Radioiodine treatment	Male	December
Nuclear medicine or Radioiodine treatment	Female	December

Nuclear medicine or Radioiodine treatment	Male	December
Nuclear medicine or Radioiodine treatment	Female	December
Nuclear medicine or Radioiodine treatment	Male	December
Nuclear medicine or Radioiodine treatment	Female	December
Nuclear medicine or Radioiodine treatment	Male	December
Nuclear medicine or Radioiodine treatment	Female	December
Nuclear medicine or Radioiodine treatment	Female	February
Nuclear medicine or Radioiodine treatment	Male	February
Nuclear medicine or Radioiodine treatment	Female	February
Nuclear medicine or Radioiodine treatment	Male	February
Nuclear medicine or Radioiodine treatment	Male	February
Nuclear medicine or Radioiodine treatment	Male	February
Nuclear medicine or Radioiodine treatment	Female	February
Nuclear medicine or Radioiodine treatment	Male	February
Nuclear medicine or Radioiodine treatment	Female	February
Nuclear medicine or Radioiodine treatment	Female	February
Nuclear medicine or Radioiodine treatment	Female	January
Nuclear medicine or Radioiodine treatment	Male	January
Nuclear medicine or Radioiodine treatment	Male	January
Nuclear medicine or Radioiodine treatment	Female	January
Nuclear medicine or Radioiodine treatment	Male	January
Nuclear medicine or Radioiodine treatment	Male	January
Nuclear medicine or Radioiodine treatment	Female	January
Nuclear medicine or Radioiodine treatment	Female	January
Nuclear medicine or Radioiodine treatment	Female	January
Nuclear medicine or Radioiodine treatment	Male	January
Nuclear medicine or Radioiodine treatment	Male	July
Nuclear medicine or Radioiodine treatment	Male	July
Nuclear medicine or Radioiodine treatment	Female	July
Nuclear medicine or Radioiodine treatment	Male	July
Nuclear medicine or Radioiodine treatment	Female	July
Nuclear medicine or Radioiodine treatment	Female	July
Nuclear medicine or Radioiodine treatment	Female	July
Nuclear medicine or Radioiodine treatment	Female	July
Nuclear medicine or Radioiodine treatment	Male	July
Nuclear medicine or Radioiodine treatment	Male	July
Nuclear medicine or Radioiodine treatment	Female	June
Nuclear medicine or Radioiodine treatment	Male	June
Nuclear medicine or Radioiodine treatment	Male	June
Nuclear medicine or Radioiodine treatment	Male	June
Nuclear medicine or Radioiodine treatment	Male	March
Nuclear medicine or Radioiodine treatment	Male	March
Nuclear medicine or Radioiodine treatment	Female	May
Nuclear medicine or Radioiodine treatment	Male	May
Nuclear medicine or Radioiodine treatment	Female	May
Nuclear medicine or Radioiodine treatment	Female	May
Nuclear medicine or Radioiodine treatment	Male	May
Nuclear medicine or Radioiodine treatment	Female	May
Nuclear medicine or Radioiodine treatment	Male	May
Nuclear medicine or Radioiodine treatment	Male	May

Nuclear medicine or Radioiodine treatment	Male	May
Nuclear medicine or Radioiodine treatment	Female	May
Nuclear medicine or Radioiodine treatment	Male	November
Nuclear medicine or Radioiodine treatment	Female	November
Nuclear medicine or Radioiodine treatment	Male	November
Nuclear medicine or Radioiodine treatment	Female	November
Nuclear medicine or Radioiodine treatment	Female	November
Nuclear medicine or Radioiodine treatment	Male	November
Nuclear medicine or Radioiodine treatment	Female	November
Nuclear medicine or Radioiodine treatment	Male	November
Nuclear medicine or Radioiodine treatment	Female	November
Nuclear medicine or Radioiodine treatment	Male	November
Nuclear medicine or Radioiodine treatment	Male	October
Nuclear medicine or Radioiodine treatment	Female	September
Nuclear medicine or Radioiodine treatment	Male	September
Nuclear medicine or Radioiodine treatment	Male	September
Nuclear medicine or Radioiodine treatment	Female	September
Nuclear medicine or Radioiodine treatment	Male	September
Nuclear medicine or Radioiodine treatment	Female	September
Nuclear medicine or Radioiodine treatment	Male	September
Nuclear medicine or Radioiodine treatment	Female	September
Nuclear medicine or Radioiodine treatment	Male	September
Nuclear medicine or Radioiodine treatment	Female	September
Nuclear medicine or Radioiodine treatment	Male	April
Nuclear medicine or Radioiodine treatment	Female	April
Nuclear medicine or Radioiodine treatment	Male	April
Nuclear medicine or Radioiodine treatment	Female	April
Nuclear medicine or Radioiodine treatment	Female	April
Nuclear medicine or Radioiodine treatment	Male	April
Nuclear medicine or Radioiodine treatment	Female	April
Nuclear medicine or Radioiodine treatment	Male	April
Nuclear medicine or Radioiodine treatment	Male	April
Nuclear medicine or Radioiodine treatment	Female	April
Nuclear medicine or Radioiodine treatment	Male	April
Nuclear medicine or Radioiodine treatment	Female	April
Nuclear medicine or Radioiodine treatment	Male	August
Nuclear medicine or Radioiodine treatment	Male	August
Nuclear medicine or Radioiodine treatment	Female	August
Nuclear medicine or Radioiodine treatment	Male	August
Nuclear medicine or Radioiodine treatment	Female	August
Nuclear medicine or Radioiodine treatment	Male	August
Nuclear medicine or Radioiodine treatment	Female	August
Nuclear medicine or Radioiodine treatment	Male	August
Nuclear medicine or Radioiodine treatment	Female	August
Nuclear medicine or Radioiodine treatment	Female	August
Nuclear medicine or Radioiodine treatment	Male	August
Nuclear medicine or Radioiodine treatment	Female	August
Nuclear medicine or Radioiodine treatment	Female	December
Nuclear medicine or Radioiodine treatment	Female	December
Nuclear medicine or Radioiodine treatment	Male	December

Nuclear medicine or Radioiodine treatment	Female	December
Nuclear medicine or Radioiodine treatment	Female	December
Nuclear medicine or Radioiodine treatment	Male	December
Nuclear medicine or Radioiodine treatment	Male	December
Nuclear medicine or Radioiodine treatment	Male	December
Nuclear medicine or Radioiodine treatment	Female	December
Nuclear medicine or Radioiodine treatment	Female	December
Nuclear medicine or Radioiodine treatment	Male	December
Nuclear medicine or Radioiodine treatment	Male	December
Nuclear medicine or Radioiodine treatment	Female	February
Nuclear medicine or Radioiodine treatment	Male	February
Nuclear medicine or Radioiodine treatment	Female	February
Nuclear medicine or Radioiodine treatment	Male	February
Nuclear medicine or Radioiodine treatment	Female	February
Nuclear medicine or Radioiodine treatment	Female	February
Nuclear medicine or Radioiodine treatment	Male	February
Nuclear medicine or Radioiodine treatment	Male	February
Nuclear medicine or Radioiodine treatment	Female	February
Nuclear medicine or Radioiodine treatment	Male	February
Nuclear medicine or Radioiodine treatment	Female	February
Nuclear medicine or Radioiodine treatment	Male	February
Nuclear medicine or Radioiodine treatment	Male	January
Nuclear medicine or Radioiodine treatment	Male	January
Nuclear medicine or Radioiodine treatment	Female	January
Nuclear medicine or Radioiodine treatment	Male	January
Nuclear medicine or Radioiodine treatment	Female	January
Nuclear medicine or Radioiodine treatment	Female	January
Nuclear medicine or Radioiodine treatment	Female	January
Nuclear medicine or Radioiodine treatment	Male	January
Nuclear medicine or Radioiodine treatment	Male	January
Nuclear medicine or Radioiodine treatment	Female	January
Nuclear medicine or Radioiodine treatment	Male	January
Nuclear medicine or Radioiodine treatment	Female	January
Nuclear medicine or Radioiodine treatment	Female	July
Nuclear medicine or Radioiodine treatment	Male	June
Nuclear medicine or Radioiodine treatment	Female	June
Nuclear medicine or Radioiodine treatment	Male	June
Nuclear medicine or Radioiodine treatment	Female	June
Nuclear medicine or Radioiodine treatment	Male	June
Nuclear medicine or Radioiodine treatment	Female	June
Nuclear medicine or Radioiodine treatment	Female	June
Nuclear medicine or Radioiodine treatment	Male	June
Nuclear medicine or Radioiodine treatment	Female	June
Nuclear medicine or Radioiodine treatment	Male	June
Nuclear medicine or Radioiodine treatment	Female	June
Nuclear medicine or Radioiodine treatment	Male	June
Nuclear medicine or Radioiodine treatment	Female	March
Nuclear medicine or Radioiodine treatment	Male	March
Nuclear medicine or Radioiodine treatment	Male	March
Nuclear medicine or Radioiodine treatment	Female	March

Nuclear medicine or Radioiodine treatment	Male	March
Nuclear medicine or Radioiodine treatment	Female	March
Nuclear medicine or Radioiodine treatment	Female	March
Nuclear medicine or Radioiodine treatment	Male	March
Nuclear medicine or Radioiodine treatment	Female	March
Nuclear medicine or Radioiodine treatment	Male	March
Nuclear medicine or Radioiodine treatment	Female	March
Nuclear medicine or Radioiodine treatment	Male	March
Nuclear medicine or Radioiodine treatment	Male	May
Nuclear medicine or Radioiodine treatment	Female	May
Nuclear medicine or Radioiodine treatment	Female	May
Nuclear medicine or Radioiodine treatment	Male	May
Nuclear medicine or Radioiodine treatment	Male	May
Nuclear medicine or Radioiodine treatment	Female	May
Nuclear medicine or Radioiodine treatment	Male	May
Nuclear medicine or Radioiodine treatment	Female	May
Nuclear medicine or Radioiodine treatment	Male	May
Nuclear medicine or Radioiodine treatment	Female	May
Nuclear medicine or Radioiodine treatment	Male	May
Nuclear medicine or Radioiodine treatment	Female	May
Nuclear medicine or Radioiodine treatment	Male	November
Nuclear medicine or Radioiodine treatment	Female	November
Nuclear medicine or Radioiodine treatment	Male	November
Nuclear medicine or Radioiodine treatment	Male	October
Nuclear medicine or Radioiodine treatment	Male	October
Nuclear medicine or Radioiodine treatment	Female	October
Nuclear medicine or Radioiodine treatment	Male	October
Nuclear medicine or Radioiodine treatment	Female	October
Nuclear medicine or Radioiodine treatment	Male	October
Nuclear medicine or Radioiodine treatment	Female	September
Nuclear medicine or Radioiodine treatment	Male	September
Nuclear medicine or Radioiodine treatment	Female	September
Nuclear medicine or Radioiodine treatment	Male	September
Nuclear medicine or Radioiodine treatment	Male	September
Nuclear medicine or Radioiodine treatment	Female	September
Nuclear medicine or Radioiodine treatment	Female	September
Nuclear medicine or Radioiodine treatment	Female	September
Nuclear medicine or Radioiodine treatment	Male	September
Nuclear medicine or Radioiodine treatment	Male	September
Nuclear medicine or Radioiodine treatment	Male	September
Nuclear medicine or Radioiodine treatment	Female	September
Surgery	Male	April
Surgery	Male	April
Surgery	Female	April
Surgery	Female	April
Surgery	Male	April
Surgery	Female	April
Surgery	Female	August
Surgery	Male	August
Surgery	Male	August

Surgery	Female	August
Surgery	Male	August
Surgery	Male	August
Surgery	Female	August
Surgery	Female	August
Surgery	Male	August
Surgery	Female	August
Surgery	Male	December
Surgery	Female	December
Surgery	Male	December
Surgery	Female	December
Surgery	Female	December
Surgery	Female	December
Surgery	Male	December
Surgery	Female	February
Surgery	Male	February
Surgery	Male	February
Surgery	Male	February
Surgery	Male	February
Surgery	Female	February
Surgery	Female	January
Surgery	Female	January
Surgery	Female	January
Surgery	Male	January
Surgery	Female	January
Surgery	Male	January
Surgery	Female	July
Surgery	Female	July
Surgery	Male	July
Surgery	Female	July
Surgery	Female	July
Surgery	Female	July
Surgery	Male	July
Surgery	Male	July
Surgery	Female	July
Surgery	Female	July
Surgery	Male	July
Surgery	Female	July
Surgery	Female	June
Surgery	Female	June
Surgery	Male	June
Surgery	Female	June
Surgery	Female	June
Surgery	Female	June
Surgery	Male	June
Surgery	Male	June
Surgery	Male	June
Surgery	Male	March
Surgery	Male	March
Surgery	Male	March

Surgery	Female	March
Surgery	Female	March
Surgery	Female	May
Surgery	Male	May
Surgery	Male	May
Surgery	Female	May
Surgery	Male	May
Surgery	Male	May
Surgery	Male	November
Surgery	Male	November
Surgery	Male	November
Surgery	Female	November
Surgery	Female	November
Surgery	Female	October
Surgery	Male	October
Surgery	Female	October
Surgery	Male	October
Surgery	Male	October
Surgery	Male	October
Surgery	Male	October
Surgery	Female	October
Surgery	Male	October
Surgery	Female	October
Surgery	Female	October
Surgery	Female	September
Surgery	Female	September
Surgery	Male	September
Surgery	Female	September
Surgery	Male	September
Surgery	Male	September
Surgery	Female	September
Surgery	Female	September
Surgery	Female	September
Surgery	Male	September
Nuclear medicine or Radioiodine treatment	Female	April
Nuclear medicine or Radioiodine treatment	Male	April
Radiotherapy	Male	August
Radiotherapy	Female	August
Radiotherapy	Female	August
Radiotherapy	Female	August
Radiotherapy	Male	December
Radiotherapy	Male	December
Radiotherapy	Female	December
Radiotherapy	Female	December
Radiotherapy	Female	December
Radiotherapy	Female	December
Radiotherapy	Male	December
Radiotherapy	Female	December
Radiotherapy	Male	January
Nuclear medicine or Radioiodine treatment	Male	July

Radiotherapy	Female	July
Nuclear medicine or Radioiodine treatment	Male	July
Radiotherapy	Male	July
Radiotherapy	Female	July
Radiotherapy	Male	July
Nuclear medicine or Radioiodine treatment	Male	June
Nuclear medicine or Radioiodine treatment	Male	June
Nuclear medicine or Radioiodine treatment	Female	June
Radiotherapy	Male	June
Radiotherapy	Male	June
Radiotherapy	Female	June
Nuclear medicine or Radioiodine treatment	Female	March
Nuclear medicine or Radioiodine treatment	Male	March
Nuclear medicine or Radioiodine treatment	Female	March
Nuclear medicine or Radioiodine treatment	Female	March
Nuclear medicine or Radioiodine treatment	Male	March
Nuclear medicine or Radioiodine treatment	Female	March
Nuclear medicine or Radioiodine treatment	Male	March
Radiotherapy	Female	May
Radiotherapy	Female	May
Radiotherapy	Male	November
Radiotherapy	Female	November
Radiotherapy	Male	November
Radiotherapy	Male	November
Nuclear medicine or Radioiodine treatment	Female	November
Nuclear medicine or Radioiodine treatment	Female	November
Radiotherapy	Female	November
Nuclear medicine or Radioiodine treatment	Male	November
Radiotherapy	Female	November
Radiotherapy	Female	November
Radiotherapy	Male	November
Radiotherapy	Female	October
Nuclear medicine or Radioiodine treatment	Female	October
Radiotherapy	Male	October
Radiotherapy	Female	October
Radiotherapy	Male	October
Nuclear medicine or Radioiodine treatment	Female	October
Nuclear medicine or Radioiodine treatment	Female	October
Radiotherapy	Male	October
Radiotherapy	Female	October
Radiotherapy	Male	September
Radiotherapy	Male	September
Radiotherapy	Female	September
Radiotherapy	Male	September
Hormonal or Targeted therapies	Female	April
Hormonal or Targeted therapies	Male	April
Hormonal or Targeted therapies	Male	April
Hormonal or Targeted therapies	Male	April
Hormonal or Targeted therapies	Female	April
Hormonal or Targeted therapies	Male	April

Hormonal or Targeted therapies	Male	April
Hormonal or Targeted therapies	Female	April
Hormonal or Targeted therapies	Male	April
Hormonal or Targeted therapies	Male	August
Hormonal or Targeted therapies	Female	August
Hormonal or Targeted therapies	Male	August
Hormonal or Targeted therapies	Female	August
Hormonal or Targeted therapies	Female	August
Hormonal or Targeted therapies	Male	August
Hormonal or Targeted therapies	Female	August
Hormonal or Targeted therapies	Male	August
Hormonal or Targeted therapies	Female	August
Hormonal or Targeted therapies	Male	August
Hormonal or Targeted therapies	Female	August
Hormonal or Targeted therapies	Female	August
Hormonal or Targeted therapies	Female	August
Hormonal or Targeted therapies	Male	August
Hormonal or Targeted therapies	Female	December
Hormonal or Targeted therapies	Male	December
Hormonal or Targeted therapies	Female	December
Hormonal or Targeted therapies	Female	December
Hormonal or Targeted therapies	Male	December
Hormonal or Targeted therapies	Female	December
Hormonal or Targeted therapies	Male	December
Hormonal or Targeted therapies	Male	December
Hormonal or Targeted therapies	Male	February
Hormonal or Targeted therapies	Female	February
Hormonal or Targeted therapies	Female	February
Hormonal or Targeted therapies	Male	February
Hormonal or Targeted therapies	Male	February
Hormonal or Targeted therapies	Male	January
Hormonal or Targeted therapies	Female	January
Hormonal or Targeted therapies	Female	January
Hormonal or Targeted therapies	Male	January
Hormonal or Targeted therapies	Female	January
Hormonal or Targeted therapies	Female	January
Hormonal or Targeted therapies	Male	January
Hormonal or Targeted therapies	Male	January
Hormonal or Targeted therapies	Female	January
Hormonal or Targeted therapies	Female	January
Hormonal or Targeted therapies	Female	January
Hormonal or Targeted therapies	Male	January
Hormonal or Targeted therapies	Female	July
Hormonal or Targeted therapies	Female	July
Hormonal or Targeted therapies	Female	July
Hormonal or Targeted therapies	Female	July
Hormonal or Targeted therapies	Female	July
Hormonal or Targeted therapies	Female	July
Hormonal or Targeted therapies	Female	July
Hormonal or Targeted therapies	Female	July

Hormonal or Targeted therapies	Female	July
Hormonal or Targeted therapies	Male	July
Hormonal or Targeted therapies	Female	June
Hormonal or Targeted therapies	Female	June
Hormonal or Targeted therapies	Female	June
Hormonal or Targeted therapies	Female	June
Hormonal or Targeted therapies	Male	June
Hormonal or Targeted therapies	Male	June
Hormonal or Targeted therapies	Male	June
Hormonal or Targeted therapies	Male	June
Hormonal or Targeted therapies	Male	June
Hormonal or Targeted therapies	Female	June
Hormonal or Targeted therapies	Male	June
Hormonal or Targeted therapies	Female	June
Hormonal or Targeted therapies	Male	March
Hormonal or Targeted therapies	Female	March
Hormonal or Targeted therapies	Male	March
Hormonal or Targeted therapies	Female	March
Hormonal or Targeted therapies	Male	March
Hormonal or Targeted therapies	Female	March
Hormonal or Targeted therapies	Female	March
Hormonal or Targeted therapies	Female	March
Hormonal or Targeted therapies	Female	March
Hormonal or Targeted therapies	Male	March
Hormonal or Targeted therapies	Male	March
Hormonal or Targeted therapies	Female	March
Hormonal or Targeted therapies	Female	March
Hormonal or Targeted therapies	Female	March
Hormonal or Targeted therapies	Female	March
Hormonal or Targeted therapies	Male	March
Hormonal or Targeted therapies	Female	March
Hormonal or Targeted therapies	Male	March
Hormonal or Targeted therapies	Male	March
Hormonal or Targeted therapies	Female	March
Hormonal or Targeted therapies	Male	May
Hormonal or Targeted therapies	Female	May
Hormonal or Targeted therapies	Female	May
Hormonal or Targeted therapies	Male	May
Hormonal or Targeted therapies	Male	May
Hormonal or Targeted therapies	Female	May
Hormonal or Targeted therapies	Male	May
Hormonal or Targeted therapies	Female	May
Hormonal or Targeted therapies	Male	May
Hormonal or Targeted therapies	Female	May
Hormonal or Targeted therapies	Male	May
Hormonal or Targeted therapies	Male	May
Hormonal or Targeted therapies	Female	May
Hormonal or Targeted therapies	Male	May
Hormonal or Targeted therapies	Female	May
Hormonal or Targeted therapies	Female	May

Hormonal or Targeted therapies	Female	May
Hormonal or Targeted therapies	Male	May
Hormonal or Targeted therapies	Female	November
Hormonal or Targeted therapies	Female	November
Hormonal or Targeted therapies	Female	November
Hormonal or Targeted therapies	Male	November
Hormonal or Targeted therapies	Male	November
Hormonal or Targeted therapies	Female	November
Hormonal or Targeted therapies	Male	November
Hormonal or Targeted therapies	Female	October
Hormonal or Targeted therapies	Female	October
Hormonal or Targeted therapies	Female	October
Hormonal or Targeted therapies	Male	October
Hormonal or Targeted therapies	Male	October
Hormonal or Targeted therapies	Female	October
Hormonal or Targeted therapies	Male	October
Hormonal or Targeted therapies	Female	October
Hormonal or Targeted therapies	Female	October
Hormonal or Targeted therapies	Female	October
Hormonal or Targeted therapies	Female	October
Hormonal or Targeted therapies	Male	September
Hormonal or Targeted therapies	Female	September
Hormonal or Targeted therapies	Male	September
Hormonal or Targeted therapies	Female	September
Hormonal or Targeted therapies	Male	September
Hormonal or Targeted therapies	Female	September
Hormonal or Targeted therapies	Male	September
Hormonal or Targeted therapies	Male	September
Hormonal or Targeted therapies	Female	September
Hormonal or Targeted therapies	Female	September
Hormonal or Targeted therapies	Male	September
Hormonal or Targeted therapies	Male	September
Hormonal or Targeted therapies	Male	September
Hormonal or Targeted therapies	Male	September
Hormonal or Targeted therapies	Female	September
Hormonal or Targeted therapies	Female	September
Hormonal or Targeted therapies	Male	September
Surgery	Female	April
Surgery	Female	April
Nuclear medicine or Radioiodine treatment	Male	April
Nuclear medicine or Radioiodine treatment	Male	April
Nuclear medicine or Radioiodine treatment	Female	April
Surgery	Female	December
Surgery	Female	December
Surgery	Female	February
Radiotherapy	Male	February
Nuclear medicine or Radioiodine treatment	Female	July
Nuclear medicine or Radioiodine treatment	Male	July
Nuclear medicine or Radioiodine treatment	Female	July
Nuclear medicine or Radioiodine treatment	Male	July

Nuclear medicine or Radioiodine treatment	Female	July
Nuclear medicine or Radioiodine treatment	Male	July
Nuclear medicine or Radioiodine treatment	Male	July
Nuclear medicine or Radioiodine treatment	Female	July
Nuclear medicine or Radioiodine treatment	Male	July
Nuclear medicine or Radioiodine treatment	Female	July
Nuclear medicine or Radioiodine treatment	Female	July
Nuclear medicine or Radioiodine treatment	Male	July
Nuclear medicine or Radioiodine treatment	Female	July
Surgery	Male	July
Surgery	Female	July
Nuclear medicine or Radioiodine treatment	Female	June
Nuclear medicine or Radioiodine treatment	Male	June
Nuclear medicine or Radioiodine treatment	Female	June
Nuclear medicine or Radioiodine treatment	Female	June
Nuclear medicine or Radioiodine treatment	Female	June
Nuclear medicine or Radioiodine treatment	Male	June
Nuclear medicine or Radioiodine treatment	Female	June
Surgery	Female	June
Surgery	Male	March
Surgery	Female	March
Surgery	Male	March
Surgery	Female	March
Nuclear medicine or Radioiodine treatment	Female	March
Nuclear medicine or Radioiodine treatment	Female	March
Nuclear medicine or Radioiodine treatment	Male	March
Surgery	Female	May
Surgery	Female	November
Surgery	Male	November
Surgery	Female	November
Nuclear medicine or Radioiodine treatment	Female	November
Nuclear medicine or Radioiodine treatment	Male	November
Nuclear medicine or Radioiodine treatment	Male	November
Radiotherapy	Male	November
Nuclear medicine or Radioiodine treatment	Female	November
Nuclear medicine or Radioiodine treatment	Female	November
Nuclear medicine or Radioiodine treatment	Male	November
Nuclear medicine or Radioiodine treatment	Female	November
Nuclear medicine or Radioiodine treatment	Female	November
Nuclear medicine or Radioiodine treatment	Male	November
Nuclear medicine or Radioiodine treatment	Female	October
Nuclear medicine or Radioiodine treatment	Female	October
Nuclear medicine or Radioiodine treatment	Male	October
Nuclear medicine or Radioiodine treatment	Female	October
Nuclear medicine or Radioiodine treatment	Male	October
Nuclear medicine or Radioiodine treatment	Female	October
Nuclear medicine or Radioiodine treatment	Male	October
Nuclear medicine or Radioiodine treatment	Male	October
Nuclear medicine or Radioiodine treatment	Female	October
Nuclear medicine or Radioiodine treatment	Male	October

Nuclear medicine or Radioiodine treatment	Female	October
Nuclear medicine or Radioiodine treatment	Male	October
Nuclear medicine or Radioiodine treatment	Female	October
Nuclear medicine or Radioiodine treatment	Male	October
Nuclear medicine or Radioiodine treatment	Female	October
Nuclear medicine or Radioiodine treatment	Male	October
Nuclear medicine or Radioiodine treatment	Female	October
Nuclear medicine or Radioiodine treatment	Male	October
Nuclear medicine or Radioiodine treatment	Female	October
Nuclear medicine or Radioiodine treatment	Male	October
Surgery	Male	September
Surgery	Female	September
Surgery	Male	September
Surgery	Male	September
Surgery	Male	April
Surgery	Female	April
Surgery	Female	April
Surgery	Female	April
Surgery	Male	April
Surgery	Male	April
Surgery	Male	April
Surgery	Female	April
Surgery	Female	August
Surgery	Female	August
Surgery	Male	August
Radiotherapy	Male	August
Surgery	Female	August
Surgery	Male	August
Surgery	Female	August
Surgery	Male	August
Radiotherapy	Female	August
Radiotherapy	Male	August
Radiotherapy	Male	August
Radiotherapy	Female	August
Radiotherapy	Male	August
Radiotherapy	Female	August
Radiotherapy	Female	August
Radiotherapy	Male	August
Radiotherapy	Female	August
Radiotherapy	Male	August
Surgery	Female	December
Surgery	Male	December
Surgery	Female	December
Surgery	Male	December
Radiotherapy	Male	December
Radiotherapy	Female	December
Radiotherapy	Male	December
Radiotherapy	Female	December
Radiotherapy	Male	December
Radiotherapy	Female	December

Radiotherapy	Male	December
Radiotherapy	Male	December
Radiotherapy	Female	December
Radiotherapy	Male	December
Radiotherapy	Female	December
Radiotherapy	Female	December
Surgery	Female	June
Surgery	Male	June
Surgery	Male	June
Surgery	Male	June
Surgery	Female	June
Surgery	Male	June
Surgery	Female	June
Surgery	Female	June
Surgery	Female	May
Surgery	Male	May
Surgery	Male	May
Surgery	Female	May
Surgery	Male	May
Surgery	Male	May
Surgery	Female	May
Surgery	Female	May
Surgery	Male	October
Surgery	Male	October
Surgery	Female	October
Surgery	Female	October
Surgery	Male	October
Surgery	Female	October
Surgery	Female	October
Surgery	Male	October
Radiotherapy	Male	September
Radiotherapy	Female	September
Radiotherapy	Male	September
Radiotherapy	Male	August
Radiotherapy	Female	August
Radiotherapy	Female	August
Radiotherapy	Male	August
Radiotherapy	Female	August
Radiotherapy	Male	August
Radiotherapy	Male	July
Radiotherapy	Female	July
Radiotherapy	Female	July
Radiotherapy	Male	July
Radiotherapy	Female	July
Surgery	Male	August
Surgery	Female	August
Surgery	Female	August
Surgery	Male	August
Surgery	Male	August
Surgery	Female	August

Surgery	Male	August
Surgery	Female	August
Surgery	Male	August
Surgery	Female	August
Surgery	Male	August
Surgery	Female	August
Surgery	Male	August
Surgery	Male	August
Surgery	Female	August
Surgery	Female	December
Surgery	Male	December
Surgery	Female	December
Surgery	Male	December
Surgery	Female	December
Surgery	Male	December
Surgery	Female	December
Surgery	Male	December
Surgery	Female	December
Surgery	Male	December
Surgery	Female	December
Surgery	Male	December
Surgery	Female	December
Surgery	Male	December
Surgery	Female	July
Surgery	Male	July
Surgery	Male	July
Surgery	Male	July
Surgery	Female	July
Surgery	Male	July
Surgery	Female	July
Surgery	Male	July
Surgery	Female	July
Surgery	Female	July
Surgery	Male	July
Surgery	Female	July
Surgery	Male	July
Surgery	Female	June
Surgery	Male	June
Surgery	Female	June
Surgery	Male	June
Surgery	Female	June
Surgery	Male	June
Surgery	Female	June
Surgery	Male	June
Surgery	Female	June
Surgery	Male	June
Surgery	Female	June
Surgery	Male	June
Surgery	Male	June
Surgery	Male	March

Surgery	Female	March
Surgery	Male	March
Surgery	Female	March
Surgery	Female	March
Surgery	Female	March
Surgery	Male	March
Surgery	Male	March
Surgery	Female	March
Surgery	Male	March
Surgery	Female	March
Surgery	Female	March
Surgery	Male	March
Surgery	Male	March
Surgery	Female	March
Surgery	Female	May
Surgery	Female	May
Surgery	Female	May
Surgery	Male	May
Surgery	Male	May
Surgery	Male	May
Surgery	Male	May
Surgery	Female	May
Surgery	Male	May
Surgery	Female	May
Surgery	Female	May
Surgery	Male	May
Surgery	Male	May
Surgery	Female	May
Surgery	Female	May
Surgery	Male	May
Surgery	Male	November
Surgery	Female	November
Surgery	Female	November
Surgery	Male	November
Surgery	Female	November
Surgery	Female	November
Surgery	Female	November
Surgery	Male	November
Surgery	Male	November
Surgery	Male	November
Surgery	Male	November
Surgery	Male	October
Surgery	Male	October
Surgery	Female	October
Surgery	Female	October
Surgery	Male	October
Surgery	Female	October
Surgery	Female	October
Surgery	Male	October
Surgery	Male	October

Surgery	Male	October
Surgery	Female	October
Surgery	Female	October
Surgery	Female	October
Surgery	Male	October
Surgery	Female	October
Surgery	Male	October
Surgery	Male	September
Surgery	Male	September
Surgery	Male	September
Surgery	Female	September
Surgery	Female	September
Surgery	Female	September
Surgery	Male	September
Surgery	Female	September
Surgery	Female	September
Surgery	Male	September
Surgery	Male	September
Surgery	Male	September
Surgery	Female	September
Surgery	Male	September
Surgery	Female	April
Surgery	Male	April
Surgery	Male	April
Surgery	Female	April
Surgery	Female	April
Surgery	Female	April
Surgery	Male	April
Surgery	Male	April
Surgery	Male	April
Surgery	Female	April
Surgery	Female	February
Surgery	Male	February
Surgery	Female	February
Surgery	Male	February
Surgery	Female	February
Surgery	Male	February
Surgery	Female	February
Surgery	Male	February
Surgery	Female	February
Surgery	Male	February
Surgery	Female	February
Surgery	Female	February
Surgery	Male	February
Surgery	Male	January
Surgery	Female	January
Surgery	Male	January
Surgery	Female	January
Surgery	Female	January
Surgery	Male	January

Surgery	Female	January
Surgery	Male	January
Surgery	Female	January
Surgery	Female	January
Surgery	Male	January
Surgery	Male	January
Surgery	Female	January
Surgery	Male	July
Surgery	Female	July
Surgery	Female	July
Surgery	Male	July
Surgery	Female	July
Surgery	Male	July
Surgery	Female	July
Surgery	Female	July
Surgery	Male	July
Surgery	Female	July
Surgery	Male	July
Surgery	Male	June
Surgery	Male	June
Surgery	Male	June
Surgery	Female	June
Surgery	Female	June
Surgery	Female	June
Surgery	Female	June
Surgery	Male	June
Surgery	Female	June
Surgery	Male	June
Surgery	Male	June
Surgery	Female	June
Surgery	Male	May
Surgery	Female	May
Surgery	Female	May
Surgery	Female	May
Surgery	Female	May
Surgery	Female	May
Surgery	Female	May
Surgery	Male	May
Surgery	Male	May
Surgery	Male	May
Surgery	Female	May
Surgery	Female	May
Surgery	Male	May
Surgery	Male	October
Surgery	Female	October
Surgery	Female	October
Surgery	Female	October
Surgery	Female	October
Surgery	Male	October
Surgery	Male	October

Surgery	Male	October
Surgery	Female	October
Surgery	Male	October
Radiotherapy	Female	April
Radiotherapy	Male	April
Radiotherapy	Female	April
Radiotherapy	Male	April
Radiotherapy	Female	April
Radiotherapy	Male	April
Radiotherapy	Female	April
Radiotherapy	Male	April
Radiotherapy	Male	April
Radiotherapy	Male	April
Radiotherapy	Female	April
Radiotherapy	Male	April
Radiotherapy	Male	April
Radiotherapy	Female	April
Radiotherapy	Female	April
Radiotherapy	Female	April
Radiotherapy	Female	December
Radiotherapy	Female	December
Radiotherapy	Male	December
Radiotherapy	Female	December
Radiotherapy	Male	December
Radiotherapy	Female	December
Radiotherapy	Male	December
Radiotherapy	Female	December
Radiotherapy	Male	December
Radiotherapy	Female	February
Radiotherapy	Male	February
Radiotherapy	Female	February
Radiotherapy	Male	February
Radiotherapy	Female	February
Radiotherapy	Male	February
Radiotherapy	Male	February
Radiotherapy	Female	February
Radiotherapy	Female	February
Radiotherapy	Male	February
Radiotherapy	Male	February
Radiotherapy	Male	February
Radiotherapy	Female	February
Radiotherapy	Female	February
Radiotherapy	Male	February
Radiotherapy	Female	February
Radiotherapy	Female	January
Radiotherapy	Female	January
Radiotherapy	Male	January
Radiotherapy	Male	January
Radiotherapy	Male	January
Radiotherapy	Female	January

Radiotherapy	Male	January
Radiotherapy	Female	January
Radiotherapy	Male	January
Radiotherapy	Male	January
Radiotherapy	Male	January
Radiotherapy	Female	January
Radiotherapy	Female	January
Radiotherapy	Female	January
Radiotherapy	Female	January
Radiotherapy	Female	June
Radiotherapy	Male	June
Radiotherapy	Male	June
Radiotherapy	Female	June
Radiotherapy	Female	June
Radiotherapy	Female	June
Radiotherapy	Male	June
Radiotherapy	Female	June
Radiotherapy	Female	June
Radiotherapy	Male	June
Radiotherapy	Female	June
Radiotherapy	Male	June
Radiotherapy	Male	June
Radiotherapy	Male	March
Radiotherapy	Female	March
Radiotherapy	Male	March
Radiotherapy	Female	March
Radiotherapy	Male	March
Radiotherapy	Female	March
Radiotherapy	Female	March
Radiotherapy	Male	March
Radiotherapy	Male	March
Radiotherapy	Female	March
Radiotherapy	Male	March
Radiotherapy	Female	March
Radiotherapy	Male	March
Radiotherapy	Female	March
Radiotherapy	Male	March
Radiotherapy	Female	March
Radiotherapy	Female	May
Radiotherapy	Male	May
Radiotherapy	Female	May
Radiotherapy	Male	May
Radiotherapy	Male	May
Radiotherapy	Female	May
Radiotherapy	Female	May
Radiotherapy	Male	May
Radiotherapy	Male	May
Radiotherapy	Female	May
Radiotherapy	Male	May
Radiotherapy	Female	May

Radiotherapy	Male	May
Radiotherapy	Male	May
Radiotherapy	Female	May
Radiotherapy	Male	November
Radiotherapy	Female	November
Radiotherapy	Male	November
Radiotherapy	Male	November
Radiotherapy	Female	November
Radiotherapy	Male	November
Radiotherapy	Female	November
Radiotherapy	Female	November
Radiotherapy	Male	November
Radiotherapy	Female	November
Radiotherapy	Female	November
Radiotherapy	Male	October
Radiotherapy	Male	October
Radiotherapy	Female	October
Radiotherapy	Female	October
Radiotherapy	Male	October
Radiotherapy	Male	October
Radiotherapy	Male	October
Radiotherapy	Female	October
Radiotherapy	Female	October
Radiotherapy	Female	October
Radiotherapy	Male	October
Radiotherapy	Female	September
Radiotherapy	Male	September
Radiotherapy	Female	September
Radiotherapy	Female	September
Radiotherapy	Male	September
Radiotherapy	Female	September
Radiotherapy	Male	September
Radiotherapy	Female	September
Radiotherapy	Female	September
Radiotherapy	Male	September
Radiotherapy	Female	September
Radiotherapy	Male	September
Hormonal or Targeted therapies	Female	April
Hormonal or Targeted therapies	Female	April
Hormonal or Targeted therapies	Male	April
Hormonal or Targeted therapies	Male	April
Hormonal or Targeted therapies	Female	April
Hormonal or Targeted therapies	Male	April
Hormonal or Targeted therapies	Male	April
Hormonal or Targeted therapies	Male	April
Hormonal or Targeted therapies	Female	April
Hormonal or Targeted therapies	Male	April
Hormonal or Targeted therapies	Female	April
Hormonal or Targeted therapies	Male	April
Hormonal or Targeted therapies	Male	August

Hormonal or Targeted therapies	Male	August
Hormonal or Targeted therapies	Female	August
Hormonal or Targeted therapies	Female	August
Hormonal or Targeted therapies	Male	August
Hormonal or Targeted therapies	Male	August
Hormonal or Targeted therapies	Male	August
Hormonal or Targeted therapies	Female	August
Hormonal or Targeted therapies	Female	August
Hormonal or Targeted therapies	Male	August
Hormonal or Targeted therapies	Female	August
Hormonal or Targeted therapies	Female	August
Hormonal or Targeted therapies	Male	August
Hormonal or Targeted therapies	Female	August
Hormonal or Targeted therapies	Male	August
Hormonal or Targeted therapies	Male	August
Hormonal or Targeted therapies	Female	August
Hormonal or Targeted therapies	Female	August
Hormonal or Targeted therapies	Male	August
Hormonal or Targeted therapies	Female	August
Hormonal or Targeted therapies	Male	August
Hormonal or Targeted therapies	Male	August
Hormonal or Targeted therapies	Male	August
Hormonal or Targeted therapies	Female	August
Hormonal or Targeted therapies	Male	August
Hormonal or Targeted therapies	Male	December
Hormonal or Targeted therapies	Male	December
Hormonal or Targeted therapies	Male	December
Hormonal or Targeted therapies	Female	December
Hormonal or Targeted therapies	Female	December
Hormonal or Targeted therapies	Male	December
Hormonal or Targeted therapies	Female	December
Hormonal or Targeted therapies	Male	December
Hormonal or Targeted therapies	Male	December
Hormonal or Targeted therapies	Female	December
Hormonal or Targeted therapies	Male	December
Hormonal or Targeted therapies	Female	December
Hormonal or Targeted therapies	Female	December
Hormonal or Targeted therapies	Male	December
Hormonal or Targeted therapies	Female	December
Hormonal or Targeted therapies	Male	December
Hormonal or Targeted therapies	Female	December
Hormonal or Targeted therapies	Male	December
Hormonal or Targeted therapies	Female	December
Hormonal or Targeted therapies	Male	December
Hormonal or Targeted therapies	Male	February
Hormonal or Targeted therapies	Male	February
Hormonal or Targeted therapies	Female	February
Hormonal or Targeted therapies	Male	February
Hormonal or Targeted therapies	Female	February
Hormonal or Targeted therapies	Male	February

Hormonal or Targeted therapies	Female	February
Hormonal or Targeted therapies	Female	February
Hormonal or Targeted therapies	Male	February
Hormonal or Targeted therapies	Male	February
Hormonal or Targeted therapies	Female	February
Hormonal or Targeted therapies	Male	January
Hormonal or Targeted therapies	Female	January
Hormonal or Targeted therapies	Female	January
Hormonal or Targeted therapies	Female	January
Hormonal or Targeted therapies	Male	January
Hormonal or Targeted therapies	Male	January
Hormonal or Targeted therapies	Female	January
Hormonal or Targeted therapies	Male	January
Hormonal or Targeted therapies	Female	January
Hormonal or Targeted therapies	Female	January
Hormonal or Targeted therapies	Male	January
Hormonal or Targeted therapies	Male	January
Hormonal or Targeted therapies	Male	July
Hormonal or Targeted therapies	Male	July
Hormonal or Targeted therapies	Male	July
Hormonal or Targeted therapies	Male	July
Hormonal or Targeted therapies	Male	July
Hormonal or Targeted therapies	Female	July
Hormonal or Targeted therapies	Male	July
Hormonal or Targeted therapies	Male	July
Hormonal or Targeted therapies	Female	July
Hormonal or Targeted therapies	Male	July
Hormonal or Targeted therapies	Female	July
Hormonal or Targeted therapies	Female	July
Hormonal or Targeted therapies	Female	July
Hormonal or Targeted therapies	Female	July
Hormonal or Targeted therapies	Male	July
Hormonal or Targeted therapies	Male	July
Hormonal or Targeted therapies	Female	July
Hormonal or Targeted therapies	Male	July
Hormonal or Targeted therapies	Male	July
Hormonal or Targeted therapies	Male	July
Hormonal or Targeted therapies	Male	July
Hormonal or Targeted therapies	Male	July
Hormonal or Targeted therapies	Female	July
Hormonal or Targeted therapies	Male	June
Hormonal or Targeted therapies	Female	June
Hormonal or Targeted therapies	Male	June
Hormonal or Targeted therapies	Female	June
Hormonal or Targeted therapies	Male	June
Hormonal or Targeted therapies	Female	June
Hormonal or Targeted therapies	Male	June
Hormonal or Targeted therapies	Male	June
Hormonal or Targeted therapies	Female	June
Hormonal or Targeted therapies	Male	June

Hormonal or Targeted therapies	Female	June
Hormonal or Targeted therapies	Female	June
Hormonal or Targeted therapies	Male	June
Hormonal or Targeted therapies	Male	June
Hormonal or Targeted therapies	Male	June
Hormonal or Targeted therapies	Male	June
Hormonal or Targeted therapies	Male	June
Hormonal or Targeted therapies	Male	March
Hormonal or Targeted therapies	Female	March
Hormonal or Targeted therapies	Female	March
Hormonal or Targeted therapies	Male	March
Hormonal or Targeted therapies	Female	March
Hormonal or Targeted therapies	Female	March
Hormonal or Targeted therapies	Male	March
Hormonal or Targeted therapies	Male	March
Hormonal or Targeted therapies	Male	March
Hormonal or Targeted therapies	Male	March
Hormonal or Targeted therapies	Male	March
Hormonal or Targeted therapies	Female	March
Hormonal or Targeted therapies	Female	March
Hormonal or Targeted therapies	Male	March
Hormonal or Targeted therapies	Male	March
Hormonal or Targeted therapies	Male	March
Hormonal or Targeted therapies	Female	March
Hormonal or Targeted therapies	Male	May
Hormonal or Targeted therapies	Male	May
Hormonal or Targeted therapies	Female	May
Hormonal or Targeted therapies	Female	May
Hormonal or Targeted therapies	Female	May
Hormonal or Targeted therapies	Male	May
Hormonal or Targeted therapies	Male	May
Hormonal or Targeted therapies	Male	May
Hormonal or Targeted therapies	Male	May
Hormonal or Targeted therapies	Female	May
Hormonal or Targeted therapies	Male	May
Hormonal or Targeted therapies	Female	May
Hormonal or Targeted therapies	Female	May
Hormonal or Targeted therapies	Female	May
Hormonal or Targeted therapies	Male	May
Hormonal or Targeted therapies	Male	May
Hormonal or Targeted therapies	Male	May
Hormonal or Targeted therapies	Male	May
Hormonal or Targeted therapies	Female	May
Hormonal or Targeted therapies	Male	May
Hormonal or Targeted therapies	Female	November
Hormonal or Targeted therapies	Male	November
Hormonal or Targeted therapies	Male	November
Hormonal or Targeted therapies	Male	November
Hormonal or Targeted therapies	Female	November
Hormonal or Targeted therapies	Male	November

Hormonal or Targeted therapies	Male	November
Hormonal or Targeted therapies	Female	November
Hormonal or Targeted therapies	Male	November
Hormonal or Targeted therapies	Female	November
Hormonal or Targeted therapies	Female	November
Hormonal or Targeted therapies	Male	November
Hormonal or Targeted therapies	Female	November
Hormonal or Targeted therapies	Male	November
Hormonal or Targeted therapies	Male	November
Hormonal or Targeted therapies	Male	November
Hormonal or Targeted therapies	Female	November
Hormonal or Targeted therapies	Male	November
Hormonal or Targeted therapies	Male	November
Hormonal or Targeted therapies	Male	November
Hormonal or Targeted therapies	Female	October
Hormonal or Targeted therapies	Male	October
Hormonal or Targeted therapies	Female	October
Hormonal or Targeted therapies	Male	October
Hormonal or Targeted therapies	Male	October
Hormonal or Targeted therapies	Female	October
Hormonal or Targeted therapies	Male	October
Hormonal or Targeted therapies	Male	October
Hormonal or Targeted therapies	Male	October
Hormonal or Targeted therapies	Female	October
Hormonal or Targeted therapies	Female	October
Hormonal or Targeted therapies	Female	October
Hormonal or Targeted therapies	Male	October
Hormonal or Targeted therapies	Male	October
Hormonal or Targeted therapies	Male	October
Hormonal or Targeted therapies	Male	October
Hormonal or Targeted therapies	Female	October
Hormonal or Targeted therapies	Male	October
Hormonal or Targeted therapies	Male	October
Hormonal or Targeted therapies	Male	October
Hormonal or Targeted therapies	Male	October
Hormonal or Targeted therapies	Male	October
Hormonal or Targeted therapies	Female	October
Hormonal or Targeted therapies	Female	October
Hormonal or Targeted therapies	Female	October
Hormonal or Targeted therapies	Female	October
Hormonal or Targeted therapies	Male	October
Hormonal or Targeted therapies	Female	October
Hormonal or Targeted therapies	Female	September
Hormonal or Targeted therapies	Male	September
Hormonal or Targeted therapies	Male	September
Hormonal or Targeted therapies	Male	September
Hormonal or Targeted therapies	Male	September
Hormonal or Targeted therapies	Male	September
Hormonal or Targeted therapies	Female	September
Hormonal or Targeted therapies	Male	September

Hormonal or Targeted therapies	Female	September
Hormonal or Targeted therapies	Female	September
Hormonal or Targeted therapies	Female	September
Hormonal or Targeted therapies	Male	September
Hormonal or Targeted therapies	Male	September
Hormonal or Targeted therapies	Female	September
Hormonal or Targeted therapies	Female	September
Hormonal or Targeted therapies	Male	September
Hormonal or Targeted therapies	Male	September
Hormonal or Targeted therapies	Female	April
Hormonal or Targeted therapies	Female	April
Hormonal or Targeted therapies	Male	April
Hormonal or Targeted therapies	Male	April
Hormonal or Targeted therapies	Male	August
Hormonal or Targeted therapies	Male	August
Hormonal or Targeted therapies	Female	August
Hormonal or Targeted therapies	Female	August
Hormonal or Targeted therapies	Male	August
Hormonal or Targeted therapies	Female	August
Hormonal or Targeted therapies	Male	August
Hormonal or Targeted therapies	Male	December
Hormonal or Targeted therapies	Female	December
Hormonal or Targeted therapies	Female	December
Hormonal or Targeted therapies	Male	December
Hormonal or Targeted therapies	Male	December
Hormonal or Targeted therapies	Male	December
Hormonal or Targeted therapies	Male	December
Hormonal or Targeted therapies	Female	February
Hormonal or Targeted therapies	Female	February
Hormonal or Targeted therapies	Female	February
Hormonal or Targeted therapies	Female	January
Hormonal or Targeted therapies	Male	January
Hormonal or Targeted therapies	Female	July
Hormonal or Targeted therapies	Female	July
Hormonal or Targeted therapies	Female	July
Hormonal or Targeted therapies	Male	July
Hormonal or Targeted therapies	Male	July
Hormonal or Targeted therapies	Male	July
Hormonal or Targeted therapies	Male	July
Hormonal or Targeted therapies	Female	July
Hormonal or Targeted therapies	Female	July
Hormonal or Targeted therapies	Female	July
Hormonal or Targeted therapies	Female	July
Hormonal or Targeted therapies	Male	June
Hormonal or Targeted therapies	Female	June
Hormonal or Targeted therapies	Female	June
Hormonal or Targeted therapies	Male	June
Hormonal or Targeted therapies	Male	June
Hormonal or Targeted therapies	Male	June
Hormonal or Targeted therapies	Female	June

Hormonal or Targeted therapies	Female	March
Hormonal or Targeted therapies	Female	March
Hormonal or Targeted therapies	Male	March
Hormonal or Targeted therapies	Male	May
Hormonal or Targeted therapies	Male	May
Hormonal or Targeted therapies	Female	May
Hormonal or Targeted therapies	Female	May
Hormonal or Targeted therapies	Female	November
Hormonal or Targeted therapies	Female	October
Hormonal or Targeted therapies	Female	October
Hormonal or Targeted therapies	Female	October
Hormonal or Targeted therapies	Female	October
Hormonal or Targeted therapies	Female	October
Hormonal or Targeted therapies	Female	October
Hormonal or Targeted therapies	Female	September
Hormonal or Targeted therapies	Female	September
Hormonal or Targeted therapies	Female	September
Hormonal or Targeted therapies	Female	September
Hormonal or Targeted therapies	Female	April
Hormonal or Targeted therapies	Female	April
Hormonal or Targeted therapies	Female	April
Hormonal or Targeted therapies	Female	April
Hormonal or Targeted therapies	Male	April
Hormonal or Targeted therapies	Male	April
Hormonal or Targeted therapies	Male	August
Hormonal or Targeted therapies	Female	August
Hormonal or Targeted therapies	Female	August
Hormonal or Targeted therapies	Male	August
Hormonal or Targeted therapies	Male	August
Hormonal or Targeted therapies	Male	August
Hormonal or Targeted therapies	Female	August
Hormonal or Targeted therapies	Female	August
Hormonal or Targeted therapies	Male	August
Hormonal or Targeted therapies	Female	December
Hormonal or Targeted therapies	Female	December
Hormonal or Targeted therapies	Male	December
Hormonal or Targeted therapies	Female	December
Hormonal or Targeted therapies	Male	February
Hormonal or Targeted therapies	Female	February
Hormonal or Targeted therapies	Male	February
Hormonal or Targeted therapies	Female	February
Hormonal or Targeted therapies	Male	February
Hormonal or Targeted therapies	Male	February
Hormonal or Targeted therapies	Male	February
Hormonal or Targeted therapies	Female	February
Hormonal or Targeted therapies	Female	February
Hormonal or Targeted therapies	Female	February
Hormonal or Targeted therapies	Female	January
Hormonal or Targeted therapies	Male	January
Hormonal or Targeted therapies	Female	January

Hormonal or Targeted therapies	Female	July
Hormonal or Targeted therapies	Male	July
Hormonal or Targeted therapies	Male	July
Hormonal or Targeted therapies	Female	July
Hormonal or Targeted therapies	Male	July
Hormonal or Targeted therapies	Male	June
Hormonal or Targeted therapies	Female	June
Hormonal or Targeted therapies	Female	June
Hormonal or Targeted therapies	Female	June
Hormonal or Targeted therapies	Female	June
Hormonal or Targeted therapies	Female	June
Hormonal or Targeted therapies	Male	June
Hormonal or Targeted therapies	Female	June
Hormonal or Targeted therapies	Male	June
Hormonal or Targeted therapies	Female	June
Hormonal or Targeted therapies	Male	June
Hormonal or Targeted therapies	Male	June
Hormonal or Targeted therapies	Female	March
Hormonal or Targeted therapies	Male	March
Hormonal or Targeted therapies	Female	March
Hormonal or Targeted therapies	Female	March
Hormonal or Targeted therapies	Female	March
Hormonal or Targeted therapies	Male	March
Hormonal or Targeted therapies	Male	May
Hormonal or Targeted therapies	Female	May
Hormonal or Targeted therapies	Female	May
Hormonal or Targeted therapies	Male	May
Hormonal or Targeted therapies	Female	May
Hormonal or Targeted therapies	Male	November
Hormonal or Targeted therapies	Female	November
Hormonal or Targeted therapies	Female	November
Hormonal or Targeted therapies	Female	November
Hormonal or Targeted therapies	Female	November
Hormonal or Targeted therapies	Female	November
Hormonal or Targeted therapies	Female	November
Hormonal or Targeted therapies	Male	October
Hormonal or Targeted therapies	Male	October
Hormonal or Targeted therapies	Female	October
Hormonal or Targeted therapies	Male	October
Hormonal or Targeted therapies	Female	October
Hormonal or Targeted therapies	Male	September
Hormonal or Targeted therapies	Female	September
Hormonal or Targeted therapies	Male	September
Hormonal or Targeted therapies	Male	September
Chemotherapy	Male	April
Chemotherapy	Male	April
Chemotherapy	Male	August
Chemotherapy	Female	August
Chemotherapy	Male	August
Chemotherapy	Female	August

Chemotherapy	Male	August
Chemotherapy	Male	December
Chemotherapy	Female	December
Chemotherapy	Male	January
Chemotherapy	Female	July
Chemotherapy	Male	July
Chemotherapy	Male	July
Chemotherapy	Female	July
Chemotherapy	Female	July
Chemotherapy	Male	July
Chemotherapy	Male	June
Chemotherapy	Female	June
Chemotherapy	Male	June
Chemotherapy	Female	June
Chemotherapy	Male	June
Chemotherapy	Female	March
Chemotherapy	Female	March
Chemotherapy	Male	March
Chemotherapy	Male	March
Chemotherapy	Male	March
Chemotherapy	Female	May
Chemotherapy	Male	May
Chemotherapy	Male	May
Chemotherapy	Female	May
Chemotherapy	Male	May
Chemotherapy	Female	May
Chemotherapy	Female	May
Chemotherapy	Male	May
Chemotherapy	Female	November
Chemotherapy	Male	November
Chemotherapy	Male	November
Chemotherapy	Female	October
Chemotherapy	Male	October
Chemotherapy	Male	October
Chemotherapy	Male	September
Chemotherapy	Female	September
Chemotherapy	Female	September
Chemotherapy	Female	September
Chemotherapy	Female	April
Chemotherapy	Male	April
Chemotherapy	Male	April
Chemotherapy	Female	April
Chemotherapy	Female	August
Chemotherapy	Male	August
Chemotherapy	Female	August
Chemotherapy	Female	August
Chemotherapy	Female	August
Chemotherapy	Male	August
Chemotherapy	Male	August
Chemotherapy	Female	December

Chemotherapy	Female	December
Chemotherapy	Male	December
Chemotherapy	Male	January
Chemotherapy	Female	July
Chemotherapy	Female	July
Chemotherapy	Male	July
Chemotherapy	Female	July
Chemotherapy	Female	July
Chemotherapy	Male	July
Chemotherapy	Male	June
Chemotherapy	Female	June
Chemotherapy	Female	June
Chemotherapy	Male	June
Chemotherapy	Male	March
Chemotherapy	Female	March
Chemotherapy	Male	March
Chemotherapy	Female	March
Chemotherapy	Female	May
Chemotherapy	Male	May
Chemotherapy	Male	May
Chemotherapy	Male	May
Chemotherapy	Male	May
Chemotherapy	Female	May
Chemotherapy	Female	May
Chemotherapy	Female	May
Chemotherapy	Male	May
Chemotherapy	Female	May
Chemotherapy	Male	May
Chemotherapy	Female	November
Chemotherapy	Female	November
Chemotherapy	Male	November
Chemotherapy	Male	November
Chemotherapy	Female	November
Chemotherapy	Female	October
Chemotherapy	Male	October
Chemotherapy	Male	October
Chemotherapy	Female	October
Chemotherapy	Female	September
Chemotherapy	Male	September
Chemotherapy	Female	September
Chemotherapy	Male	September
Chemotherapy	Female	September
Chemotherapy	Male	September
Chemotherapy	Female	April
Chemotherapy	Female	April
Chemotherapy	Male	April
Chemotherapy	Female	April
Chemotherapy	Female	April
Chemotherapy	Male	April
Chemotherapy	Female	April

Chemotherapy	Female	April
Chemotherapy	Female	April
Chemotherapy	Male	April
Chemotherapy	Female	April
Chemotherapy	Female	August
Chemotherapy	Female	August
Chemotherapy	Male	August
Chemotherapy	Female	August
Chemotherapy	Female	August
Chemotherapy	Male	August
Chemotherapy	Male	August
Chemotherapy	Female	August
Chemotherapy	Male	August
Chemotherapy	Female	August
Chemotherapy	Male	December
Chemotherapy	Female	December
Chemotherapy	Female	December
Chemotherapy	Male	December
Chemotherapy	Female	December
Chemotherapy	Female	December
Chemotherapy	Female	December
Chemotherapy	Female	December
Chemotherapy	Female	December
Chemotherapy	Male	December
Chemotherapy	Female	February
Chemotherapy	Female	February
Chemotherapy	Female	February
Chemotherapy	Female	February
Chemotherapy	Male	February
Chemotherapy	Female	February
Chemotherapy	Female	February
Chemotherapy	Female	February
Chemotherapy	Female	February
Chemotherapy	Female	January
Chemotherapy	Female	January
Chemotherapy	Male	January
Chemotherapy	Female	January
Chemotherapy	Female	July
Chemotherapy	Female	July
Chemotherapy	Female	July
Chemotherapy	Female	July
Chemotherapy	Male	July
Chemotherapy	Female	July
Chemotherapy	Male	July
Chemotherapy	Male	July
Chemotherapy	Female	July
Chemotherapy	Male	July
Chemotherapy	Female	July
Chemotherapy	Female	June
Chemotherapy	Female	June

Chemotherapy	Male	June
Chemotherapy	Male	June
Chemotherapy	Female	June
Chemotherapy	Female	June
Chemotherapy	Male	June
Chemotherapy	Male	June
Chemotherapy	Female	June
Chemotherapy	Male	March
Chemotherapy	Female	March
Chemotherapy	Male	March
Chemotherapy	Female	March
Chemotherapy	Female	March
Chemotherapy	Female	March
Chemotherapy	Male	March
Chemotherapy	Female	March
Chemotherapy	Female	March
Chemotherapy	Male	March
Chemotherapy	Male	May
Chemotherapy	Female	May
Chemotherapy	Female	May
Chemotherapy	Female	May
Chemotherapy	Male	May
Chemotherapy	Female	May
Chemotherapy	Female	May
Chemotherapy	Male	May
Chemotherapy	Female	May
Chemotherapy	Female	May
Chemotherapy	Female	May
Chemotherapy	Male	May
Chemotherapy	Female	November
Chemotherapy	Male	November
Chemotherapy	Male	November
Chemotherapy	Male	November
Chemotherapy	Female	November
Chemotherapy	Female	November
Chemotherapy	Female	November
Chemotherapy	Female	November
Chemotherapy	Female	November
Chemotherapy	Female	October
Chemotherapy	Female	October
Chemotherapy	Male	October
Chemotherapy	Male	October
Chemotherapy	Female	October
Chemotherapy	Male	October
Chemotherapy	Female	October
Chemotherapy	Female	October
Chemotherapy	Female	October
Chemotherapy	Male	October
Chemotherapy	Female	October
Chemotherapy	Female	October

Chemotherapy	Male	September
Chemotherapy	Male	September
Chemotherapy	Female	September
Chemotherapy	Female	September
Chemotherapy	Male	September
Chemotherapy	Female	September
Chemotherapy	Female	September
Chemotherapy	Female	September
Chemotherapy	Male	September
Chemotherapy	Female	September
Chemotherapy	Female	September
Chemotherapy	Female	September
Chemotherapy	Male	September
Chemotherapy	Female	April
Chemotherapy	Male	April
Chemotherapy	Female	April
Chemotherapy	Male	April
Chemotherapy	Female	April
Chemotherapy	Male	April
Chemotherapy	Female	April
Chemotherapy	Female	April
Chemotherapy	Female	April
Chemotherapy	Male	April
Chemotherapy	Female	August
Chemotherapy	Male	August
Chemotherapy	Male	August
Chemotherapy	Female	August
Chemotherapy	Female	August
Chemotherapy	Male	August
Chemotherapy	Female	August
Chemotherapy	Male	August
Chemotherapy	Female	August
Chemotherapy	Male	August
Chemotherapy	Female	August
Chemotherapy	Female	August
Chemotherapy	Male	December
Chemotherapy	Female	December
Chemotherapy	Male	December
Chemotherapy	Female	December
Chemotherapy	Female	December
Chemotherapy	Female	December
Chemotherapy	Female	December
Chemotherapy	Male	December
Chemotherapy	Male	December
Chemotherapy	Female	December
Chemotherapy	Male	December
Chemotherapy	Female	December
Chemotherapy	Female	December
Chemotherapy	Female	December
Chemotherapy	Male	December

Chemotherapy	Male	February
Chemotherapy	Female	February
Chemotherapy	Female	February
Chemotherapy	Female	February
Chemotherapy	Female	February
Chemotherapy	Male	February
Chemotherapy	Female	February
Chemotherapy	Female	January
Chemotherapy	Female	January
Chemotherapy	Male	January
Chemotherapy	Female	January
Chemotherapy	Male	January
Chemotherapy	Female	January
Chemotherapy	Female	January
Chemotherapy	Male	January
Chemotherapy	Female	January
Chemotherapy	Female	January
Chemotherapy	Female	July
Chemotherapy	Female	July
Chemotherapy	Male	July
Chemotherapy	Female	July
Chemotherapy	Female	July
Chemotherapy	Male	July
Chemotherapy	Male	July
Chemotherapy	Female	July
Chemotherapy	Female	July
Chemotherapy	Male	July
Chemotherapy	Male	July
Chemotherapy	Male	July
Chemotherapy	Female	July
Chemotherapy	Female	June
Chemotherapy	Male	June
Chemotherapy	Female	June
Chemotherapy	Female	June
Chemotherapy	Female	June
Chemotherapy	Male	June
Chemotherapy	Female	June
Chemotherapy	Female	June
Chemotherapy	Male	June
Chemotherapy	Male	June
Chemotherapy	Female	June
Chemotherapy	Female	June
Chemotherapy	Female	June
Chemotherapy	Male	June
Chemotherapy	Female	March
Chemotherapy	Male	March
Chemotherapy	Female	March
Chemotherapy	Male	March
Chemotherapy	Male	March
Chemotherapy	Female	March

Chemotherapy	Female	March
Chemotherapy	Female	March
Chemotherapy	Female	March
Chemotherapy	Female	March
Chemotherapy	Male	March
Chemotherapy	Female	March
Chemotherapy	Female	May
Chemotherapy	Male	May
Chemotherapy	Female	May
Chemotherapy	Male	May
Chemotherapy	Female	May
Chemotherapy	Female	May
Chemotherapy	Female	May
Chemotherapy	Female	May
Chemotherapy	Male	May
Chemotherapy	Female	May
Chemotherapy	Male	May
Chemotherapy	Female	May
Chemotherapy	Male	May
Chemotherapy	Female	May
Chemotherapy	Female	November
Chemotherapy	Male	November
Chemotherapy	Female	November
Chemotherapy	Female	November
Chemotherapy	Female	November
Chemotherapy	Male	November
Chemotherapy	Female	November
Chemotherapy	Female	November
Chemotherapy	Male	November
Chemotherapy	Male	November
Chemotherapy	Male	November
Chemotherapy	Male	November
Chemotherapy	Female	November
Chemotherapy	Male	October
Chemotherapy	Female	October
Chemotherapy	Male	October
Chemotherapy	Female	October
Chemotherapy	Male	October
Chemotherapy	Female	October
Chemotherapy	Female	October
Chemotherapy	Female	October
Chemotherapy	Male	October
Chemotherapy	Female	October
Chemotherapy	Female	October
Chemotherapy	Male	October
Chemotherapy	Female	October
Chemotherapy	Female	October
Chemotherapy	Male	October
Chemotherapy	Male	September
Chemotherapy	Female	September

Chemotherapy	Male	September
Chemotherapy	Female	September
Chemotherapy	Female	September
Chemotherapy	Male	September
Chemotherapy	Female	September
Chemotherapy	Male	September
Chemotherapy	Female	September
Chemotherapy	Male	September
Chemotherapy	Female	September
Chemotherapy	Male	September
Chemotherapy	Female	September
Chemotherapy	Female	September
Chemotherapy	Female	September
Chemotherapy	Male	September
Chemotherapy	Male	April
Chemotherapy	Female	April
Chemotherapy	Female	April
Chemotherapy	Female	April
Chemotherapy	Female	April
Chemotherapy	Male	April
Chemotherapy	Female	April
Chemotherapy	Male	April
Chemotherapy	Female	April
Chemotherapy	Male	April
Chemotherapy	Male	April
Chemotherapy	Male	April
Chemotherapy	Female	April
Chemotherapy	Female	April
Chemotherapy	Female	April
Chemotherapy	Male	April
Chemotherapy	Male	August
Chemotherapy	Male	August
Chemotherapy	Male	August
Chemotherapy	Female	August
Chemotherapy	Male	August
Chemotherapy	Female	August
Chemotherapy	Female	August
Chemotherapy	Male	August
Chemotherapy	Male	August
Chemotherapy	Female	August
Chemotherapy	Male	August
Chemotherapy	Female	August
Chemotherapy	Female	August
Chemotherapy	Male	August
Chemotherapy	Female	December
Chemotherapy	Male	December
Chemotherapy	Male	December
Chemotherapy	Female	December
Chemotherapy	Male	December
Chemotherapy	Male	December

Chemotherapy	Female	December
Chemotherapy	Male	December
Chemotherapy	Female	December
Chemotherapy	Male	December
Chemotherapy	Female	December
Chemotherapy	Male	February
Chemotherapy	Male	February
Chemotherapy	Female	February
Chemotherapy	Male	February
Chemotherapy	Female	February
Chemotherapy	Female	February
Chemotherapy	Male	February
Chemotherapy	Male	February
Chemotherapy	Female	February
Chemotherapy	Female	February
Chemotherapy	Male	January
Chemotherapy	Female	January
Chemotherapy	Male	January
Chemotherapy	Female	January
Chemotherapy	Female	January
Chemotherapy	Male	January
Chemotherapy	Male	January
Chemotherapy	Male	January
Chemotherapy	Female	January
Chemotherapy	Male	January
Chemotherapy	Male	January
Chemotherapy	Female	July
Chemotherapy	Male	July
Chemotherapy	Female	July
Chemotherapy	Male	July
Chemotherapy	Male	July
Chemotherapy	Female	July
Chemotherapy	Male	July
Chemotherapy	Male	July
Chemotherapy	Female	July
Chemotherapy	Male	July
Chemotherapy	Female	July
Chemotherapy	Male	July
Chemotherapy	Male	June
Chemotherapy	Female	June
Chemotherapy	Male	June
Chemotherapy	Female	June
Chemotherapy	Male	June
Chemotherapy	Female	June
Chemotherapy	Male	June
Chemotherapy	Male	June
Chemotherapy	Male	June
Chemotherapy	Female	June
Chemotherapy	Male	June
Chemotherapy	Male	June

Chemotherapy	Female	June
Chemotherapy	Female	March
Chemotherapy	Male	March
Chemotherapy	Female	March
Chemotherapy	Male	March
Chemotherapy	Female	March
Chemotherapy	Male	March
Chemotherapy	Male	March
Chemotherapy	Female	March
Chemotherapy	Male	March
Chemotherapy	Female	March
Chemotherapy	Male	March
Chemotherapy	Female	March
Chemotherapy	Male	March
Chemotherapy	Female	March
Chemotherapy	Male	March
Chemotherapy	Female	March
Chemotherapy	Male	March
Chemotherapy	Male	May
Chemotherapy	Male	May
Chemotherapy	Female	May
Chemotherapy	Female	May
Chemotherapy	Female	May
Chemotherapy	Female	May
Chemotherapy	Male	May
Chemotherapy	Female	May
Chemotherapy	Male	May
Chemotherapy	Male	May
Chemotherapy	Male	May
Chemotherapy	Female	May
Chemotherapy	Male	May
Chemotherapy	Female	May
Chemotherapy	Male	May
Chemotherapy	Male	November
Chemotherapy	Female	November
Chemotherapy	Female	November
Chemotherapy	Male	November
Chemotherapy	Male	November
Chemotherapy	Female	November
Chemotherapy	Female	November
Chemotherapy	Male	November
Chemotherapy	Male	November
Chemotherapy	Female	November
Chemotherapy	Female	October
Chemotherapy	Male	October
Chemotherapy	Male	October
Chemotherapy	Female	October
Chemotherapy	Male	October
Chemotherapy	Female	October
Chemotherapy	Male	October

Chemotherapy	Male	October
Chemotherapy	Female	October
Chemotherapy	Female	October
Chemotherapy	Female	October
Chemotherapy	Female	October
Chemotherapy	Male	October
Chemotherapy	Male	October
Chemotherapy	Male	September
Chemotherapy	Female	September
Chemotherapy	Female	September
Chemotherapy	Male	September
Chemotherapy	Male	September
Chemotherapy	Female	September
Chemotherapy	Female	September
Chemotherapy	Male	September
Chemotherapy	Male	September
Chemotherapy	Male	September
Chemotherapy	Female	September
Chemotherapy	Female	September
Chemotherapy	Male	September
Chemotherapy	Male	April
Chemotherapy	Female	April
Chemotherapy	Female	April
Chemotherapy	Female	April
Chemotherapy	Male	April
Chemotherapy	Female	April
Chemotherapy	Male	April
Chemotherapy	Male	April
Chemotherapy	Female	April
Chemotherapy	Female	April
Chemotherapy	Male	April
Chemotherapy	Female	April
Chemotherapy	Male	April
Chemotherapy	Male	April
Chemotherapy	Female	April
Chemotherapy	Female	August
Chemotherapy	Male	August
Chemotherapy	Female	August
Chemotherapy	Female	August
Chemotherapy	Male	August
Chemotherapy	Male	August
Chemotherapy	Male	August
Chemotherapy	Female	August
Chemotherapy	Female	August
Chemotherapy	Female	August
Chemotherapy	Male	August
Chemotherapy	Female	August
Chemotherapy	Male	August
Chemotherapy	Male	December
Chemotherapy	Male	December

Chemotherapy	Female	December
Chemotherapy	Female	December
Chemotherapy	Female	December
Chemotherapy	Female	December
Chemotherapy	Male	December
Chemotherapy	Male	December
Chemotherapy	Male	December
Chemotherapy	Female	December
Chemotherapy	Female	December
Chemotherapy	Female	December
Chemotherapy	Male	December
Chemotherapy	Male	February
Chemotherapy	Female	February
Chemotherapy	Female	February
Chemotherapy	Male	February
Chemotherapy	Female	February
Chemotherapy	Female	February
Chemotherapy	Male	February
Chemotherapy	Male	February
Chemotherapy	Female	February
Chemotherapy	Female	February
Chemotherapy	Female	February
Chemotherapy	Male	February
Chemotherapy	Male	January
Chemotherapy	Male	January
Chemotherapy	Female	January
Chemotherapy	Female	January
Chemotherapy	Male	January
Chemotherapy	Male	January
Chemotherapy	Male	January
Chemotherapy	Female	January
Chemotherapy	Female	January
Chemotherapy	Female	January
Chemotherapy	Female	January
Chemotherapy	Male	January
Chemotherapy	Male	January
Chemotherapy	Female	July
Chemotherapy	Male	July
Chemotherapy	Female	July
Chemotherapy	Male	July
Chemotherapy	Female	July
Chemotherapy	Male	July
Chemotherapy	Female	July
Chemotherapy	Male	July
Chemotherapy	Female	July
Chemotherapy	Female	July
Chemotherapy	Male	July
Chemotherapy	Female	July
Chemotherapy	Female	July
Chemotherapy	Male	July

Chemotherapy	Male	July
Chemotherapy	Female	July
Chemotherapy	Male	July
Chemotherapy	Male	June
Chemotherapy	Female	June
Chemotherapy	Male	June
Chemotherapy	Female	June
Chemotherapy	Female	June
Chemotherapy	Male	June
Chemotherapy	Male	June
Chemotherapy	Female	June
Chemotherapy	Female	June
Chemotherapy	Male	June
Chemotherapy	Female	June
Chemotherapy	Male	June
Chemotherapy	Male	June
Chemotherapy	Female	June
Chemotherapy	Male	June
Chemotherapy	Female	June
Chemotherapy	Male	March
Chemotherapy	Female	March
Chemotherapy	Male	March
Chemotherapy	Female	March
Chemotherapy	Female	March
Chemotherapy	Male	March
Chemotherapy	Male	March
Chemotherapy	Female	March
Chemotherapy	Female	March
Chemotherapy	Female	March
Chemotherapy	Female	March
Chemotherapy	Male	March
Chemotherapy	Female	March
Chemotherapy	Male	May
Chemotherapy	Male	May
Chemotherapy	Male	May
Chemotherapy	Female	May
Chemotherapy	Female	May
Chemotherapy	Female	May
Chemotherapy	Female	May
Chemotherapy	Female	May
Chemotherapy	Female	May
Chemotherapy	Male	May
Chemotherapy	Female	May
Chemotherapy	Male	May
Chemotherapy	Male	May
Chemotherapy	Female	May
Chemotherapy	Male	May
Chemotherapy	Female	May
Chemotherapy	Male	May
Chemotherapy	Female	May

Chemotherapy	Female	November
Chemotherapy	Male	November
Chemotherapy	Female	November
Chemotherapy	Male	November
Chemotherapy	Female	November
Chemotherapy	Male	November
Chemotherapy	Male	November
Chemotherapy	Female	November
Chemotherapy	Female	November
Chemotherapy	Male	November
Chemotherapy	Female	November
Chemotherapy	Male	November
Chemotherapy	Female	November
Chemotherapy	Male	November
Chemotherapy	Female	November
Chemotherapy	Male	October
Chemotherapy	Female	October
Chemotherapy	Male	October
Chemotherapy	Female	October
Chemotherapy	Female	October
Chemotherapy	Male	October
Chemotherapy	Female	October
Chemotherapy	Female	October
Chemotherapy	Male	October
Chemotherapy	Female	October
Chemotherapy	Male	October
Chemotherapy	Female	October
Chemotherapy	Female	October
Chemotherapy	Male	October
Chemotherapy	Female	October
Chemotherapy	Male	October
Chemotherapy	Male	September
Chemotherapy	Female	September
Chemotherapy	Female	September
Chemotherapy	Female	September
Chemotherapy	Male	September
Chemotherapy	Male	September
Chemotherapy	Female	September
Chemotherapy	Male	September
Chemotherapy	Female	September
Chemotherapy	Male	September
Chemotherapy	Female	September
Chemotherapy	Male	September
Chemotherapy	Female	September
Chemotherapy	Male	September
Chemotherapy	Male	September
Chemotherapy	Male	September
Chemotherapy	Female	September
Chemotherapy	Female	April
Chemotherapy	Male	April

Chemotherapy	Female	April
Chemotherapy	Male	April
Chemotherapy	Female	April
Chemotherapy	Male	April
Chemotherapy	Female	April
Chemotherapy	Male	April
Chemotherapy	Female	April
Chemotherapy	Male	April
Chemotherapy	Female	April
Chemotherapy	Male	April
Chemotherapy	Female	April
Chemotherapy	Female	April
Chemotherapy	Male	April
Chemotherapy	Male	August
Chemotherapy	Female	August
Chemotherapy	Female	August
Chemotherapy	Male	August
Chemotherapy	Female	August
Chemotherapy	Female	August
Chemotherapy	Female	August
Chemotherapy	Female	August
Chemotherapy	Female	August
Chemotherapy	Male	August
Chemotherapy	Female	August
Chemotherapy	Male	August
Chemotherapy	Male	August
Chemotherapy	Male	August
Chemotherapy	Female	December
Chemotherapy	Male	December
Chemotherapy	Male	December
Chemotherapy	Female	December
Chemotherapy	Male	December
Chemotherapy	Male	December
Chemotherapy	Female	December
Chemotherapy	Male	December
Chemotherapy	Female	December
Chemotherapy	Female	December
Chemotherapy	Male	December
Chemotherapy	Male	December
Chemotherapy	Male	December
Chemotherapy	Female	February
Chemotherapy	Male	February
Chemotherapy	Female	February
Chemotherapy	Male	February
Chemotherapy	Female	February
Chemotherapy	Male	February
Chemotherapy	Male	February
Chemotherapy	Female	February
Chemotherapy	Female	February
Chemotherapy	Male	February

Chemotherapy	Female	February
Chemotherapy	Male	February
Chemotherapy	Female	February
Chemotherapy	Male	January
Chemotherapy	Female	January
Chemotherapy	Female	January
Chemotherapy	Male	January
Chemotherapy	Male	January
Chemotherapy	Male	January
Chemotherapy	Female	January
Chemotherapy	Female	January
Chemotherapy	Male	January
Chemotherapy	Female	January
Chemotherapy	Male	January
Chemotherapy	Male	January
Chemotherapy	Female	January
Chemotherapy	Male	January
Chemotherapy	Female	January
Chemotherapy	Female	January
Chemotherapy	Female	July
Chemotherapy	Male	July
Chemotherapy	Female	July
Chemotherapy	Male	July
Chemotherapy	Female	July
Chemotherapy	Female	July
Chemotherapy	Male	July
Chemotherapy	Female	July
Chemotherapy	Female	July
Chemotherapy	Female	July
Chemotherapy	Male	July
Chemotherapy	Male	July
Chemotherapy	Female	July
Chemotherapy	Female	July
Chemotherapy	Male	July
Chemotherapy	Female	July
Chemotherapy	Female	July
Chemotherapy	Male	July
Chemotherapy	Male	June
Chemotherapy	Female	June
Chemotherapy	Male	June
Chemotherapy	Female	June
Chemotherapy	Female	June
Chemotherapy	Female	June
Chemotherapy	Male	June
Chemotherapy	Female	June
Chemotherapy	Male	June
Chemotherapy	Male	June
Chemotherapy	Male	June
Chemotherapy	Male	June
Chemotherapy	Female	June

Chemotherapy	Female	June
Chemotherapy	Female	June
Chemotherapy	Female	March
Chemotherapy	Male	March
Chemotherapy	Female	March
Chemotherapy	Male	March
Chemotherapy	Female	March
Chemotherapy	Male	March
Chemotherapy	Male	March
Chemotherapy	Female	March
Chemotherapy	Female	March
Chemotherapy	Male	March
Chemotherapy	Female	March
Chemotherapy	Male	March
Chemotherapy	Female	March
Chemotherapy	Female	March
Chemotherapy	Male	March
Chemotherapy	Male	May
Chemotherapy	Female	May
Chemotherapy	Female	May
Chemotherapy	Male	May
Chemotherapy	Female	May
Chemotherapy	Female	May
Chemotherapy	Female	May
Chemotherapy	Male	May
Chemotherapy	Male	May
Chemotherapy	Female	May
Chemotherapy	Male	May
Chemotherapy	Male	May
Chemotherapy	Female	May
Chemotherapy	Male	May
Chemotherapy	Male	May
Chemotherapy	Female	May
Chemotherapy	Female	May
Chemotherapy	Female	November
Chemotherapy	Male	November
Chemotherapy	Male	November
Chemotherapy	Female	November
Chemotherapy	Male	November
Chemotherapy	Male	November
Chemotherapy	Female	November
Chemotherapy	Female	November
Chemotherapy	Male	November
Chemotherapy	Female	November
Chemotherapy	Male	November
Chemotherapy	Female	November
Chemotherapy	Male	November
Chemotherapy	Female	November
Chemotherapy	Male	November
Chemotherapy	Female	October

Chemotherapy	Male	October
Chemotherapy	Female	October
Chemotherapy	Male	October
Chemotherapy	Female	October
Chemotherapy	Female	October
Chemotherapy	Male	October
Chemotherapy	Female	October
Chemotherapy	Male	October
Chemotherapy	Male	October
Chemotherapy	Female	October
Chemotherapy	Female	October
Chemotherapy	Male	October
Chemotherapy	Male	September
Chemotherapy	Female	September
Chemotherapy	Male	September
Chemotherapy	Female	September
Chemotherapy	Male	September
Chemotherapy	Female	September
Chemotherapy	Male	September
Chemotherapy	Female	September
Chemotherapy	Male	September
Chemotherapy	Female	September
Chemotherapy	Female	September
Chemotherapy	Male	September
Chemotherapy	Female	September
Chemotherapy	Male	September
Chemotherapy	Male	September
Chemotherapy	Male	September
Chemotherapy	Male	September
Chemotherapy	Female	September
Chemotherapy	Female	September
Chemotherapy	Male	April
Chemotherapy	Female	April
Chemotherapy	Male	April
Chemotherapy	Male	April
Chemotherapy	Female	April
Chemotherapy	Female	April
Chemotherapy	Female	April
Chemotherapy	Female	April
Chemotherapy	Male	April
Chemotherapy	Female	April
Chemotherapy	Male	April
Chemotherapy	Female	April
Chemotherapy	Female	April
Chemotherapy	Female	April
Chemotherapy	Male	August
Chemotherapy	Female	August
Chemotherapy	Male	August
Chemotherapy	Male	August
Chemotherapy	Male	August

Chemotherapy	Female	August
Chemotherapy	Male	August
Chemotherapy	Female	August
Chemotherapy	Female	December
Chemotherapy	Female	December
Chemotherapy	Male	December
Chemotherapy	Male	December
Chemotherapy	Male	December
Chemotherapy	Female	December
Chemotherapy	Female	December
Chemotherapy	Male	December
Chemotherapy	Female	December
Chemotherapy	Female	December
Chemotherapy	Male	December
Chemotherapy	Male	December
Chemotherapy	Male	December
Chemotherapy	Male	December
Chemotherapy	Female	December
Chemotherapy	Female	February
Chemotherapy	Male	February
Chemotherapy	Male	February
Chemotherapy	Male	February
Chemotherapy	Male	February
Chemotherapy	Female	February
Chemotherapy	Male	February
Chemotherapy	Female	February
Chemotherapy	Male	February
Chemotherapy	Female	February
Chemotherapy	Male	February
Chemotherapy	Female	February
Chemotherapy	Female	February
Chemotherapy	Female	February
Chemotherapy	Male	February
Chemotherapy	Male	February
Chemotherapy	Female	January
Chemotherapy	Female	January
Chemotherapy	Male	January
Chemotherapy	Female	January
Chemotherapy	Female	January
Chemotherapy	Female	January
Chemotherapy	Male	January
Chemotherapy	Male	January
Chemotherapy	Female	January
Chemotherapy	Male	January
Chemotherapy	Female	January
Chemotherapy	Male	January
Chemotherapy	Female	July
Chemotherapy	Male	July
Chemotherapy	Male	July
Chemotherapy	Male	July

Chemotherapy	Female	July
Chemotherapy	Female	July
Chemotherapy	Male	July
Chemotherapy	Male	July
Chemotherapy	Female	June
Chemotherapy	Male	June
Chemotherapy	Male	June
Chemotherapy	Male	June
Chemotherapy	Female	June
Chemotherapy	Male	June
Chemotherapy	Female	June
Chemotherapy	Female	June
Chemotherapy	Female	March
Chemotherapy	Male	March
Chemotherapy	Male	March
Chemotherapy	Female	March
Chemotherapy	Male	March
Chemotherapy	Female	March
Chemotherapy	Male	March
Chemotherapy	Male	March
Chemotherapy	Female	March
Chemotherapy	Male	May
Chemotherapy	Male	May
Chemotherapy	Male	May
Chemotherapy	Female	May
Chemotherapy	Female	May
Chemotherapy	Male	May
Chemotherapy	Female	May
Chemotherapy	Male	May
Chemotherapy	Female	May
Chemotherapy	Male	May
Chemotherapy	Female	May
Chemotherapy	Female	May
Chemotherapy	Female	November
Chemotherapy	Male	November
Chemotherapy	Female	November
Chemotherapy	Male	November
Chemotherapy	Male	November
Chemotherapy	Male	November
Chemotherapy	Male	November
Chemotherapy	Female	November
Chemotherapy	Male	November
Chemotherapy	Male	November
Chemotherapy	Male	November
Chemotherapy	Female	November
Chemotherapy	Female	November
Chemotherapy	Male	November
Chemotherapy	Female	November
Chemotherapy	Female	November
Chemotherapy	Female	November

Chemotherapy	Female	October
Chemotherapy	Female	October
Chemotherapy	Male	October
Chemotherapy	Male	October
Chemotherapy	Female	October
Chemotherapy	Male	October
Chemotherapy	Female	October
Chemotherapy	Male	October
Chemotherapy	Male	October
Chemotherapy	Female	October
Chemotherapy	Male	October
Chemotherapy	Female	September
Chemotherapy	Female	September
Chemotherapy	Male	September
Chemotherapy	Female	September
Chemotherapy	Female	September
Chemotherapy	Female	September
Chemotherapy	Male	September
Chemotherapy	Female	September
Chemotherapy	Male	September
Chemotherapy	Male	September
Chemotherapy	Male	April
Chemotherapy	Female	April
Chemotherapy	Male	April
Chemotherapy	Male	April
Chemotherapy	Female	April
Chemotherapy	Female	April
Chemotherapy	Female	April
Chemotherapy	Male	April
Chemotherapy	Male	April
Chemotherapy	Female	April
Chemotherapy	Male	April
Chemotherapy	Male	April
Chemotherapy	Female	April
Chemotherapy	Male	April
Chemotherapy	Female	April
Chemotherapy	Female	April
Chemotherapy	Male	April
Chemotherapy	Male	April
Chemotherapy	Female	April
Chemotherapy	Male	April
Chemotherapy	Male	August
Chemotherapy	Female	August
Chemotherapy	Female	August
Chemotherapy	Male	August
Chemotherapy	Male	August
Chemotherapy	Female	August
Chemotherapy	Male	August
Chemotherapy	Female	August
Chemotherapy	Female	August

Chemotherapy	Female	August
Chemotherapy	Female	August
Chemotherapy	Female	August
Chemotherapy	Female	August
Chemotherapy	Male	August
Chemotherapy	Male	August
Chemotherapy	Female	August
Chemotherapy	Male	August
Chemotherapy	Female	December
Chemotherapy	Female	December
Chemotherapy	Female	December
Chemotherapy	Female	December
Chemotherapy	Female	December
Chemotherapy	Male	December
Chemotherapy	Female	December
Chemotherapy	Female	December
Chemotherapy	Male	December
Chemotherapy	Male	December
Chemotherapy	Female	December
Chemotherapy	Male	December
Chemotherapy	Male	December
Chemotherapy	Female	December
Chemotherapy	Female	December
Chemotherapy	Male	December
Chemotherapy	Female	December
Chemotherapy	Male	December
Chemotherapy	Male	December
Chemotherapy	Female	December
Chemotherapy	Male	December
Chemotherapy	Female	December
Chemotherapy	Female	December
Chemotherapy	Female	December
Chemotherapy	Male	December
Chemotherapy	Male	December
Chemotherapy	Female	December
Chemotherapy	Female	December
Chemotherapy	Female	February
Chemotherapy	Female	February
Chemotherapy	Female	February
Chemotherapy	Male	February
Chemotherapy	Male	February
Chemotherapy	Male	February
Chemotherapy	Male	February
Chemotherapy	Female	February
Chemotherapy	Female	February
Chemotherapy	Male	February
Chemotherapy	Female	February
Chemotherapy	Female	February
Chemotherapy	Female	February
Chemotherapy	Male	February

Chemotherapy	Female	February
Chemotherapy	Male	February
Chemotherapy	Male	February
Chemotherapy	Female	February
Chemotherapy	Male	January
Chemotherapy	Female	January
Chemotherapy	Female	January
Chemotherapy	Female	January
Chemotherapy	Female	January
Chemotherapy	Female	January
Chemotherapy	Female	January
Chemotherapy	Male	January
Chemotherapy	Male	January
Chemotherapy	Female	January
Chemotherapy	Male	January
Chemotherapy	Male	January
Chemotherapy	Female	January
Chemotherapy	Female	January
Chemotherapy	Male	January
Chemotherapy	Male	July
Chemotherapy	Female	July
Chemotherapy	Male	July
Chemotherapy	Male	July
Chemotherapy	Female	July
Chemotherapy	Female	July
Chemotherapy	Male	July
Chemotherapy	Male	July
Chemotherapy	Female	July
Chemotherapy	Male	July
Chemotherapy	Female	July
Chemotherapy	Male	July
Chemotherapy	Female	July
Chemotherapy	Female	July
Chemotherapy	Male	July
Chemotherapy	Male	July
Chemotherapy	Female	July
Chemotherapy	Female	July
Chemotherapy	Female	June
Chemotherapy	Female	June
Chemotherapy	Female	June
Chemotherapy	Female	June
Chemotherapy	Male	June
Chemotherapy	Male	June
Chemotherapy	Female	June
Chemotherapy	Female	June
Chemotherapy	Female	June
Chemotherapy	Male	June
Chemotherapy	Female	June
Chemotherapy	Female	June
Chemotherapy	Female	June

Chemotherapy	Female	June
Chemotherapy	Male	June
Chemotherapy	Male	March
Chemotherapy	Female	March
Chemotherapy	Female	March
Chemotherapy	Female	March
Chemotherapy	Male	March
Chemotherapy	Male	March
Chemotherapy	Male	March
Chemotherapy	Female	March
Chemotherapy	Female	March
Chemotherapy	Male	March
Chemotherapy	Female	March
Chemotherapy	Female	March
Chemotherapy	Female	March
Chemotherapy	Male	March
Chemotherapy	Female	March
Chemotherapy	Male	March
Chemotherapy	Male	March
Chemotherapy	Female	March
Chemotherapy	Female	May
Chemotherapy	Female	May
Chemotherapy	Female	May
Chemotherapy	Male	May
Chemotherapy	Female	May
Chemotherapy	Female	May
Chemotherapy	Female	May
Chemotherapy	Male	May
Chemotherapy	Male	May
Chemotherapy	Female	May
Chemotherapy	Female	May
Chemotherapy	Male	May
Chemotherapy	Female	May
Chemotherapy	Female	May
Chemotherapy	Male	May
Chemotherapy	Female	May
Chemotherapy	Male	May
Chemotherapy	Male	May
Chemotherapy	Male	May
Chemotherapy	Female	May
Chemotherapy	Male	May
Chemotherapy	Male	May
Chemotherapy	Male	May
Chemotherapy	Male	May
Chemotherapy	Female	November
Chemotherapy	Male	November
Chemotherapy	Female	November
Chemotherapy	Male	November
Chemotherapy	Male	November
Chemotherapy	Female	November

Chemotherapy	Male	November
Chemotherapy	Male	November
Chemotherapy	Male	November
Chemotherapy	Female	November
Chemotherapy	Female	November
Chemotherapy	Female	November
Chemotherapy	Male	October
Chemotherapy	Female	October
Chemotherapy	Male	October
Chemotherapy	Male	October
Chemotherapy	Male	October
Chemotherapy	Male	October
Chemotherapy	Male	October
Chemotherapy	Female	October
Chemotherapy	Female	October
Chemotherapy	Female	October
Chemotherapy	Male	October
Chemotherapy	Female	October
Chemotherapy	Female	October
Chemotherapy	Male	October
Chemotherapy	Male	October
Chemotherapy	Female	October
Chemotherapy	Male	October
Chemotherapy	Male	September
Chemotherapy	Female	September
Chemotherapy	Male	September
Chemotherapy	Female	September
Chemotherapy	Male	September
Chemotherapy	Female	September
Chemotherapy	Female	September
Chemotherapy	Female	September
Chemotherapy	Male	September
Chemotherapy	Female	September
Chemotherapy	Female	September
Chemotherapy	Male	September
Chemotherapy	Male	September
Chemotherapy	Female	September
Chemotherapy	Female	September
Chemotherapy	Male	September
Chemotherapy	Male	September
Chemotherapy	Female	September
Chemotherapy	Female	September
Chemotherapy	Male	September
Chemotherapy	Female	April
Chemotherapy	Female	April
Chemotherapy	Male	April
Chemotherapy	Male	April
Chemotherapy	Male	April
Chemotherapy	Male	April
Chemotherapy	Female	April

Chemotherapy	Female	April
Chemotherapy	Female	April
Chemotherapy	Male	April
Chemotherapy	Female	April
Chemotherapy	Female	April
Chemotherapy	Male	April
Chemotherapy	Male	April
Chemotherapy	Female	April
Chemotherapy	Male	April
Chemotherapy	Female	April
Chemotherapy	Male	April
Chemotherapy	Female	April
Chemotherapy	Female	April
Chemotherapy	Female	April
Chemotherapy	Male	April
Chemotherapy	Female	April
Chemotherapy	Male	April
Chemotherapy	Female	August
Chemotherapy	Male	August
Chemotherapy	Female	August
Chemotherapy	Male	August
Chemotherapy	Male	August
Chemotherapy	Female	August
Chemotherapy	Female	August
Chemotherapy	Male	August
Chemotherapy	Female	August
Chemotherapy	Male	August
Chemotherapy	Male	August
Chemotherapy	Male	August
Chemotherapy	Female	August
Chemotherapy	Male	August
Chemotherapy	Female	August
Chemotherapy	Male	August
Chemotherapy	Female	August
Chemotherapy	Female	August
Chemotherapy	Male	December
Chemotherapy	Male	December
Chemotherapy	Male	December
Chemotherapy	Male	December
Chemotherapy	Male	December
Chemotherapy	Female	December
Chemotherapy	Female	December
Chemotherapy	Male	December
Chemotherapy	Female	December
Chemotherapy	Female	December
Chemotherapy	Male	December
Chemotherapy	Male	December
Chemotherapy	Male	December
Chemotherapy	Female	December
Chemotherapy	Female	December

Chemotherapy	Male	December
Chemotherapy	Male	December
Chemotherapy	Female	December
Chemotherapy	Male	February
Chemotherapy	Male	February
Chemotherapy	Male	February
Chemotherapy	Female	February
Chemotherapy	Male	February
Chemotherapy	Male	February
Chemotherapy	Female	February
Chemotherapy	Male	February
Chemotherapy	Female	February
Chemotherapy	Male	February
Chemotherapy	Female	February
Chemotherapy	Male	February
Chemotherapy	Female	February
Chemotherapy	Female	February
Chemotherapy	Male	February
Chemotherapy	Female	February
Chemotherapy	Male	February
Chemotherapy	Male	February
Chemotherapy	Female	February
Chemotherapy	Female	February
Chemotherapy	Male	February
Chemotherapy	Male	February
Chemotherapy	Male	February
Chemotherapy	Female	February
Chemotherapy	Male	February
Chemotherapy	Male	February
Chemotherapy	Female	February
Chemotherapy	Male	February
Chemotherapy	Male	February
Chemotherapy	Female	February
Chemotherapy	Female	January
Chemotherapy	Male	January
Chemotherapy	Female	January
Chemotherapy	Female	January
Chemotherapy	Female	January
Chemotherapy	Female	January
Chemotherapy	Female	January
Chemotherapy	Female	January
Chemotherapy	Male	January
Chemotherapy	Male	January
Chemotherapy	Female	January
Chemotherapy	Female	January
Chemotherapy	Male	January
Chemotherapy	Male	January
Chemotherapy	Female	January
Chemotherapy	Male	January
Chemotherapy	Male	January

Chemotherapy	Male	July
Chemotherapy	Female	July
Chemotherapy	Female	July
Chemotherapy	Female	July
Chemotherapy	Male	July
Chemotherapy	Male	July
Chemotherapy	Male	July
Chemotherapy	Female	July
Chemotherapy	Female	July
Chemotherapy	Female	July
Chemotherapy	Female	July
Chemotherapy	Male	July
Chemotherapy	Male	July
Chemotherapy	Male	July
Chemotherapy	Female	July
Chemotherapy	Female	July
Chemotherapy	Female	July
Chemotherapy	Female	July
Chemotherapy	Female	July
Chemotherapy	Female	July
Chemotherapy	Male	July
Chemotherapy	Female	July
Chemotherapy	Male	June
Chemotherapy	Male	June
Chemotherapy	Female	June
Chemotherapy	Female	June
Chemotherapy	Male	June
Chemotherapy	Female	June
Chemotherapy	Male	June
Chemotherapy	Female	June
Chemotherapy	Male	June
Chemotherapy	Female	June
Chemotherapy	Female	June
Chemotherapy	Male	June
Chemotherapy	Male	June
Chemotherapy	Male	June
Chemotherapy	Male	June
Chemotherapy	Female	June
Chemotherapy	Female	June
Chemotherapy	Female	June
Chemotherapy	Male	June
Chemotherapy	Female	June
Chemotherapy	Male	June
Chemotherapy	Female	June
Chemotherapy	Male	June
Chemotherapy	Female	June
Chemotherapy	Male	June
Chemotherapy	Female	March
Chemotherapy	Male	March
Chemotherapy	Female	March

Chemotherapy	Female	March
Chemotherapy	Male	March
Chemotherapy	Male	March
Chemotherapy	Female	March
Chemotherapy	Male	March
Chemotherapy	Male	March
Chemotherapy	Female	March
Chemotherapy	Female	March
Chemotherapy	Female	March
Chemotherapy	Female	March
Chemotherapy	Male	March
Chemotherapy	Male	March
Chemotherapy	Female	March
Chemotherapy	Female	March
Chemotherapy	Male	March
Chemotherapy	Male	March
Chemotherapy	Male	March
Chemotherapy	Male	March
Chemotherapy	Male	March
Chemotherapy	Male	May
Chemotherapy	Female	May
Chemotherapy	Male	May
Chemotherapy	Male	May
Chemotherapy	Female	May
Chemotherapy	Female	May
Chemotherapy	Male	May
Chemotherapy	Male	May
Chemotherapy	Female	May
Chemotherapy	Male	May
Chemotherapy	Female	May
Chemotherapy	Male	May
Chemotherapy	Male	May
Chemotherapy	Female	May
Chemotherapy	Male	May
Chemotherapy	Female	May
Chemotherapy	Male	May
Chemotherapy	Male	May
Chemotherapy	Female	May
Chemotherapy	Female	May
Chemotherapy	Female	May
Chemotherapy	Female	May
Chemotherapy	Female	May
Chemotherapy	Male	May
Chemotherapy	Male	May
Chemotherapy	Female	May
Chemotherapy	Female	November
Chemotherapy	Female	November
Chemotherapy	Male	November
Chemotherapy	Male	November
Chemotherapy	Male	November

Chemotherapy	Male	November
Chemotherapy	Male	November
Chemotherapy	Female	November
Chemotherapy	Male	November
Chemotherapy	Male	November
Chemotherapy	Male	November
Chemotherapy	Female	November
Chemotherapy	Male	November
Chemotherapy	Female	November
Chemotherapy	Male	November
Chemotherapy	Female	November
Chemotherapy	Male	November
Chemotherapy	Female	November
Chemotherapy	Female	November
Chemotherapy	Female	November
Chemotherapy	Male	November
Chemotherapy	Female	November
Chemotherapy	Male	November
Chemotherapy	Male	November
Chemotherapy	Female	November
Chemotherapy	Female	October
Chemotherapy	Male	October
Chemotherapy	Female	October
Chemotherapy	Female	October
Chemotherapy	Male	October
Chemotherapy	Female	October
Chemotherapy	Female	October
Chemotherapy	Female	October
Chemotherapy	Male	October
Chemotherapy	Male	October
Chemotherapy	Female	October
Chemotherapy	Male	October
Chemotherapy	Female	October
Chemotherapy	Male	October
Chemotherapy	Male	October
Chemotherapy	Male	October
Chemotherapy	Male	October
Chemotherapy	Male	October
Chemotherapy	Male	October
Chemotherapy	Male	October
Chemotherapy	Male	October
Chemotherapy	Female	October
Chemotherapy	Male	October
Chemotherapy	Male	October
Chemotherapy	Female	October
Chemotherapy	Female	October
Chemotherapy	Male	October
Chemotherapy	Female	September
Chemotherapy	Male	September
Chemotherapy	Male	September

Chemotherapy	Female	September
Chemotherapy	Male	September
Chemotherapy	Female	September
Chemotherapy	Male	September
Chemotherapy	Male	September
Chemotherapy	Female	September
Chemotherapy	Female	September
Chemotherapy	Male	September
Chemotherapy	Male	September
Chemotherapy	Female	September
Chemotherapy	Male	September
Chemotherapy	Male	September
Chemotherapy	Female	September
Chemotherapy	Male	September
Chemotherapy	Female	September
Chemotherapy	Male	September
Chemotherapy	Female	September
Chemotherapy	Male	September
Chemotherapy	Female	September

Value	Age Group
0	30- 39 Yrs
0	30- 39 Yrs
0	30- 39 Yrs
0	30- 39 Yrs
0	30- 39 Yrs
0	30- 39 Yrs
0	30- 39 Yrs
0	30- 39 Yrs
0	30- 39 Yrs
0	30- 39 Yrs
0	30- 39 Yrs
0	30- 39 Yrs
0	30- 39 Yrs
0	30- 39 Yrs
0	30- 39 Yrs
0	30- 39 Yrs
0	30- 39 Yrs
0	30- 39 Yrs
0	30- 39 Yrs
0	30- 39 Yrs
0	30- 39 Yrs
0	30- 39 Yrs
0	30- 39 Yrs
0	30- 39 Yrs
0	30- 39 Yrs
0	30- 39 Yrs
0	30- 39 Yrs
0	30- 39 Yrs
0	30- 39 Yrs
0	30- 39 Yrs
0	30- 39 Yrs
0	30- 39 Yrs
0	30- 39 Yrs
0	30- 39 Yrs
0	30- 39 Yrs
0	30- 39 Yrs
0	30- 39 Yrs
0	30- 39 Yrs
0	30- 39 Yrs
0	30- 39 Yrs
0	30- 39 Yrs
0	30- 39 Yrs
0	30- 39 Yrs
0	30- 39 Yrs
0	30- 39 Yrs
0	30- 39 Yrs
0	30- 39 Yrs
0	30- 39 Yrs
0	30- 39 Yrs

0 30- 39 Yrs
0 30- 39 Yrs
0 30- 39 Yrs
0 30- 39 Yrs
0 30- 39 Yrs
0 30- 39 Yrs
0 30- 39 Yrs
0 30- 39 Yrs
0 30- 39 Yrs
0 30- 39 Yrs
0 30- 39 Yrs
0 30- 39 Yrs
0 30- 39 Yrs
0 30- 39 Yrs
0 30- 39 Yrs
0 30- 39 Yrs
0 30- 39 Yrs
0 30- 39 Yrs
0 30- 39 Yrs
0 30- 39 Yrs
0 30- 39 Yrs
0 30- 39 Yrs
0 30- 39 Yrs
0 30- 39 Yrs
0 30- 39 Yrs
0 30- 39 Yrs
0 30- 39 Yrs
0 30- 39 Yrs
0 30- 39 Yrs
0 30- 39 Yrs
0 30- 39 Yrs
0 30- 39 Yrs
0 30- 39 Yrs
0 30- 39 Yrs
0 30- 39 Yrs
0 30- 39 Yrs
0 30- 39 Yrs
0 30- 39 Yrs
0 30- 39 Yrs
0 30- 39 Yrs
0 30- 39 Yrs
0 30- 39 Yrs
0 30- 39 Yrs
0 30- 39 Yrs
0 30- 39 Yrs
0 30- 39 Yrs
0 30- 39 Yrs
0 30- 39 Yrs
0 30- 39 Yrs
0 30- 39 Yrs

0 30- 39 Yrs
0 30- 39 Yrs
0 30- 39 Yrs
0 30- 39 Yrs
0 30- 39 Yrs
0 30- 39 Yrs
0 30- 39 Yrs
0 30- 39 Yrs
0 30- 39 Yrs
0 30- 39 Yrs
0 30- 39 Yrs
0 30- 39 Yrs
0 30- 39 Yrs
0 30- 39 Yrs
0 30- 39 Yrs
0 30- 39 Yrs
0 30- 39 Yrs
0 30- 39 Yrs
0 30- 39 Yrs
0 30- 39 Yrs
0 < 10 Yrs
0 < 10 Yrs
0 < 10 Yrs
0 < 10 Yrs
0 < 10 Yrs
0 < 10 Yrs
0 < 10 Yrs
0 < 10 Yrs
0 < 10 Yrs
0 < 10 Yrs
0 < 10 Yrs
0 < 10 Yrs
0 < 10 Yrs
0 < 10 Yrs
0 < 10 Yrs
0 < 10 Yrs
0 < 10 Yrs
0 < 10 Yrs
0 < 10 Yrs
0 < 10 Yrs
0 < 10 Yrs
0 < 10 Yrs
0 < 10 Yrs
0 < 10 Yrs
0 < 10 Yrs
0 < 10 Yrs
0 < 10 Yrs
0 < 10 Yrs
0 < 10 Yrs
0 < 10 Yrs

0 < 10 Yrs
0 < 10 Yrs
0 < 10 Yrs
0 < 10 Yrs
0 < 10 Yrs
0 < 10 Yrs
0 < 10 Yrs
0 < 10 Yrs
0 < 10 Yrs
0 < 10 Yrs
0 < 10 Yrs
0 < 10 Yrs
0 < 10 Yrs
0 < 10 Yrs
0 < 10 Yrs
0 < 10 Yrs
0 < 10 Yrs
0 < 10 Yrs
0 < 10 Yrs
0 < 10 Yrs
0 < 10 Yrs
0 < 10 Yrs
0 < 10 Yrs
0 < 10 Yrs
0 < 10 Yrs
0 < 10 Yrs
0 < 10 Yrs
0 < 10 Yrs
0 < 10 Yrs
0 < 10 Yrs
0 < 10 Yrs
0 < 10 Yrs
0 < 10 Yrs
0 < 10 Yrs
0 < 10 Yrs
0 < 10 Yrs
0 < 10 Yrs
0 < 10 Yrs
0 < 10 Yrs
0 < 10 Yrs
0 < 10 Yrs
0 < 10 Yrs
0 < 10 Yrs
0 < 10 Yrs
0 < 10 Yrs
0 < 10 Yrs
0 < 10 Yrs
0 < 10 Yrs
0 < 10 Yrs
0 < 10 Yrs

0	< 10 Yrs
0	< 10 Yrs
0	< 10 Yrs
0	< 10 Yrs
0	< 10 Yrs
0	< 10 Yrs
0	< 10 Yrs
0	< 10 Yrs
0	< 10 Yrs
0	< 10 Yrs
0	< 10 Yrs
0	< 10 Yrs
0	< 10 Yrs
0	< 10 Yrs
0	< 10 Yrs
0	< 10 Yrs
0	< 10 Yrs
0	< 10 Yrs
0	< 10 Yrs
0	< 10 Yrs
0	< 10 Yrs
0	< 10 Yrs
0	< 10 Yrs
0	< 10 Yrs
0	< 10 Yrs
0	10- 19 Yrs
0	10- 19 Yrs
0	10- 19 Yrs
0	10- 19 Yrs
0	10- 19 Yrs
0	10- 19 Yrs
0	10- 19 Yrs
0	10- 19 Yrs
0	10- 19 Yrs
0	10- 19 Yrs
0	10- 19 Yrs
0	10- 19 Yrs
0	10- 19 Yrs
0	10- 19 Yrs
0	10- 19 Yrs
0	10- 19 Yrs
0	10- 19 Yrs
0	10- 19 Yrs
0	10- 19 Yrs
0	10- 19 Yrs
0	10- 19 Yrs
0	10- 19 Yrs
0	10- 19 Yrs
0	10- 19 Yrs
0	10- 19 Yrs

0 10- 19 Yrs
0 10- 19 Yrs
0 10- 19 Yrs
0 10- 19 Yrs
0 10- 19 Yrs
0 10- 19 Yrs
0 10- 19 Yrs
0 10- 19 Yrs
0 10- 19 Yrs
0 10- 19 Yrs
0 10- 19 Yrs
0 10- 19 Yrs
0 10- 19 Yrs
0 10- 19 Yrs
0 10- 19 Yrs
0 10- 19 Yrs
0 10- 19 Yrs
0 10- 19 Yrs
0 10- 19 Yrs
0 10- 19 Yrs
0 10- 19 Yrs
0 10- 19 Yrs
0 10- 19 Yrs
0 10- 19 Yrs
0 10- 19 Yrs
0 10- 19 Yrs
0 10- 19 Yrs
0 10- 19 Yrs
0 10- 19 Yrs
0 10- 19 Yrs
0 10- 19 Yrs
0 10- 19 Yrs
0 10- 19 Yrs
0 10- 19 Yrs
0 10- 19 Yrs
0 10- 19 Yrs
0 10- 19 Yrs
0 10- 19 Yrs
0 10- 19 Yrs
0 10- 19 Yrs
0 10- 19 Yrs
0 10- 19 Yrs
0 10- 19 Yrs
0 10- 19 Yrs
0 10- 19 Yrs
0 10- 19 Yrs
0 10- 19 Yrs
0 10- 19 Yrs
0 10- 19 Yrs
0 10- 19 Yrs

0	10- 19 Yrs
0	10- 19 Yrs
0	10- 19 Yrs
0	10- 19 Yrs
0	10- 19 Yrs
0	10- 19 Yrs
0	10- 19 Yrs
0	10- 19 Yrs
0	10- 19 Yrs
0	10- 19 Yrs
0	10- 19 Yrs
0	10- 19 Yrs
0	10- 19 Yrs
0	10- 19 Yrs
0	10- 19 Yrs
0	10- 19 Yrs
0	10- 19 Yrs
0	10- 19 Yrs
0	10- 19 Yrs
0	10- 19 Yrs
0	10- 19 Yrs
0	10- 19 Yrs
0	10- 19 Yrs
0	10- 19 Yrs
0	10- 19 Yrs
0	10- 19 Yrs
0	10- 19 Yrs
0	10- 19 Yrs
0	10- 19 Yrs
0	10- 19 Yrs
0	10- 19 Yrs
0	10- 19 Yrs
0	10- 19 Yrs
0	10- 19 Yrs
0	10- 19 Yrs
0	10- 19 Yrs
0	10- 19 Yrs
0	10- 19 Yrs
0	10- 19 Yrs
0	20- 29 Yrs
0	20- 29 Yrs
0	20- 29 Yrs
0	20- 29 Yrs
0	20- 29 Yrs
0	20- 29 Yrs
0	20- 29 Yrs
0	20- 29 Yrs
0	20- 29 Yrs
0	20- 29 Yrs
0	20- 29 Yrs

0 20- 29 Yrs
0 20- 29 Yrs
0 20- 29 Yrs
0 20- 29 Yrs
0 20- 29 Yrs
0 20- 29 Yrs
0 20- 29 Yrs
0 20- 29 Yrs
0 20- 29 Yrs
0 20- 29 Yrs
0 20- 29 Yrs
0 20- 29 Yrs
0 20- 29 Yrs
0 20- 29 Yrs
0 20- 29 Yrs
0 20- 29 Yrs
0 20- 29 Yrs
0 20- 29 Yrs
0 20- 29 Yrs
0 20- 29 Yrs
0 20- 29 Yrs
0 20- 29 Yrs
0 20- 29 Yrs
0 20- 29 Yrs
0 20- 29 Yrs
0 20- 29 Yrs
0 20- 29 Yrs
0 20- 29 Yrs
0 20- 29 Yrs
0 20- 29 Yrs
0 20- 29 Yrs
0 20- 29 Yrs
0 20- 29 Yrs
0 20- 29 Yrs
0 20- 29 Yrs
0 20- 29 Yrs
0 20- 29 Yrs
0 20- 29 Yrs
0 20- 29 Yrs
0 20- 29 Yrs
0 20- 29 Yrs
0 20- 29 Yrs
0 20- 29 Yrs
0 20- 29 Yrs
0 20- 29 Yrs
0 20- 29 Yrs
0 20- 29 Yrs
0 20- 29 Yrs
0 20- 29 Yrs
0 20- 29 Yrs

0 20- 29 Yrs
0 20- 29 Yrs
0 20- 29 Yrs
0 20- 29 Yrs
0 20- 29 Yrs
0 20- 29 Yrs
0 20- 29 Yrs
0 20- 29 Yrs
0 20- 29 Yrs
0 20- 29 Yrs
0 20- 29 Yrs
0 20- 29 Yrs
0 20- 29 Yrs
0 20- 29 Yrs
0 20- 29 Yrs
0 20- 29 Yrs
0 20- 29 Yrs
0 20- 29 Yrs
0 20- 29 Yrs
0 20- 29 Yrs
0 20- 29 Yrs
0 20- 29 Yrs
0 20- 29 Yrs
0 20- 29 Yrs
0 20- 29 Yrs
0 20- 29 Yrs
0 20- 29 Yrs
0 20- 29 Yrs
0 20- 29 Yrs
0 20- 29 Yrs
0 20- 29 Yrs
0 20- 29 Yrs
0 20- 29 Yrs
0 20- 29 Yrs
0 20- 29 Yrs
0 20- 29 Yrs
0 20- 29 Yrs
0 20- 29 Yrs
0 20- 29 Yrs
0 20- 29 Yrs
0 20- 29 Yrs
0 20- 29 Yrs
0 20- 29 Yrs
0 20- 29 Yrs
0 20- 29 Yrs
0 20- 29 Yrs
0 20- 29 Yrs
0 20- 29 Yrs
0 20- 29 Yrs
0 20- 29 Yrs

0 20- 29 Yrs
0 20- 29 Yrs
0 20- 29 Yrs
0 20- 29 Yrs
0 20- 29 Yrs
0 20- 29 Yrs
0 70+ Yrs
0 70+ Yrs
0 70+ Yrs
0 70+ Yrs
0 70+ Yrs
0 70+ Yrs
0 70+ Yrs
0 70+ Yrs
0 70+ Yrs
0 70+ Yrs
0 70+ Yrs
0 70+ Yrs
0 70+ Yrs
0 70+ Yrs
0 70+ Yrs
0 70+ Yrs
0 70+ Yrs
0 70+ Yrs
0 70+ Yrs
0 70+ Yrs
0 70+ Yrs
0 70+ Yrs
0 70+ Yrs
0 70+ Yrs
0 70+ Yrs
0 70+ Yrs
0 70+ Yrs
0 70+ Yrs
0 70+ Yrs
0 70+ Yrs
0 70+ Yrs
0 70+ Yrs
0 70+ Yrs
0 70+ Yrs
0 70+ Yrs
0 70+ Yrs
0 70+ Yrs
0 70+ Yrs
0 70+ Yrs
0 70+ Yrs
0 70+ Yrs
0 70+ Yrs
0 70+ Yrs
0 70+ Yrs

0	70+ Yrs
0	70+ Yrs
0	70+ Yrs
0	70+ Yrs
0	70+ Yrs
0	70+ Yrs
0	70+ Yrs
0	70+ Yrs
0	70+ Yrs
0	70+ Yrs
0	70+ Yrs
0	70+ Yrs
0	70+ Yrs
0	70+ Yrs
0	70+ Yrs
0	70+ Yrs
0	70+ Yrs
0	70+ Yrs
0	70+ Yrs
0	70+ Yrs
0	70+ Yrs
0	70+ Yrs
0	70+ Yrs
0	70+ Yrs
0	70+ Yrs
0	70+ Yrs
0	70+ Yrs
0	70+ Yrs
0	70+ Yrs
0	70+ Yrs
0	70+ Yrs
0	70+ Yrs
0	70+ Yrs
0	70+ Yrs
0	70+ Yrs
0	70+ Yrs
0	70+ Yrs
0	70+ Yrs
0	70+ Yrs
0	70+ Yrs
0	70+ Yrs
0	70+ Yrs
0	70+ Yrs
0	70+ Yrs
0	70+ Yrs
0	70+ Yrs
0	70+ Yrs
0	70+ Yrs
0	70+ Yrs
0	70+ Yrs

0 70+ Yrs
0 70+ Yrs
0 70+ Yrs
0 70+ Yrs
0 70+ Yrs
0 70+ Yrs
0 50- 59 Yrs
0 50- 59 Yrs
0 50- 59 Yrs
0 50- 59 Yrs
0 50- 59 Yrs
0 50- 59 Yrs
0 50- 59 Yrs
0 50- 59 Yrs
0 50- 59 Yrs
0 50- 59 Yrs
0 50- 59 Yrs
0 50- 59 Yrs
0 50- 59 Yrs
0 50- 59 Yrs
0 50- 59 Yrs
0 50- 59 Yrs
0 50- 59 Yrs
0 50- 59 Yrs
0 50- 59 Yrs
0 50- 59 Yrs
0 50- 59 Yrs
0 50- 59 Yrs
0 50- 59 Yrs
0 50- 59 Yrs
0 50- 59 Yrs
0 50- 59 Yrs
0 50- 59 Yrs
0 50- 59 Yrs
0 50- 59 Yrs
0 50- 59 Yrs
0 50- 59 Yrs
0 50- 59 Yrs
0 50- 59 Yrs
0 50- 59 Yrs
0 50- 59 Yrs
0 50- 59 Yrs
0 50- 59 Yrs
0 50- 59 Yrs
0 50- 59 Yrs
0 50- 59 Yrs
0 50- 59 Yrs
0 50- 59 Yrs
0 50- 59 Yrs
0 50- 59 Yrs

0 50- 59 Yrs
0 50- 59 Yrs
0 50- 59 Yrs
0 50- 59 Yrs
0 50- 59 Yrs
0 50- 59 Yrs
0 50- 59 Yrs
0 50- 59 Yrs
0 50- 59 Yrs
0 50- 59 Yrs
0 50- 59 Yrs
0 50- 59 Yrs
0 50- 59 Yrs
0 50- 59 Yrs
0 50- 59 Yrs
0 50- 59 Yrs
0 50- 59 Yrs
0 50- 59 Yrs
0 50- 59 Yrs
0 50- 59 Yrs
0 50- 59 Yrs
0 50- 59 Yrs
0 50- 59 Yrs
0 50- 59 Yrs
0 50- 59 Yrs
0 50- 59 Yrs
0 50- 59 Yrs
0 50- 59 Yrs
0 50- 59 Yrs
0 50- 59 Yrs
0 50- 59 Yrs
0 50- 59 Yrs
0 50- 59 Yrs
0 50- 59 Yrs
0 50- 59 Yrs
0 50- 59 Yrs
0 50- 59 Yrs
0 50- 59 Yrs
0 60- 69 Yrs
0 60- 69 Yrs
0 60- 69 Yrs
0 60- 69 Yrs
0 60- 69 Yrs
0 60- 69 Yrs
0 60- 69 Yrs
0 60- 69 Yrs
0 60- 69 Yrs
0 60- 69 Yrs
0 60- 69 Yrs
0 60- 69 Yrs

0	60- 69 Yrs
0	60- 69 Yrs
0	60- 69 Yrs
0	60- 69 Yrs
0	60- 69 Yrs
0	60- 69 Yrs
0	60- 69 Yrs
0	60- 69 Yrs
0	60- 69 Yrs
0	60- 69 Yrs
0	60- 69 Yrs
0	60- 69 Yrs
0	60- 69 Yrs
0	60- 69 Yrs
0	60- 69 Yrs
0	60- 69 Yrs
0	60- 69 Yrs
0	60- 69 Yrs
0	60- 69 Yrs
0	60- 69 Yrs
0	60- 69 Yrs
0	60- 69 Yrs
0	60- 69 Yrs
0	60- 69 Yrs
0	60- 69 Yrs
0	60- 69 Yrs
0	60- 69 Yrs
0	60- 69 Yrs
0	60- 69 Yrs
0	60- 69 Yrs
0	60- 69 Yrs
0	60- 69 Yrs
0	60- 69 Yrs
0	60- 69 Yrs
0	60- 69 Yrs
0	60- 69 Yrs
0	60- 69 Yrs
0	60- 69 Yrs
0	60- 69 Yrs
0	60- 69 Yrs
0	60- 69 Yrs
0	60- 69 Yrs
0	60- 69 Yrs
0	60- 69 Yrs
0	60- 69 Yrs
0	60- 69 Yrs
0	60- 69 Yrs
0	60- 69 Yrs
0	60- 69 Yrs
0	60- 69 Yrs

0 60- 69 Yrs
0 60- 69 Yrs
0 60- 69 Yrs
0 60- 69 Yrs
0 60- 69 Yrs
0 60- 69 Yrs
0 60- 69 Yrs
0 60- 69 Yrs
0 60- 69 Yrs
0 40- 49 Yrs
0 40- 49 Yrs
0 40- 49 Yrs
0 40- 49 Yrs
0 40- 49 Yrs
0 40- 49 Yrs
0 40- 49 Yrs
0 40- 49 Yrs
0 40- 49 Yrs
0 40- 49 Yrs
0 40- 49 Yrs
0 40- 49 Yrs
0 40- 49 Yrs
0 40- 49 Yrs
0 40- 49 Yrs
0 40- 49 Yrs
0 40- 49 Yrs
0 40- 49 Yrs
0 40- 49 Yrs
0 40- 49 Yrs
0 40- 49 Yrs
0 40- 49 Yrs
0 40- 49 Yrs
0 40- 49 Yrs
0 40- 49 Yrs
0 40- 49 Yrs
0 40- 49 Yrs
0 40- 49 Yrs
0 40- 49 Yrs
0 40- 49 Yrs
0 40- 49 Yrs
0 40- 49 Yrs
0 40- 49 Yrs
0 40- 49 Yrs
0 40- 49 Yrs
0 40- 49 Yrs
0 40- 49 Yrs
0 40- 49 Yrs
0 40- 49 Yrs
0 40- 49 Yrs
0 40- 49 Yrs

0 40- 49 Yrs
0 40- 49 Yrs
0 40- 49 Yrs
0 40- 49 Yrs
0 40- 49 Yrs
0 40- 49 Yrs
0 40- 49 Yrs
0 40- 49 Yrs
0 40- 49 Yrs
0 40- 49 Yrs
0 40- 49 Yrs
0 40- 49 Yrs
0 40- 49 Yrs
0 40- 49 Yrs
0 40- 49 Yrs
0 40- 49 Yrs
0 40- 49 Yrs
0 40- 49 Yrs
0 40- 49 Yrs
0 40- 49 Yrs
0 40- 49 Yrs
0 40- 49 Yrs
0 40- 49 Yrs
0 40- 49 Yrs
0 40- 49 Yrs
0 40- 49 Yrs
0 40- 49 Yrs
0 40- 49 Yrs
0 40- 49 Yrs
0 40- 49 Yrs
0 10- 19 Yrs
0 10- 19 Yrs
0 10- 19 Yrs
0 10- 19 Yrs
0 10- 19 Yrs
0 10- 19 Yrs
0 10- 19 Yrs
0 10- 19 Yrs
0 10- 19 Yrs
0 10- 19 Yrs
0 10- 19 Yrs
0 70+ Yrs
0 70+ Yrs
0 70+ Yrs
0 70+ Yrs
0 70+ Yrs
0 70+ Yrs
0 70+ Yrs
0 70+ Yrs
0 70+ Yrs

0	70+ Yrs
0	70+ Yrs
0	70+ Yrs
0	70+ Yrs
0	70+ Yrs
0	70+ Yrs
0	70+ Yrs
0	70+ Yrs
0	70+ Yrs
0	70+ Yrs
0	70+ Yrs
0	70+ Yrs
0	70+ Yrs
0	70+ Yrs
0	70+ Yrs
0	70+ Yrs
0	70+ Yrs
0	70+ Yrs
0	70+ Yrs
0	70+ Yrs
0	70+ Yrs
0	70+ Yrs
0	70+ Yrs
0	70+ Yrs
0	70+ Yrs
0	70+ Yrs
0	70+ Yrs
0	70+ Yrs
0	70+ Yrs
0	70+ Yrs
0	70+ Yrs
0	70+ Yrs
0	70+ Yrs
0	70+ Yrs
0	70+ Yrs
0	70+ Yrs
0	70+ Yrs
0	70+ Yrs
0	70+ Yrs
0	70+ Yrs
0	70+ Yrs
0	70+ Yrs
0	70+ Yrs
0	70+ Yrs
0	70+ Yrs
0	70+ Yrs
0	70+ Yrs
0	70+ Yrs
0	70+ Yrs
0	70+ Yrs

0	70+ Yrs
0	70+ Yrs
0	70+ Yrs
0	70+ Yrs
0	70+ Yrs
0	70+ Yrs
0	70+ Yrs
0	70+ Yrs
0	70+ Yrs
0	70+ Yrs
0	70+ Yrs
0	70+ Yrs
0	70+ Yrs
0	70+ Yrs
0	70+ Yrs
0	70+ Yrs
0	70+ Yrs
0	70+ Yrs
0	70+ Yrs
0	70+ Yrs
0	20- 29 Yrs
0	20- 29 Yrs
0	20- 29 Yrs
0	20- 29 Yrs
0	20- 29 Yrs
0	20- 29 Yrs
0	20- 29 Yrs
0	20- 29 Yrs
0	20- 29 Yrs
0	20- 29 Yrs
0	20- 29 Yrs
0	20- 29 Yrs
0	20- 29 Yrs
0	20- 29 Yrs
0	20- 29 Yrs
0	20- 29 Yrs
0	20- 29 Yrs
0	20- 29 Yrs
0	20- 29 Yrs
0	20- 29 Yrs
0	20- 29 Yrs
0	20- 29 Yrs
0	20- 29 Yrs
0	20- 29 Yrs
0	20- 29 Yrs
0	20- 29 Yrs
0	20- 29 Yrs
0	20- 29 Yrs
0	20- 29 Yrs
0	20- 29 Yrs

0	20- 29 Yrs
0	20- 29 Yrs
0	20- 29 Yrs
0	20- 29 Yrs
0	20- 29 Yrs
0	20- 29 Yrs
0	20- 29 Yrs
0	20- 29 Yrs
0	20- 29 Yrs
0	20- 29 Yrs
0	20- 29 Yrs
0	20- 29 Yrs
0	20- 29 Yrs
0	20- 29 Yrs
0	20- 29 Yrs
0	20- 29 Yrs
0	20- 29 Yrs
0	20- 29 Yrs
0	20- 29 Yrs
0	20- 29 Yrs
0	20- 29 Yrs
0	20- 29 Yrs
0	20- 29 Yrs
0	20- 29 Yrs
0	20- 29 Yrs
0	20- 29 Yrs
0	20- 29 Yrs
0	20- 29 Yrs
0	20- 29 Yrs
0	20- 29 Yrs
0	20- 29 Yrs
0	20- 29 Yrs
0	20- 29 Yrs
0	20- 29 Yrs
0	20- 29 Yrs
0	20- 29 Yrs
0	20- 29 Yrs
0	20- 29 Yrs
0	20- 29 Yrs
0	20- 29 Yrs
0	20- 29 Yrs
0	20- 29 Yrs
0	20- 29 Yrs
0	20- 29 Yrs
0	20- 29 Yrs
0	20- 29 Yrs
0	20- 29 Yrs
0	20- 29 Yrs
0	20- 29 Yrs
0	20- 29 Yrs

0	20- 29 Yrs
0	20- 29 Yrs
0	20- 29 Yrs
0	20- 29 Yrs
0	20- 29 Yrs
0	20- 29 Yrs
0	20- 29 Yrs
0	20- 29 Yrs
0	20- 29 Yrs
0	20- 29 Yrs
0	20- 29 Yrs
0	20- 29 Yrs
0	60- 69 Yrs
0	60- 69 Yrs
0	60- 69 Yrs
0	60- 69 Yrs
0	60- 69 Yrs
0	60- 69 Yrs
0	60- 69 Yrs
0	60- 69 Yrs
0	60- 69 Yrs
0	60- 69 Yrs
0	60- 69 Yrs
0	60- 69 Yrs
0	60- 69 Yrs
0	60- 69 Yrs
0	60- 69 Yrs
0	60- 69 Yrs
0	60- 69 Yrs
0	60- 69 Yrs
0	60- 69 Yrs
0	60- 69 Yrs
0	60- 69 Yrs
0	60- 69 Yrs
0	60- 69 Yrs
0	60- 69 Yrs
0	60- 69 Yrs
0	60- 69 Yrs
0	60- 69 Yrs
0	60- 69 Yrs
0	60- 69 Yrs
0	60- 69 Yrs
0	60- 69 Yrs
0	60- 69 Yrs
0	60- 69 Yrs
0	60- 69 Yrs
0	60- 69 Yrs
0	60- 69 Yrs
0	60- 69 Yrs
0	60- 69 Yrs

0 60- 69 Yrs
0 60- 69 Yrs
0 60- 69 Yrs
0 60- 69 Yrs
0 60- 69 Yrs
0 60- 69 Yrs
0 60- 69 Yrs
0 60- 69 Yrs
0 60- 69 Yrs
0 60- 69 Yrs
0 60- 69 Yrs
0 60- 69 Yrs
0 60- 69 Yrs
0 60- 69 Yrs
0 60- 69 Yrs
0 60- 69 Yrs
0 60- 69 Yrs
0 60- 69 Yrs
0 60- 69 Yrs
0 60- 69 Yrs
0 60- 69 Yrs
0 60- 69 Yrs
0 60- 69 Yrs
0 60- 69 Yrs
0 60- 69 Yrs
0 60- 69 Yrs
0 60- 69 Yrs
0 60- 69 Yrs
0 60- 69 Yrs
0 60- 69 Yrs
0 60- 69 Yrs
0 60- 69 Yrs
0 60- 69 Yrs
0 60- 69 Yrs
0 60- 69 Yrs
0 60- 69 Yrs
0 60- 69 Yrs
0 60- 69 Yrs
0 60- 69 Yrs
0 60- 69 Yrs
0 60- 69 Yrs
0 60- 69 Yrs
0 60- 69 Yrs
0 60- 69 Yrs
0 60- 69 Yrs
0 60- 69 Yrs
0 60- 69 Yrs
0 60- 69 Yrs
0 60- 69 Yrs
0 60- 69 Yrs

0 60- 69 Yrs
0 60- 69 Yrs
0 60- 69 Yrs
0 60- 69 Yrs
0 60- 69 Yrs
0 60- 69 Yrs
0 60- 69 Yrs
0 60- 69 Yrs
0 60- 69 Yrs
0 60- 69 Yrs
0 60- 69 Yrs
0 60- 69 Yrs
0 60- 69 Yrs
0 60- 69 Yrs
0 60- 69 Yrs
0 60- 69 Yrs
0 60- 69 Yrs
0 60- 69 Yrs
0 60- 69 Yrs
0 60- 69 Yrs
0 60- 69 Yrs
0 60- 69 Yrs
0 50- 59 Yrs
0 50- 59 Yrs
0 50- 59 Yrs
0 50- 59 Yrs
0 50- 59 Yrs
0 50- 59 Yrs
0 50- 59 Yrs
0 50- 59 Yrs
0 50- 59 Yrs
0 50- 59 Yrs
0 50- 59 Yrs
0 50- 59 Yrs
0 50- 59 Yrs
0 50- 59 Yrs
0 50- 59 Yrs
0 50- 59 Yrs
0 50- 59 Yrs
0 50- 59 Yrs
0 50- 59 Yrs
0 50- 59 Yrs
0 50- 59 Yrs
0 50- 59 Yrs
0 50- 59 Yrs
0 50- 59 Yrs
0 50- 59 Yrs
0 50- 59 Yrs
0 50- 59 Yrs
0 50- 59 Yrs

0 50- 59 Yrs
0 50- 59 Yrs
0 50- 59 Yrs
0 50- 59 Yrs
0 50- 59 Yrs
0 50- 59 Yrs
0 50- 59 Yrs
0 50- 59 Yrs
0 50- 59 Yrs
0 50- 59 Yrs
0 50- 59 Yrs
0 50- 59 Yrs
0 50- 59 Yrs
0 50- 59 Yrs
0 50- 59 Yrs
0 50- 59 Yrs
0 50- 59 Yrs
0 50- 59 Yrs
0 50- 59 Yrs
0 50- 59 Yrs
0 50- 59 Yrs
0 50- 59 Yrs
0 50- 59 Yrs
0 50- 59 Yrs
0 50- 59 Yrs
0 50- 59 Yrs
0 50- 59 Yrs
0 50- 59 Yrs
0 50- 59 Yrs
0 50- 59 Yrs
0 50- 59 Yrs
0 50- 59 Yrs
0 50- 59 Yrs
0 50- 59 Yrs
0 50- 59 Yrs
0 50- 59 Yrs
0 50- 59 Yrs
0 50- 59 Yrs
0 50- 59 Yrs
0 50- 59 Yrs
0 50- 59 Yrs
0 50- 59 Yrs
0 50- 59 Yrs
0 50- 59 Yrs
0 50- 59 Yrs
0 50- 59 Yrs
0 50- 59 Yrs
0 50- 59 Yrs
0 50- 59 Yrs
0 50- 59 Yrs

0 50- 59 Yrs
0 50- 59 Yrs
0 50- 59 Yrs
0 50- 59 Yrs
0 50- 59 Yrs
0 50- 59 Yrs
0 50- 59 Yrs
0 50- 59 Yrs
0 50- 59 Yrs
0 50- 59 Yrs
0 50- 59 Yrs
0 50- 59 Yrs
0 50- 59 Yrs
0 50- 59 Yrs
0 50- 59 Yrs
0 50- 59 Yrs
0 50- 59 Yrs
0 50- 59 Yrs
0 50- 59 Yrs
0 50- 59 Yrs
0 50- 59 Yrs
0 50- 59 Yrs
0 50- 59 Yrs
0 50- 59 Yrs
0 50- 59 Yrs
0 30- 39 Yrs
0 30- 39 Yrs
0 30- 39 Yrs
0 30- 39 Yrs
0 30- 39 Yrs
0 30- 39 Yrs
0 30- 39 Yrs
0 30- 39 Yrs
0 30- 39 Yrs
0 30- 39 Yrs
0 30- 39 Yrs
0 30- 39 Yrs
0 30- 39 Yrs
0 30- 39 Yrs
0 30- 39 Yrs
0 30- 39 Yrs
0 30- 39 Yrs
0 30- 39 Yrs
0 30- 39 Yrs
0 30- 39 Yrs
0 30- 39 Yrs
0 30- 39 Yrs
0 30- 39 Yrs
0 30- 39 Yrs
0 30- 39 Yrs

0	30- 39 Yrs
0	30- 39 Yrs
0	30- 39 Yrs
0	30- 39 Yrs
0	30- 39 Yrs
0	30- 39 Yrs
0	30- 39 Yrs
0	30- 39 Yrs
0	30- 39 Yrs
0	30- 39 Yrs
0	30- 39 Yrs
0	30- 39 Yrs
0	30- 39 Yrs
0	30- 39 Yrs
0	30- 39 Yrs
0	30- 39 Yrs
0	30- 39 Yrs
0	30- 39 Yrs
0	30- 39 Yrs
0	30- 39 Yrs
0	30- 39 Yrs
0	30- 39 Yrs
0	30- 39 Yrs
0	30- 39 Yrs
0	30- 39 Yrs
0	30- 39 Yrs
0	30- 39 Yrs
0	30- 39 Yrs
0	30- 39 Yrs
0	30- 39 Yrs
0	30- 39 Yrs
0	30- 39 Yrs
0	30- 39 Yrs
0	30- 39 Yrs
0	30- 39 Yrs
0	30- 39 Yrs
0	30- 39 Yrs
0	30- 39 Yrs
0	30- 39 Yrs
0	30- 39 Yrs
0	30- 39 Yrs
0	30- 39 Yrs
0	30- 39 Yrs
0	30- 39 Yrs
0	30- 39 Yrs
0	30- 39 Yrs
0	30- 39 Yrs
0	30- 39 Yrs
0	30- 39 Yrs
0	30- 39 Yrs

0 30- 39 Yrs
0 30- 39 Yrs
0 30- 39 Yrs
0 30- 39 Yrs
0 30- 39 Yrs
0 30- 39 Yrs
0 30- 39 Yrs
0 30- 39 Yrs
0 30- 39 Yrs
0 30- 39 Yrs
0 30- 39 Yrs
0 30- 39 Yrs
0 30- 39 Yrs
0 30- 39 Yrs
0 30- 39 Yrs
0 30- 39 Yrs
0 30- 39 Yrs
0 30- 39 Yrs
0 30- 39 Yrs
0 30- 39 Yrs
0 30- 39 Yrs
0 30- 39 Yrs
0 30- 39 Yrs
0 40- 49 Yrs
0 40- 49 Yrs
0 40- 49 Yrs
0 40- 49 Yrs
0 40- 49 Yrs
0 40- 49 Yrs
0 40- 49 Yrs
0 40- 49 Yrs
0 40- 49 Yrs
0 40- 49 Yrs
0 40- 49 Yrs
0 40- 49 Yrs
0 40- 49 Yrs
0 40- 49 Yrs
0 40- 49 Yrs
0 40- 49 Yrs
0 40- 49 Yrs
0 40- 49 Yrs
0 40- 49 Yrs
0 40- 49 Yrs
0 40- 49 Yrs
0 40- 49 Yrs
0 40- 49 Yrs
0 40- 49 Yrs
0 40- 49 Yrs
0 40- 49 Yrs
0 40- 49 Yrs

0 40- 49 Yrs
0 40- 49 Yrs
0 40- 49 Yrs
0 40- 49 Yrs
0 40- 49 Yrs
0 40- 49 Yrs
0 40- 49 Yrs
0 40- 49 Yrs
0 40- 49 Yrs
0 40- 49 Yrs
0 40- 49 Yrs
0 40- 49 Yrs
0 40- 49 Yrs
0 40- 49 Yrs
0 40- 49 Yrs
0 40- 49 Yrs
0 40- 49 Yrs
0 40- 49 Yrs
0 40- 49 Yrs
0 40- 49 Yrs
0 40- 49 Yrs
0 40- 49 Yrs
0 40- 49 Yrs
0 40- 49 Yrs
0 40- 49 Yrs
0 40- 49 Yrs
0 40- 49 Yrs
0 40- 49 Yrs
0 40- 49 Yrs
0 40- 49 Yrs
0 40- 49 Yrs
0 40- 49 Yrs
0 40- 49 Yrs
0 40- 49 Yrs
0 40- 49 Yrs
0 40- 49 Yrs
0 40- 49 Yrs
0 40- 49 Yrs
0 40- 49 Yrs
0 40- 49 Yrs
0 40- 49 Yrs
0 40- 49 Yrs
0 40- 49 Yrs
0 40- 49 Yrs
0 40- 49 Yrs
0 40- 49 Yrs
0 40- 49 Yrs
0 40- 49 Yrs
0 40- 49 Yrs
0 40- 49 Yrs

0 40- 49 Yrs
0 40- 49 Yrs
0 40- 49 Yrs
0 40- 49 Yrs
0 40- 49 Yrs
0 40- 49 Yrs
0 40- 49 Yrs
0 40- 49 Yrs
0 40- 49 Yrs
0 40- 49 Yrs
0 40- 49 Yrs
0 40- 49 Yrs
0 40- 49 Yrs
0 40- 49 Yrs
0 40- 49 Yrs
0 40- 49 Yrs
0 40- 49 Yrs
0 40- 49 Yrs
0 40- 49 Yrs
0 40- 49 Yrs
0 40- 49 Yrs
0 40- 49 Yrs
0 40- 49 Yrs
0 40- 49 Yrs
0 40- 49 Yrs
0 40- 49 Yrs
0 40- 49 Yrs
0 40- 49 Yrs
0 40- 49 Yrs
0 40- 49 Yrs
0 40- 49 Yrs
0 40- 49 Yrs
0 40- 49 Yrs
0 40- 49 Yrs
0 40- 49 Yrs
0 40- 49 Yrs
0 40- 49 Yrs
0 40- 49 Yrs
0 40- 49 Yrs
0 40- 49 Yrs
0 40- 49 Yrs
0 40- 49 Yrs
0 40- 49 Yrs
0 40- 49 Yrs
0 40- 49 Yrs
0 40- 49 Yrs
0 40- 49 Yrs
0 40- 49 Yrs
0 40- 49 Yrs
0 40- 49 Yrs

0 40- 49 Yrs
0 40- 49 Yrs
0 40- 49 Yrs
0 40- 49 Yrs
0 40- 49 Yrs
0 40- 49 Yrs
0 40- 49 Yrs
0 40- 49 Yrs
0 40- 49 Yrs
0 40- 49 Yrs
0 40- 49 Yrs
0 40- 49 Yrs
0 40- 49 Yrs
0 40- 49 Yrs
0 40- 49 Yrs
0 40- 49 Yrs
0 40- 49 Yrs
0 40- 49 Yrs
0 40- 49 Yrs
0 40- 49 Yrs
0 30- 39 Yrs
0 40- 49 Yrs
0 30- 39 Yrs
0 10- 19 Yrs
0 20- 29 Yrs
0 10- 19 Yrs
0 20- 29 Yrs
0 40- 49 Yrs
0 60- 69 Yrs
0 70+ Yrs
0 70+ Yrs
0 < 10 Yrs
0 50- 59 Yrs
0 50- 59 Yrs
0 60- 69 Yrs
0 < 10 Yrs
0 50- 59 Yrs
0 60- 69 Yrs
0 70+ Yrs
0 50- 59 Yrs
0 70+ Yrs
0 60- 69 Yrs
0 40- 49 Yrs
0 70+ Yrs
0 60- 69 Yrs
0 50- 59 Yrs
0 70+ Yrs
0 40- 49 Yrs
0 40- 49 Yrs
0 70+ Yrs

0	60- 69 Yrs
0	50- 59 Yrs
0	< 10 Yrs
0	60- 69 Yrs
0	50- 59 Yrs
0	60- 69 Yrs
0	< 10 Yrs
0	70+ Yrs
0	70+ Yrs
0	30- 39 Yrs
0	40- 49 Yrs
0	20- 29 Yrs
0	40- 49 Yrs
0	30- 39 Yrs
0	10- 19 Yrs
0	20- 29 Yrs
0	10- 19 Yrs
0	50- 59 Yrs
0	50- 59 Yrs
0	70+ Yrs
0	60- 69 Yrs
0	< 10 Yrs
0	70+ Yrs
0	< 10 Yrs
0	30- 39 Yrs
0	10- 19 Yrs
0	50- 59 Yrs
0	10- 19 Yrs
0	50- 59 Yrs
0	30- 39 Yrs
0	20- 29 Yrs
0	40- 49 Yrs
0	40- 49 Yrs
0	40- 49 Yrs
0	20- 29 Yrs
0	60- 69 Yrs
0	70+ Yrs
0	60- 69 Yrs
0	< 10 Yrs
0	40- 49 Yrs
0	70+ Yrs
0	50- 59 Yrs
0	60- 69 Yrs
0	50- 59 Yrs
0	60- 69 Yrs
0	70+ Yrs
0	40- 49 Yrs
0	70+ Yrs
0	70+ Yrs
0	40- 49 Yrs

0 10- 19 Yrs
0 70+ Yrs
0 50- 59 Yrs
0 60- 69 Yrs
0 40- 49 Yrs
0 50- 59 Yrs
0 70+ Yrs
0 60- 69 Yrs
0 60- 69 Yrs
0 50- 59 Yrs
0 70+ Yrs
0 70+ Yrs
0 60- 69 Yrs
0 < 10 Yrs
0 < 10 Yrs
0 40- 49 Yrs
0 40- 49 Yrs
0 50- 59 Yrs
0 60- 69 Yrs
0 70+ Yrs
0 40- 49 Yrs
0 60- 69 Yrs
0 40- 49 Yrs
0 30- 39 Yrs
0 50- 59 Yrs
0 50- 59 Yrs
0 40- 49 Yrs
0 40- 49 Yrs
0 30- 39 Yrs
0 50- 59 Yrs
0 60- 69 Yrs
0 40- 49 Yrs
0 50- 59 Yrs
0 30- 39 Yrs
0 40- 49 Yrs
0 40- 49 Yrs
0 50- 59 Yrs
0 60- 69 Yrs
0 50- 59 Yrs
0 50- 59 Yrs
0 50- 59 Yrs
0 40- 49 Yrs
0 60- 69 Yrs
0 30- 39 Yrs
0 40- 49 Yrs
0 60- 69 Yrs
0 50- 59 Yrs
0 40- 49 Yrs
0 40- 49 Yrs
0 30- 39 Yrs

0	50- 59 Yrs
0	30- 39 Yrs
0	50- 59 Yrs
0	60- 69 Yrs
0	40- 49 Yrs
0	40- 49 Yrs
0	40- 49 Yrs
0	30- 39 Yrs
0	40- 49 Yrs
0	60- 69 Yrs
0	50- 59 Yrs
0	50- 59 Yrs
0	50- 59 Yrs
0	40- 49 Yrs
0	50- 59 Yrs
0	40- 49 Yrs
0	50- 59 Yrs
0	60- 69 Yrs
0	30- 39 Yrs
0	40- 49 Yrs
0	30- 39 Yrs
0	50- 59 Yrs
0	40- 49 Yrs
0	50- 59 Yrs
0	60- 69 Yrs
0	60- 69 Yrs
0	50- 59 Yrs
0	50- 59 Yrs
0	40- 49 Yrs
0	40- 49 Yrs
0	30- 39 Yrs
0	40- 49 Yrs
0	30- 39 Yrs
0	60- 69 Yrs
0	50- 59 Yrs
0	40- 49 Yrs
0	50- 59 Yrs
0	60- 69 Yrs
0	50- 59 Yrs
0	50- 59 Yrs
0	40- 49 Yrs
0	30- 39 Yrs
0	30- 39 Yrs
0	20- 29 Yrs
0	10- 19 Yrs
0	30- 39 Yrs
0	20- 29 Yrs
0	10- 19 Yrs
0	30- 39 Yrs
0	70+ Yrs

0	60- 69 Yrs
0	70+ Yrs
0	70+ Yrs
0	60- 69 Yrs
0	50- 59 Yrs
0	50- 59 Yrs
0	30- 39 Yrs
0	30- 39 Yrs
0	40- 49 Yrs
0	70+ Yrs
0	50- 59 Yrs
0	30- 39 Yrs
0	20- 29 Yrs
0	10- 19 Yrs
0	30- 39 Yrs
0	40- 49 Yrs
0	50- 59 Yrs
0	30- 39 Yrs
0	20- 29 Yrs
0	10- 19 Yrs
0	60- 69 Yrs
0	60- 69 Yrs
0	70+ Yrs
0	30- 39 Yrs
0	20- 29 Yrs
0	30- 39 Yrs
0	30- 39 Yrs
0	60- 69 Yrs
0	10- 19 Yrs
0	50- 59 Yrs
0	30- 39 Yrs
0	40- 49 Yrs
0	50- 59 Yrs
0	60- 69 Yrs
0	30- 39 Yrs
0	10- 19 Yrs
0	20- 29 Yrs
0	20- 29 Yrs
0	10- 19 Yrs
0	30- 39 Yrs
0	50- 59 Yrs
0	40- 49 Yrs
0	30- 39 Yrs
0	60- 69 Yrs
0	20- 29 Yrs
0	30- 39 Yrs
0	30- 39 Yrs
0	60- 69 Yrs
0	70+ Yrs
0	50- 59 Yrs

0 50- 59 Yrs
0 10- 19 Yrs
0 20- 29 Yrs
0 60- 69 Yrs
0 10- 19 Yrs
0 50- 59 Yrs
0 60- 69 Yrs
0 30- 39 Yrs
0 70+ Yrs
0 50- 59 Yrs
0 50- 59 Yrs
0 40- 49 Yrs
0 70+ Yrs
0 30- 39 Yrs
0 60- 69 Yrs
0 30- 39 Yrs
0 20- 29 Yrs
0 10- 19 Yrs
0 10- 19 Yrs
0 60- 69 Yrs
0 60- 69 Yrs
0 20- 29 Yrs
0 50- 59 Yrs
0 40- 49 Yrs
0 20- 29 Yrs
0 20- 29 Yrs
0 30- 39 Yrs
0 10- 19 Yrs
0 40- 49 Yrs
0 < 10 Yrs
0 40- 49 Yrs
0 < 10 Yrs
0 30- 39 Yrs
0 30- 39 Yrs
0 40- 49 Yrs
0 60- 69 Yrs
0 70+ Yrs
0 60- 69 Yrs
0 50- 59 Yrs
0 50- 59 Yrs
0 70+ Yrs
0 10- 19 Yrs
0 10- 19 Yrs
0 20- 29 Yrs
0 20- 29 Yrs
0 40- 49 Yrs
0 50- 59 Yrs
0 50- 59 Yrs
0 40- 49 Yrs
0 60- 69 Yrs

0	60- 69 Yrs
0	70+ Yrs
0	70+ Yrs
0	< 10 Yrs
0	30- 39 Yrs
0	10- 19 Yrs
0	10- 19 Yrs
0	20- 29 Yrs
0	20- 29 Yrs
0	30- 39 Yrs
0	< 10 Yrs
0	20- 29 Yrs
0	10- 19 Yrs
0	10- 19 Yrs
0	20- 29 Yrs
0	40- 49 Yrs
0	30- 39 Yrs
0	60- 69 Yrs
0	50- 59 Yrs
0	50- 59 Yrs
0	40- 49 Yrs
0	10- 19 Yrs
0	10- 19 Yrs
0	30- 39 Yrs
0	20- 29 Yrs
0	20- 29 Yrs
0	60- 69 Yrs
0	< 10 Yrs
0	70+ Yrs
0	< 10 Yrs
0	70+ Yrs
0	20- 29 Yrs
0	10- 19 Yrs
0	10- 19 Yrs
0	70+ Yrs
0	< 10 Yrs
0	30- 39 Yrs
0	40- 49 Yrs
0	40- 49 Yrs
0	< 10 Yrs
0	50- 59 Yrs
0	60- 69 Yrs
0	60- 69 Yrs
0	20- 29 Yrs
0	20- 29 Yrs
0	50- 59 Yrs
0	30- 39 Yrs
0	70+ Yrs
0	40- 49 Yrs
0	40- 49 Yrs

0 60- 69 Yrs
0 60- 69 Yrs
0 < 10 Yrs
0 < 10 Yrs
0 70+ Yrs
0 70+ Yrs
0 10- 19 Yrs
0 30- 39 Yrs
0 20- 29 Yrs
0 30- 39 Yrs
0 20- 29 Yrs
0 50- 59 Yrs
0 50- 59 Yrs
0 40- 49 Yrs
0 10- 19 Yrs
0 40- 49 Yrs
0 70+ Yrs
0 70+ Yrs
0 10- 19 Yrs
0 70+ Yrs
0 70+ Yrs
0 70+ Yrs
0 20- 29 Yrs
0 10- 19 Yrs
0 70+ Yrs
0 60- 69 Yrs
0 60- 69 Yrs
0 50- 59 Yrs
0 70+ Yrs
0 50- 59 Yrs
0 30- 39 Yrs
0 20- 29 Yrs
0 10- 19 Yrs
0 20- 29 Yrs
0 40- 49 Yrs
0 40- 49 Yrs
0 30- 39 Yrs
0 60- 69 Yrs
0 70+ Yrs
0 70+ Yrs
0 50- 59 Yrs
0 60- 69 Yrs
0 50- 59 Yrs
0 40- 49 Yrs
0 30- 39 Yrs
0 30- 39 Yrs
0 40- 49 Yrs
0 10- 19 Yrs
0 20- 29 Yrs
0 20- 29 Yrs

0 60- 69 Yrs
0 40- 49 Yrs
0 50- 59 Yrs
0 70+ Yrs
0 50- 59 Yrs
0 70+ Yrs
0 60- 69 Yrs
0 10- 19 Yrs
0 30- 39 Yrs
0 40- 49 Yrs
0 30- 39 Yrs
0 20- 29 Yrs
0 20- 29 Yrs
0 70+ Yrs
0 10- 19 Yrs
0 50- 59 Yrs
0 40- 49 Yrs
0 40- 49 Yrs
0 60- 69 Yrs
0 50- 59 Yrs
0 60- 69 Yrs
0 10- 19 Yrs
0 20- 29 Yrs
0 30- 39 Yrs
0 30- 39 Yrs
0 20- 29 Yrs
0 70+ Yrs
0 70+ Yrs
0 70+ Yrs
0 70+ Yrs
0 10- 19 Yrs
0 20- 29 Yrs
0 60- 69 Yrs
0 60- 69 Yrs
0 40- 49 Yrs
0 40- 49 Yrs
0 20- 29 Yrs
0 50- 59 Yrs
0 30- 39 Yrs
0 70+ Yrs
0 70+ Yrs
0 50- 59 Yrs
0 10- 19 Yrs
0 30- 39 Yrs
0 70+ Yrs
0 70+ Yrs
0 30- 39 Yrs
0 40- 49 Yrs
0 30- 39 Yrs
0 10- 19 Yrs

0	20- 29 Yrs
0	20- 29 Yrs
0	70+ Yrs
0	70+ Yrs
0	50- 59 Yrs
0	50- 59 Yrs
0	40- 49 Yrs
0	60- 69 Yrs
0	60- 69 Yrs
0	70+ Yrs
0	70+ Yrs
0	60- 69 Yrs
0	60- 69 Yrs
0	50- 59 Yrs
0	50- 59 Yrs
0	40- 49 Yrs
0	30- 39 Yrs
0	10- 19 Yrs
0	50- 59 Yrs
0	40- 49 Yrs
0	60- 69 Yrs
0	60- 69 Yrs
0	70+ Yrs
0	50- 59 Yrs
0	70+ Yrs
0	10- 19 Yrs
0	30- 39 Yrs
0	70+ Yrs
0	70+ Yrs
0	60- 69 Yrs
0	60- 69 Yrs
0	50- 59 Yrs
0	50- 59 Yrs
0	40- 49 Yrs
0	30- 39 Yrs
0	10- 19 Yrs
0	60- 69 Yrs
0	60- 69 Yrs
0	70+ Yrs
0	70+ Yrs
0	50- 59 Yrs
0	10- 19 Yrs
0	30- 39 Yrs
0	40- 49 Yrs
0	50- 59 Yrs
0	50- 59 Yrs
0	50- 59 Yrs
0	30- 39 Yrs
0	60- 69 Yrs
0	40- 49 Yrs

0	60- 69 Yrs
0	70+ Yrs
0	70+ Yrs
0	10- 19 Yrs
0	70+ Yrs
0	70+ Yrs
0	50- 59 Yrs
0	50- 59 Yrs
0	30- 39 Yrs
0	10- 19 Yrs
0	70+ Yrs
0	60- 69 Yrs
0	60- 69 Yrs
0	50- 59 Yrs
0	40- 49 Yrs
0	30- 39 Yrs
0	10- 19 Yrs
0	50- 59 Yrs
0	70+ Yrs
0	70+ Yrs
0	70+ Yrs
0	10- 19 Yrs
0	30- 39 Yrs
0	40- 49 Yrs
0	50- 59 Yrs
0	50- 59 Yrs
0	60- 69 Yrs
0	60- 69 Yrs
0	10- 19 Yrs
0	50- 59 Yrs
0	60- 69 Yrs
0	50- 59 Yrs
0	30- 39 Yrs
0	40- 49 Yrs
0	60- 69 Yrs
0	70+ Yrs
0	70+ Yrs
0	70+ Yrs
0	40- 49 Yrs
0	10- 19 Yrs
0	60- 69 Yrs
0	70+ Yrs
0	60- 69 Yrs
0	50- 59 Yrs
0	60- 69 Yrs
0	60- 69 Yrs
0	70+ Yrs
0	10- 19 Yrs
0	40- 49 Yrs
0	50- 59 Yrs

0 40- 49 Yrs
0 30- 39 Yrs
0 50- 59 Yrs
0 60- 69 Yrs
0 50- 59 Yrs
0 70+ Yrs
0 70+ Yrs
0 60- 69 Yrs
0 60- 69 Yrs
0 30- 39 Yrs
0 10- 19 Yrs
0 30- 39 Yrs
0 10- 19 Yrs
0 20- 29 Yrs
0 20- 29 Yrs
0 40- 49 Yrs
0 50- 59 Yrs
0 50- 59 Yrs
0 40- 49 Yrs
0 20- 29 Yrs
0 10- 19 Yrs
0 10- 19 Yrs
0 20- 29 Yrs
0 70+ Yrs
0 70+ Yrs
0 40- 49 Yrs
0 40- 49 Yrs
0 30- 39 Yrs
0 30- 39 Yrs
0 60- 69 Yrs
0 50- 59 Yrs
0 50- 59 Yrs
0 60- 69 Yrs
0 10- 19 Yrs
0 10- 19 Yrs
0 30- 39 Yrs
0 40- 49 Yrs
0 40- 49 Yrs
0 20- 29 Yrs
0 30- 39 Yrs
0 20- 29 Yrs
0 60- 69 Yrs
0 70+ Yrs
0 70+ Yrs
0 50- 59 Yrs
0 50- 59 Yrs
0 60- 69 Yrs
0 20- 29 Yrs
0 30- 39 Yrs
0 20- 29 Yrs

0 10- 19 Yrs
0 10- 19 Yrs
0 70+ Yrs
0 60- 69 Yrs
0 70+ Yrs
0 30- 39 Yrs
0 40- 49 Yrs
0 50- 59 Yrs
0 50- 59 Yrs
0 40- 49 Yrs
0 60- 69 Yrs
0 40- 49 Yrs
0 50- 59 Yrs
0 40- 49 Yrs
0 30- 39 Yrs
0 20- 29 Yrs
0 10- 19 Yrs
0 10- 19 Yrs
0 30- 39 Yrs
0 20- 29 Yrs
0 60- 69 Yrs
0 60- 69 Yrs
0 70+ Yrs
0 50- 59 Yrs
0 70+ Yrs
0 10- 19 Yrs
0 50- 59 Yrs
0 60- 69 Yrs
0 50- 59 Yrs
0 70+ Yrs
0 70+ Yrs
0 60- 69 Yrs
0 40- 49 Yrs
0 20- 29 Yrs
0 10- 19 Yrs
0 20- 29 Yrs
0 40- 49 Yrs
0 30- 39 Yrs
0 30- 39 Yrs
0 10- 19 Yrs
0 50- 59 Yrs
0 50- 59 Yrs
0 10- 19 Yrs
0 30- 39 Yrs
0 20- 29 Yrs
0 40- 49 Yrs
0 30- 39 Yrs
0 40- 49 Yrs
0 20- 29 Yrs
0 60- 69 Yrs

0 70+ Yrs
0 70+ Yrs
0 60- 69 Yrs
0 50- 59 Yrs
0 60- 69 Yrs
0 70+ Yrs
0 40- 49 Yrs
0 30- 39 Yrs
0 70+ Yrs
0 60- 69 Yrs
0 50- 59 Yrs
0 40- 49 Yrs
0 20- 29 Yrs
0 20- 29 Yrs
0 30- 39 Yrs
0 10- 19 Yrs
0 10- 19 Yrs
0 70+ Yrs
0 60- 69 Yrs
0 40- 49 Yrs
0 10- 19 Yrs
0 10- 19 Yrs
0 20- 29 Yrs
0 30- 39 Yrs
0 30- 39 Yrs
0 20- 29 Yrs
0 40- 49 Yrs
0 60- 69 Yrs
0 50- 59 Yrs
0 50- 59 Yrs
0 70+ Yrs
0 60- 69 Yrs
0 10- 19 Yrs
0 10- 19 Yrs
0 40- 49 Yrs
0 20- 29 Yrs
0 30- 39 Yrs
0 20- 29 Yrs
0 50- 59 Yrs
0 40- 49 Yrs
0 40- 49 Yrs
0 50- 59 Yrs
0 40- 49 Yrs
0 30- 39 Yrs
0 50- 59 Yrs
0 60- 69 Yrs
0 60- 69 Yrs
0 70+ Yrs
0 70+ Yrs
0 30- 39 Yrs

0 30- 39 Yrs
0 40- 49 Yrs
0 40- 49 Yrs
0 50- 59 Yrs
0 50- 59 Yrs
0 60- 69 Yrs
0 60- 69 Yrs
0 70+ Yrs
0 50- 59 Yrs
0 50- 59 Yrs
0 40- 49 Yrs
0 40- 49 Yrs
0 30- 39 Yrs
0 70+ Yrs
0 60- 69 Yrs
0 60- 69 Yrs
0 70+ Yrs
0 30- 39 Yrs
0 60- 69 Yrs
0 40- 49 Yrs
0 40- 49 Yrs
0 60- 69 Yrs
0 50- 59 Yrs
0 50- 59 Yrs
0 60- 69 Yrs
0 50- 59 Yrs
0 50- 59 Yrs
0 40- 49 Yrs
0 40- 49 Yrs
0 30- 39 Yrs
0 60- 69 Yrs
0 70+ Yrs
0 70+ Yrs
0 60- 69 Yrs
0 50- 59 Yrs
0 60- 69 Yrs
0 40- 49 Yrs
0 50- 59 Yrs
0 40- 49 Yrs
0 30- 39 Yrs
0 60- 69 Yrs
0 60- 69 Yrs
0 40- 49 Yrs
0 30- 39 Yrs
0 40- 49 Yrs
0 50- 59 Yrs
0 50- 59 Yrs
0 70+ Yrs
0 40- 49 Yrs
0 50- 59 Yrs

0 60- 69 Yrs
0 30- 39 Yrs
0 50- 59 Yrs
0 70+ Yrs
0 40- 49 Yrs
0 60- 69 Yrs
0 50- 59 Yrs
0 50- 59 Yrs
0 40- 49 Yrs
0 40- 49 Yrs
0 70+ Yrs
0 60- 69 Yrs
0 30- 39 Yrs
0 60- 69 Yrs
0 70+ Yrs
0 40- 49 Yrs
0 40- 49 Yrs
0 30- 39 Yrs
0 60- 69 Yrs
0 60- 69 Yrs
0 50- 59 Yrs
0 50- 59 Yrs
0 10- 19 Yrs
0 50- 59 Yrs
0 60- 69 Yrs
0 50- 59 Yrs
0 70+ Yrs
0 40- 49 Yrs
0 50- 59 Yrs
0 10- 19 Yrs
0 40- 49 Yrs
0 50- 59 Yrs
0 60- 69 Yrs
0 50- 59 Yrs
0 60- 69 Yrs
0 70+ Yrs
0 50- 59 Yrs
0 10- 19 Yrs
0 40- 49 Yrs
0 60- 69 Yrs
0 50- 59 Yrs
0 70+ Yrs
0 40- 49 Yrs
0 50- 59 Yrs
0 10- 19 Yrs
0 70+ Yrs
0 50- 59 Yrs
0 40- 49 Yrs
0 60- 69 Yrs
0 50- 59 Yrs

0	10- 19 Yrs
0	70+ Yrs
0	60- 69 Yrs
0	50- 59 Yrs
0	40- 49 Yrs
0	10- 19 Yrs
0	10- 19 Yrs
0	40- 49 Yrs
0	50- 59 Yrs
0	50- 59 Yrs
0	60- 69 Yrs
0	70+ Yrs
0	40- 49 Yrs
0	50- 59 Yrs
0	50- 59 Yrs
0	60- 69 Yrs
0	10- 19 Yrs
0	70+ Yrs
0	10- 19 Yrs
0	40- 49 Yrs
0	70+ Yrs
0	60- 69 Yrs
0	50- 59 Yrs
0	40- 49 Yrs
0	70+ Yrs
0	10- 19 Yrs
0	50- 59 Yrs
0	50- 59 Yrs
0	70+ Yrs
0	60- 69 Yrs
0	10- 19 Yrs
0	40- 49 Yrs
0	50- 59 Yrs
0	50- 59 Yrs
0	70+ Yrs
0	60- 69 Yrs
0	50- 59 Yrs
0	40- 49 Yrs
0	40- 49 Yrs
0	60- 69 Yrs
0	60- 69 Yrs
0	70+ Yrs
0	70+ Yrs
0	20- 29 Yrs
0	40- 49 Yrs
0	20- 29 Yrs
0	30- 39 Yrs
0	40- 49 Yrs
0	30- 39 Yrs
0	50- 59 Yrs

0 10- 19 Yrs
0 10- 19 Yrs
0 50- 59 Yrs
0 50- 59 Yrs
0 10- 19 Yrs
0 50- 59 Yrs
0 40- 49 Yrs
0 40- 49 Yrs
0 50- 59 Yrs
0 40- 49 Yrs
0 30- 39 Yrs
0 20- 29 Yrs
0 50- 59 Yrs
0 40- 49 Yrs
0 40- 49 Yrs
0 30- 39 Yrs
0 40- 49 Yrs
0 20- 29 Yrs
0 10- 19 Yrs
0 10- 19 Yrs
0 50- 59 Yrs
0 70+ Yrs
0 70+ Yrs
0 50- 59 Yrs
0 60- 69 Yrs
0 60- 69 Yrs
0 40- 49 Yrs
0 50- 59 Yrs
0 40- 49 Yrs
0 50- 59 Yrs
0 50- 59 Yrs
0 40- 49 Yrs
0 50- 59 Yrs
0 10- 19 Yrs
0 50- 59 Yrs
0 40- 49 Yrs
0 50- 59 Yrs
0 10- 19 Yrs
0 40- 49 Yrs
0 10- 19 Yrs
0 50- 59 Yrs
0 50- 59 Yrs
0 60- 69 Yrs
0 60- 69 Yrs
0 50- 59 Yrs
0 60- 69 Yrs
0 60- 69 Yrs
0 50- 59 Yrs
0 50- 59 Yrs
0 50- 59 Yrs

0 60- 69 Yrs
0 50- 59 Yrs
0 60- 69 Yrs
0 60- 69 Yrs
0 60- 69 Yrs
0 50- 59 Yrs
0 50- 59 Yrs
0 60- 69 Yrs
0 60- 69 Yrs
0 50- 59 Yrs
0 50- 59 Yrs
0 50- 59 Yrs
0 50- 59 Yrs
0 50- 59 Yrs
0 60- 69 Yrs
0 60- 69 Yrs
0 60- 69 Yrs
0 50- 59 Yrs
0 60- 69 Yrs
0 60- 69 Yrs
0 50- 59 Yrs
0 50- 59 Yrs
0 60- 69 Yrs
0 60- 69 Yrs
0 50- 59 Yrs
0 50- 59 Yrs
0 60- 69 Yrs
0 60- 69 Yrs
0 60- 69 Yrs
0 50- 59 Yrs
0 50- 59 Yrs
0 60- 69 Yrs
0 60- 69 Yrs
0 50- 59 Yrs
0 50- 59 Yrs
0 60- 69 Yrs
0 60- 69 Yrs
0 50- 59 Yrs
0 50- 59 Yrs
0 50- 59 Yrs
0 50- 59 Yrs
0 50- 59 Yrs
0 50- 59 Yrs
0 60- 69 Yrs
0 50- 59 Yrs
0 50- 59 Yrs
0 60- 69 Yrs
0 60- 69 Yrs
0 60- 69 Yrs
0 60- 69 Yrs

0 60- 69 Yrs
0 50- 59 Yrs
0 50- 59 Yrs
0 60- 69 Yrs
0 60- 69 Yrs
0 60- 69 Yrs
0 50- 59 Yrs
0 50- 59 Yrs
0 60- 69 Yrs
0 60- 69 Yrs
0 50- 59 Yrs
0 50- 59 Yrs
0
0
0
0
0
0
0
0
0
0
0
0
0
0
0
0
0
0
0
0
0
0
0
0
0
0
0
0
0
0
0
0
0
0
0
0
0
0

0
0
0
0
0
0
0
0
0
0
0
0
0
0
0
0
0
0
0
0
0
0
0
0
0
0
0
0
0
0
0
0
0
0
0
0
0
0
0
0
0
0
0
0
0
0
0
0
0
0

0
0
0
0
0
0
0
0
0
0
0
0
0
0
0
0
0
0
0
0
0
0
0
0
0
0
0
0
0
0
0
0
0
0
0
0
0
0
0
0
0
0
0
0
0
0
0
0
0
0

0
0
0
0
0
0
0
0
0
0
0
0
0
0
0
0
0
0
0
0
0
0
0
0
0
0
0
0
0
0
0
0
0
0
0
0
0
0
0
0
0
0
0
0
0
0
0
0
0
0

0	
0	
0	
0	
0	
0	
0	
0	
0	
0	
0	
0	
0	
0	
0	
0	
0	
0	
0	
0	
0	
0	
0	
0	
0	
0	
0	
0	
0	
0	
0	
0	
0	
0	
0	
0	
0	
0	
0	
0	
0	
1	70+ Yrs
1	60- 69 Yrs
1	70+ Yrs
1	10- 19 Yrs
1	40- 49 Yrs
1	60- 69 Yrs
1	< 10 Yrs
1	50- 59 Yrs
1	60- 69 Yrs

1	60- 69 Yrs
1	70+ Yrs
1	70+ Yrs
1	50- 59 Yrs
1	40- 49 Yrs
1	50- 59 Yrs
1	60- 69 Yrs
1	60- 69 Yrs
1	60- 69 Yrs
1	50- 59 Yrs
1	30- 39 Yrs
1	20- 29 Yrs
1	70+ Yrs
1	60- 69 Yrs
1	70+ Yrs
1	60- 69 Yrs
1	50- 59 Yrs
1	30- 39 Yrs
1	10- 19 Yrs
1	10- 19 Yrs
1	50- 59 Yrs
1	70+ Yrs
1	40- 49 Yrs
1	30- 39 Yrs
1	10- 19 Yrs
1	60- 69 Yrs
1	70+ Yrs
1	< 10 Yrs
1	60- 69 Yrs
1	60- 69 Yrs
1	40- 49 Yrs
1	30- 39 Yrs
1	30- 39 Yrs
1	70+ Yrs
1	50- 59 Yrs
1	50- 59 Yrs
1	70+ Yrs
1	40- 49 Yrs
1	50- 59 Yrs
1	60- 69 Yrs
1	70+ Yrs
1	< 10 Yrs
1	70+ Yrs
1	60- 69 Yrs
1	60- 69 Yrs
1	70+ Yrs
1	20- 29 Yrs
1	70+ Yrs
1	50- 59 Yrs
1	40- 49 Yrs

1	40- 49 Yrs
1	30- 39 Yrs
1	60- 69 Yrs
1	70+ Yrs
1	60- 69 Yrs
1	60- 69 Yrs
1	10- 19 Yrs
1	50- 59 Yrs
1	50- 59 Yrs
1	70+ Yrs
1	70+ Yrs
1	50- 59 Yrs
1	30- 39 Yrs
1	10- 19 Yrs
1	20- 29 Yrs
1	20- 29 Yrs
1	60- 69 Yrs
1	10- 19 Yrs
1	70+ Yrs
1	50- 59 Yrs
1	30- 39 Yrs
1	20- 29 Yrs
1	20- 29 Yrs
1	60- 69 Yrs
1	60- 69 Yrs
1	70+ Yrs
1	10- 19 Yrs
1	< 10 Yrs
1	60- 69 Yrs
1	70+ Yrs
1	70+ Yrs
1	30- 39 Yrs
1	70+ Yrs
1	50- 59 Yrs
1	
1	50- 59 Yrs
1	10- 19 Yrs
1	10- 19 Yrs
1	70+ Yrs
1	70+ Yrs
1	20- 29 Yrs
1	30- 39 Yrs
1	20- 29 Yrs
1	10- 19 Yrs
1	
1	
1	10- 19 Yrs
1	10- 19 Yrs
1	20- 29 Yrs
1	

1 50- 59 Yrs
1
1 60- 69 Yrs
1 40- 49 Yrs
1 70+ Yrs
1 50- 59 Yrs
1
1
1 20- 29 Yrs
1 10- 19 Yrs
1
1
1
1
1
1
1
1
1 10- 19 Yrs
1 50- 59 Yrs
1 20- 29 Yrs
1
1
1 50- 59 Yrs
1
1
1 50- 59 Yrs
1
1 10- 19 Yrs
1 40- 49 Yrs
1 10- 19 Yrs
1
1
1 10- 19 Yrs
1 40- 49 Yrs
1 10- 19 Yrs
1
1
1 20- 29 Yrs
1 20- 29 Yrs
1
1 60- 69 Yrs
1 30- 39 Yrs
1 20- 29 Yrs
1 20- 29 Yrs
1 30- 39 Yrs
1 40- 49 Yrs
1 60- 69 Yrs
1 60- 69 Yrs
1 50- 59 Yrs

1 30- 39 Yrs
1 40- 49 Yrs
1 50- 59 Yrs
1 30- 39 Yrs
1 70+ Yrs
1 50- 59 Yrs
1 60- 69 Yrs
1 20- 29 Yrs
1 50- 59 Yrs
1 30- 39 Yrs
1 60- 69 Yrs
1 50- 59 Yrs
1 50- 59 Yrs
1 70+ Yrs
1 70+ Yrs
1 60- 69 Yrs
1 40- 49 Yrs
1 30- 39 Yrs
1 60- 69 Yrs
1 40- 49 Yrs
1 70+ Yrs
1 50- 59 Yrs
1 50- 59 Yrs
1 40- 49 Yrs
1 60- 69 Yrs
1 20- 29 Yrs
1 30- 39 Yrs
1 50- 59 Yrs
1 50- 59 Yrs
1 20- 29 Yrs
1 70+ Yrs
1 70+ Yrs
1 60- 69 Yrs
1 50- 59 Yrs
1 30- 39 Yrs
1 60- 69 Yrs
1 50- 59 Yrs
1 50- 59 Yrs
1 60- 69 Yrs
1 70+ Yrs
1 20- 29 Yrs
1 70+ Yrs
1 30- 39 Yrs
1 70+ Yrs
1 60- 69 Yrs
1 50- 59 Yrs
1 50- 59 Yrs
1 30- 39 Yrs
1 40- 49 Yrs
1 30- 39 Yrs

1	70+ Yrs
1	50- 59 Yrs
1	60- 69 Yrs
1	70+ Yrs
1	60- 69 Yrs
1	30- 39 Yrs
1	60- 69 Yrs
1	20- 29 Yrs
1	30- 39 Yrs
1	40- 49 Yrs
1	60- 69 Yrs
1	60- 69 Yrs
1	50- 59 Yrs
1	50- 59 Yrs
1	70+ Yrs
1	60- 69 Yrs
1	60- 69 Yrs
1	40- 49 Yrs
1	50- 59 Yrs
1	50- 59 Yrs
1	30- 39 Yrs
1	70+ Yrs
1	60- 69 Yrs
1	70+ Yrs
1	60- 69 Yrs
1	60- 69 Yrs
1	40- 49 Yrs
1	30- 39 Yrs
1	50- 59 Yrs
1	50- 59 Yrs
1	60- 69 Yrs
1	40- 49 Yrs
1	60- 69 Yrs
1	40- 49 Yrs
1	30- 39 Yrs
1	40- 49 Yrs
1	20- 29 Yrs
1	50- 59 Yrs
1	20- 29 Yrs
1	40- 49 Yrs
1	20- 29 Yrs
1	40- 49 Yrs
1	30- 39 Yrs
1	70+ Yrs
1	50- 59 Yrs
1	50- 59 Yrs
1	60- 69 Yrs
1	70+ Yrs
1	70+ Yrs
1	20- 29 Yrs

1	40- 49 Yrs
1	50- 59 Yrs
1	30- 39 Yrs
1	40- 49 Yrs
1	20- 29 Yrs
1	40- 49 Yrs
1	60- 69 Yrs
1	70+ Yrs
1	50- 59 Yrs
1	60- 69 Yrs
1	20- 29 Yrs
1	40- 49 Yrs
1	60- 69 Yrs
1	30- 39 Yrs
1	70+ Yrs
1	60- 69 Yrs
1	40- 49 Yrs
1	70+ Yrs
1	60- 69 Yrs
1	50- 59 Yrs
1	70+ Yrs
1	60- 69 Yrs
1	60- 69 Yrs
1	40- 49 Yrs
1	40- 49 Yrs
1	70+ Yrs
1	70+ Yrs
1	50- 59 Yrs
1	50- 59 Yrs
1	70+ Yrs
1	50- 59 Yrs
1	60- 69 Yrs
1	70+ Yrs
1	50- 59 Yrs
1	30- 39 Yrs
1	40- 49 Yrs
1	60- 69 Yrs
3	70+ Yrs
2	60- 69 Yrs
4	60- 69 Yrs
5	
2	60- 69 Yrs
2	20- 29 Yrs
2	10- 19 Yrs
2	60- 69 Yrs
3	50- 59 Yrs
14	
19	60- 69 Yrs
12	60- 69 Yrs
15	50- 59 Yrs

23	50- 59 Yrs
29	
6	
13	
2	
21	
2	
3	
3	
4	60- 69 Yrs
3	70+ Yrs
6	
6	60- 69 Yrs
3	60- 69 Yrs
3	
5	50- 59 Yrs
3	
5	
3	70+ Yrs
2	50- 59 Yrs
3	70+ Yrs
2	60- 69 Yrs
2	60- 69 Yrs
2	60- 69 Yrs
3	
2	60- 69 Yrs
3	40- 49 Yrs
2	70+ Yrs
4	30- 39 Yrs
2	40- 49 Yrs
3	
7	
2	
2	60- 69 Yrs
9	
8	50- 59 Yrs
3	50- 59 Yrs
5	60- 69 Yrs
5	
2	
6	
11	50- 59 Yrs
5	50- 59 Yrs
3	60- 69 Yrs
8	60- 69 Yrs
3	
4	
2	
6	
2	

6	
2	
7	
3	
5	50- 59 Yrs
3	50- 59 Yrs
10	60- 69 Yrs
17	60- 69 Yrs
12	
10	
2	70+ Yrs
2	50- 59 Yrs
2	70+ Yrs
2	30- 39 Yrs
3	20- 29 Yrs
4	20- 29 Yrs
5	50- 59 Yrs
5	40- 49 Yrs
4	40- 49 Yrs
4	30- 39 Yrs
3	50- 59 Yrs
6	30- 39 Yrs
2	20- 29 Yrs
6	30- 39 Yrs
2	20- 29 Yrs
2	70+ Yrs
2	50- 59 Yrs
4	30- 39 Yrs
7	40- 49 Yrs
4	40- 49 Yrs
273	40- 49 Yrs
178	30- 39 Yrs
234	40- 49 Yrs
9	20- 29 Yrs
4	20- 29 Yrs
204	30- 39 Yrs
9	60- 69 Yrs
9	60- 69 Yrs
142	50- 59 Yrs
114	50- 59 Yrs
2	50- 59 Yrs
2	40- 49 Yrs
3	40- 49 Yrs
2	30- 39 Yrs
8	70+ Yrs
8	70+ Yrs
11	60- 69 Yrs
9	60- 69 Yrs
224	50- 59 Yrs
149	50- 59 Yrs

191	40- 49 Yrs
141	30- 39 Yrs
124	30- 39 Yrs
16	20- 29 Yrs
21	20- 29 Yrs
201	40- 49 Yrs
6	50- 59 Yrs
7	50- 59 Yrs
4	20- 29 Yrs
8	30- 39 Yrs
7	30- 39 Yrs
9	40- 49 Yrs
11	40- 49 Yrs
5	20- 29 Yrs
3	50- 59 Yrs
2	50- 59 Yrs
8	40- 49 Yrs
6	20- 29 Yrs
5	20- 29 Yrs
4	30- 39 Yrs
8	40- 49 Yrs
7	30- 39 Yrs
5	30- 39 Yrs
8	50- 59 Yrs
2	10- 19 Yrs
4	30- 39 Yrs
4	60- 69 Yrs
8	40- 49 Yrs
2	50- 59 Yrs
14	40- 49 Yrs
2	50- 59 Yrs
3	70+ Yrs
3	70+ Yrs
17	60- 69 Yrs
19	60- 69 Yrs
21	50- 59 Yrs
20	50- 59 Yrs
22	40- 49 Yrs
10	40- 49 Yrs
2	50- 59 Yrs
2	60- 69 Yrs
2	50- 59 Yrs
3	40- 49 Yrs
2	30- 39 Yrs
5	< 10 Yrs
6	70+ Yrs
30	60- 69 Yrs
17	60- 69 Yrs
12	70+ Yrs
13	30- 39 Yrs

7	30- 39 Yrs
6	20- 29 Yrs
2	20- 29 Yrs
36	50- 59 Yrs
21	50- 59 Yrs
31	40- 49 Yrs
15	40- 49 Yrs
3	10- 19 Yrs
2	10- 19 Yrs
9	10- 19 Yrs
4	10- 19 Yrs
7	20- 29 Yrs
3	20- 29 Yrs
12	30- 39 Yrs
4	30- 39 Yrs
11	40- 49 Yrs
3	40- 49 Yrs
7	50- 59 Yrs
6	50- 59 Yrs
3	60- 69 Yrs
2	60- 69 Yrs
3	< 10 Yrs
2	< 10 Yrs
32	40- 49 Yrs
12	60- 69 Yrs
19	30- 39 Yrs
29	50- 59 Yrs
20	50- 59 Yrs
2	70+ Yrs
11	60- 69 Yrs
26	40- 49 Yrs
2	10- 19 Yrs
20	30- 39 Yrs
3	10- 19 Yrs
12	20- 29 Yrs
7	20- 29 Yrs
2	10- 19 Yrs
4	60- 69 Yrs
2	60- 69 Yrs
11	50- 59 Yrs
7	50- 59 Yrs
13	40- 49 Yrs
20	40- 49 Yrs
4	10- 19 Yrs
5	20- 29 Yrs
6	20- 29 Yrs
19	30- 39 Yrs
7	30- 39 Yrs
2	< 10 Yrs
19	40- 49 Yrs

30	40- 49 Yrs
10	30- 39 Yrs
14	30- 39 Yrs
25	50- 59 Yrs
12	70+ Yrs
7	60- 69 Yrs
5	20- 29 Yrs
15	60- 69 Yrs
20	50- 59 Yrs
3	20- 29 Yrs
3	10- 19 Yrs
10	70+ Yrs
2	< 10 Yrs
3	< 10 Yrs
5	60- 69 Yrs
53	40- 49 Yrs
26	50- 59 Yrs
15	40- 49 Yrs
10	50- 59 Yrs
2	60- 69 Yrs
3	< 10 Yrs
9	70+ Yrs
3	70+ Yrs
2	< 10 Yrs
12	10- 19 Yrs
7	20- 29 Yrs
6	30- 39 Yrs
32	30- 39 Yrs
20	20- 29 Yrs
2	10- 19 Yrs
12	50- 59 Yrs
32	40- 49 Yrs
9	10- 19 Yrs
7	30- 39 Yrs
27	30- 39 Yrs
30	50- 59 Yrs
10	20- 29 Yrs
6	10- 19 Yrs
9	20- 29 Yrs
5	60- 69 Yrs
11	40- 49 Yrs
24	50- 59 Yrs
6	< 10 Yrs
26	20- 29 Yrs
22	50- 59 Yrs
11	20- 29 Yrs
8	10- 19 Yrs
2	70+ Yrs
11	60- 69 Yrs
3	70+ Yrs

3	10- 19 Yrs
4	< 10 Yrs
12	60- 69 Yrs
13	30- 39 Yrs
17	40- 49 Yrs
29	40- 49 Yrs
17	30- 39 Yrs
2	10- 19 Yrs
25	30- 39 Yrs
5	20- 29 Yrs
10	20- 29 Yrs
20	30- 39 Yrs
3	10- 19 Yrs
2	< 10 Yrs
32	50- 59 Yrs
36	40- 49 Yrs
31	50- 59 Yrs
27	40- 49 Yrs
6	70+ Yrs
7	60- 69 Yrs
2	60- 69 Yrs
4	60- 69 Yrs
4	50- 59 Yrs
7	60- 69 Yrs
2	30- 39 Yrs
4	40- 49 Yrs
6	50- 59 Yrs
2	10- 19 Yrs
3	70+ Yrs
12	< 10 Yrs
26	< 10 Yrs
7	50- 59 Yrs
10	50- 59 Yrs
3	40- 49 Yrs
6	40- 49 Yrs
4	< 10 Yrs
9	< 10 Yrs
7	60- 69 Yrs
6	60- 69 Yrs
5	70+ Yrs
14	70+ Yrs
5	30- 39 Yrs
3	20- 29 Yrs
4	20- 29 Yrs
3	40- 49 Yrs
8	40- 49 Yrs
4	50- 59 Yrs
14	50- 59 Yrs
3	20- 29 Yrs
2	20- 29 Yrs

4	30- 39 Yrs
5	70+ Yrs
7	70+ Yrs
10	< 10 Yrs
23	< 10 Yrs
2	10- 19 Yrs
15	60- 69 Yrs
3	40- 49 Yrs
9	40- 49 Yrs
5	50- 59 Yrs
3	50- 59 Yrs
4	< 10 Yrs
5	< 10 Yrs
3	60- 69 Yrs
3	70+ Yrs
3	60- 69 Yrs
2	20- 29 Yrs
2	10- 19 Yrs
2	10- 19 Yrs
2	40- 49 Yrs
4	60- 69 Yrs
3	50- 59 Yrs
11	< 10 Yrs
10	70+ Yrs
6	30- 39 Yrs
6	30- 39 Yrs
10	40- 49 Yrs
7	< 10 Yrs
3	50- 59 Yrs
10	60- 69 Yrs
3	30- 39 Yrs
3	70+ Yrs
5	40- 49 Yrs
2	10- 19 Yrs
2	20- 29 Yrs
12	< 10 Yrs
6	30- 39 Yrs
2	70+ Yrs
4	60- 69 Yrs
14	< 10 Yrs
3	50- 59 Yrs
8	60- 69 Yrs
10	40- 49 Yrs
22	50- 59 Yrs
8	60- 69 Yrs
16	50- 59 Yrs
6	30- 39 Yrs
6	70+ Yrs
5	70+ Yrs
5	60- 69 Yrs

3	30- 39 Yrs
15	< 10 Yrs
8	< 10 Yrs
3	20- 29 Yrs
2	10- 19 Yrs
2	10- 19 Yrs
4	30- 39 Yrs
4	30- 39 Yrs
2	20- 29 Yrs
2	
3	
16	70+ Yrs
9	60- 69 Yrs
8	70+ Yrs
6	40- 49 Yrs
8	50- 59 Yrs
10	60- 69 Yrs
15	50- 59 Yrs
16	40- 49 Yrs
5	30- 39 Yrs
7	40- 49 Yrs
3	40- 49 Yrs
6	50- 59 Yrs
3	50- 59 Yrs
4	60- 69 Yrs
5	60- 69 Yrs
5	70+ Yrs
5	70+ Yrs
2	10- 19 Yrs
2	10- 19 Yrs
14	40- 49 Yrs
5	40- 49 Yrs
3	20- 29 Yrs
4	30- 39 Yrs
2	20- 29 Yrs
9	30- 39 Yrs
7	70+ Yrs
14	70+ Yrs
8	60- 69 Yrs
8	50- 59 Yrs
9	60- 69 Yrs
2	
3	
13	50- 59 Yrs
2	
7	30- 39 Yrs
12	70+ Yrs
3	30- 39 Yrs
2	10- 19 Yrs
2	20- 29 Yrs

3	
2	10- 19 Yrs
5	40- 49 Yrs
7	60- 69 Yrs
6	50- 59 Yrs
8	60- 69 Yrs
11	50- 59 Yrs
6	70+ Yrs
12	40- 49 Yrs
10	30- 39 Yrs
7	30- 39 Yrs
11	40- 49 Yrs
17	40- 49 Yrs
2	20- 29 Yrs
2	10- 19 Yrs
2	
10	60- 69 Yrs
10	70+ Yrs
9	50- 59 Yrs
12	50- 59 Yrs
7	70+ Yrs
8	60- 69 Yrs
3	20- 29 Yrs
18	50- 59 Yrs
10	50- 59 Yrs
18	40- 49 Yrs
7	40- 49 Yrs
3	
9	70+ Yrs
19	70+ Yrs
4	
12	60- 69 Yrs
11	60- 69 Yrs
2	10- 19 Yrs
3	10- 19 Yrs
5	30- 39 Yrs
5	30- 39 Yrs
4	20- 29 Yrs
7	50- 59 Yrs
3	40- 49 Yrs
3	30- 39 Yrs
2	10- 19 Yrs
2	20- 29 Yrs
2	20- 29 Yrs
5	40- 49 Yrs
3	30- 39 Yrs
12	60- 69 Yrs
2	
13	70+ Yrs
6	70+ Yrs

12	50- 59 Yrs
3	
7	60- 69 Yrs
3	30- 39 Yrs
10	50- 59 Yrs
4	70+ Yrs
3	40- 49 Yrs
4	70+ Yrs
8	50- 59 Yrs
4	60- 69 Yrs
7	30- 39 Yrs
4	60- 69 Yrs
7	40- 49 Yrs
2	20- 29 Yrs
5	70+ Yrs
2	
6	60- 69 Yrs
15	40- 49 Yrs
12	60- 69 Yrs
8	50- 59 Yrs
4	30- 39 Yrs
8	30- 39 Yrs
5	70+ Yrs
15	50- 59 Yrs
8	40- 49 Yrs
16	50- 59 Yrs
11	50- 59 Yrs
11	60- 69 Yrs
7	70+ Yrs
7	70+ Yrs
4	
13	60- 69 Yrs
8	30- 39 Yrs
3	20- 29 Yrs
5	40- 49 Yrs
14	40- 49 Yrs
4	30- 39 Yrs
4	40- 49 Yrs
4	70+ Yrs
4	50- 59 Yrs
9	60- 69 Yrs
5	40- 49 Yrs
13	60- 69 Yrs
11	70+ Yrs
18	70+ Yrs
4	70+ Yrs
6	70+ Yrs
7	50- 59 Yrs
6	70+ Yrs
4	70+ Yrs

6	60- 69 Yrs
5	40- 49 Yrs
7	60- 69 Yrs
6	70+ Yrs
5	60- 69 Yrs
8	70+ Yrs
40	40- 49 Yrs
4	40- 49 Yrs
21	40- 49 Yrs
4	40- 49 Yrs
7	50- 59 Yrs
8	70+ Yrs
37	50- 59 Yrs
9	60- 69 Yrs
6	60- 69 Yrs
7	60- 69 Yrs
4	50- 59 Yrs
19	70+ Yrs
17	70+ Yrs
8	70+ Yrs
20	50- 59 Yrs
17	60- 69 Yrs
46	60- 69 Yrs
9	70+ Yrs
4	70+ Yrs
5	70+ Yrs
4	60- 69 Yrs
14	50- 59 Yrs
4	70+ Yrs
12	60- 69 Yrs
5	60- 69 Yrs
6	50- 59 Yrs
11	70+ Yrs
5	30- 39 Yrs
7	70+ Yrs
7	30- 39 Yrs
8	40- 49 Yrs
9	40- 49 Yrs
13	50- 59 Yrs
11	50- 59 Yrs
20	60- 69 Yrs
17	60- 69 Yrs
4	70+ Yrs
6	70+ Yrs
11	70+ Yrs
5	70+ Yrs
4	40- 49 Yrs
16	70+ Yrs
6	50- 59 Yrs
11	70+ Yrs

4	40- 49 Yrs
5	60- 69 Yrs
8	60- 69 Yrs
6	60- 69 Yrs
5	70+ Yrs
13	70+ Yrs
6	40- 49 Yrs
5	60- 69 Yrs
6	50- 59 Yrs
8	60- 69 Yrs
14	70+ Yrs
4	50- 59 Yrs
9	70+ Yrs
7	70+ Yrs
4	50- 59 Yrs
8	60- 69 Yrs
6	70+ Yrs
4	60- 69 Yrs
4	70+ Yrs
11	70+ Yrs
6	70+ Yrs
14	70+ Yrs
7	50- 59 Yrs
6	60- 69 Yrs
5	40- 49 Yrs
10	50- 59 Yrs
6	50- 59 Yrs
20	40- 49 Yrs
4	60- 69 Yrs
11	60- 69 Yrs
10	30- 39 Yrs
4	60- 69 Yrs
12	70+ Yrs
5	70+ Yrs
11	60- 69 Yrs
4	50- 59 Yrs
8	60- 69 Yrs
4	60- 69 Yrs
11	70+ Yrs
4	70+ Yrs
7	70+ Yrs
20	70+ Yrs
31	60- 69 Yrs
36	60- 69 Yrs
19	50- 59 Yrs
27	50- 59 Yrs
12	40- 49 Yrs
11	70+ Yrs
7	30- 39 Yrs
6	30- 39 Yrs

10	40- 49 Yrs
14	40- 49 Yrs
9	70+ Yrs
4	50- 59 Yrs
6	60- 69 Yrs
13	70+ Yrs
5	70+ Yrs
5	70+ Yrs
4	50- 59 Yrs
18	60- 69 Yrs
15	50- 59 Yrs
34	40- 49 Yrs
20	50- 59 Yrs
20	40- 49 Yrs
10	60- 69 Yrs
9	70+ Yrs
6	60- 69 Yrs
11	70+ Yrs
4	30- 39 Yrs
15	70+ Yrs
6	60- 69 Yrs
4	70+ Yrs
16	70+ Yrs
5	50- 59 Yrs
9	70+ Yrs
10	60- 69 Yrs
4	50- 59 Yrs
4	60- 69 Yrs
5	30- 39 Yrs
11	70+ Yrs
24	70+ Yrs
8	70+ Yrs
6	70+ Yrs
6	50- 59 Yrs
4	60- 69 Yrs
15	50- 59 Yrs
16	40- 49 Yrs
5	40- 49 Yrs
8	70+ Yrs
6	50- 59 Yrs
19	70+ Yrs
6	60- 69 Yrs
6	70+ Yrs
19	70+ Yrs
11	50- 59 Yrs
5	70+ Yrs
7	40- 49 Yrs
10	50- 59 Yrs
4	30- 39 Yrs
5	60- 69 Yrs

7	70+ Yrs
6	60- 69 Yrs
7	60- 69 Yrs
8	40- 49 Yrs
7	50- 59 Yrs
4	50- 59 Yrs
5	40- 49 Yrs
6	60- 69 Yrs
5	70+ Yrs
6	70+ Yrs
4	50- 59 Yrs
16	70+ Yrs
8	70+ Yrs
16	60- 69 Yrs
8	30- 39 Yrs
7	30- 39 Yrs
8	40- 49 Yrs
5	10- 19 Yrs
7	70+ Yrs
4	50- 59 Yrs
4	50- 59 Yrs
19	70+ Yrs
11	60- 69 Yrs
5	50- 59 Yrs
4	60- 69 Yrs
4	70+ Yrs
5	70+ Yrs
4	60- 69 Yrs
5	70+ Yrs
12	60- 69 Yrs
5	60- 69 Yrs
6	50- 59 Yrs
11	60- 69 Yrs
11	70+ Yrs
11	70+ Yrs
9	50- 59 Yrs
7	70+ Yrs
4	70+ Yrs
12	60- 69 Yrs
10	50- 59 Yrs
7	40- 49 Yrs
5	30- 39 Yrs
10	20- 29 Yrs
4	60- 69 Yrs
13	70+ Yrs
6	70+ Yrs
6	30- 39 Yrs
12	50- 59 Yrs
12	40- 49 Yrs
13	40- 49 Yrs

5	60- 69 Yrs
5	40- 49 Yrs
11	50- 59 Yrs
5	20- 29 Yrs
10	60- 69 Yrs
13	50- 59 Yrs
12	30- 39 Yrs
6	70+ Yrs
6	70+ Yrs
3	60- 69 Yrs
3	30- 39 Yrs
3	60- 69 Yrs
3	70+ Yrs
3	60- 69 Yrs
3	60- 69 Yrs
3	50- 59 Yrs
3	70+ Yrs
3	70+ Yrs
3	70+ Yrs
3	50- 59 Yrs
3	40- 49 Yrs
3	40- 49 Yrs
3	50- 59 Yrs
3	30- 39 Yrs
3	70+ Yrs
3	70+ Yrs
3	60- 69 Yrs
3	50- 59 Yrs
3	30- 39 Yrs
3	60- 69 Yrs
3	40- 49 Yrs
3	60- 69 Yrs
3	30- 39 Yrs
3	70+ Yrs
3	60- 69 Yrs
3	50- 59 Yrs
3	60- 69 Yrs
3	50- 59 Yrs
3	60- 69 Yrs
3	40- 49 Yrs
3	70+ Yrs
3	60- 69 Yrs
3	60- 69 Yrs
3	60- 69 Yrs
3	70+ Yrs
3	70+ Yrs
3	70+ Yrs
3	60- 69 Yrs
3	60- 69 Yrs
3	40- 49 Yrs

3	40- 49 Yrs
3	40- 49 Yrs
3	70+ Yrs
3	60- 69 Yrs
3	60- 69 Yrs
3	60- 69 Yrs
3	50- 59 Yrs
3	30- 39 Yrs
3	50- 59 Yrs
3	40- 49 Yrs
3	30- 39 Yrs
3	40- 49 Yrs
3	60- 69 Yrs
3	60- 69 Yrs
3	70+ Yrs
3	60- 69 Yrs
3	50- 59 Yrs
3	30- 39 Yrs
2	50- 59 Yrs
2	60- 69 Yrs
2	50- 59 Yrs
2	30- 39 Yrs
2	70+ Yrs
2	60- 69 Yrs
2	70+ Yrs
2	30- 39 Yrs
2	50- 59 Yrs
2	50- 59 Yrs
2	40- 49 Yrs
2	50- 59 Yrs
2	60- 69 Yrs
2	40- 49 Yrs
2	70+ Yrs
2	50- 59 Yrs
2	60- 69 Yrs
2	70+ Yrs
2	40- 49 Yrs
2	30- 39 Yrs
2	40- 49 Yrs
2	70+ Yrs
2	60- 69 Yrs
2	60- 69 Yrs
2	60- 69 Yrs
2	50- 59 Yrs
2	70+ Yrs
2	70+ Yrs
2	60- 69 Yrs
2	30- 39 Yrs
2	50- 59 Yrs
2	40- 49 Yrs

2	40- 49 Yrs
2	70+ Yrs
2	50- 59 Yrs
2	40- 49 Yrs
2	70+ Yrs
2	60- 69 Yrs
2	40- 49 Yrs
2	50- 59 Yrs
2	20- 29 Yrs
2	60- 69 Yrs
2	50- 59 Yrs
2	50- 59 Yrs
2	30- 39 Yrs
2	70+ Yrs
2	50- 59 Yrs
2	70+ Yrs
2	50- 59 Yrs
2	60- 69 Yrs
2	60- 69 Yrs
2	60- 69 Yrs
2	70+ Yrs
2	30- 39 Yrs
2	50- 59 Yrs
2	60- 69 Yrs
2	50- 59 Yrs
2	60- 69 Yrs
2	60- 69 Yrs
2	70+ Yrs
2	30- 39 Yrs
2	70+ Yrs
2	60- 69 Yrs
2	60- 69 Yrs
2	60- 69 Yrs
2	20- 29 Yrs
2	70+ Yrs
2	60- 69 Yrs
2	70+ Yrs
2	50- 59 Yrs
2	70+ Yrs
2	40- 49 Yrs
2	70+ Yrs
2	70+ Yrs
2	70+ Yrs
2	60- 69 Yrs
10	10- 19 Yrs
10	10- 19 Yrs
9	10- 19 Yrs
8	10- 19 Yrs
6	10- 19 Yrs
4	10- 19 Yrs

4	10- 19 Yrs
5	10- 19 Yrs
12	10- 19 Yrs
4	10- 19 Yrs
8	10- 19 Yrs
17	10- 19 Yrs
4	10- 19 Yrs
4	10- 19 Yrs
6	10- 19 Yrs
5	10- 19 Yrs
4	< 10 Yrs
4	10- 19 Yrs
10	10- 19 Yrs
6	10- 19 Yrs
5	10- 19 Yrs
10	10- 19 Yrs
7	< 10 Yrs
4	< 10 Yrs
9	10- 19 Yrs
8	< 10 Yrs
4	< 10 Yrs
19	10- 19 Yrs
5	< 10 Yrs
8	10- 19 Yrs
5	10- 19 Yrs
4	10- 19 Yrs
4	< 10 Yrs
6	10- 19 Yrs
10	10- 19 Yrs
6	10- 19 Yrs
4	10- 19 Yrs
8	10- 19 Yrs
4	10- 19 Yrs
8	10- 19 Yrs
9	10- 19 Yrs
6	10- 19 Yrs
4	< 10 Yrs
4	10- 19 Yrs
6	20- 29 Yrs
7	20- 29 Yrs
41	20- 29 Yrs
23	20- 29 Yrs
16	20- 29 Yrs
5	20- 29 Yrs
11	20- 29 Yrs
8	20- 29 Yrs
7	20- 29 Yrs
4	20- 29 Yrs
4	20- 29 Yrs
4	20- 29 Yrs

20	20- 29 Yrs
10	20- 29 Yrs
5	20- 29 Yrs
16	20- 29 Yrs
7	20- 29 Yrs
6	20- 29 Yrs
10	20- 29 Yrs
26	20- 29 Yrs
20	20- 29 Yrs
13	20- 29 Yrs
11	20- 29 Yrs
26	20- 29 Yrs
20	20- 29 Yrs
6	20- 29 Yrs
20	20- 29 Yrs
14	20- 29 Yrs
4	20- 29 Yrs
4	20- 29 Yrs
4	20- 29 Yrs
4	20- 29 Yrs
68	20- 29 Yrs
6	20- 29 Yrs
53	20- 29 Yrs
13	20- 29 Yrs
7	20- 29 Yrs
5	20- 29 Yrs
17	20- 29 Yrs
10	20- 29 Yrs
56	20- 29 Yrs
22	20- 29 Yrs
12	20- 29 Yrs
48	20- 29 Yrs
8	20- 29 Yrs
24	20- 29 Yrs
20	20- 29 Yrs
11	20- 29 Yrs
26	20- 29 Yrs
5	20- 29 Yrs
4	20- 29 Yrs
4	20- 29 Yrs
5	20- 29 Yrs
20	20- 29 Yrs
10	20- 29 Yrs
4	30- 39 Yrs
8	30- 39 Yrs
4	30- 39 Yrs
8	30- 39 Yrs
10	30- 39 Yrs
8	30- 39 Yrs
15	30- 39 Yrs

4	30- 39 Yrs
11	30- 39 Yrs
50	30- 39 Yrs
62	30- 39 Yrs
14	30- 39 Yrs
32	30- 39 Yrs
58	30- 39 Yrs
79	30- 39 Yrs
5	30- 39 Yrs
4	30- 39 Yrs
9	30- 39 Yrs
27	30- 39 Yrs
14	30- 39 Yrs
47	30- 39 Yrs
4	30- 39 Yrs
29	30- 39 Yrs
4	30- 39 Yrs
4	30- 39 Yrs
7	30- 39 Yrs
12	30- 39 Yrs
12	30- 39 Yrs
26	30- 39 Yrs
18	30- 39 Yrs
7	30- 39 Yrs
8	30- 39 Yrs
9	30- 39 Yrs
12	30- 39 Yrs
18	30- 39 Yrs
7	30- 39 Yrs
4	30- 39 Yrs
4	30- 39 Yrs
15	30- 39 Yrs
13	30- 39 Yrs
5	30- 39 Yrs
25	30- 39 Yrs
7	30- 39 Yrs
8	30- 39 Yrs
7	30- 39 Yrs
23	30- 39 Yrs
6	30- 39 Yrs
30	30- 39 Yrs
5	30- 39 Yrs
26	30- 39 Yrs
12	30- 39 Yrs
4	30- 39 Yrs
73	30- 39 Yrs
82	30- 39 Yrs
6	30- 39 Yrs
8	30- 39 Yrs
37	30- 39 Yrs

5	30- 39 Yrs
21	30- 39 Yrs
31	30- 39 Yrs
73	30- 39 Yrs
7	30- 39 Yrs
82	30- 39 Yrs
10	30- 39 Yrs
4	30- 39 Yrs
4	30- 39 Yrs
18	30- 39 Yrs
32	30- 39 Yrs
13	30- 39 Yrs
31	30- 39 Yrs
6	30- 39 Yrs
4	30- 39 Yrs
5	30- 39 Yrs
5	30- 39 Yrs
55	30- 39 Yrs
5	30- 39 Yrs
46	30- 39 Yrs
18	30- 39 Yrs
4	30- 39 Yrs
6	30- 39 Yrs
9	30- 39 Yrs
5	30- 39 Yrs
35	30- 39 Yrs
5	30- 39 Yrs
20	30- 39 Yrs
7	30- 39 Yrs
7	30- 39 Yrs
71	30- 39 Yrs
11	30- 39 Yrs
6	30- 39 Yrs
26	30- 39 Yrs
13	30- 39 Yrs
24	30- 39 Yrs
6	30- 39 Yrs
62	30- 39 Yrs
48	30- 39 Yrs
13	30- 39 Yrs
53	30- 39 Yrs
17	30- 39 Yrs
10	30- 39 Yrs
5	30- 39 Yrs
8	30- 39 Yrs
4	30- 39 Yrs
17	30- 39 Yrs
4	30- 39 Yrs
26	30- 39 Yrs
11	30- 39 Yrs

5	30- 39 Yrs
102	30- 39 Yrs
5	30- 39 Yrs
51	30- 39 Yrs
4	30- 39 Yrs
114	30- 39 Yrs
5	30- 39 Yrs
9	30- 39 Yrs
5	30- 39 Yrs
10	30- 39 Yrs
8	30- 39 Yrs
21	30- 39 Yrs
10	30- 39 Yrs
22	40- 49 Yrs
6	40- 49 Yrs
15	40- 49 Yrs
4	40- 49 Yrs
16	40- 49 Yrs
7	40- 49 Yrs
20	40- 49 Yrs
4	40- 49 Yrs
68	40- 49 Yrs
45	40- 49 Yrs
18	40- 49 Yrs
9	40- 49 Yrs
102	40- 49 Yrs
125	40- 49 Yrs
5	40- 49 Yrs
5	40- 49 Yrs
53	40- 49 Yrs
20	40- 49 Yrs
16	40- 49 Yrs
17	40- 49 Yrs
36	40- 49 Yrs
6	40- 49 Yrs
127	40- 49 Yrs
131	40- 49 Yrs
8	40- 49 Yrs
7	40- 49 Yrs
4	40- 49 Yrs
10	40- 49 Yrs
16	40- 49 Yrs
5	40- 49 Yrs
6	40- 49 Yrs
25	40- 49 Yrs
5	40- 49 Yrs
4	40- 49 Yrs
32	40- 49 Yrs
31	40- 49 Yrs
7	40- 49 Yrs

5	40- 49 Yrs
13	40- 49 Yrs
6	40- 49 Yrs
6	40- 49 Yrs
29	40- 49 Yrs
13	40- 49 Yrs
27	40- 49 Yrs
4	40- 49 Yrs
27	40- 49 Yrs
16	40- 49 Yrs
42	40- 49 Yrs
4	40- 49 Yrs
4	40- 49 Yrs
4	40- 49 Yrs
5	40- 49 Yrs
13	40- 49 Yrs
5	40- 49 Yrs
5	40- 49 Yrs
24	40- 49 Yrs
11	40- 49 Yrs
8	40- 49 Yrs
24	40- 49 Yrs
7	40- 49 Yrs
5	40- 49 Yrs
5	40- 49 Yrs
32	40- 49 Yrs
4	40- 49 Yrs
31	40- 49 Yrs
69	40- 49 Yrs
75	40- 49 Yrs
4	40- 49 Yrs
4	40- 49 Yrs
25	40- 49 Yrs
6	40- 49 Yrs
9	40- 49 Yrs
33	40- 49 Yrs
47	40- 49 Yrs
5	40- 49 Yrs
11	40- 49 Yrs
5	40- 49 Yrs
75	40- 49 Yrs
8	40- 49 Yrs
18	40- 49 Yrs
69	40- 49 Yrs
70	40- 49 Yrs
40	40- 49 Yrs
16	40- 49 Yrs
6	40- 49 Yrs
21	40- 49 Yrs
35	40- 49 Yrs

25	40- 49 Yrs
12	40- 49 Yrs
5	40- 49 Yrs
16	40- 49 Yrs
6	40- 49 Yrs
7	40- 49 Yrs
30	40- 49 Yrs
21	40- 49 Yrs
4	40- 49 Yrs
6	40- 49 Yrs
27	40- 49 Yrs
36	40- 49 Yrs
12	40- 49 Yrs
15	40- 49 Yrs
32	40- 49 Yrs
42	40- 49 Yrs
15	40- 49 Yrs
8	40- 49 Yrs
5	40- 49 Yrs
15	40- 49 Yrs
9	40- 49 Yrs
7	40- 49 Yrs
36	40- 49 Yrs
9	40- 49 Yrs
29	40- 49 Yrs
11	40- 49 Yrs
14	40- 49 Yrs
91	40- 49 Yrs
6	40- 49 Yrs
4	40- 49 Yrs
10	40- 49 Yrs
87	40- 49 Yrs
35	40- 49 Yrs
82	40- 49 Yrs
77	40- 49 Yrs
20	40- 49 Yrs
30	40- 49 Yrs
10	40- 49 Yrs
17	40- 49 Yrs
5	40- 49 Yrs
13	40- 49 Yrs
17	40- 49 Yrs
4	40- 49 Yrs
4	40- 49 Yrs
7	40- 49 Yrs
33	40- 49 Yrs
14	40- 49 Yrs
9	40- 49 Yrs
16	40- 49 Yrs
32	40- 49 Yrs

87	40- 49 Yrs
9	40- 49 Yrs
132	40- 49 Yrs
4	40- 49 Yrs
6	40- 49 Yrs
5	40- 49 Yrs
29	40- 49 Yrs
20	40- 49 Yrs
17	40- 49 Yrs
4	40- 49 Yrs
34	40- 49 Yrs
17	40- 49 Yrs
10	40- 49 Yrs
6	40- 49 Yrs
18	70+ Yrs
11	70+ Yrs
6	70+ Yrs
5	70+ Yrs
8	70+ Yrs
16	70+ Yrs
4	70+ Yrs
11	70+ Yrs
8	70+ Yrs
8	70+ Yrs
5	70+ Yrs
8	70+ Yrs
8	70+ Yrs
5	70+ Yrs
18	70+ Yrs
11	70+ Yrs
42	70+ Yrs
7	70+ Yrs
10	70+ Yrs
6	70+ Yrs
6	70+ Yrs
4	70+ Yrs
14	70+ Yrs
20	70+ Yrs
18	70+ Yrs
15	70+ Yrs
14	70+ Yrs
16	70+ Yrs
17	70+ Yrs
4	70+ Yrs
11	70+ Yrs
12	70+ Yrs
5	70+ Yrs
4	70+ Yrs
4	70+ Yrs
11	70+ Yrs

23	70+ Yrs
16	70+ Yrs
10	70+ Yrs
25	70+ Yrs
12	70+ Yrs
17	70+ Yrs
5	70+ Yrs
13	70+ Yrs
14	70+ Yrs
7	70+ Yrs
13	70+ Yrs
10	70+ Yrs
7	70+ Yrs
4	70+ Yrs
4	70+ Yrs
10	70+ Yrs
5	70+ Yrs
24	70+ Yrs
8	70+ Yrs
10	70+ Yrs
10	70+ Yrs
4	70+ Yrs
14	70+ Yrs
7	70+ Yrs
8	70+ Yrs
4	70+ Yrs
16	70+ Yrs
19	70+ Yrs
11	70+ Yrs
25	70+ Yrs
4	70+ Yrs
4	70+ Yrs
11	70+ Yrs
4	70+ Yrs
7	70+ Yrs
9	70+ Yrs
22	70+ Yrs
12	70+ Yrs
44	70+ Yrs
4	70+ Yrs
6	70+ Yrs
30	70+ Yrs
6	70+ Yrs
11	70+ Yrs
7	70+ Yrs
13	70+ Yrs
4	70+ Yrs
10	70+ Yrs
7	70+ Yrs
11	70+ Yrs

13	70+ Yrs
8	70+ Yrs
9	70+ Yrs
11	70+ Yrs
19	70+ Yrs
7	70+ Yrs
20	70+ Yrs
24	70+ Yrs
6	70+ Yrs
7	70+ Yrs
10	70+ Yrs
16	70+ Yrs
9	70+ Yrs
10	70+ Yrs
4	70+ Yrs
9	70+ Yrs
7	70+ Yrs
5	70+ Yrs
13	70+ Yrs
7	70+ Yrs
6	70+ Yrs
6	70+ Yrs
5	70+ Yrs
4	70+ Yrs
5	70+ Yrs
9	70+ Yrs
13	70+ Yrs
37	70+ Yrs
17	70+ Yrs
18	70+ Yrs
10	70+ Yrs
21	70+ Yrs
6	70+ Yrs
36	70+ Yrs
18	70+ Yrs
5	70+ Yrs
30	70+ Yrs
45	70+ Yrs
47	70+ Yrs
4	70+ Yrs
10	70+ Yrs
6	70+ Yrs
9	70+ Yrs
17	70+ Yrs
13	70+ Yrs
7	70+ Yrs
5	70+ Yrs
5	70+ Yrs
6	70+ Yrs
6	70+ Yrs

12	70+ Yrs
6	70+ Yrs
13	70+ Yrs
8	70+ Yrs
11	70+ Yrs
14	70+ Yrs
40	70+ Yrs
12	70+ Yrs
13	70+ Yrs
5	70+ Yrs
45	70+ Yrs
4	70+ Yrs
12	70+ Yrs
9	70+ Yrs
8	70+ Yrs
5	70+ Yrs
20	70+ Yrs
13	70+ Yrs
18	70+ Yrs
33	70+ Yrs
4	50- 59 Yrs
14	50- 59 Yrs
15	50- 59 Yrs
9	50- 59 Yrs
4	50- 59 Yrs
4	50- 59 Yrs
8	50- 59 Yrs
4	50- 59 Yrs
14	50- 59 Yrs
24	50- 59 Yrs
13	50- 59 Yrs
12	50- 59 Yrs
9	50- 59 Yrs
33	50- 59 Yrs
42	50- 59 Yrs
29	50- 59 Yrs
15	50- 59 Yrs
36	50- 59 Yrs
7	50- 59 Yrs
42	50- 59 Yrs
6	50- 59 Yrs
26	50- 59 Yrs
43	50- 59 Yrs
37	50- 59 Yrs
13	50- 59 Yrs
31	50- 59 Yrs
9	50- 59 Yrs
4	50- 59 Yrs
7	50- 59 Yrs
171	50- 59 Yrs

74	50- 59 Yrs
12	50- 59 Yrs
8	50- 59 Yrs
9	50- 59 Yrs
5	50- 59 Yrs
19	50- 59 Yrs
21	50- 59 Yrs
30	50- 59 Yrs
30	50- 59 Yrs
22	50- 59 Yrs
7	50- 59 Yrs
4	50- 59 Yrs
13	50- 59 Yrs
23	50- 59 Yrs
7	50- 59 Yrs
12	50- 59 Yrs
15	50- 59 Yrs
7	50- 59 Yrs
9	50- 59 Yrs
31	50- 59 Yrs
9	50- 59 Yrs
25	50- 59 Yrs
11	50- 59 Yrs
4	50- 59 Yrs
11	50- 59 Yrs
21	50- 59 Yrs
48	50- 59 Yrs
22	50- 59 Yrs
8	50- 59 Yrs
6	50- 59 Yrs
9	50- 59 Yrs
6	50- 59 Yrs
5	50- 59 Yrs
13	50- 59 Yrs
4	50- 59 Yrs
7	50- 59 Yrs
26	50- 59 Yrs
18	50- 59 Yrs
30	50- 59 Yrs
10	50- 59 Yrs
12	50- 59 Yrs
5	50- 59 Yrs
7	50- 59 Yrs
8	50- 59 Yrs
4	50- 59 Yrs
26	50- 59 Yrs
20	50- 59 Yrs
10	50- 59 Yrs
9	50- 59 Yrs
9	50- 59 Yrs

12	50- 59 Yrs
6	50- 59 Yrs
4	50- 59 Yrs
31	50- 59 Yrs
12	50- 59 Yrs
5	50- 59 Yrs
41	50- 59 Yrs
5	50- 59 Yrs
8	50- 59 Yrs
34	50- 59 Yrs
49	50- 59 Yrs
10	50- 59 Yrs
5	50- 59 Yrs
10	50- 59 Yrs
7	50- 59 Yrs
9	50- 59 Yrs
12	50- 59 Yrs
12	50- 59 Yrs
9	50- 59 Yrs
30	50- 59 Yrs
43	50- 59 Yrs
9	50- 59 Yrs
15	50- 59 Yrs
25	50- 59 Yrs
23	50- 59 Yrs
4	50- 59 Yrs
18	50- 59 Yrs
20	50- 59 Yrs
6	50- 59 Yrs
11	50- 59 Yrs
6	50- 59 Yrs
15	50- 59 Yrs
19	50- 59 Yrs
7	50- 59 Yrs
10	50- 59 Yrs
20	50- 59 Yrs
9	50- 59 Yrs
21	50- 59 Yrs
22	50- 59 Yrs
4	50- 59 Yrs
26	50- 59 Yrs
5	50- 59 Yrs
23	50- 59 Yrs
9	50- 59 Yrs
20	50- 59 Yrs
49	50- 59 Yrs
18	50- 59 Yrs
16	50- 59 Yrs
7	50- 59 Yrs
12	50- 59 Yrs

10	50- 59 Yrs
6	50- 59 Yrs
6	50- 59 Yrs
25	50- 59 Yrs
33	50- 59 Yrs
15	50- 59 Yrs
46	50- 59 Yrs
29	50- 59 Yrs
19	50- 59 Yrs
26	50- 59 Yrs
51	50- 59 Yrs
4	50- 59 Yrs
8	50- 59 Yrs
10	50- 59 Yrs
55	50- 59 Yrs
102	50- 59 Yrs
63	50- 59 Yrs
29	50- 59 Yrs
34	50- 59 Yrs
17	50- 59 Yrs
10	50- 59 Yrs
4	50- 59 Yrs
7	50- 59 Yrs
6	50- 59 Yrs
45	50- 59 Yrs
17	50- 59 Yrs
5	50- 59 Yrs
39	50- 59 Yrs
19	50- 59 Yrs
26	50- 59 Yrs
18	50- 59 Yrs
4	50- 59 Yrs
15	50- 59 Yrs
60	50- 59 Yrs
10	50- 59 Yrs
25	50- 59 Yrs
5	50- 59 Yrs
29	50- 59 Yrs
4	50- 59 Yrs
53	50- 59 Yrs
22	50- 59 Yrs
23	50- 59 Yrs
6	50- 59 Yrs
27	50- 59 Yrs
112	50- 59 Yrs
18	50- 59 Yrs
14	50- 59 Yrs
19	50- 59 Yrs
13	60- 69 Yrs
15	60- 69 Yrs

9	60- 69 Yrs
5	60- 69 Yrs
8	60- 69 Yrs
9	60- 69 Yrs
10	60- 69 Yrs
9	60- 69 Yrs
10	60- 69 Yrs
26	60- 69 Yrs
19	60- 69 Yrs
6	60- 69 Yrs
10	60- 69 Yrs
41	60- 69 Yrs
30	60- 69 Yrs
6	60- 69 Yrs
6	60- 69 Yrs
21	60- 69 Yrs
13	60- 69 Yrs
4	60- 69 Yrs
21	60- 69 Yrs
31	60- 69 Yrs
9	60- 69 Yrs
37	60- 69 Yrs
21	60- 69 Yrs
11	60- 69 Yrs
18	60- 69 Yrs
13	60- 69 Yrs
48	60- 69 Yrs
11	60- 69 Yrs
12	60- 69 Yrs
31	60- 69 Yrs
47	60- 69 Yrs
5	60- 69 Yrs
6	60- 69 Yrs
11	60- 69 Yrs
20	60- 69 Yrs
25	60- 69 Yrs
20	60- 69 Yrs
18	60- 69 Yrs
5	60- 69 Yrs
4	60- 69 Yrs
4	60- 69 Yrs
5	60- 69 Yrs
16	60- 69 Yrs
30	60- 69 Yrs
9	60- 69 Yrs
8	60- 69 Yrs
10	60- 69 Yrs
12	60- 69 Yrs
20	60- 69 Yrs
19	60- 69 Yrs

4	60- 69 Yrs
10	60- 69 Yrs
6	60- 69 Yrs
8	60- 69 Yrs
8	60- 69 Yrs
15	60- 69 Yrs
14	60- 69 Yrs
10	60- 69 Yrs
31	60- 69 Yrs
21	60- 69 Yrs
8	60- 69 Yrs
6	60- 69 Yrs
10	60- 69 Yrs
8	60- 69 Yrs
6	60- 69 Yrs
6	60- 69 Yrs
5	60- 69 Yrs
10	60- 69 Yrs
9	60- 69 Yrs
25	60- 69 Yrs
29	60- 69 Yrs
17	60- 69 Yrs
38	60- 69 Yrs
4	60- 69 Yrs
4	60- 69 Yrs
11	60- 69 Yrs
13	60- 69 Yrs
12	60- 69 Yrs
12	60- 69 Yrs
18	60- 69 Yrs
11	60- 69 Yrs
12	60- 69 Yrs
6	60- 69 Yrs
5	60- 69 Yrs
7	60- 69 Yrs
11	60- 69 Yrs
10	60- 69 Yrs
4	60- 69 Yrs
8	60- 69 Yrs
39	60- 69 Yrs
9	60- 69 Yrs
29	60- 69 Yrs
17	60- 69 Yrs
20	60- 69 Yrs
5	60- 69 Yrs
7	60- 69 Yrs
18	60- 69 Yrs
8	60- 69 Yrs
8	60- 69 Yrs
14	60- 69 Yrs

10	60- 69 Yrs
12	60- 69 Yrs
11	60- 69 Yrs
9	60- 69 Yrs
29	60- 69 Yrs
39	60- 69 Yrs
17	60- 69 Yrs
19	60- 69 Yrs
18	60- 69 Yrs
13	60- 69 Yrs
22	60- 69 Yrs
8	60- 69 Yrs
7	60- 69 Yrs
11	60- 69 Yrs
19	60- 69 Yrs
6	60- 69 Yrs
7	60- 69 Yrs
24	60- 69 Yrs
20	60- 69 Yrs
8	60- 69 Yrs
7	60- 69 Yrs
9	60- 69 Yrs
12	60- 69 Yrs
10	60- 69 Yrs
6	60- 69 Yrs
12	60- 69 Yrs
24	60- 69 Yrs
26	60- 69 Yrs
6	60- 69 Yrs
24	60- 69 Yrs
43	60- 69 Yrs
23	60- 69 Yrs
23	60- 69 Yrs
11	60- 69 Yrs
21	60- 69 Yrs
29	60- 69 Yrs
5	60- 69 Yrs
7	60- 69 Yrs
9	60- 69 Yrs
36	60- 69 Yrs
12	60- 69 Yrs
5	60- 69 Yrs
36	60- 69 Yrs
48	60- 69 Yrs
9	60- 69 Yrs
11	60- 69 Yrs
5	60- 69 Yrs
6	60- 69 Yrs
5	60- 69 Yrs
26	60- 69 Yrs

14	60- 69 Yrs
16	60- 69 Yrs
40	60- 69 Yrs
7	60- 69 Yrs
4	60- 69 Yrs
14	60- 69 Yrs
17	60- 69 Yrs
17	60- 69 Yrs
20	60- 69 Yrs
19	60- 69 Yrs
16	60- 69 Yrs
24	60- 69 Yrs
5	60- 69 Yrs
8	60- 69 Yrs
11	60- 69 Yrs
31	60- 69 Yrs
4	60- 69 Yrs
14	60- 69 Yrs
6	60- 69 Yrs
11	60- 69 Yrs
16	60- 69 Yrs
4	60- 69 Yrs
14	60- 69 Yrs
7	60- 69 Yrs
14	60- 69 Yrs
17	60- 69 Yrs
16	60- 69 Yrs
8	60- 69 Yrs
40	60- 69 Yrs
25	60- 69 Yrs
6	60- 69 Yrs
3	40- 49 Yrs
3	20- 29 Yrs
3	40- 49 Yrs
3	< 10 Yrs
3	40- 49 Yrs
3	60- 69 Yrs
3	60- 69 Yrs
3	60- 69 Yrs
3	70+ Yrs
3	30- 39 Yrs
3	60- 69 Yrs
3	50- 59 Yrs
3	40- 49 Yrs
3	10- 19 Yrs
3	50- 59 Yrs
3	40- 49 Yrs
3	20- 29 Yrs
3	60- 69 Yrs
3	30- 39 Yrs

3	50- 59 Yrs
3	40- 49 Yrs
3	10- 19 Yrs
3	70+ Yrs
3	60- 69 Yrs
3	40- 49 Yrs
3	30- 39 Yrs
3	50- 59 Yrs
3	60- 69 Yrs
3	70+ Yrs
3	70+ Yrs
3	20- 29 Yrs
3	60- 69 Yrs
3	20- 29 Yrs
3	10- 19 Yrs
3	70+ Yrs
3	< 10 Yrs
3	60- 69 Yrs
3	40- 49 Yrs
3	50- 59 Yrs
3	30- 39 Yrs
3	< 10 Yrs
3	40- 49 Yrs
3	20- 29 Yrs
3	20- 29 Yrs
3	30- 39 Yrs
3	30- 39 Yrs
3	50- 59 Yrs
3	70+ Yrs
3	60- 69 Yrs
3	20- 29 Yrs
3	20- 29 Yrs
3	30- 39 Yrs
3	40- 49 Yrs
3	50- 59 Yrs
3	20- 29 Yrs
3	40- 49 Yrs
3	40- 49 Yrs
3	30- 39 Yrs
3	20- 29 Yrs
3	60- 69 Yrs
3	< 10 Yrs
3	30- 39 Yrs
3	30- 39 Yrs
3	30- 39 Yrs
3	40- 49 Yrs
3	30- 39 Yrs
3	20- 29 Yrs
3	30- 39 Yrs
3	20- 29 Yrs

3	40- 49 Yrs
3	10- 19 Yrs
3	30- 39 Yrs
3	70+ Yrs
3	30- 39 Yrs
3	20- 29 Yrs
3	60- 69 Yrs
3	40- 49 Yrs
3	60- 69 Yrs
3	30- 39 Yrs
3	< 10 Yrs
3	20- 29 Yrs
3	20- 29 Yrs
3	50- 59 Yrs
3	10- 19 Yrs
3	60- 69 Yrs
3	60- 69 Yrs
3	30- 39 Yrs
3	20- 29 Yrs
3	60- 69 Yrs
3	30- 39 Yrs
3	70+ Yrs
3	70+ Yrs
3	50- 59 Yrs
3	30- 39 Yrs
3	40- 49 Yrs
3	40- 49 Yrs
3	40- 49 Yrs
3	< 10 Yrs
3	70+ Yrs
3	30- 39 Yrs
3	30- 39 Yrs
3	10- 19 Yrs
3	60- 69 Yrs
3	40- 49 Yrs
3	70+ Yrs
3	60- 69 Yrs
3	70+ Yrs
3	50- 59 Yrs
3	70+ Yrs
3	50- 59 Yrs
3	10- 19 Yrs
3	70+ Yrs
3	30- 39 Yrs
3	40- 49 Yrs
3	< 10 Yrs
3	20- 29 Yrs
3	70+ Yrs
3	30- 39 Yrs
3	20- 29 Yrs

3	< 10 Yrs
3	40- 49 Yrs
3	60- 69 Yrs
3	70+ Yrs
3	30- 39 Yrs
3	70+ Yrs
3	60- 69 Yrs
3	70+ Yrs
3	60- 69 Yrs
3	20- 29 Yrs
3	20- 29 Yrs
3	20- 29 Yrs
3	10- 19 Yrs
3	30- 39 Yrs
3	30- 39 Yrs
3	60- 69 Yrs
3	< 10 Yrs
3	70+ Yrs
3	20- 29 Yrs
3	20- 29 Yrs
3	50- 59 Yrs
2	20- 29 Yrs
2	20- 29 Yrs
2	20- 29 Yrs
2	10- 19 Yrs
2	10- 19 Yrs
2	40- 49 Yrs
2	< 10 Yrs
2	50- 59 Yrs
2	20- 29 Yrs
2	50- 59 Yrs
2	70+ Yrs
2	40- 49 Yrs
2	40- 49 Yrs
2	40- 49 Yrs
2	10- 19 Yrs
2	20- 29 Yrs
2	70+ Yrs
2	< 10 Yrs
2	20- 29 Yrs
2	50- 59 Yrs
2	10- 19 Yrs
2	20- 29 Yrs
2	70+ Yrs
2	20- 29 Yrs
2	20- 29 Yrs
2	60- 69 Yrs
2	50- 59 Yrs
2	30- 39 Yrs
2	30- 39 Yrs

2 60- 69 Yrs
2 60- 69 Yrs
2 50- 59 Yrs
2 40- 49 Yrs
2 < 10 Yrs
2 40- 49 Yrs
2 < 10 Yrs
2 60- 69 Yrs
2 10- 19 Yrs
2 40- 49 Yrs
2 30- 39 Yrs
2 < 10 Yrs
2 20- 29 Yrs
2 60- 69 Yrs
2 50- 59 Yrs
2 30- 39 Yrs
2 30- 39 Yrs
2 60- 69 Yrs
2 60- 69 Yrs
2 50- 59 Yrs
2 20- 29 Yrs
2 50- 59 Yrs
2 20- 29 Yrs
2 30- 39 Yrs
2 50- 59 Yrs
2 50- 59 Yrs
2 50- 59 Yrs
2 60- 69 Yrs
2 60- 69 Yrs
2 70+ Yrs
2 < 10 Yrs
2 70+ Yrs
2 70+ Yrs
2 60- 69 Yrs
2 30- 39 Yrs
2 40- 49 Yrs
2 20- 29 Yrs
2 < 10 Yrs
2 70+ Yrs
2 70+ Yrs
2 50- 59 Yrs
2 40- 49 Yrs
2 20- 29 Yrs
2 30- 39 Yrs
2 20- 29 Yrs
2 70+ Yrs
2 60- 69 Yrs
2 40- 49 Yrs
2 70+ Yrs
2 20- 29 Yrs

2 40- 49 Yrs
2 60- 69 Yrs
2 10- 19 Yrs
2 10- 19 Yrs
2 30- 39 Yrs
2 60- 69 Yrs
2 20- 29 Yrs
2 70+ Yrs
2 30- 39 Yrs
2 20- 29 Yrs
2 < 10 Yrs
2 20- 29 Yrs
2 50- 59 Yrs
2 40- 49 Yrs
2 10- 19 Yrs
2 40- 49 Yrs
2 70+ Yrs
2 70+ Yrs
2 70+ Yrs
2 30- 39 Yrs
2 40- 49 Yrs
2 40- 49 Yrs
2 40- 49 Yrs
2 < 10 Yrs
2 40- 49 Yrs
2 50- 59 Yrs
2 60- 69 Yrs
2 70+ Yrs
2 20- 29 Yrs
2 40- 49 Yrs
2 10- 19 Yrs
2 70+ Yrs
2 70+ Yrs
2 20- 29 Yrs
2 50- 59 Yrs
2 70+ Yrs
2 20- 29 Yrs
2 70+ Yrs
2 20- 29 Yrs
2 20- 29 Yrs
2 30- 39 Yrs
2 30- 39 Yrs
2 10- 19 Yrs
2 70+ Yrs
2 30- 39 Yrs
2 40- 49 Yrs
2 < 10 Yrs
2 70+ Yrs
2 30- 39 Yrs
2 20- 29 Yrs

2	10- 19 Yrs
2	30- 39 Yrs
2	60- 69 Yrs
2	40- 49 Yrs
2	60- 69 Yrs
2	20- 29 Yrs
2	< 10 Yrs
2	20- 29 Yrs
2	30- 39 Yrs
2	40- 49 Yrs
2	60- 69 Yrs
2	50- 59 Yrs
2	< 10 Yrs
2	20- 29 Yrs
2	10- 19 Yrs
2	30- 39 Yrs
2	10- 19 Yrs
2	10- 19 Yrs
2	20- 29 Yrs
2	30- 39 Yrs
2	40- 49 Yrs
2	30- 39 Yrs
2	30- 39 Yrs
2	50- 59 Yrs
2	10- 19 Yrs
2	60- 69 Yrs
2	50- 59 Yrs
2	40- 49 Yrs
2	30- 39 Yrs
2	20- 29 Yrs
2	< 10 Yrs
2	60- 69 Yrs
2	30- 39 Yrs
2	40- 49 Yrs
2	30- 39 Yrs
2	20- 29 Yrs
2	10- 19 Yrs
2	10- 19 Yrs
2	60- 69 Yrs
2	50- 59 Yrs
2	40- 49 Yrs
2	20- 29 Yrs
2	50- 59 Yrs
2	70+ Yrs
2	10- 19 Yrs
2	70+ Yrs
2	60- 69 Yrs
2	10- 19 Yrs
2	30- 39 Yrs
2	60- 69 Yrs

2	< 10 Yrs
2	60- 69 Yrs
2	20- 29 Yrs
2	40- 49 Yrs
2	70+ Yrs
2	10- 19 Yrs
2	< 10 Yrs
2	50- 59 Yrs
2	70+ Yrs
2	50- 59 Yrs
2	20- 29 Yrs
2	< 10 Yrs
2	60- 69 Yrs
2	40- 49 Yrs
2	< 10 Yrs
2	10- 19 Yrs
2	30- 39 Yrs
2	70+ Yrs
2	60- 69 Yrs
2	50- 59 Yrs
2	30- 39 Yrs
2	60- 69 Yrs
2	20- 29 Yrs
2	40- 49 Yrs
2	30- 39 Yrs
2	40- 49 Yrs
2	40- 49 Yrs
2	70+ Yrs
2	60- 69 Yrs
2	70+ Yrs
2	60- 69 Yrs
2	60- 69 Yrs
2	40- 49 Yrs
2	40- 49 Yrs
2	< 10 Yrs
2	70+ Yrs
2	70+ Yrs
2	10- 19 Yrs
2	10- 19 Yrs
2	20- 29 Yrs
2	60- 69 Yrs
2	70+ Yrs
2	70+ Yrs
1	60- 69 Yrs
1	70+ Yrs
1	70+ Yrs
1	60- 69 Yrs
1	30- 39 Yrs
1	< 10 Yrs
1	50- 59 Yrs

1	30- 39 Yrs
1	30- 39 Yrs
1	50- 59 Yrs
1	50- 59 Yrs
1	40- 49 Yrs
1	60- 69 Yrs
1	50- 59 Yrs
1	40- 49 Yrs
1	40- 49 Yrs
1	30- 39 Yrs
1	70+ Yrs
1	< 10 Yrs
1	30- 39 Yrs
1	20- 29 Yrs
1	20- 29 Yrs
1	40- 49 Yrs
1	60- 69 Yrs
1	10- 19 Yrs
1	40- 49 Yrs
1	10- 19 Yrs
1	30- 39 Yrs
1	70+ Yrs
1	40- 49 Yrs
1	50- 59 Yrs
1	50- 59 Yrs
1	20- 29 Yrs
1	40- 49 Yrs
1	60- 69 Yrs
1	20- 29 Yrs
1	50- 59 Yrs
1	40- 49 Yrs
1	< 10 Yrs
1	30- 39 Yrs
1	70+ Yrs
1	70+ Yrs
1	< 10 Yrs
1	20- 29 Yrs
1	40- 49 Yrs
1	20- 29 Yrs
1	40- 49 Yrs
1	20- 29 Yrs
1	10- 19 Yrs
1	70+ Yrs
1	40- 49 Yrs
1	30- 39 Yrs
1	40- 49 Yrs
1	70+ Yrs
1	10- 19 Yrs
1	10- 19 Yrs
1	30- 39 Yrs

1 10- 19 Yrs
1 40- 49 Yrs
1 < 10 Yrs
1 60- 69 Yrs
1 40- 49 Yrs
1 70+ Yrs
1 50- 59 Yrs
1 30- 39 Yrs
1 10- 19 Yrs
1 20- 29 Yrs
1 20- 29 Yrs
1 50- 59 Yrs
1 60- 69 Yrs
1 10- 19 Yrs
1 10- 19 Yrs
1 10- 19 Yrs
1 20- 29 Yrs
1 50- 59 Yrs
1 10- 19 Yrs
1 < 10 Yrs
1 10- 19 Yrs
1 20- 29 Yrs
1 20- 29 Yrs
1 60- 69 Yrs
1 50- 59 Yrs
1 40- 49 Yrs
1 40- 49 Yrs
1 40- 49 Yrs
1 50- 59 Yrs
1 40- 49 Yrs
1 50- 59 Yrs
1 60- 69 Yrs
1 40- 49 Yrs
1 40- 49 Yrs
1 50- 59 Yrs
1 50- 59 Yrs
1 70+ Yrs
1 30- 39 Yrs
1 10- 19 Yrs
1 10- 19 Yrs
1 20- 29 Yrs
1 40- 49 Yrs
1 40- 49 Yrs
1 60- 69 Yrs
1 50- 59 Yrs
1 10- 19 Yrs
1 50- 59 Yrs
1 10- 19 Yrs
1 < 10 Yrs
1 < 10 Yrs

1	30- 39 Yrs
1	10- 19 Yrs
1	< 10 Yrs
1	20- 29 Yrs
1	< 10 Yrs
1	70+ Yrs
1	40- 49 Yrs
1	30- 39 Yrs
1	70+ Yrs
1	10- 19 Yrs
1	< 10 Yrs
1	70+ Yrs
1	< 10 Yrs
1	50- 59 Yrs
1	70+ Yrs
1	60- 69 Yrs
1	40- 49 Yrs
1	50- 59 Yrs
1	20- 29 Yrs
1	30- 39 Yrs
1	60- 69 Yrs
1	20- 29 Yrs
1	60- 69 Yrs
1	< 10 Yrs
1	< 10 Yrs
1	70+ Yrs
1	40- 49 Yrs
1	50- 59 Yrs
1	50- 59 Yrs
1	60- 69 Yrs
1	50- 59 Yrs
1	40- 49 Yrs
1	30- 39 Yrs
1	30- 39 Yrs
1	< 10 Yrs
1	40- 49 Yrs
1	60- 69 Yrs
1	50- 59 Yrs
1	10- 19 Yrs
1	20- 29 Yrs
1	20- 29 Yrs
1	50- 59 Yrs
1	60- 69 Yrs
1	< 10 Yrs
1	20- 29 Yrs
1	20- 29 Yrs
1	10- 19 Yrs
1	30- 39 Yrs
1	40- 49 Yrs
1	50- 59 Yrs

1	40- 49 Yrs
1	40- 49 Yrs
1	20- 29 Yrs
1	40- 49 Yrs
1	60- 69 Yrs
1	30- 39 Yrs
1	60- 69 Yrs
1	50- 59 Yrs
1	< 10 Yrs
1	30- 39 Yrs
1	50- 59 Yrs
1	20- 29 Yrs
1	20- 29 Yrs
1	10- 19 Yrs
1	70+ Yrs
1	40- 49 Yrs
1	30- 39 Yrs
1	40- 49 Yrs
1	40- 49 Yrs
1	40- 49 Yrs
1	40- 49 Yrs
1	50- 59 Yrs
1	60- 69 Yrs
1	50- 59 Yrs
1	20- 29 Yrs
1	50- 59 Yrs
1	60- 69 Yrs
1	60- 69 Yrs
1	10- 19 Yrs
1	20- 29 Yrs
1	40- 49 Yrs
1	20- 29 Yrs
1	< 10 Yrs
1	< 10 Yrs
1	70+ Yrs
1	10- 19 Yrs
1	70+ Yrs
1	60- 69 Yrs
1	10- 19 Yrs
1	70+ Yrs
1	50- 59 Yrs
1	< 10 Yrs
1	< 10 Yrs
1	60- 69 Yrs
1	60- 69 Yrs
1	10- 19 Yrs
1	30- 39 Yrs
1	50- 59 Yrs
1	10- 19 Yrs
1	30- 39 Yrs

1	40- 49 Yrs
1	30- 39 Yrs
1	20- 29 Yrs
1	10- 19 Yrs
1	60- 69 Yrs
1	20- 29 Yrs
1	< 10 Yrs
1	40- 49 Yrs
1	40- 49 Yrs
1	50- 59 Yrs
1	70+ Yrs
1	< 10 Yrs
1	50- 59 Yrs
1	60- 69 Yrs
1	10- 19 Yrs
1	< 10 Yrs
1	70+ Yrs
1	30- 39 Yrs
1	70+ Yrs
1	< 10 Yrs
1	10- 19 Yrs
1	40- 49 Yrs
1	30- 39 Yrs
1	70+ Yrs
1	50- 59 Yrs
1	70+ Yrs
1	40- 49 Yrs
1	50- 59 Yrs
1	50- 59 Yrs
1	60- 69 Yrs
1	50- 59 Yrs
1	40- 49 Yrs
1	30- 39 Yrs
1	60- 69 Yrs
1	20- 29 Yrs
1	10- 19 Yrs
1	40- 49 Yrs
1	60- 69 Yrs
1	40- 49 Yrs
1	10- 19 Yrs
1	20- 29 Yrs
1	20- 29 Yrs
1	40- 49 Yrs
1	70+ Yrs
1	10- 19 Yrs
1	20- 29 Yrs
1	30- 39 Yrs
1	10- 19 Yrs
1	10- 19 Yrs
1	30- 39 Yrs

1	10- 19 Yrs
1	20- 29 Yrs
1	70+ Yrs
1	70+ Yrs
1	40- 49 Yrs
1	50- 59 Yrs
1	50- 59 Yrs
1	30- 39 Yrs
1	30- 39 Yrs
1	30- 39 Yrs
1	< 10 Yrs
1	40- 49 Yrs
1	70+ Yrs
1	< 10 Yrs
1	< 10 Yrs
1	60- 69 Yrs
1	40- 49 Yrs
1	70+ Yrs
1	50- 59 Yrs
